# Enantioselective Contrathermodynamic Olefin Isomerization

**DOI:** 10.1021/jacs.6c04825

**Published:** 2026-05-01

**Authors:** Bianca L. Imbriaco, Sumin Lee, Eve Yuanwei Xu, Kuo Zhao, Robert R. Knowles

**Affiliations:** Department of Chemistry, 6740 Princeton University, Princeton, New Jersey 08544, United States

## Abstract

A light-driven method for the enantioselective contrathermodynamic positional isomerization of olefins based on excited-state electron transfer is reported. Sequential oxidation and deprotonation of a tetrasubstituted enol ether generates an allylic radical which is captured by a Cr­(II) cocatalyst bearing a chiral bioxazoline (BiOX) ligand. Regio- and enantioselective protodemetalation of the nascent Cr­(III) allyl complex by methanol yields the terminal olefin product with enantioselectivities of up to 95:5 er. Kinetic and isotopic labeling studies support an enantiodetermining protodemetalation and reveal the presence of two primary KIEs within the same catalytic manifold.

## Graphical abstract

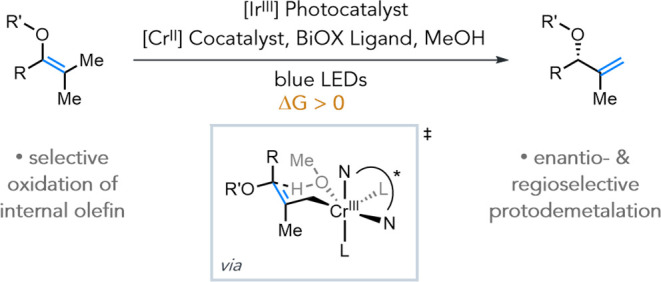

Catalytic asymmetric olefin isomerization is a powerful strategy for constructing allylic stereocenters from simple alkene starting materials.^1^ In a seminal report from 1982, Noyori and co-workers reported the enantioselective isomerization of allylamines to access optically active enamines through asymmetric hydride transfer catalyzed by a Rh-BINAP complex.^2,3^ Since then, the scope and generality of the asymmetric isomerization has been greatly expanded to include alkenyl ethers and alcohols with leading efforts from Frauenrath, Fu, Mazet, Zhao, Liu, and others.^4–10^

These thermal reactions all convert thermodynamically less stable alkene starting materials to their more stable positional isomers (Figure 1A). The reverse process, enantioselective contrathermodynamic olefin isomerization, remains a challenge in synthetic chemistry. According to the principle of microscopic reversibility, any thermal pathway capable of forming a higher-energy product must also allow for the reverse process through a common transition state, leading to an equilibrium distribution of products.^11^ Photochemistry offers a solution to this thermodynamic limitation by enabling access to excited-state pathways that can proceed in opposition to an energetic bias. Photon absorption not only supplies the external energy necessary to drive a formally “uphill” reaction but also enables elementary steps that occur on excited-state energy surfaces, thereby preventing thermal back-reaction. Leveraging this principle, several elegant strategies have been developed for asymmetric contrathermodynamic olefin isomerization through the photoexcitation of conjugated carbonyl compounds. Pioneering studies by Pete, and subsequent advances by Gilmour and Liao, established that deconjugative isomerization can proceed via substrate excitation followed by geometric isomerization, 1,5-hydrogen atom transfer (HAT) or 1,5-hydride shift, and enantioselective protonation (Figure 1B).^12–14^ While powerful, these photoenolization-based strategies require specific substrate classes and migratory-based mechanisms. This raises the question as to whether asymmetric contrathermodynamic olefin isomerization could also be achieved through alternative activation modes.^15–17^

**Figure 1:**
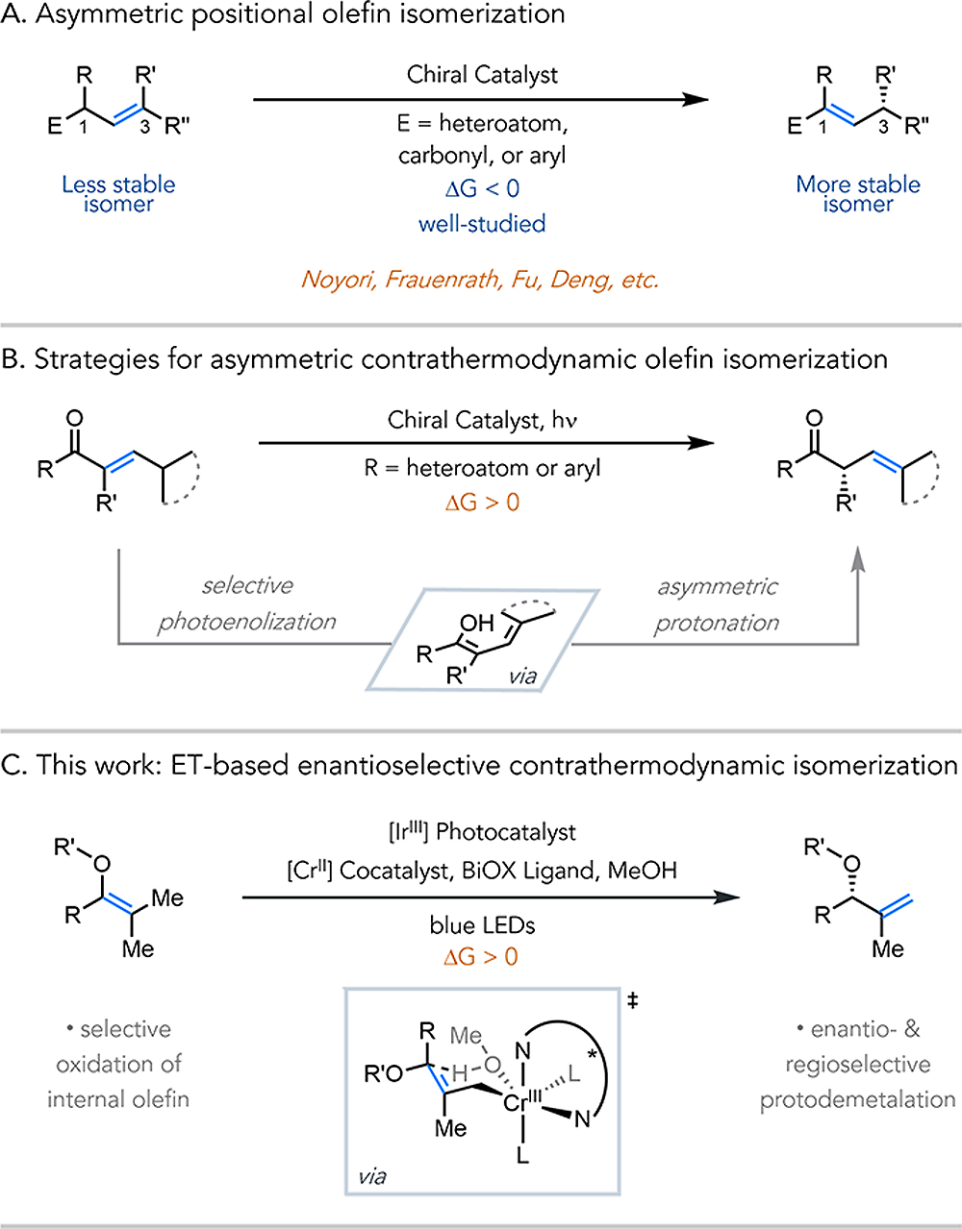
(A) Overview of asymmetric positional olefin isomerization. (B) Current strategies for asymmetric contrathermodynamic positional olefin isomerization. (C) Enantioselective contrathermodynamic positional olefin isomerization based on electron transfer activation.

Recently, our group and the Wendlandt group independently reported light-driven strategies to achieve contrathermodynamic positional isomerization of olefins using dual photoredox and transition metal catalysis.^18,19^ In our reaction, isomerization is initiated by sequential oxidation and deprotonation of an internal alkene to generate an allylic radical. Subsequent radical capture by a Cr­(II) species forms a Cr­(III) allyl intermediate. Regioselective protodemetalation then furnishes the less substituted and less stable olefin isomer. The higher oxidation potential of this product renders it inert to further reaction with the excited-state oxidant. Despite the generality of this protocol, achieving stereocontrol within such excited-state electron-transfer-driven manifold has remained elusive. However, Kanai, Wang, and Glorius have shown that allylic radicals generated under photoredox conditions can be intercepted by chiral chromium catalysts to enable highly enantioselective C–C bond formation, most notably in asymmetric allylation reactions.^20–24^ In these systems, stereoselectivity is commonly rationalized by a Zimmerman–Traxler-type transition state involving a chiral Cr­(III)–allyl intermediate and a coordinating aldehyde. We questioned whether a similar mode of stereochemical control could be translated to olefin isomerization (Figure 1C). We hypothesize that a small and weakly coordinating alcohol proton donor could coordinate to the allylchromium complex in a similar way, allowing delivery of a proton to one face of this prochiral intermediate.^25,26^

We began our investigations by evaluating chiral ligands in the isomerization of silyl enol ether **1** into allylic alcohol derivative **1a**, which is calculated to be energetically uphill by +5.5 kcal/mol (see SI for computational details). Upon blue light irradiation of an acetonitrile solution of **1** in the presence of 2 mol % [Ir­(dF­(CF_3_)­ppy)_2_(dtbbpy)]­PF_6_ (*E*_1/2_*Ir­(III)/Ir­(II) = +0.81 V vs Fc^+^/Fc),^27,28^ 10 mol % CrCl_2_, 11 mol % chiral BiOX ligand **L1**, and 1 equiv of MeOH, product **1a** was obtained in 38% yield and 94:6 er (Table 1, entry 1). Use of the more oxidizing catalyst [Ir­(dF­(CF_3_)­ppy)_2_(5,5′-d­(CF_3_)­bpy)]­PF_6_ (*E*_1/2_*Ir­(III)/Ir­(II) = +1.30 V vs Fc^+^/Fc)^18^ that was employed in our previous work led to diminished yields and selectivity (see SI for further details). In their dual chromium and photoredox system for the asymmetric allylation of aldehydes, Kanai and co-workers showed that addition of inorganic salts led to an increase in yield.^20^ Similarly, we found that addition of 20 mol % LiCl led to an increase in the reaction yield to 57% (entry 2). Addition of KCl showed a similar improvement in yield, albeit with a decrease in enantioselectivity (entry 3). These results suggested that the improved yield was likely due to the addition of chloride. By changing the PF_6_^–^ counterion of the Ir photocatalyst to Cl^–^, the reaction yielded **1a** in 56% yield and 94:6 er in the absence of LiCl (entry 4).^29^ Addition of LiCl did not further improve the yield (entry 5). On the other hand, addition of LiPF_6_ decreased yield and selectivity (entry 6), indicating that the PF_6_^–^ anion is detrimental to the reaction. Using the chloride salt of the photocatalyst, we found that lowering CrCl_2_ and ligand loading to 5 and 6 mol %, respectively, further improved yield to 62% and selectivity to 95:5 er (entry 7). Further lowering the loading of CrCl_2_ and ligand did not improve yield and an evaluation of different proton sources proved MeOH to be optimal (see SI for additional optimization details). An evaluation of additional BiOX ligands (entries 8−12) confirmed that **L1** was the optimal ligand. The absolute configuration of **1a** was determined to be (*R*) by single-crystal X-ray diffraction analysis of a ferrocene-containing ester derivative (see SI for the experimental details).

**Table 1:**
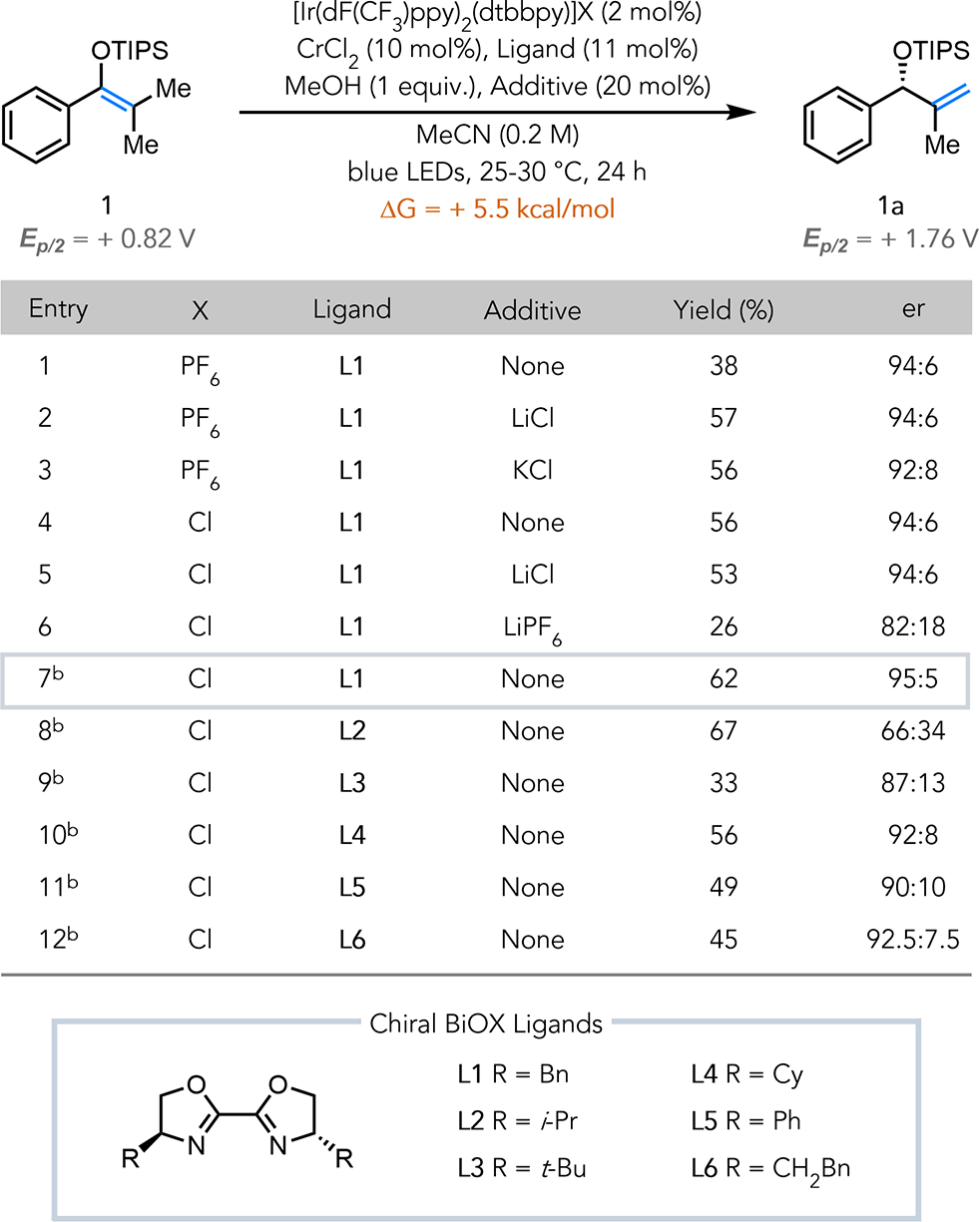
Reaction Optimization^a^

a Reactions were performed on a 0.1 mmol scale. Yields were determined via ^1^H NMR analysis of crude reaction mixtures relative to 1,3,5-trimethoxybenzene internal standard. Oxidation potentials are referenced to Fc^+^/Fc in MeCN.

b 5 mol % CrCl_2_, 6 mol % ligand.

With optimized conditions established, we sought to evaluate the scope of this method (Table 2).^30,31^ On the preparative scale, isomerization of **1** provided **1a** in 63% isolated yield and 94:6 er. The reactivity and selectivity were retained upon variation of the *para*-substituents of the arene (**2a**–**6a**). Methyl substitution at *meta*- or *ortho*-positions of the arene were tolerated, and product **7a** and **8a** were obtained with good yields and selectivities. Enol ethers containing disubstituted arenes were isomerized efficiently with good selectivities (**9a**, **10a**). The isomerization was amenable to an enol ether bearing a trisubstituted arene (**11a**), albeit with a lower selectivity. The reaction accommodated benzodioxole (**12a**), benzofuran (**13a**), and indole (**14a**, **15a**) heterocycles with moderate yields and good selectivities. Importantly, homologation of the phenyl moiety led to selective formation of the terminal alkene, albeit with lower selectivity (**16a**).

**Table 2:**
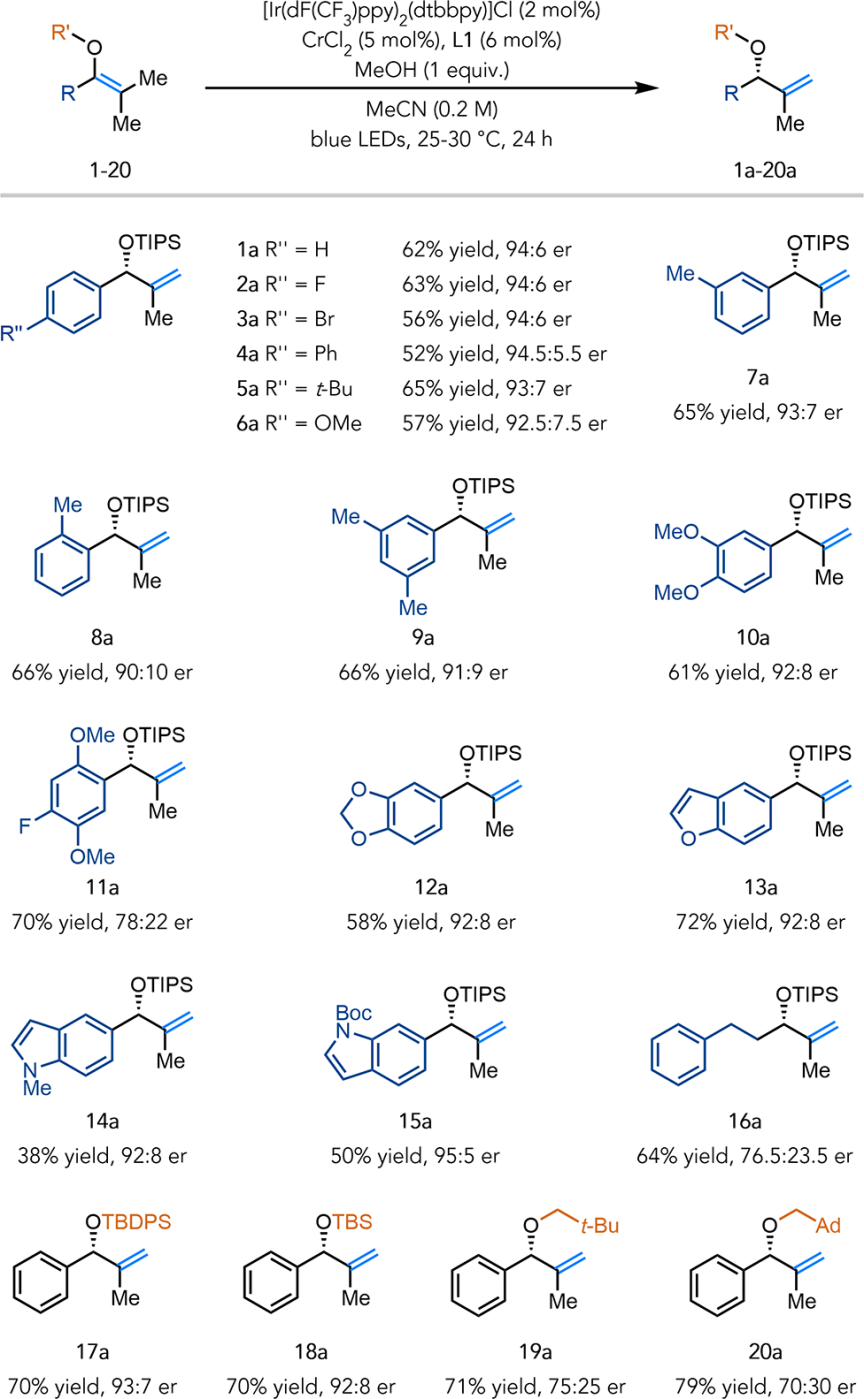
Scope of Enantioselective Olefin Isomerization^a^

a Reactions were run on a 0.5 mmol scale. Yields and enantiomeric ratio are reported for isolated products and are the average of two experiments.

We next investigated olefin isomerization on substrates bearing various ether protecting groups. TBDPS and TBS enol ethers provided the terminal olefin isomer in good yields and selectivities (**17a**, **18a**). Alkyl enol ethers underwent isomerization successfully but showed diminished selectivities (**19a**, **20a**).

Based on our prior work,^18^ we proposed the following mechanism for the enantioselective positional isomerization of olefins (Figure 2): Electron transfer (ET) between the Ir photocatalyst (*E*_1/2_*Ir­(III)/Ir­(II) = +0.81 V vs Fc^+^/Fc)^27,28^ and enol ether substrate (*E*_p/2_(**1**) = +0.82 V vs Fc^+^/Fc in MeCN) leads to the formation of an enol radical cation with enhanced acidity at the allylic C–H bond (est. p*K*_*a*_ ∼ 2.6 in MeCN, see SI for details). Deprotonation of the radical cation generates an allylic radical, which is captured by Cr­(II) to form an Cr­(III) allyl complex. Subsequent regio- and enantioselective protodemetalation via an S_E_2′ mechanism occurs through a putative chair-like transition state to yield the isomerized product. ET between Ir­(II) and the Cr complex regenerates the Cr­(II) and Ir­(III) catalysts.

**Figure 2:**
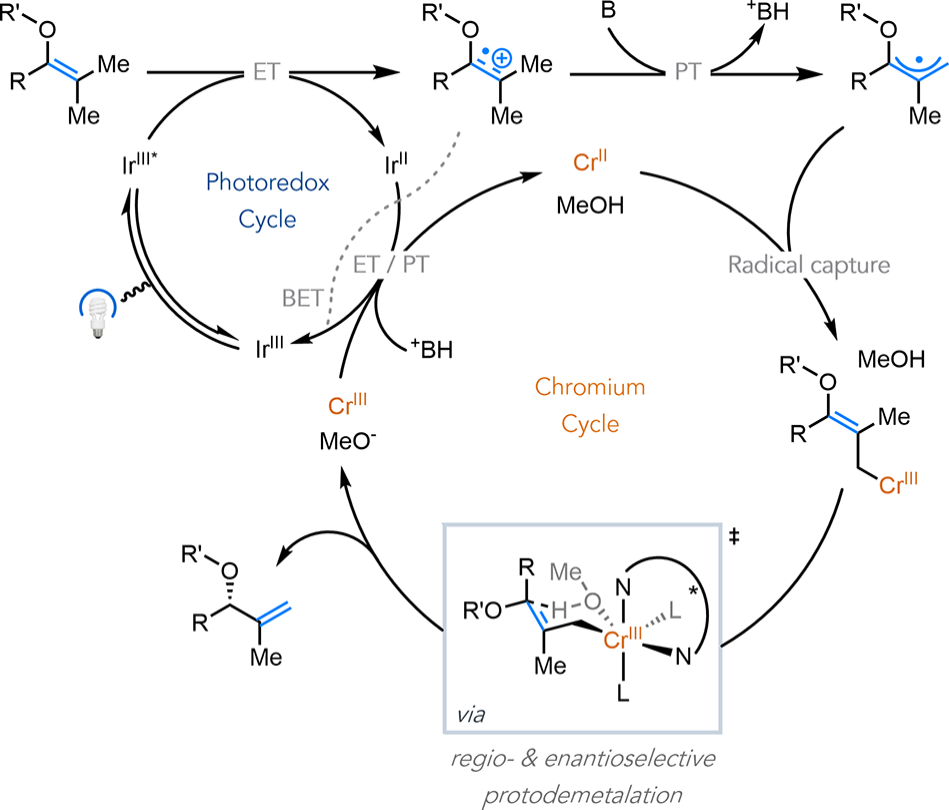
Proposed catalytic cycle. B = methoxide, solvent, ligand, and residual water.

To interrogate the loss of mass balance, we carried out kinetic experiments using substrate **2** and monitored the reaction progress using ^19^F NMR (Figure 3A). Interestingly, we found that the reaction exhibits an induction period, during which no product is formed despite the consumption of substrate. The consumption of the substrate could be attributed in part to the polymerization of **2**, as evidenced by gel permeation chromatography analysis of the crude reaction mixture (see SI for details). After the induction period, the reaction proceeds with a good mass balance, and the consumption of **2** follows first-order kinetics (Figure 3A).

**Figure 3:**
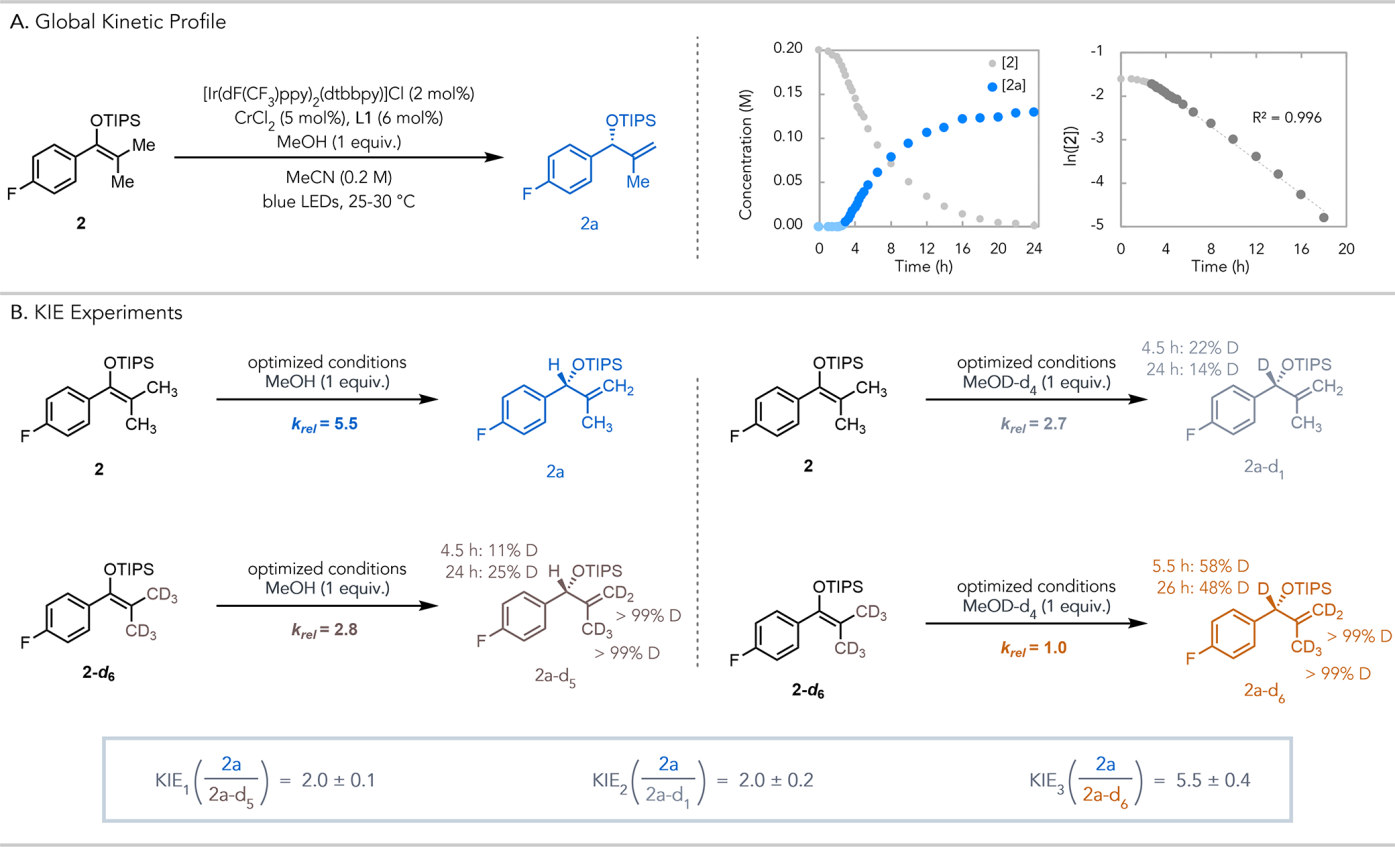
(A) Global kinetic profile shows induction period and first-order kinetic dependence on **2**. (B) Summary of parallel KIE experiments. Rates of product formation were measured after the induction period (see SI for the experimental details). KIE_1,2,3_ values 2.0 ± 0.1, 2.0 ± 0.2, and 5.5 ± 0.4 refer to the ratio between the measured rates of formation of protic product **2a** and deuterated products **2a-** ***d***_**5/1/6**_, respectively.

To shed additional light on the reaction mechanism, we conducted a series of kinetic isotope effect (KIE) experiments (Figure 3B). A set of parallel reactions were carried out to measure the reaction rate to ∼15% product formation: one with **2** and MeOH (Figure 3B, top left), and another with **2-** ***d***_**6**_ and MeOH (Figure 3B, bottom left). Comparison of the initial rates of the two experiments resulted in a *k*_H_/*k*_D_ ratio of 2.0 ± 0.1 (Figure 3B, KIE_1_). No hydrogen exchange was observed on the terminal methyl groups of recovered **2-** ***d***_**6**_, excluding the possibility of reversible proton exchange.

We also performed a separate experiment with MeOD-*d*_4_ using protiated substrate **2** (Figure 3B, top right). A k_H_/k_D_ ratio of 2.0 ± 0.2 was obtained by comparing the rates of the reactions with either MeOH or MeOD-*d*_4_ using protiated substrate **2** (Figure 3B., KIE_2_). Of note, only 22% deuterium incorporation in the benzylic position of **2a** was observed at the end of the rate measurement, and this value decreased to 14% at the end of the reaction. We partially attribute this to the formation of MeOH as the reaction proceeds due to methoxide (est. p*K*_*a*_ = 28.7 in MeCN)^32^ being sufficiently basic to deprotonate the radical cation of **2**. Such proton exchange resulted in a measured KIE value that is lower than the intrinsic isotope effect of the reaction. Other proton sources in the reaction were MeCN and residual water in the reaction solvent. When increasing MeOH or MeOD-*d*_4_ loading to 10 equiv or using MeCN-*d*_3_ as solvent, higher deuterium incorporation and KIEs were observed (see SI for additional KIE experiments).

Comparing the measured rates of the experiments performed using **2** and MeOH (Figure 3B, top left), or **2-** ***d***_**6**_ and MeOD-*d*_4_ (Figure 3B, bottom right), yielded a KIE of 5.5 ± 0.4 (Figure 3B, KIE_3_). This value is larger than the KIE when only the substrate or methanol is deuterated, showing a compounding effect of two independent steps. Together, these experiments indicate that the reaction has two steps that exhibit primary KIEs, one involving methanol and the other involving the allylic hydrogen of the substrate. The observation of two primary KIEs is well documented in biocatalytic cascade reactions.^33^ Although less common in small-molecule catalysis, interconnected catalytic cycles can similarly give rise to multiple kinetically relevant steps.^34^ Considering the growing body of literature showing the complexity in understanding full catalytic network operation, further mechanistic investigation is required to elucidate the significance of the observed KIEs.

In conclusion, we have leveraged dual photoredox and Cr catalysis to develop a method for enantioselective contrathermodynamic positional isomerization of enol ethers. Mechanistic studies are consistent with a regio- and enantioselective protodemetalation from the *in situ* generated Cr­(III) allyl complex. These findings establish a new approach to enantioselective protodemetalation of an allylchromium intermediate as a viable strategy for enantioinduction. We are optimistic that this construct will provide future opportunities for the development of asymmetric contrathermodynamic transformations.

## Supplementary Material



## Notes

Experimental details, crystallographic and computational information, NMR spectra, and HPLC traces (PDF)
