# Supplementary material for: Enantioselective Contrathermodynamic Olefin Isomerization
Source: J Am Chem Soc. 2026 May 1;148(18):18621–6. doi: 10.1021/jacs.6c04825 (PMC13185105; doi:10.1021/jacs.6c04825)
Supplement: Supplementary file 1 [file ja6c04825_si_001.pdf]

Supporting Information

Enantioselective Contrathermodynamic Olefin Isomerization

Bianca L. Imbriaco[#], Sumin Lee[#], Eve Yuanwei Xu[#], Kuo Zhao, and Robert R. Knowles*

Department of Chemistry, Princeton University, Princeton, New Jersey 08544, United States

#Denotes equal contributions

*Corresponding author. Email: rknowles@princeton.edu

Table of Contents

1. **General Information** **3**
2. **Synthesis of Starting Materials** **4**
   - Synthesis of Ketones 4
   - Synthesis and Characterization of Enol Ethers 5
3. **Synthesis of Catalysts and Ligands** **16**
   - Synthesis of Photocatalysts 16
   - Synthesis of Ligands 17
4. **Reaction Optimizations** **18**
5. **Synthesis of Products** **23**
6. **Substrates with Low Reactivity** **31**
7. **Determination of Product Absolute Configuration** **32**
   - Synthesis of Enantiopure Product & (*R*)-ferrocenyl 2-methyl-3-phenylprop-2-enoate 32
   - Crystallography Data 33
8. **GPC Analysis** **36**
9. **Mechanistic Studies** **37**
   - Kinetic Experiments 37
   - KIE Experiments 39
   - Non-Linear Effects (NLE) Study 57
   - Cyclic Voltammetry Experiments 58
10. **Computational Studies** **59**
    - pKa Calculation 59
11. **NMR Spectra** **64**
12. **HPLC Traces** **106**
13. **References** **118**

1. General Information

Commercially available reagents were purchased and used as received unless otherwise noted. Air- and moisture-sensitive reagents were stored and handled inside a N_2-filled glovebox. All reactions were performed under nitrogen or argon atmosphere unless indicated otherwise. Thin layer chromatography (TLC) was performed on Silicycle 250μm silica gel plates with indicator F-254. Visualization was accomplished with UV fluorescence and treatment with phosphomolybdic, ceric ammonium molybdate, or potassium permanganate stain. Normal-phase flash column chromatography was carried out on a Biotage Isolera One purification system with cartridges containing Silicycle F60 silica gel or Sigma-Aldrich aluminium oxide (activated, basic, Brockmann I).

^{1}H and ^{13}C NMR spectra were recorded on a Bruker 500 (500 and 126 MHz) instrument. ^{19}F NMR spectra were recorded on a Bruker 400 (376 MHz) or 500 (470 MHz) instruments. The chemical shifts are internally referenced to residual solvent signals. Chemical shifts (*δ*) are reported in parts per million (ppm), coupling constants (*J*) are reported in Hz, and multiplicity is described using the following abbreviations: singlet (s), broad (b), multiplet (m), doublet (d), triplet (t), and quartet (q). High-resolution mass spectra were obtained at Princeton University Mass Spectrometry Facility using an Agilent 6210 TOF LC/MS (Electrospray Ionization, ESI) or an Agilent 7200 Q-TOF GC/MS (Electron Ionization, EI). IR spectra were recorded on a Thermo Scientific Nicolet 6700 FT-IR spectrometer and are reported in wavenumbers (cm^{-1}). The enantiomeric ratio (er) was determined by Supercritical fluid chromatography (SFC) performed on an ColumnTek EnantioCel® A6-5 column (4.6 × 250 mm) or High-Performance Liquid Chromatography (HPLC) performed on ChiralPak/ChiralCel columns, notably ChiralPak AS-H (5 μm particle size, 4.6 mm × 250 mm, ChiralPak AD-H (5 μm particle size, 4.6 mm × 250 mm), ChiralCel OD-H (5 μm particle size, 4.6 mm × 250 mm),), ChiralCel OJ-H (5 μm particle size, 4.6 mm × 250 mm), ChiralCel OZ-H (5 μm particle size, 4.6 mm × 250 mm). Optical rotations were measured on a Jasco P-1010 polarimeter at the sodium D-line (589 nm) using a cell of 50 mm path length. The concentration values (c) are reported in g/100 mL. Gel Permeation Chromatography (GPC) spectra were obtained using a Tosoh EcoSEC HLC 8420 GPC system with Super HM-N and Super2500 columns in parallel at 40 °C and 0.20 mL/min flow rate. Tetrahydrofuran was used as the eluent and number-average molecular weight (Mn) was calculated using refractive index (RI) chromatograms against TSK gel polystyrene standards.

2. Synthesis of Starting Materials

- **Synthesis of Ketones**

General procedure A

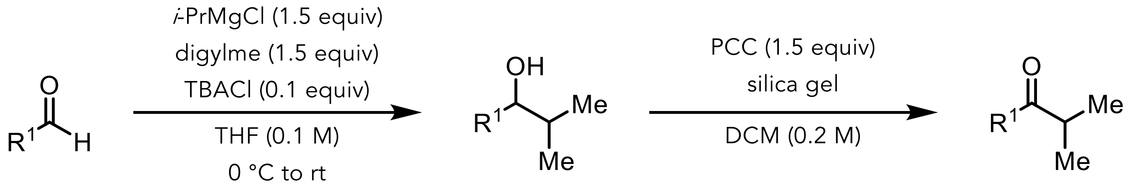

To a solution of tetrabutylammonium chloride (0.1 equiv) in THF (1.0 M), isopropylmagnesium chloride solution (2.0 M in THF, 1.5 equiv) and diethylene glycol dimethyl ether (1.5 equiv) were added dropwise at 0 °C, and the mixture was stirred for 30 min at 0 °C. Then, aldehyde (1.0 equiv) was added dropwise over 1 hour at the same temperature. The reaction mixture was slowly warmed overnight and quenched by saturated NH_4Cl solutions. The organic layer was separated and extracted with ethyl acetate (3 x 50 mL). The combined organic layers were washed with brine and dried over anhydrous sodium sulfate. After the filtration of the drying agent, the filtrate was evaporated, and the crude product was purified by silica gel column chromatography using ethyl acetate and hexanes as eluent. The resulting alcohol was dissolved in anhydrous DCM (0.2 M) at room temperature. PCC (1.5 equiv) and silica gel (1:1 wt % to PCC) were added and stirred at room temperature overnight. Diethyl ether (20 mL) was added to the reaction mixture, and the mixture was stirred for an additional 30 min. The resulting mixture was filtered through a short pad of silica gel and rinsed with diethyl ether. The filtrate was concentrated in vacuo, and the crude material was purified by column chromatography using ethyl acetate and hexanes as eluent to give the corresponding ketone.

General procedure B

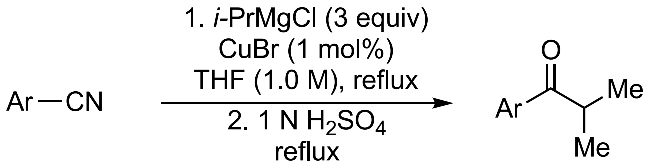

To a solution of copper bromide (1 mol%) and aryl nitrile (1 equiv) in THF (1.0 M) was added isopropyl magnesium chloride (2.0 M in THF, 3 equiv) dropwise at 0 °C. The resulting solution was refluxed for 4 hours. The solution was then cooled to 0 ºC, and water (1 mL/mmol) was added slowly. Once the vigorous reaction ceased, a 1N solution of sulfuric acid (3 mL/mmol) was added, and the resulting solution was heated to reflux for 1 hour, following which time it was cooled to room temperature, basified with 2 M NaOH until pH = 9-10. The reaction mixture was extracted with ethyl acetate (3 x 50 mL), and the combined organic extracts were washed with brine and dried over anhydrous sodium sulfate. After the filtration of the drying agent, the filtrate was evaporated, and the crude product was purified by column chromatography using ethyl acetate and hexanes as eluent to give the corresponding ketone.

General procedure C

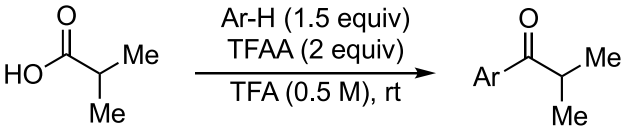

The arene (1.5 equiv) and isobutyric acid (1.0 equiv) were added to trifluoroacetic acid (0.5 M) at room temperature. Then, trifluoroacetic anhydride (2.0 equiv) was added to the reaction mixture in one portion. The resulting mixture was stirred at room temperature overnight, concentrated to a small volume, diluted with hexanes (20 mL), and washed with saturated aqueous $NaHCO_3$ solution (3 x 25 mL) to remove the residual trifluoroacetic acid. The organic layer was separated, and the aqueous layer was extracted with ethyl acetate (3 x 50 mL). The combined organic layer was dried over anhydrous sodium sulfate. After the filtration of the drying agent, the filtrate was concentrated in vacuo, and the crude material was purified by column chromatography using ethyl acetate and hexanes as eluent to give the corresponding ketone.

General procedure D

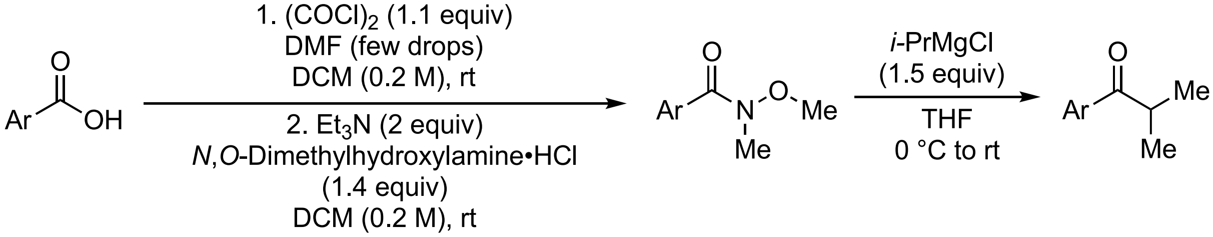

The solution of carboxylic acid (1 equiv) in DCM (0.2 M) was cooled to 0 ºC and oxalyl chloride (1.1 equiv) was added dropwise. After adding the catalytic amount of DMF (several drops), the mixture was warmed to room temperature and stirred for 3 hours. The volatiles were removed by rotary evaporator, and the crude acid chloride was dissolved in DCM (5 mL) and added dropwise to the flask containing a solution of *N*,*O*-dimethylhydroxylamine (1.4 equiv), and triethylamine (2 equiv) in DCM (0.2 M) cooled to 0 ºC. After stirring for 2 hours at room temperature, the reaction was quenched with a saturated aqueous solution of $NaHCO_3$. The organic layer was separated, and the aqueous layer was extracted (3 x 50 mL) with DCM. The combined organic layer was dried over anhydrous sodium sulfate. After the filtration of the drying agent, the filtrate was concentrated in vacuo, and the crude material was purified by column chromatography using ethyl acetate and hexanes as eluent to give the corresponding Weinreb amide. The Weinreb amide was dissolved in THF (0.2 M) and cooled to 0 ºC. Isopropyl magnesium chloride (2.0 M in THF, 1.5 equiv) was added dropwise, and the resulting solution was warmed to room temperature and stirred overnight. The reaction mixture was cooled to 0 ºC, saturated aqueous $NaHCO_3$ solution was added to quench the reaction, and ethyl acetate was added. The organic layer was separated, and the aqueous layer was extracted with ethyl acetate (3 x 50 mL). The combined organic layer was dried over Na_2SO_4. After the filtration of the drying agent, the filtrate was concentrated in vacuo, and the crude material was purified by column chromatography using ethyl acetate and hexanes as eluent to give the corresponding ketone.

• Synthesis and Characterization of Enol Ethers

General procedure E

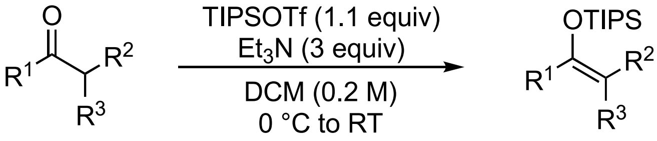

To a solution of ketone (1.0 equiv) and Et_3N (3.0 equiv) in DCM (0.5 M) was added TIPSOTf (1.1 equiv) at 0 ºC dropwise. After the mixture was stirred at room temperature overnight, the reaction was quenched with saturated aqueous $NaHCO_3$. The organic materials were extracted with Et_2O (3 x 50 mL), and the combined organic extracts were washed with brine and dried over anhydrous magnesium sulfate. After the filtration of the drying agent, the filtrate was evaporated, and the crude product was purified by silica gel column chromatography using ethyl acetate and hexanes as eluent to give the corresponding silyl enol ether.

General procedure F

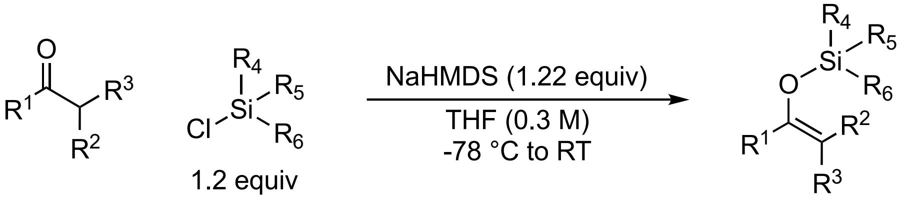

In an oven-dried flask sealed with a septum under N_2 atmosphere, ketone (1 equiv) was dissolved in anhydrous THF (0.3 M). The solution was cooled to -78 °C, and NaHMDS (1 M in THF, 1.22 equiv) was added dropwise. The cold bath is removed, and the solution was stirred for 1 hour at room temperature. The reaction was cooled again to -78 °C, and the silyl chloride (1.2 equiv) was added dropwise. The reaction mixture was warmed to room temperature and stirred until the starting ketone was fully consumed. Then, the reaction was diluted with ethyl acetate and saturated aqueous $NaHCO_3$ solution was added. The organic layer was separated, and the aqueous layer was extracted with ethyl acetate (3 x 50 mL). The combined organic extracts were washed with brine and dried over anhydrous sodium sulfate. After the filtration of the drying agent, the filtrate was evaporated, and the crude product was purified by silica gel column chromatography using ethyl acetate and hexanes as eluent to give the corresponding silyl enol ether.

General procedure G

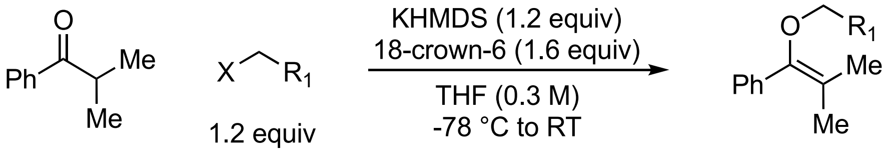

A flame-dried flask equipped with a stir bar was brought into the glovebox. KHMDS (1.2 equiv) and 18-crown-6 (1.6 equiv) were added to the flask. The flask was sealed with a septum and electric tape and brought out of the box. Under an argon balloon, anhydrous THF (0.3 M) was added to the flask. The solution was stirred for a minute at room temperature to dissolve all the solids. The solution was cooled to -78 ºC, and ketone (1 equiv, neat) was added dropwise. The cold bath was removed, and the yellow/orange solution was stirred for 1 hour at room temperature. The reaction was cooled again at -78 °C, and alkyl halide (1.2 equiv) was added dropwise. The cold bath was removed, and the solution was stirred at room temperature overnight. The reaction mixture was quenched with saturated aqueous $NaHCO_3$ solution. The mixture was diluted with diethyl ether, and the aqueous layer was extracted with diethyl ether (3 x 50 mL). The combined

organic layers were washed with brine, dried with Na_2SO_4 and concentrated. The crude material was purified with basic alumina column chromatography using hexanes as eluent to give the corresponding alkyl enol ether.

Triisopropyl((2-methyl-1-phenylprop-1-en-1-yl)oxy)silane (1)

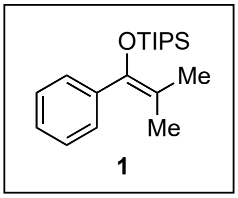

Silyl enol ether (**1**) was synthesized according to general procedure **F** from 20.0 mmol of commercially available isobutyrophenone. Colorless oil, 5.73 g, 94% yield. **^{1}H NMR (500 MHz, CDCl$_3$)** δ 7.33 – 7.27 (m, 4H), 7.26 – 7.21 (m, 1H), 1.82 (s, 3H), 1.57 (s, 3H), 0.98 – 0.91 (m, 21H). **^{13}C NMR (126 MHz, CDCl$_3$)** δ 144.29, 139.49, 129.55, 127.72, 127.31, 111.76, 20.11, 18.35, 18.03, 13.27. **IR (cm^{-1})** ν 2944, 2866, 2358, 2339, 1671, 1464, 1293, 1161, 883, 762, 201, 682. **HRMS (EI):** m/z [$C_{19}H_{32}OSi$]$^{\bullet+}$ ([M]$^{\bullet+}$) calculated: 304.22169; found: 304.22079.

Triisopropyl((1-(4-fluorophenyl)-2-methylprop-1-en-1-yl)oxy)silane (2)

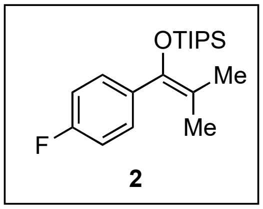

The ketone precursor was synthesized according to general procedure **A** from commercially available 4-F-benzaldehyde. The spectral data are consistent with literature values.[1] Silyl enol ether (**2**) was synthesized according to general procedure **F** from 8.42 mmol of the corresponding ketone. Colorless oil, 2.16 g, 80% yield. **^{1}H NMR (500 MHz, CDCl$_3$)** δ 7.31 – 7.24 (m, 2H), 7.02 – 6.96 (m, 2H), 1.81 (s, 3H), 1.55 (s, 3H), 1.01 – 0.87 (m, 21H). **^{13}C NMR (126 MHz, CDCl$_3$)** δ 161.98 (d, J = 246.0 Hz), 143.29, 135.57 (d, J = 3.4 Hz), 131.15 (d, J = 8.0 Hz), 114.66 (d, J = 21.2 Hz), 112.09, 20.07, 18.33, 18.02, 13.25. **^{19}F NMR (376 MHz, CDCl$_3$)** δ -114.72 – -114.80 (m). **IR (cm^{-1})** ν 2944, 2867, 2358, 1601, 1508, 1464, 1291, 1225, 1154, 882, 682. **HRMS (EI):** m/z [$C_{19}H_{31}FOSi$]$^{\bullet+}$ ([M]$^{\bullet+}$) calculated: 322.21227; found: 322.21225.

Triisopropyl((1-(4-fluorophenyl)-2-(methyl-*d*$_3$)prop-1-en-1-yl-3,3,3-*d*$_3$)oxy)silane (2-*d*$_6$)

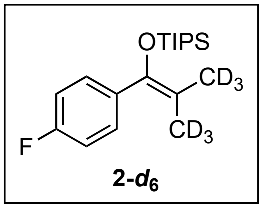

The ketone precursor was synthesized according to general procedure **A** from commercially available 4-F-benzaldehyde and Grignard reagent prepared from isopropybromide-*d*$_7$. Silyl enol ether (**2-*d*$_6$**) was synthesized according to general procedure **F** from the corresponding ketone. Colorless oil. **^{1}H NMR (500 MHz, CDCl$_3$)** δ 7.32 – 7.24 (m, 2H), 7.03 – 6.95 (m, 2H), 1.10 – 0.87 (m, 21H). **^{13}C NMR (126 MHz, CDCl$_3$, CD$_3$ signals are low and unassigned)** δ 162.00 (d, J = 246.0 Hz), 143.37, 135.61 (d, J = 3.4 Hz), 131.15 (d, J = 7.9 Hz), 114.66 (d, J = 21.2 Hz), 111.83, 18.02, 13.27. **^{19}F NMR (471 MHz, CDCl$_3$)** δ -114.67 – -114.89 (m). **IR (neat, cm^{-1})** ν 2945, 2892, 2867, 2355, 1739, 1603, 1508, 1464, 1366, 1299, 1229,

1152, 1076, 1067, 882, 853, 806, 680. **HRMS (EI):** m/z [$C_{19}H_{25}D_6FOSi$]$^{•+}$ ([M]$^{•+}$) calculated: 328.24993; found: 328.24962.

Triisopropyl((1-(4-bromophenyl)-2-methylprop-1-en-1-yl)oxy)silane (3)

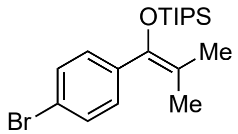

3

The ketone precursor was synthesized according to general procedure **A** from commercially available 4-bromobenzaldehyde. The spectral data are consistent with literature values.[2] Silyl enol ether (**3**) was synthesized according to general procedure **F** from 5.28 mmol of the corresponding ketone. Colorless oil, 1.73 g, 85% yield. **^{1}H NMR (500 MHz, CDCl$_3$)** δ 7.45 – 7.41 (m, 2H), 7.21 – 7.16 (m, 2H), 1.80 (s, 3H), 1.56 (s, 3H), 1.00 – 0.87 (m, 21H). **^{13}C NMR (126 MHz, CDCl$_3$)** δ 143.18, 138.40, 131.15, 130.91, 121.15, 112.67, 20.08, 18.43, 18.02, 13.26. **IR (cm^{-1})** ν 2943, 2866, 2161, 2027, 2007, 1974, 1463, 1289, 1162, 1070, 1038, 1011, 883, 844, 682. **HRMS (EI):** m/z [$C_{19}H_{31}BrOSi$]$^{•+}$ ([M]$^{•+}$) calculated: 382.13221, 384.13016; found: 382.12970, 384.12710.

Triisopropyl((1-([1,1'-biphenyl]-4-yl)-2-methylprop-1-en-1-yl)oxy)silane (4)

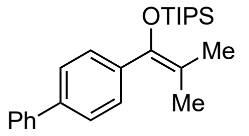

4

The ketone precursor was synthesized according to general procedure **B** from 10.0 mmol of commercially available 4-phenylbenzonitrile. White solid, 2.19 g, 98% yield. The spectral data are consistent with literature values.[3] Silyl enol ether (**4**) was synthesized according to general procedure **F** from 2.68 mmol of the corresponding ketone. White solid. 2.19 g, 98% yield. **^{1}H NMR (500 MHz, CDCl$_3$)** δ 7.66 – 7.59 (m, 2H), 7.58 – 7.51 (m, 2H), 7.47 – 7.41 (m, 2H), 7.40 – 7.31 (m, 3H), 1.85 (s, 3H), 1.63 (s, 3H), 1.00 – 0.94 (m, 21H). **^{13}C NMR (126 MHz, CDCl$_3$)** δ 144.00, 140.97, 139.88, 138.47, 129.92, 128.89, 127.37, 127.12, 126.34, 112.15, 20.22, 18.46, 18.06, 13.30. **IR (cm^{-1})** ν 2943, 2865, 2217, 1485, 1463, 1292, 1159, 853, 682. **HRMS (EI):** m/z [$C_{25}H_{36}OSi$]$^{•+}$ ([M]$^{•+}$) calculated: 380.25299; found: 380.25264.

Triisopropyl((1-(4-(tert-butyl)phenyl)-2-methylprop-1-en-1-yl)oxy)silane (5)

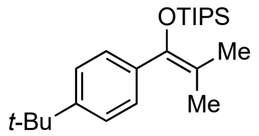

5

The ketone precursor was synthesized according to general procedure **D** from commercially available 4-*tert*-butylbenzoic acid. Colorless oil, 896 mg, 81% yield (over two steps, using *i*-PrLi instead of *i*-PrMgCl).

The spectral data are consistent with literature values.[4] Silyl enol ether (**5**) was synthesized according to general procedure **F** from 3.00 mmol of the corresponding ketone. Colorless oil, 840 mg, 78% yield. **^{1}H NMR (500 MHz, CDCl$_3$)** δ 7.33 – 7.27 (m, 2H), 7.25 – 7.19 (m, 2H), 1.81 (s, 3H), 1.58 (s, 3H), 1.31 (s, 9H), 0.98 – 0.92 (m, 21H). **^{13}C NMR (126 MHz, CDCl$_3$)** δ 150.24, 144.32, 136.54, 129.18, 124.55, 111.30, 34.67, 31.48, 20.18, 18.29, 17.99, 13.26. **IR (cm^{-1})** ν 2945, 2866, 2361, 2337, 1669, 1507, 1463, 1293, 1158, 1043, 1014, 882, 681, 655. **HRMS (EI):** m/z [$C_{23}H_{40}OSi$]$^{\bullet+}$ ([M]$^{\bullet+}$) calculated: 360.28429; found: 360.28235.

Triisopropyl((1-(4-methoxyphenyl)-2-methylprop-1-en-1-yl)oxy)silane (6)

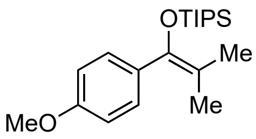

6

The ketone precursor was synthesized according to general procedure **C** from 20 mmol of isobutyric acid. Colorless oil, 3.55 g, 19.9 mmol, 99% yield. The spectral data are consistent with literature values.[5] Silyl enol ether (**6**) was synthesized according to general procedure **F** from 9.50 mmol of the corresponding ketone. Colorless oil, 867 mg, 27% yield. **^{1}H NMR (500 MHz, CDCl$_3$)** δ 7.25 – 7.19 (m, 2H), 6.86 – 6.80 (m, 2H), 3.81 (s, 3H), 1.81 (s, 3H), 1.56 (s, 3H), 1.02 – 0.88 (m, 21H). **^{13}C NMR (126 MHz, CDCl$_3$)** δ 158.72, 143.99, 132.04, 130.71, 113.02, 111.27, 55.35, 20.16, 18.35, 18.07, 13.28. **IR (cm^{-1})** ν 2945, 2866, 1667, 1608, 1510, 1464, 1299, 1285, 1246, 1158, 1047, 882, 845, 682. **HRMS (EI):** m/z [$C_{20}H_{34}O_2Si$]$^{\bullet+}$ ([M]$^{\bullet+}$) calculated: 334.23226; found: 334.23132.

Triisopropyl((2-methyl-1-(*m*-tolyl)prop-1-en-1-yl)oxy)silane (7)

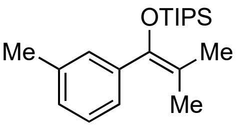

7

The ketone precursor was synthesized according to general procedure **D** from 12.3 mmol of the Weinreb amide prepared from commercially available 3-methylbenzoic acid. Colorless oil, 740 mg, 37% yield. The spectral data are consistent with literature values.[6] Silyl enol ether (**7**) was synthesized according to general procedure **F** from 4.31 mmol of the corresponding ketone. Colorless oil, 1.05 g, 76% yield. **^{1}H NMR (500 MHz, CDCl$_3$)** δ 7.23 – 7.13 (m, 2H), 7.11 – 7.07 (m, 1H), 7.07 – 7.03 (m, 1H) 2.33 (s, 3H), 1.81 (s, 3H), 1.58 (s, 3H), 1.00 – 0.86 (m, 21H). **^{13}C NMR (126 MHz, CDCl$_3$)** δ 144.37, 139.32, 137.18, 130.23, 127.97, 127.49, 126.66, 111.51, 21.53, 20.14, 18.34, 18.03, 13.24. **IR (cm^{-1})** ν 2944, 2924, 2866, 1463, 1300, 1186, 1152, 1053, 882, 835, 810, 709, 681. **HRMS (EI):** m/z [$C_{20}H_{34}OSi$]$^{\bullet+}$ ([M]$^{\bullet+}$) calculated: 318.23734; found: 318.23657.

Triisopropyl((2-methyl-1-(*o*-tolyl)prop-1-en-1-yl)oxy)silane (8)

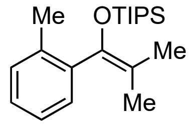

8

The ketone precursor was synthesized according to general procedure **B** from 15.0 mmol of commercially available 2-methylbenzonitrile. Yellow oil, 2.42 g, 99% yield. The spectral data are consistent with literature values.[6] Silyl enol ether (**8**) was synthesized according to general procedure **F** from 8.32 mmol of the corresponding ketone. Colorless oil, 2.65 g, 70% yield. **^{1}H NMR (500 MHz, CDCl$_3$)** δ 7.18 – 7.08 (m, 4H), 2.28 (s, 3H), 1.81 (s, 3H), 1.39 (s, 3H), 1.02 – 0.85 (m, 21H). **^{13}C NMR (126 MHz, CDCl$_3$)** δ 143.53, 139.20, 137.45, 130.03, 130.02, 127.60, 125.15, 111.39, 19.64, 18.01, 17.93, 17.61, 13.22. **IR (cm^{-1})** ν 2943, 2923, 2866, 1673, 1463, 1382, 1293, 1156, 1032, 907, 882, 833, 729, 682, 669, 652. **HRMS (EI):** m/z $[C_{20}H_{34}OSi]^{\bullet+}$ ($[M]^{\bullet+}$) calculated: 318.23734; found: 318.23514.

Triisopropyl((1-(3,5-dimethylphenyl)-2-methylprop-1-en-1-yl)oxy)silane (9)

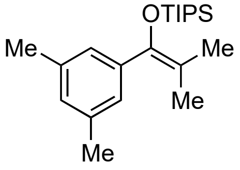

9

The ketone precursor was synthesized according to general procedure **D** from 11.0 mmol of the Weinreb amide prepared from commercially available 3,5-dimethylbenzoic acid. Silyl enol ether (**9**) was synthesized according to general procedure **F** from 3.0 mmol of the corresponding ketone. Colorless oil, 931 mg, 93% yield. **^{1}H NMR (500 MHz, CDCl$_3$)** δ 6.92 (s, 2H), 6.87 (s, 1H), 2.29 (s, 6H), 1.81 (s, 3H), 1.59 (s, 3H), 1.03 – 0.88 (m, 21H). **^{13}C NMR (126 MHz, CDCl$_3$)** δ 144.52, 139.22, 136.94, 128.77, 127.38, 111.19, 21.41, 20.17, 18.33, 18.04, 13.25. **IR (cm^{-1})** ν 2944, 2923, 2866, 2358, 2338, 1975, 1966, 1671, 1601, 1464, 1382, 1321, 1246, 1192, 1153, 1070, 999, 939, 903, 884, 858, 779, 711, 681. **HRMS (ESI):** m/z $[C_{21}H_{37}OSi]^+$ ($[M+H]^+$) calculated: 333.26082; found: 333.26220.

Triisopropyl((1-(3,4-dimethoxyphenyl)-2-methylprop-1-en-1-yl)oxy)silane (10)

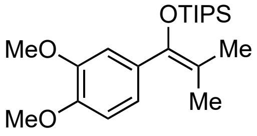

10

The ketone precursor was synthesized according to general procedure **C** from 20 mmol of isobutyric acid. Colorless oil, 3.58 g, 17.2 mmol, 86% yield. Silyl enol ether (**10**) was synthesized according to general procedure **E** from 10.0 mmol of the corresponding ketone. White solid, 2.47 g, 77% yield. **^{1}H NMR (500 MHz, CDCl$_3$)** δ 6.89 (d, J = 1.9 Hz, 1H), 6.83 (dd, J = 8.2, 1.9 Hz, 1H), 6.79 (d, J = 8.2 Hz, 1H), 3.89 (s, 3H), 3.86 (s, 3H), 1.81 (s, 3H), 1.59 (s, 3H), 1.00 – 0.94 (m, 21H). **^{13}C NMR (126 MHz, CDCl$_3$)** δ 148.20, 148.18, 144.05, 132.15, 122.19, 112.56, 111.53, 110.06, 55.93, 55.87, 20.22, 18.37, 18.11, 13.30. **IR (cm^{-}**

[1]) ν 2944, 2866, 2361, 2337, 1669, 1513, 1463, 1252, 1173, 1136, 1031, 882, 869, 820, 688. **HRMS (EI):** m/z $[C_{21}H_{36}O_3Si]^{\bullet+}$ ($[M]^{\bullet+}$) calculated: 364.24282; found: 364.24124.

Triisopropyl((1-(4-fluoro-2,5-dimethoxyphenyl)-2-methylprop-1-en-1-yl)oxy)silane (11)

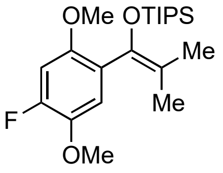

11

Silyl enol ether (**11**) was synthesized following a literature procedure.[7] The spectral data are consistent with literature values.

Triisopropyl((1-(benzo[*d*][1,3]dioxol-5-yl)-2-methylprop-1-en-1-yl)oxy)silane (12)

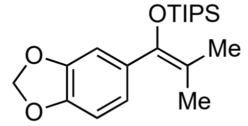

12

The ketone precursor was synthesized according to general procedure **C** from 20 mmol of isobutyric acid. Colorless oil, 3.45 g, 90% yield. The spectral data are consistent with literature values.[8] Silyl enol ether (**12**) was synthesized according to general procedure **E** from 5.0 mmol of the corresponding ketone. Colorless oil, 1.60 g, 92% yield. **^{1}H NMR (500 MHz, CDCl$_3$)** δ 6.81 – 6.72 (m, 3H), 5.95 (s, 2H), 1.79 (s, 3H), 1.56 (s, 3H), 1.03 – 0.91 (m, 21H). **^{13}C NMR (126 MHz, CDCl$_3$)** δ 146.98, 146.62, 143.82, 133.52, 123.18, 111.51, 110.07, 107.65, 101.00, 20.18, 18.35, 18.07, 13.29. **IR (cm^{-1})** ν 2944, 2867, 2355, 2340, 1486, 1435, 1288, 1236, 1208, 1163, 1042, 933, 882, 829, 680. **HRMS (ESI):** m/z $[C_{20}H_{33}O_3Si]^+$ ($[M+H]^+$) calculated: 349.21935; found: 349.22036.

Triisopropyl((1-(benzofuran-5-yl)-2-methylprop-1-en-1-yl)oxy)silane (13)

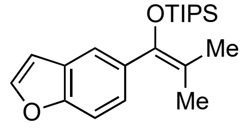

13

The ketone precursor was synthesized according to general procedure **B** from 10.0 mmol of commercially available benzofuran-5-carbonitrile. Yellow oil, 1.88 g, 51% yield. Silyl enol ether (**13**) was synthesized according to general procedure **F** from 3.00 mmol of the corresponding ketone. Colorless oil, 740.2 mg, 72% yield. **^{1}H NMR (500 MHz, CDCl$_3$)** δ 7.62 (d, J = 2.2 Hz, 1H), 7.52 (d, J = 1.6 Hz, 1H), 7.43 (d, J = 8.6 Hz, 1H), 7.27 (dd, J = 8.6, 1.6 Hz, 1H), 6.76 (d, J = 2.2 Hz, 1H), 1.85 (s, 3H), 1.57 (s, 3H), 1.02 – 0.87 (m, 21H). **^{13}C NMR (126 MHz, CDCl$_3$)** δ 154.26, 145.29, 144.39, 134.37, 126.85, 126.23, 122.20, 111.50, 110.60, 106.88, 20.24, 18.37, 18.06, 13.28. **IR (cm^{-1})** ν 2943, 2865, 2355, 2340, 1464, 1291, 1210, 1166,

1140, 1130, 1108, 1032, 883, 831, 768, 731, 680. **HRMS (ESI):** m/z $[C_{21}H_{33}O_2Si]^+$ ($[M+H]^+$) calculated: 345.22443; found: 345.22447.

1-Methyl-5-(2-methyl-1-((triisopropylsilyl)oxy)prop-1-en-1-yl)-1*H*-indole (14)

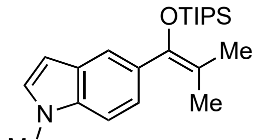

14

The ketone precursor was synthesized according to general procedure **B** from 9.2 mmol of commercially available 1-methyl-1*H*-indole-5-carbonitrile. Colorless oil, 1.86 g, 2.12 mmol, 23% yield. Silyl enol ether (**14**) was synthesized according to general procedure **F** from 2.12 mmol of the corresponding ketone. Colorless oil, 700.0 mg, 92% yield. **^{1}H NMR (300 MHz, CDCl$_3$)** δ 7.57 – 7.51 (m, 1H), 7.30 – 7.15 (m, 2H), 7.04 (d, J = 3.1 Hz, 1H), 6.51 – 6.43 (m, 1H), 3.80 (s, 3H), 1.86 (s, 3H), 1.60 (s, 3H), 1.05 – 0.90 (m, 21H). **^{13}C NMR (126 MHz, CDCl$_3$)** δ 145.37, 136.07, 130.65, 129.02, 127.79, 123.71, 122.01, 110.71, 108.28, 101.29, 33.02, 20.37, 18.44, 18.13, 13.31. **IR (cm^{-1})** ν 2943, 2866, 2355, 2340, 1670, 1514, 1490, 1464, 1423, 1334, 1242, 1176, 1144, 1039, 883, 833. 719, 682. **HRMS (ESI):** m/z $[C_{22}H_{36}NOSi]^+$ ($[M+H]^+$) calculated: 358.25607; found 358.25650.

***tert*-Butyl 6-(2-methyl-1-((triisopropylsilyl)oxy)prop-1-en-1-yl)-1*H*-indole-1-carboxylate (15)**

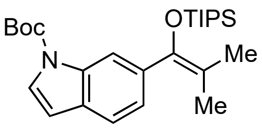

15

Silyl enol ether (**15**) was synthesized following a literature procedure.[7] The spectral data are consistent with literature values.

Triisopropyl((2-methyl-5-phenylpent-2-en-3-yl)oxy)silane (16)

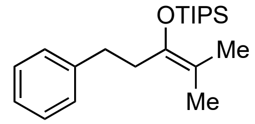

16

Silyl enol ether (**16**) was synthesized following a literature procedure.[9] The spectral data are consistent with literature values.

***tert*-Butyl((2-methyl-1-phenylprop-1-en-1-yl)oxy)diphenylsilane (17)**

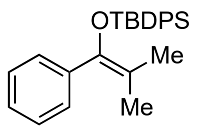

17

Silyl enol ether (**17**) was synthesized according to general procedure **F** from 5.0 mmol of commercially available isobutyrophenone. Colorless oil, 574.0 mg, 1.48 mmol, 30% yield. **^{1}H NMR (500 MHz, $CDCl_3$)** δ 7.52 (d, J = 7.9 Hz, 4H), 7.35 (s, 2H), 7.26 (m, 4H), 7.07 – 6.93 (m, 5H), 1.73 (s, 3H), 1.48 (s, 3H), 0.98 (s, 9H). **^{13}C NMR (126 MHz, $CDCl_3$)** δ 144.11, 138.28, 135.82, 134.21, 129.80, 129.37, 127.45, 127.30, 127.05, 112.32, 27.07, 19.93, 19.74, 18.47. **IR (cm^{-1})** ν 2930, 2857, 2355, 2340, 1428, 1152, 1112, 841, 699, 678, 504. **HRMS (ESI):** m/z $[C_{26}H_{31}OSi]^+$ ($[M+H]^+$) requires m/z 387.21387; found: 387.2144.

***tert*-Butyldimethyl((2-methyl-1-phenylprop-1-en-1-yl)oxy)silane (18)**

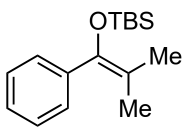

18

Silyl enol ether (**18**) was synthesized according to general procedure **F** from 10.0 mmol of commercially available isobutyrophenone. Colorless oil, 2.15 g, 82% yield. **^{1}H NMR (500 MHz, $CDCl_3$)** δ 7.53 – 7.42 (m, 5H), 2.02 (s, 3H), 1.86 (s, 3H), 1.11 (s, 9H), 0.02 (s, 6H). **^{13}C NMR (126 MHz, $CDCl_3$)** δ 143.80, 139.36, 129.47, 127.76, 127.23, 112.66, 25.95, 20.01, 18.36, 18.34, -4.18. **IR (cm^{-1})** ν 2956, 2928, 2857, 1670, 1472, 1463, 1388, 1361, 1294, 1281, 1251, 1218, 1150, 1072, 1045, 1023, 1006, 919, 894, 854, 837, 778, 701. **HRMS (ESI):** m/z $[C_{16}H_{27}OSi]^+$ ($[M+H]^+$) calculated: 263.18257; found: 263.1822.

(2-Methyl-1-(neopentyloxy)prop-1-en-1-yl)benzene (19)

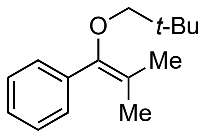

19

Alkyl enol ether (**19**) was synthesized according to general procedure **G** from 5 mmol of commercially available isobutyrophenone and 1-iodo-2,2-dimethylpropane. Colorless oil, 0.497 g, 46% yield. **^{1}H NMR (500 MHz, $CDCl_3$)** δ 7.37 – 7.30 (m, 4H), 7.29 – 7.24 (m, 1H), 3.02 (s, 2H), 1.87 (s, 3H), 1.68 (s, 3H), 0.94 (s, 9H). **^{13}C NMR (126 MHz, $CDCl_3$)** δ 148.33, 136.33, 129.67, 127.94, 127.32, 115.66, 79.64, 32.26, 26.84, 19.90, 17.81. **IR (cm^{-1})** ν 2953, 2905, 2859, 1670, 1599, 1576, 1476, 1490, 1457, 1443, 1398, 1381, 1362, 1288, 1213, 1139, 1072, 1048, 1025, 995, 923, 786, 758, 708, 698. **HRMS (EI):** m/z $[C_{15}H_{22}O]^{\bullet+}$ ($[M]^{\bullet+}$) calculated: 218.16652; found: 218.16698.

1-(((2-Methyl-1-phenylprop-1-en-1-yl)oxy)methyl)adamantane (20)

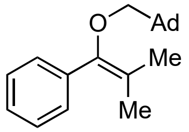

20

Alkyl enol ether (**20**) was synthesized according to general procedure **G** from 3.5 mmol of commercially available isobutyrophenone and 1-(bromomethyl)adamantane. Colorless oil, 0.309 g, 30% yield. **^{1}H NMR (500 MHz, $CDCl_3$)** δ 7.38 – 7.29 (m, 4H), 7.28 – 7.24 (m, 1H), 2.93 (s, 2H), 1.97 (h, J = 2.3 Hz, 3H), 1.85 (s, 3H), 1.75 – 1.63 (m, 9H), 1.58 (d, J = 2.9 Hz, 6H). **^{13}C NMR (126 MHz, $CDCl_3$)** δ 148.31, 136.31, 129.65, 127.91, 127.29, 115.81, 80.02, 39.72, 37.36, 34.24, 28.44, 19.91, 17.85. **IR (cm^{-1})** ν 2899, 2846, 1668, 1600, 1490, 1444, 1381, 1363, 1344, 1290, 1276. 1212, 1140, 1045, 1027, 784, 774, 733, 699. **HRMS (EI):** m/z $[C_{21}H_{28}O]^{\bullet+}$ ($[M]^{\bullet+}$) calculated: m/z 296.21347; found: 296.21411.

3. Synthesis of Catalysts and Ligands

- **Synthesis of Photocatalysts**

[Ir(dF(CF3)ppy)2(5,5'-d(CF3)bpy)]PF6 ([Ir-A]PF6)

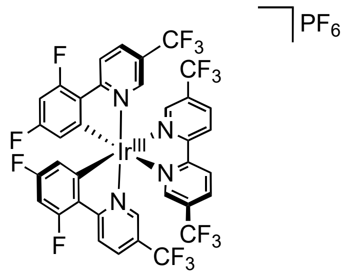

[Ir-A]PF6

[Ir-A]PF_6 was synthesized following a literature procedure.[10] The spectral data are consistent with literature values.

[Ir(dF(CF3)ppy)2(4,4'-dtbbpy)]PF6 ([Ir-B]PF6)

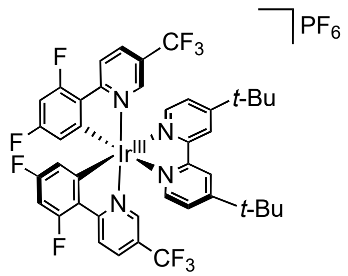

[Ir-B]PF6

[Ir-B]PF_6 was synthesized following a literature procedure.[11] The spectral data are consistent with literature values.

[Ir(dF(CF3)ppy)2(4,4'-dtbbpy)]Cl ([Ir-B]Cl)

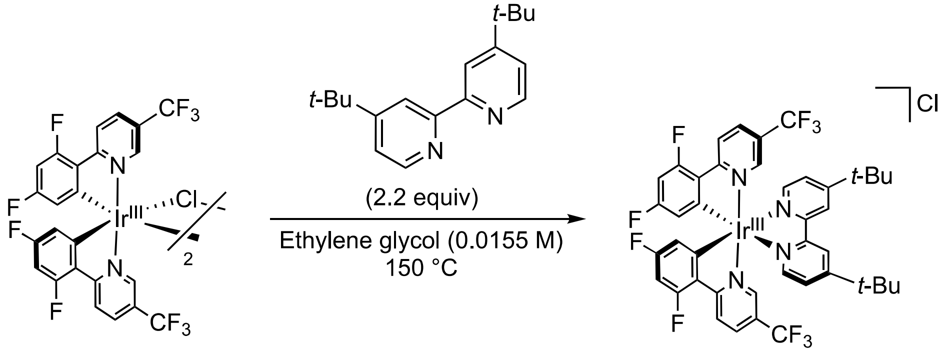

[(dF(CF_3)ppy)$_2$-Ir-μ-Cl]$_2$ was prepared following a literature procedure and used without purification.[10] In a flask equipped with a reflux condenser and a stir bar, [(dF(CF_3)ppy)$_2$-Ir-μ-Cl]$_2$ (1 equiv) and 4-*tert*-butyl-2-(4-*tert*-butyl-2-pyridyl)pyridine (2.2 equiv) were added. The flask was evacuated and backfilled with

nitrogen three times. Ethylene glycol (0.0155 M) was added. The resulting solution was heated to 150 ºC overnight. After the reaction mixture was cooled to room temp, the solution was partitioned between dichloromethane and water. The organic layer was washed with copious amounts of water to remove ethylene glycol. The organic layer was dried with Na_2SO_4 and concentrated. The crude material was recrystallized from dichloromethane/pentane. The spectral data are consistent with literature values.[12]

- **Synthesis of Ligands**

L1-15 are commercially available.

(4*S*,4'*S*)-4,4'-diphenethyl-4,4',5,5'-tetrahydro-2,2'-bioxazole (L6)

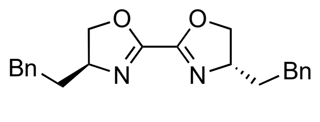

L6

L6 was synthesized following a literature procedure.[13] The spectral data are consistent with literature values.

4. Reaction Optimizations

Reaction optimization was conducted according to the following procedure: Inside a N_2-filled glove box, a stock solution was prepared with $CrCl_2$, ligand, photocatalyst, and alcohol in degassed anhydrous solvent. An oven-dried 2-dram vial equipped with a magnetic stir bar was charged with enol ether **1** (0.1 mmol, 1.00 equiv), stock solution, and additives (if applicable). The vial was sealed with a cap fitted with a PTFE septum. Electrical tape was used to seal the sides of the cap. The vial was removed from the glovebox and placed approximately 2.5 cm away from 34W Kessil PR160-456 nm blue LED lamps (25-100% intensity) on a stir plate (see Figure S1). The reaction mixture was stirred and irradiated for 24 hours. During irradiation, two rotary fans were placed adjacent to the lamps to cool the reaction setup. The crude reaction mixture was diluted with ethyl acetate and filtered through a pipette silica plug eluting with ethyl acetate. The filtrate was concentrated under reduced pressure. Yields were assessed by quantitative ^{1}H NMR spectroscopy using 1,3,5-trimethoxybenzene as an internal standard.

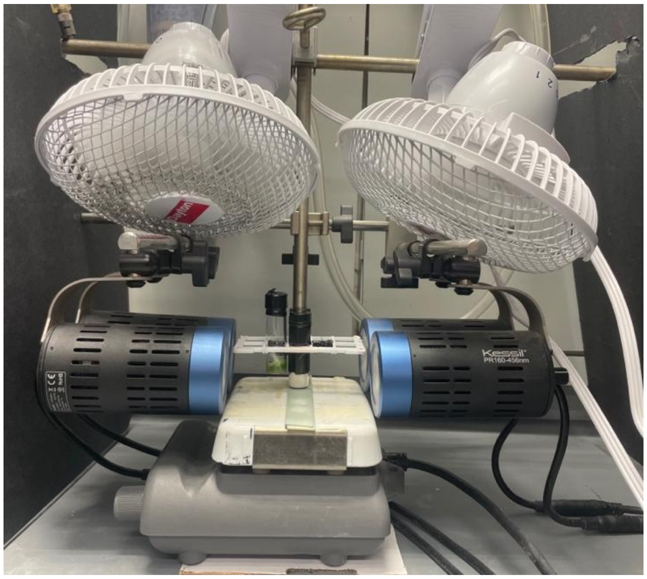

Figure S1. Typical reaction setup

Table S1. Preliminary Chiral Ligand Screen.

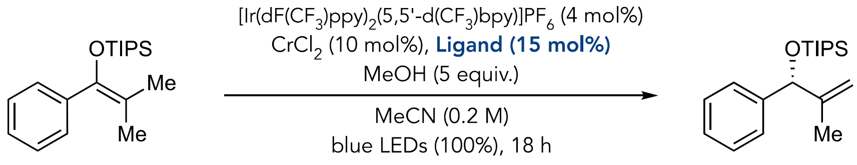

1 **1a**

Entry	Ligand	RSM (%)	Yield 1a (%)	e.r.
1	**L1**	<2	20	89:11
2	**L2**	<1	10	70.5:29.5
3	**L3**	6	9	82:18
4	**L4**	3	23	86:14
5	**L5**	5	18	89:11
6	**L6**	<5	7	85.5:14.5
7	**L7**	<1	14	89:11
8	**L8**	<2	17	38:62
9	**L9**	14	<1	55:45
10	**L10**	43	<1	69:31
11	**L11**	47	<1	67:33
12	**L12**	<1	29	56:44
13	**L13**	4	17	56.5:43.5
14	**L14**	13	1	78.5:21.5
15	**L15**	18	<2	69.5:30.5

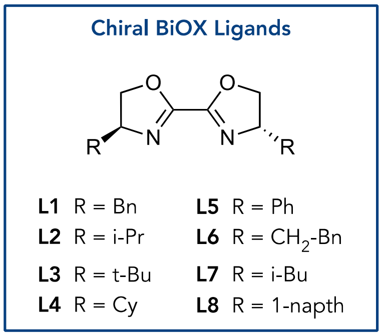

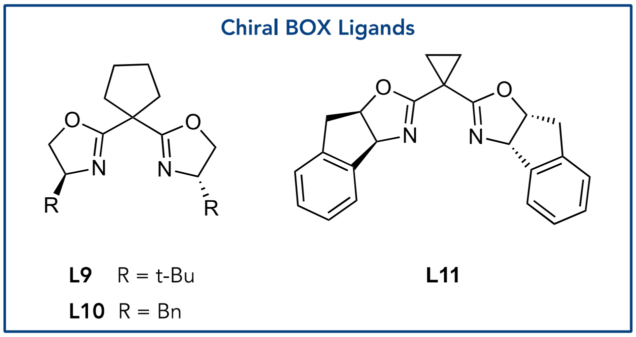

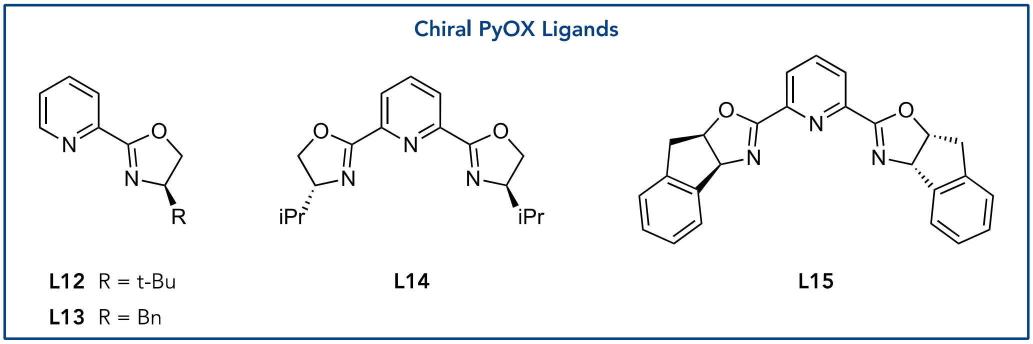

Table S2. Photocatalyst Screen.

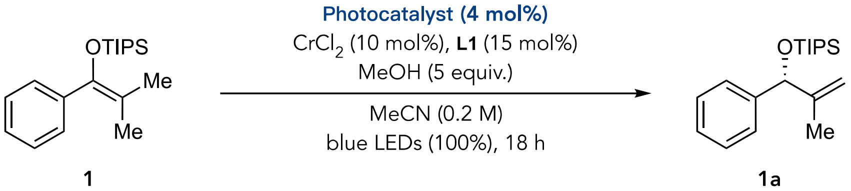

Entry	Photocatalyst	$E^{\circ}_{1/2}$ (Ir^{III*}/Ir^{II}) (V)[a]	RSM (%)	Yield 1a (%)	e.r.
1	$[Ir(dF(CF_3)ppy)_2(5,5'\text{-}d(CF_3)bpy]PF_6$	1.30	<2	20	89:11
2	$[Ir(dF(CF_3)ppy)_2bpy]PF_6$	1.04	<3	15	92:8
3	$[Ir(dF(CF_3)ppy)_2(4,4'\text{-}dtbbpy]PF_6$	0.81	<2	28.5	92:8
4	$[Ir(dF(Me)ppy)_2(4,4'\text{-}dtbbpy]PF_6$	0.59	2	12.5	88:12
5	$[Ir(ppy)_2(4,4'\text{-}dtbbpy]PF_6$	0.26	60	trace	-

[a]Potential reported as V vs Fc^+/Fc

Table S3. Light Intensity Screen.

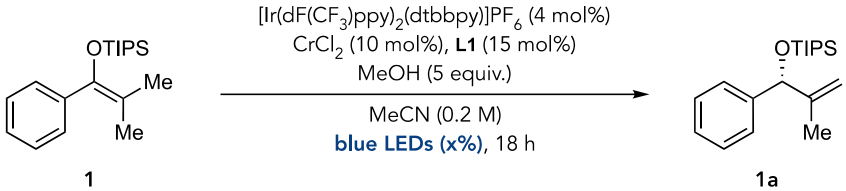

Entry	Light Intensity	Temperature (°C)	RSM (%)	Yield 1a (%)	e.r.
1	100%	35	<2	20	92:8
2	75%	30	<1	29	92:8
3	50%	30	<1	27	92:8
4	25%	29	<2	23	92:8
5[a]	25%	29	29	38	94:6

[a]Reaction was carried out using 2 mol% $[Ir(dF(CF_3)ppy)_2(dtbbpy)]PF_6$, 11 mol% **L1**, and 1 equiv. MeOH for 24h.

Table S4. Additive and Photocatalyst Counteranion Screen.

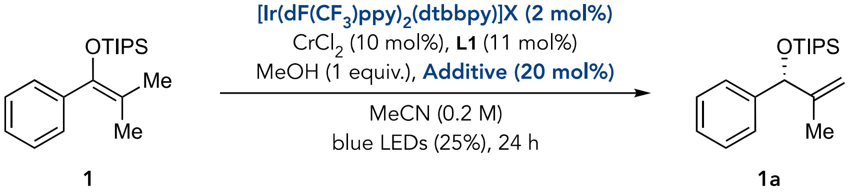

Entry	X	Additive	RSM (%)	Yield 1a (%)	e.r.
1	PF_6	none	29	38	94:6
2	PF_6	$LiPF_6$	trace	trace	-
3	PF_6	LiCl	2	57	94:6
4	PF_6	KCl	2	56	92:8
5	Cl	none	<3	56	94:6
6	Cl	$LiPF_6$	25	26	82:18
7	Cl	LiCl	3	53	94:6
8	Cl	KCl	<3	57	94:6

Table S5. Proton Source, Solvent, and Reagent Loading Optimizations.

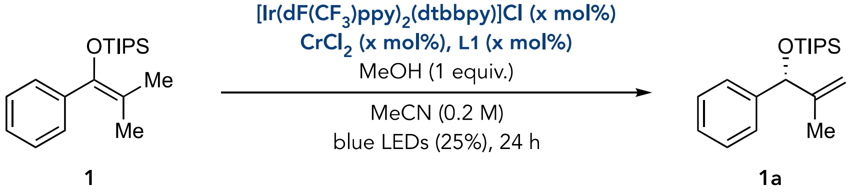

Entry	Photocatalyst (mol%)	Cr / L1 (mol%)	RSM (%)	Yield 1a (%)	e.r.
1	2	10 / 11	<3	56	94:6
2	0.5	10 / 11	20	34	95:5
3	0.25	10 / 11	40	28	96:4
4	2	5 / 6	5	62	95:5
5	2	2 / 4	30	37	95.5:4.5
6	2	1 / 1.3	79	4	92:8
	Deviation from entry 4				
7	EtOH instead of MeOH		10	55	92:8
8	HFIP instead of MeOH		5	17	88:12
9	(R)-phenylethanol instead of MeOH		<1	3	94:6
10	TFT instead of MeCN		35	26	84:16
11	0.1 M MeCN		16	49	94:6
12	0.5 M MeCN		<1	59	94:6

Table S6. Control Experiments.

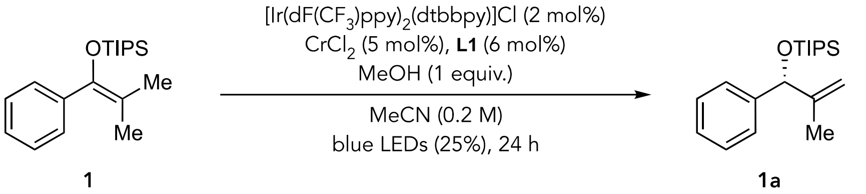

Entry	Deviation	Yield 1a (%)	e.r.
1	None	62	95:5
2	no light	0	-
3	no photocatlyst	0	-
4	no Cr/**L1**	0	-
5	no Cr	0	-
6	no MeOH	45	92:8

Notably, the reaction proceeds in the absence of MeOH albeit with lower yield and enantioselectivity. We hypothesize that adventitious water in the reaction could serve as the proton donor in the protodemetalation step.

5. Synthesis of Products

General Procedure for Preparative Scale Enantioselective Isomerization of Olefins: Inside a N_2-filled glove box, a stock solution (0.2 M to enol ether substrate) was prepared with $CrCl_2$ (5 mol%), **L1** (6 mol%), [Ir(dF(CF_3)ppy)$_2$(dtbbpy)]Cl (2 mol%), and MeOH (1 equiv) in degassed and anhydrous MeCN. An oven-dried 2-dram vial equipped with a magnetic stir bar was charged with enol ether substrate (0.5 mmol, 1.00 equiv) and stock solution (2.5 mL). The vial was sealed with a cap fitted with a PTFE septum. Electrical tape was used to seal the sides of the cap. The vial was removed from the glovebox and placed approximately 2.5 cm away from 34W Kessil PR160-456 nm blue LED lamps (25% intensity) on a stir plate (see Figure S1). The reaction mixture was stirred and irradiated for 24 hours. During irradiation, two rotary fans were placed adjacent to the lamps to cool the reaction setup. The temperature of the setup was measured to be 28±5 °C. The crude reaction mixture was diluted with ethyl acetate and filtered through a pipette silica plug eluting with ethyl acetate. The filtrate was concentrated under reduced pressure. The crude product was purified using silica gel chromatography.

***(R)*-Triisopropyl((2-methyl-1-phenylallyl)oxy)silane (1a)**

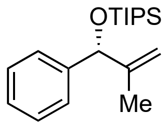

1a

The title compound was obtained as a colorless oil in an average isolated yield of 63% (97.4 mg) and 94:6 er after silica gel chromatography (100% hexanes). **^{1}H NMR (500 MHz, CDCl$_3$)** δ 7.40 – 7.35 (m, 2H), 7.31 – 7.27 (m, 2H), 7.25 – 7.18 (m, 1H), 5.22 – 5.19 (m, 1H), 5.19 – 5.16 (m, 1H), 4.80 – 4.77 (m, 1H), 1.55 (s, 3H), 1.15 – 1.06 (m, 3H), 1.05 – 0.96 (m, 18 H). **^{13}C NMR (126 MHz, CDCl$_3$)** δ 148.38, 143.83, 127.95, 126.96, 126.16, 110.35, 78.88, 18.15, 18.13, 17.14, 12.39. **IR (neat, cm^{-1})** ν 3066, 3027, 2943, 2891, 2866, 1491, 1463, 1450, 1384, 1370, 1189, 1095, 1064, 1028, 1013, 997, 971, 897, 882, 847, 741, 698, 681, 659, 575, 530. **HRMS (EI):** m/z [$C_{19}H_{32}OSi$]$^{\bullet+}$ ([M]$^{\bullet+}$) calculated: 304.22169; found: 304.22213. **HPLC (after desilylation)**: ChiralPak AS-H, 2% *i*-PrOH/hexanes, 1 mL/min, 210 nm, t_r (major) = 9.557 min, t_r (minor) = 10.429 min. $[\alpha]_D^{24}$ = +2.5° (c = 1.000, CHCl$_3$).

***(R)*-Triisopropyl((1-(4-fluorophenyl)-2-methylallyl)oxy)silane (2a)**

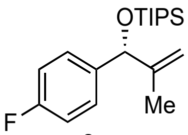

2a

The title compound was obtained as a colorless oil in an average isolated yield of 64% (103 mg) and 94:6 er after silica gel chromatography (100% hexanes). **^{1}H NMR (500 MHz, CDCl$_3$)** δ 7.34 (td, *J* = 8.6, 2.3 Hz, 2H), 6.98 (td, *J* = 8.6, 2.3 Hz, 2H), 5.18 (s, 1H), 5.17 (s, 1H), 4.80 (s, 1H), 1.54 (s, 3H), 1.16 – 0.97 (m, 21H). **^{13}C NMR (126 MHz, CDCl$_3$, mixture of rotamers)** δ 161.98 (d, *J* = 244.2), 148.15, 139.59 (d, *J* = 3.0), 127.65 (d, *J* = 7.9), 114.77 (d, *J* = 21.3), 110.52, 78.28, 18.13, 18.11, 17.04, 12.36. **^{19}F NMR (376 MHz, CDCl$_3$)** δ -116.3 – -116.4 (m). **IR (neat, cm^{-1})** ν 3066, 3027, 2943, 2891, 2866, 1491, 1463, 1450, 1384, 1370, 1189, 1095, 1064, 1028, 1013, 997, 971, 897, 882, 847, 741, 698, 681, 659, 575, 530. **HRMS**

(EI): m/z $[C_{19}H_{31}FOSi]^{\bullet+}$ ($[M]^{\bullet+}$) calculated: 322.21227; found: 322.21179. **HPLC (after desilylation)**: ChiralPak AS-H, 1% *i*-PrOH/hexanes, 1 mL/min, 210 nm, t_r (major) = 15.043 min, t_r (minor) = 16.429 min. $[\alpha]_D^{24}$ = +0.8° (c = 1.000, $CHCl_3$).

***(R)*-Triisopropyl((1-(4-bromophenyl)-2-methylallyl)oxy)silane (3a)**

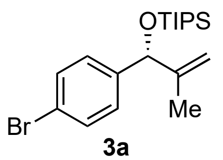

3a

The title compound was obtained as a colorless oil in an average isolated yield of 56% (108.9 mg) and 94:6 er after silica gel chromatography (100% hexanes). **^{1}H NMR (500 MHz, $CDCl_3$)** δ 7.42 (d, *J* = 8.5 Hz, 2H), 7.26 (d, *J* = 8.5 Hz, 2H), 5.16 (s, 2H), 4.80 (s, 1H), 1.53 (s, 3H), 1.16 – 0.98 (m, 21H). **^{13}C NMR (126 MHz, $CDCl_3$)** δ 147.88, 142.96, 131.10, 127.91, 120.78, 110.89, 78.41, 18.13, 16.91, 12.38. **IR (neat, cm^{-1})** *v* 2944, 2891, 2866, 2354, 2340, 1485, 1464, 1106, 1088, 1068, 1011, 900, 882, 844, 794, 682, 663. **HRMS (EI):** m/z $[C_{19}H_{31}BrOSi]^{\bullet+}$ ($[M]^{\bullet+}$) calculated: 382.13221, 384.13016; found: 382.13134, 384.13037. **HPLC (after desilylation)**: ChiralPak AS-H, 2% *i*-PrOH/hexanes, 1 mL/min, 210 nm, t_r (major) = 11.557 min, t_r (minor) = 13.047 min. $[\alpha]_D^{24}$ = –3.4° (c = 1.000, $CHCl_3$).

***(R)*-Triisopropyl((1-([1,1'-biphenyl]-4-yl)-2-methylallyl)oxy)silane (4a)**

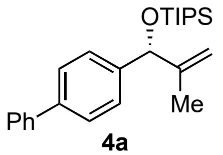

4a

The title compound was obtained as a colorless oil in an average isolated yield of 52% (99.4 mg) and 94.5:5.5 er after silica gel chromatography (100% hexanes). **^{1}H NMR (500 MHz, $CDCl_3$)** δ 7.60 (d, *J* = 7.7 Hz, 2H), 7.54 (d, *J* = 8.0, 2H), 7.47 – 7.39 (m, 4H), 7.36 – 7.29 (m, 1H), 5.26 (s, 1H), 5.21 – 5.19 (m, 1H), 4.83 – 4.80 (m, 1H), 1.60 (s, 3H), 1.19 – 1.08 (m, 3H), 1.09 – 0.99 (m, 18H). **^{13}C NMR (126 MHz, $CDCl_3$)** δ 148.36, 142.95, 141.18, 139.75, 128.83, 127.20, 127.16, 126.71, 126.54, 110.52, 78.71, 18.18, 17.14, 12.42. **IR (neat, cm^{-1})** *v* 2943, 2890, 2866, 1485, 1463, 1090, 1067, 908, 882, 848, 762, 734, 696, 681. **HRMS (EI):** m/z $[C_{25}H_{36}OSi]^{\bullet+}$ ($[M]^{\bullet+}$) calculated: 380.25299; found: 380.25256. **HPLC (after desilylation)**: ChiralPak AS-H, 2% *i*-PrOH/hexanes, 1 mL/min, 210 nm, t_r (major) = 13.322 min, t_r (minor) = 15.070 min. $[\alpha]_D^{24}$ = –3.6° (c = 1.000, $CHCl_3$).

***(R)*-Triisopropyl((1-(4-(tert-butyl)phenyl)-2-methylallyl)oxy)silane (5a)**

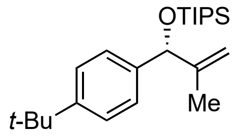

5a

The title compound was obtained as a colorless oil in an average isolated yield of 65% (117.9 mg) and 93:7 er after silica gel chromatography (100% hexanes). **^{1}H NMR** (500 MHz, $CDCl_3$) δ 7.36 – 7.31 (m, 4H), 5.24 (s, 1H), 5.20 (s, 1H), 4.83 (s, 1H), 1.61 (s, 3H), 1.35 (s, 9H), 1.21 – 1.02 (m, 21H). **^{13}C NMR (126 MHz, $CDCl_3$)** δ 149.71, 148.62, 140.69, 125.77, 124.81, 110.22, 78.80, 34.57, 31.60, 18.22, 17.24, 12.51. **IR (neat, cm^{-1})** ν 2944, 2866, 2355, 2340, 1463, 1120, 1090, 1066, 1015, 882, 849, 680, 657, 579. **HRMS (EI):** m/z $[C_{23}H_{40}OSi]^{\bullet+}$ ($[M]^{\bullet+}$) calculated: 360.28429; found: 360.28350. **HPLC (after desilylation)**: ChiralPak AS-H, 2% *i*-PrOH/hexanes, 1 mL/min, 210 nm, t_r (major) = 6.838 min, t_r (minor) = 7.645 min. $[\alpha]_D^{24}$ = –0.6° (c = 1.000, $CHCl_3$).

***(R)*-Triisopropyl((1-(4-methoxyphenyl)-2-methylallyl)oxy)silane (6a)**

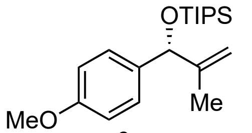

6a

The title compound was obtained as a colorless oil in an average isolated yield of 57% (95.0 mg) and 92.5:7.5 er after silica gel chromatography. **^{1}H NMR (500 MHz, $CDCl_3$)** δ 7.28 (m, 2H), 6.83 (m, 2H), 5.17 – 5.13 (m, 2H), 4.80 – 4.75 (m, 1H), 3.80 (s, 3H), 1.55 (s, 3H), 1.15 – 1.05 (m, 3H), 1.05 – 0.98 (m, 18H). **^{13}C NMR (126 MHz, $CDCl_3$, mixture of rotamers)** δ 158.65, 148.61, 136.12, 127.27, 113.33, 109.94, 78.44, 55.33, 18.17, 18.15, 17.23, 12.43. **IR (neat, cm^{-1})** ν 2942, 2865, 2358, 2340, 1611, 1509, 1463, 1302, 1245, 1180, 1169, 1110, 1086, 1065, 1012, 882, 845, 680, 658, 570. **HRMS (EI):** m/z $[C_{20}H_{34}O_2Si]^{\bullet+}$ ($[M]^{\bullet+}$) calculated: 334.23226; found: 334.23182. **HPLC (after desilylation)**: ChiralPak AS-H, 5% *i*-PrOH/hexanes, 1 mL/min, 210 nm, t_r (major) = 10.951 min, t_r (minor) = 13.988 min. $[\alpha]_D^{24}$ = +6.6° (c = 1.000, $CHCl_3$).

***(R)*-Triisopropyl((2-methyl-1-(m-tolyl)allyl)oxy)silane (7a)**

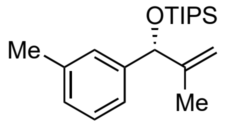

7a

The title compound was obtained as a colorless oil in an average isolated yield of 65% (103.8 mg) and 93:7 er after silica gel chromatography (100% hexanes). **^{1}H NMR (500 MHz, $CDCl_3$)** δ 7.21 – 7.14 (m, 3H), 7.06 – 7.00 (m, 1H), 5.19 – 5.14 (m, 2H), 4.81 – 4.76 (m, 1H), 2.34 (s, 3H), 1.56 (s, 3H), 1.16 – 1.06 (m, 3H), 1.06 – 0.98 (m, 18H). **^{13}C NMR (126 MHz, $CDCl_3$, mixture of rotamers)** δ 148.47, 143.78, 137.40, 127.80, 127.70, 126.87, 123.29, 110.28, 78.90, 21.68, 18.16, 18.15, 17.19, 12.44. **IR (neat, cm^{-1})** ν 2942, 2865, 2357, 2340, 1463, 1100, 1083, 1065, 1013, 997, 896, 882, 839, 762, 700, 680, 659. **HRMS (EI):** m/z $[C_{20}H_{34}OSi]^{\bullet+}$ ($[M]^{\bullet+}$) calculated: 318.23734; found: 318.23734. **HPLC (after desilylation)**: ChiralCel OZ-H, 1% *i*-PrOH/hexanes, 1 mL/min, 210 nm, t_r (major) = 13.386 min, t_r (minor) = 11.829 min. $[\alpha]_D^{24}$ = +1.7° (c = 1.000, $CHCl_3$).

***(R)*-Triisopropyl((2-methyl-1-(o-tolyl)allyl)oxy)silane (8a)**

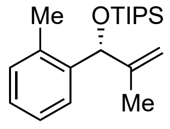

8a

The title compound was obtained as a colorless oil in an average isolated yield of 66% (105.3 mg) and 90:10 er after silica gel chromatography (100% hexanes). **^{1}H NMR (500 MHz, CDCl$_3$)** δ 7.56 (dd, J = 7.6, 1.5 Hz, 1H), 7.19 (td, J = 7.6, 1.5 Hz, 1H), 7.14 (td, J = 7.5, 1.6 Hz, 1H), 7.07 (dd, J = 7.5, 1.6 Hz, 1H), 5.37 (s, 1H), 5.13 (s, 1H), 4.84 (s, 1H), 2.31 (s, 3H), 1.57 (s, 3H), 1.14 – 1.05 (m, 3H), 1.00 (dd, J = 20.7, 7.2 Hz, 18H). **^{13}C NMR (126 MHz, CDCl$_3$, mixture of rotamers)** δ 146.51, 141.34, 134.53, 130.26, 127.20, 126.86, 125.67, 111.17, 75.72, 19.60, 18.14, 18.06, 17.31, 12.41. **IR (neat, cm^{-1})** ν 2943, 2892, 2866, 2355, 2340, 1463, 1113, 1079, 1062, 1013, 997, 898, 883, 850, 837, 748, 723, 681, 659. **HRMS (EI):** m/z [$C_{20}H_{34}OSi$]$^{•+}$ ([M]$^{•+}$) calculated: 318.23734; found: 318.23680. **HPLC (after desilylation)**: ChiralCel OZ-H, 1% *i*-PrOH/hexanes, 1 mL/min, 210 nm, t_r (major) = 12.848 min, t_r (minor) = 14.966 min. $[\alpha]_D^{24}$ = –3.9° (c = 1.000, CHCl$_3$).

***(R)*-Triisopropyl((1-(3,5-dimethylphenyl)-2-methylallyl)oxy)silane (9a)**

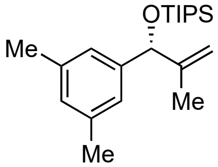

9a

The title compound was obtained as a colorless oil in an average isolated yield of 66% (109.2 mg) and 90:10 er after silica gel chromatography (100% hexanes). **^{1}H NMR (500 MHz, CDCl$_3$)** δ 6.98 (s, 2H), 6.85 (s, 1H), 5.17 – 5.14 (m, 1H), 5.13 (s, 1H), 4.80 – 4.76 (m, 1H), 2.29 (s, 6H), 1.56 (s, 3H), 1.14 – 1.06 (m, 3H), 1.06 – 0.98 (m, 18H). **^{13}C NMR (126 MHz, CDCl$_3$, mixture of rotamers)** δ 148.56, 143.74, 137.23, 128.55, 124.01, 110.22, 78.89, 21.55, 18.17, 18.16, 17.24, 12.45. **IR (neat, cm^{-1})** ν 2942, 2892, 2866, 1608, 1463, 1387, 1370, 1148, 1094, 1067, 1013, 997, 896, 882, 849, 700, 681. **HRMS (EI):** m/z [$C_{21}H_{36}OSi$]$^{•+}$ ([M]$^{•+}$) calculated 332.25299; found: 332.25307. **HPLC (after desilylation)**: ChiralCel OZ-H, 1% *i*-PrOH/hexanes, 1 mL/min, 210 nm, t_r (major) = 10.389 min, t_r (minor) = 11.552 min. $[\alpha]_D^{24}$ = +2.1° (c = 1.000, CHCl$_3$).

***(R)*-Triisopropyl((1-(3,4-dimethoxyphenyl)-2-methylallyl)oxy)silane (10a)**

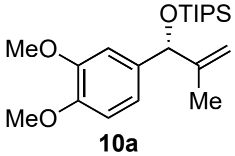

10a

The title compound was obtained as a colorless oil in an average isolated yield of 61% (93.3 mg) and 92:8 er after silica gel chromatography. **^{1}H NMR (500 MHz, CDCl$_3$)** δ 6.97 (d, J = 1.8 Hz, 1H), 6.87 (dd, J = 8.2, 1.8 Hz, 1H), 6.79 (d, J = 8.2 Hz, 1H), 5.16 – 5.14 (m, 2H), 4.80 – 4.77 (m, 1H), 3.87 (s, 6H), 1.55 (s,

3H), 1.15 – 1.06 (m, 3H), 1.06 – 0.98 (m, 18H). **^{13}C NMR (126 MHz, CDCl$_3$, mixture of rotamers)** *δ* 148.62, 148.48, 147.88, 136.56, 118.32, 110.43, 110.18, 109.25, 78.49, 55.94, 55.86, 18.17, 18.15, 17.23, 12.41. **IR (neat, cm^{-1})** *ν* 2942, 2865, 2358, 1593, 1514, 1464, 1417, 1388, 1368, 1256, 1232, 1137, 1150, 1089, 1065, 1030, 881, 854, 758, 679, 668, 656. **HRMS (EI):** m/z [$C_{21}H_{36}O_3Si$]$^{•+}$ ([M]$^{•+}$) calculated: 364.24282; found: 364.24236. **HPLC (after desilylation)**: ChiralCel OZ-H, 15% *i*-PrOH/hexanes, 1 mL/min, 210 nm, t_r (major) = 11.083 min, t_r (minor) = 14.629 min. $[\alpha]_D^{24}$ = +5.1° (c = 1.000, CHCl$_3$).

(*R*)-Triisopropyl((1-(4-fluoro-2,5-dimethoxyphenyl)-2-methylallyl)oxy)silane (11a)

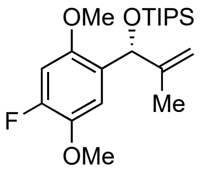

11a

The title compound was obtained as a colorless oil in an average isolated yield of 70% (133.3 mg) and 78:22 er after silica gel chromatography. **^{1}H NMR (500 MHz, CDCl$_3$)** *δ* 7.19 (d, *J* = 10.3 Hz, 1H), 6.63 (d, *J* = 12.3 Hz, 1H), 5.58 (s, 1H), 5.19 – 5.15 (m, 1H), 4.79 – 4.74 (m, 1H), 3.84 (s, 3H), 3.75 (s, 3H), 1.58 (s, 3H), 1.15 – 1.04 (m, 3H), 1.00 (dd, *J* = 18.6, 7.3 Hz, 18H). **^{13}C NMR (126 MHz, CDCl$_3$, mixture of rotamers)** *δ* 151.66 (d, *J* = 244.4 Hz), 149.86 (d, *J* = 8.5 Hz), 147.18, 141.26 (d, *J* = 10.8 Hz), 128.05 (d, *J* = 3.3 Hz), 113.39 (d, *J* = 3.3 Hz), 110.13, 100.29 (d, *J* = 22.2 Hz), 70.92, 57.06, 56.16, 18.10, 18.03, 17.34, 12.32. **^{19}F NMR (470 MHz, CDCl$_3$)** *δ* -135.10 (dd, *J* = 12.8, 10.0 Hz). **IR (neat, cm^{-1})** *ν* 2942, 2865, 2358, 2341, 1503, 1464, 1363, 1208, 1184, 1115, 1083, 1063, 1040, 996, 881, 865, 826, 768, 680, 619. **HRMS (EI):** m/z [$C_{21}H_{35}FO_3Si$]$^{•+}$ ([M]$^{•+}$) 382.23340; found: 382.23401. **HPLC**: ChiralCel OZ-H, 100% Hexanes, 1 mL/min, 210 nm, t_r (major) = 13.971 min, t_r (minor) = 17.294 min. $[\alpha]_D^{24}$ = +16.6° (c = 1.000, CHCl$_3$).

***(R)*- Triisopropyl((1-(benzo[*d*][1,3]dioxol-5-yl)-2-methylallyl)oxy)silane (12a)**

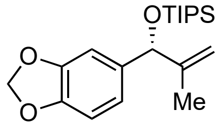

12a

The title compound was obtained as a colorless oil in an average isolated yield of 58% (101.9 mg) and 92:8 er after silica gel chromatography. **^{1}H NMR (500 MHz, CDCl$_3$)** *δ* 6.89 (d, *J* = 1.6 Hz, 1H), 6.81 (dd, *J* = 8.0, 1.6 Hz, 1H), 6.73 (d, *J* = 8.0 Hz, 1H), 5.95 – 5.92 (m, 2H), 5.16 – 5.13 (m, 1H), 5.11 (s, 1H), 4.80 – 4.76 (m, 1H), 1.55 (s, 3H), 1.15 – 1.06 (m, 3H), 1.06 – 0.99 (m, 18H). **^{13}C NMR (126 MHz, CDCl$_3$, mixture of rotamers)** *δ* 148.39, 147.45, 146.50, 138.13, 119.41, 110.23, 107.65, 106.78, 100.95, 78.58, 18.16, 18.15, 17.23, 12.41. **IR (neat, cm^{-1})** *ν* 2942, 2891, 2865, 2354, 2341, 1503, 1485, 1440, 1243, 1094, 1065, 1041, 932, 898, 882, 853, 681, 668, 660. **HRMS (EI):** m/z [$C_{20}H_{32}O_3Si$]$^{•+}$ ([M]$^{•+}$) calculated: 348.21152; found m/z 348.21109. **HPLC (after desilylation)**: ChiralPak AS-H, 5% *i*-PrOH/hexanes, 1 mL/min, 210 nm, t_r (major) = 16.238 min, t_r (minor) = 17.766 min. $[\alpha]_D^{24}$ = +4.7° (c = 1.000, CHCl$_3$).

***(R)*-Triisopropyl((1-(benzofuran-5-yl)-2-methylallyl)oxy)silane (13a)**

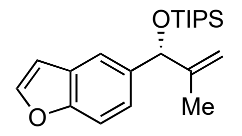

13a

The title compound was obtained as a colorless oil in an average isolated yield of 72% (124.4 mg) and 92:8 er after silica gel chromatography. **^{1}H NMR (500 MHz, $CDCl_3$)** δ 7.61 (d, J = 1.7 Hz, 1H), 7.60 (d, J = 2.2 Hz, 1H), 7.42 (dt, J = 8.5, 0.8 Hz, 1H), 7.32 (dd, J = 8.5, 1.7 Hz, 1H), 6.74 (dd, J = 2.2, 0.8 Hz, 1H), 5.31 (s, 1H), 5.24 – 5.20 (m, 1H), 4.83 – 4.78 (m, 1H), 1.56 (s, 3H), 1.18 – 1.08 (m, 3H), 1.07 – 0.99 (m, 18H). **^{13}C NMR (126 MHz, $CDCl_3$, mixture of rotamers)** δ 154.37, 148.72, 145.18, 138.59, 127.07, 122.83, 118.68, 110.75, 110.11, 106.88, 78.95, 18.18, 18.17, 17.26, 12.45. **IR (neat, cm^{-1})** ν 2943, 2891, 2866, 2358, 2341, 1465, 1263, 1138, 1124, 1106, 1089, 1066, 884, 843, 765, 735, 682, 661. **HRMS (EI):** m/z $[C_{21}H_{32}O_2Si]^{\bullet+}$ ($[M]^{\bullet+}$) calculated: 344.21661; found: 344.21728. **HPLC (after desilylation)**: ChiralPak AS-H, 10% *i*-PrOH/hexanes, 1 mL/min, 210 nm, t_r (major) = 7.365 min, t_r (minor) = 8.994 min. $[\alpha]_D^{24}$ = +9.5° (c = 1.000, $CHCl_3$).

***(R)*-1-Methyl-5-(2-methyl-1-((triisopropylsilyl)oxy)allyl)-1*H*-indole (14a)**

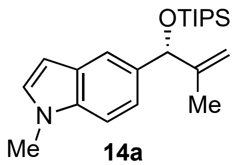

14a

The title compound was obtained as a colorless oil in an average isolated yield of 38% (67.2 mg) and 92:8 er after silica gel chromatography. **^{1}H NMR (500 MHz, $CDCl_3$)** δ 7.64 – 7.60 (m, 1H), 7.29 – 7.22 (m, 2H), 7.03 (d, J = 3.1 Hz, 1H), 6.46 (d, J = 3.1 Hz, 1H), 5.32 (s, 1H), 5.25 – 5.20 (m, 1H), 4.80 – 4.75 (m, 1H), 3.78 (s, 3H), 1.57 (s, 3H), 1.18 – 1.00 (m, 21H). **^{13}C NMR (126 MHz, $CDCl_3$, mixture of rotamers)** δ 149.22, 136.18, 134.93, 128.89, 128.09, 120.38, 118.49, 109.46, 108.66, 101.08, 79.30, 33.00, 18.23, 18.21, 17.49, 12.49. **IR (neat, cm^{-1})** ν 2942, 2865, 2357, 2340, 1464, 1447, 1086, 1066, 883, 843, 716, 680, 669, 658. **HRMS (EI):** m/z $[C_{22}H_{35}NOSi]^{\bullet+}$ ($[M]^{\bullet+}$) calculated: 357.24824; found: 357.24902. **HPLC (after desilylation)**: ChiralPak AS-H, 10% *i*-PrOH/hexanes, 1 mL/min, 210 nm, t_r (major) = 15.065 min, t_r (minor) = 24.833 min. $[\alpha]_D^{24}$ = +14.0° (c = 1.000, $CHCl_3$).

***tert*-Butyl *(R)*-6-(2-methyl-1-((triisopropylsilyl)oxy)allyl)-1*H*-indole-1-carboxylate (15a)**

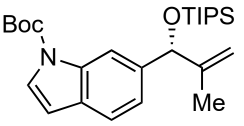

15a

The title compound was obtained as a colorless oil in an average isolated yield of 50% (109.8 mg) and 95:5 er. after purification by silica gel chromatography. **^{1}H NMR (500 MHz, $CDCl_3$)** δ 8.12 (s, 1H), 7.57 (d, J

= 3.7 Hz, 1H), 7.47 (d, *J* = 8.1 Hz, 1H), 7.31 (d, *J* = 8.1 Hz, 1H), 6.53 (d, *J* = 3.7 Hz, 1H), 5.31 (s, 1H), 5.28 – 5.22 (m, 1H), 4.82 – 4.77 (m, 1H), 1.67 (s, 9H), 1.59 (s, 3H), 1.17 – 1.07 (m, 3H), 1.02 (m, 18H). **^{13}C NMR (126 MHz, CDCl$_3$, mixture of rotamers)** *δ* 150.00, 148.77, 140.48, 135.13, 129.82, 126.04, 121.26, 120.30, 113.24, 109.96, 107.28, 83.62, 79.38, 28.37, 18.19, 18.17, 17.48, 12.44. **IR (neat, cm^{-1})** *ν* 2942, 2865, 2355, 2341, 1731, 1435, 1369, 1332, 1248, 1168, 1142, 1123, 1091, 1066, 882, 841, 766, 681, 644. **HRMS (EI):** m/z [$C_{26}H_{41}NO_3Si$]$^{•+}$ ([M]$^{•+}$) calculated: 443.28502; found: 443.28677. **HPLC (after desilylation)**: ChiralPak AS-H, 5% *i*-PrOH/hexanes, 1 mL/min, 210 nm, t$_r$ (major) = 8.303 min, t$_r$ (minor) = 10.470 min. $[\alpha]_D^{24}$ = +25.0° (c = 1.000, CHCl$_3$).

(*S*)-Triisopropyl((2-methyl-5-phenylpent-1-en-3-yl)oxy)silane (16a)

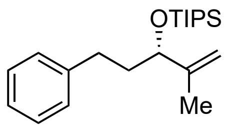

16a

The title compound was obtained as a colorless oil in an average isolated yield of 64% (106.0 mg) and 76.5:23.5 er after purification by silica gel chromatography. **^{1}H NMR (500 MHz, CDCl$_3$)** *δ* 7.30 – 7.25 (m, 2H), 7.21 – 7.15 (m, 3H), 4.94 (m, 1H), 4.87 (m, 1H), 4.24 (t, *J* = 6.1 Hz, 1H), 2.64 – 2.45 (m, 2H), 1.94 – 1.84 (m, 2H), 1.74 (s, 3H), 1.12 – 1.03 (m, 21H). **^{13}C NMR (126 MHz, CDCl$_3$, mixture of rotamers)** *δ* 146.89, 142.72, 128.53, 128.44, 125.77, 111.68, 76.45, 37.79, 31.33, 18.26, 18.24, 17.25, 12.56. **IR (neat, cm^{-1})** *ν* 3066, 3027, 2943, 2891, 2866, 1652, 1605, 1496, 1463, 1383, 1371, 1248, 1086, 1064, 998, 899, 883, 748, 698, 680. **HRMS (EI):** m/z [$C_{21}H_{36}OSi$]$^{•+}$ ([M]$^{•+}$) calculated 332.25299; found: 332.25421. **HPLC (after desilylation)**: ChiralPak AS-H, 2% *i*-PrOH/hexanes, 1 mL/min, 210 nm, t$_r$ (major) = 8.593 min, t$_r$ (minor) = 9.510 min. $[\alpha]_D^{24}$ = +8.0° (c = 0.94, CHCl$_3$).

***(R)-tert*-Butyl((2-methyl-1-phenylallyl)oxy)diphenylsilane (17a)**

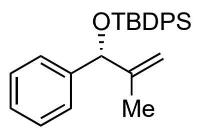

17a

The title compound was obtained as a colorless oil in an average isolated yield of 70% (135.1 mg) and 93:7 er after purification by silica gel chromatography. **^{1}H NMR (500 MHz, CDCl$_3$)** *δ* 7.68 (d, *J* = 6.9 Hz, 2H), 7.51 – 7.46 (m, 2H), 7.46 – 7.37 (m, 1H), 7.36 – 7.30 (m, 3H), 7.27 – 7.16 (m, 7H), 5.08 (s, 1H), 5.07 – 5.03 (m, 1H), 4.76 – 4.72 (m, 1H), 1.53 (s, 3H), 1.09 (s, 9H). **^{13}C NMR (126 MHz, CDCl$_3$)** *δ* 147.24, 143.04, 136.04, 135.99, 134.02, 133.81, 129.70, 129.56, 127.97, 127.56, 127.45, 127.04, 126.50, 110.83, 79.50, 27.12, 19.63, 17.69. **IR (neat, cm^{-1})** *ν* 2922, 2854, 2354, 1457, 1427, 1111, 1085, 1063, 1028, 844, 821, 739, 698, 609, 546, 502, 486. **HRMS (EI):** m/z [$C_{26}H_{30}OSi$]$^{•+}$ ([M]$^{•+}$) calculated: 386.20604; found: 386.20526. **HPLC (after desilylation)**: ChiralPak AS-H, 2% *i*-PrOH/hexanes, 1 mL/min, 210 nm, t$_r$ (major) = 10.089 min, t$_r$ (minor) = 10.987 min. $[\alpha]_D^{24}$ = +59.7° (c = 1.000, CHCl$_3$)

***(R)-tert*-Butyldimethyl((2-methyl-1-phenylallyl)oxy)silane (18a)**

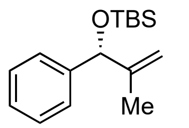

18a

The title compound was obtained as a colorless oil in an average isolated yield of 70% (92.3 mg) and 92:8 er after purification by silica gel chromatography. **^{1}H NMR (500 MHz, CDCl$_3$)** δ 7.38 – 7.33 (m, 2H), 7.33 – 7.28 (m, 2H), 7.23 (m, 1H), 5.14 – 5.09 (m, 2H), 4.84 (m, 1H), 1.54 (s, 3H), 0.93 (m, 9H), 0.07 (s, 3H), -0.01 (s, 3H). **^{13}C NMR (126 MHz, CDCl$_3$, mixture of rotamers)** δ 148.09, 143.41, 128.04, 127.00, 126.19, 111.02, 78.69, 25.98, 25.95, 18.47, 17.30, -4.78, -4.89. **IR (neat, cm^{-1})** ν 2955, 2929, 2856, 2354, 2340, 1472, 1450, 1250, 1090, 1065, 1029, 1005, 898, 864, 835, 775, 739, 698, 672. **HRMS (EI):** m/z [$C_{16}H_{26}OSi$]$^{\bullet+}$ ([M]$^{\bullet+}$) calculated: 262.17474; found: 262.17385. **HPLC (after desilylation)**: ChiralPak AS-H, 2% *i*-PrOH/hexanes, 1 mL/min, 210 nm, t_r (major) = 10.071 min, t_r (minor) = 10.994 min. $[\alpha]_D^{24}$ = –6.2° (c = 1.000, CHCl$_3$)

(*R*)-(2-Methyl-1-(neopentyloxy)allyl)benzene (19a)

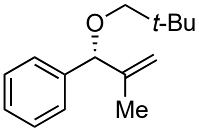

19a

The title compound was obtained as a colorless oil in an average isolated yield of 71% (77.0 mg) and 75:25 er after purification by silica gel chromatography. **^{1}H NMR (500 MHz, CDCl$_3$)** δ 7.40 – 7.30 (m, 4H), 7.28 – 7.23 (m, 1H), 5.10 (m, 1H), 4.95 (m, 1H), 4.66 (s, 1H), 3.11 (dd, *J* = 8.5, 1.0 Hz, 1H), 3.00 (dd, *J* = 8.5, 1.0 Hz, 1H), 1.56 (s, 3H), 0.97 (s, 9H). **^{13}C NMR (126 MHz, CDCl$_3$)** δ 145.94, 141.44, 128.12, 127.17, 126.60, 112.86, 85.52, 79.04, 32.26, 27.05, 17.48. **IR (neat, cm^{-1})** ν 3066, 3027, 2897, 2845, 1650, 1602, 1492, 1448, 1371, 1344, 1289, 1257, 1227, 1189, 1156, 1089, 1073, 1028, 898, 740, 697. **HRMS (EI):** m/z [$C_{15}H_{22}O$]$^{\bullet+}$ ([M]$^{\bullet+}$) calculated: 218.16652; found: 218.16725. **HPLC**: ChiralCel OJ-H, 100% Hexanes, 0.5 mL/min, 210 nm, t_r (major) = 7.578 min, t_r (minor) = 8.016 min. $[\alpha]_D^{23}$ = –17.3° (c = 1.000, CHCl$_3$).

1-((((*R*)-2-Methyl-1-phenylallyl)oxy)methyl)adamantane (20a)

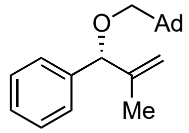

20a

The title compound was obtained as a colorless oil in an average isolated yield of 79% (94.4 mg) and 70:30 er. after purification by silica gel chromatography. **^{1}H NMR (500 MHz, CDCl$_3$)** δ 7.43 – 7.32 (m, 4H), 7.30 – 7.25 (m, 1H), 5.12 (m, 1H), 4.98 (m, 1H), 4.65 (s, 1H), 3.05 (d, *J* = 8.7 Hz, 1H), 2.95 (d, *J* = 8.7 Hz, 1H), 2.01 (p, *J* = 3.2 Hz, 3H), 1.80 – 1.69 (m, 6H), 1.65 (d, *J* = 3.1 Hz, 6H), 1.58 (s, 3H). **^{13}C NMR (126 MHz, CDCl$_3$)** δ 145.96, 141.46, 128.09, 127.13, 126.59, 112.88, 85.40, 79.44, 40.05, 37.46, 34.24, 28.51,

17.47. **IR (neat, cm^{-1})** ν 3066, 3028, 2972, 2954, 2904, 2866, 2845. 1653, 1636, 1493, 1478, 1450, 1403, 1372. 1362, 1338,1288, 1221, 1191, 1096, 901, 737, 698. **HRMS (EI):** m/z $[C_{21}H_{28}O]^{\bullet+}$ ($[M]^{\bullet+}$) calculated: 296.21347; found: 296.21398. **SFC**: ColumnTek EnantioCel® A6-5, 10% MeOH (0.1% diethylamine) 90% CO2, 2.0 mL/min, 220 nm, t_r (major) = 3.334 min, t_r (minor) = 3.05 min. $[\alpha]_D^{23}$ = –12.5 (c = 1.000, $CHCl_3$).

6. Substrates with Low Reactivity

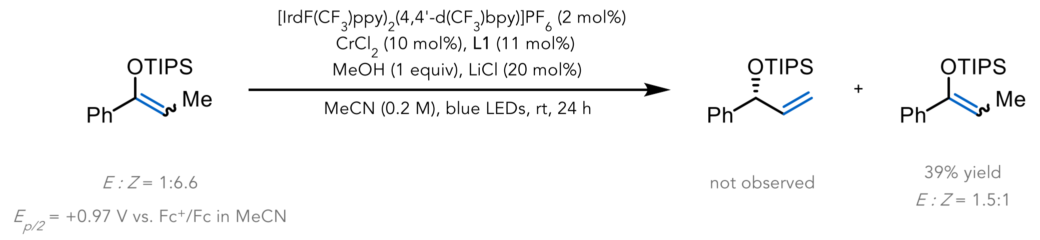

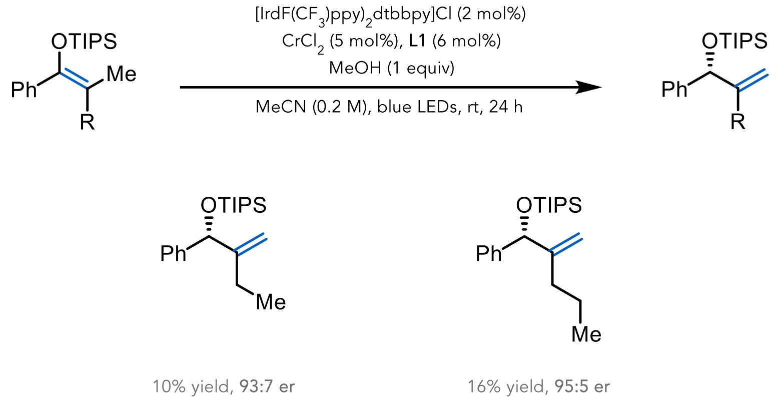

7. Determination of Product Absolute Configuration

- **Synthesis of Enantiopure Product & (*R*)-ferrocenyl 2-methyl-3-phenylprop-2-enoate**

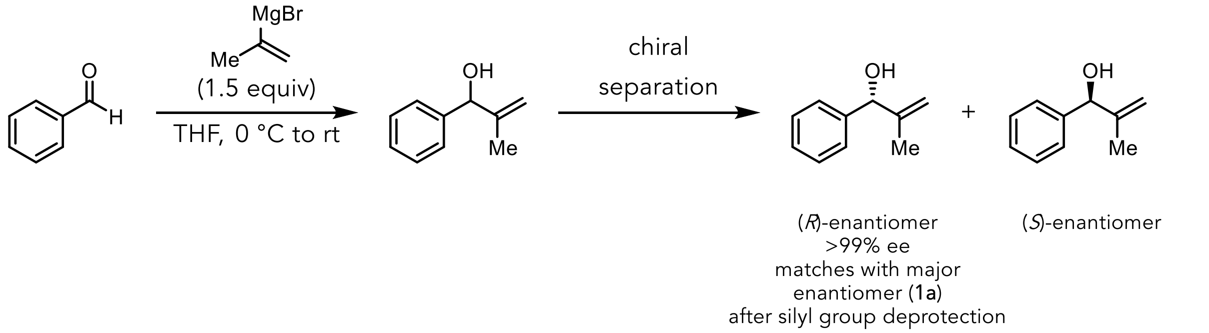

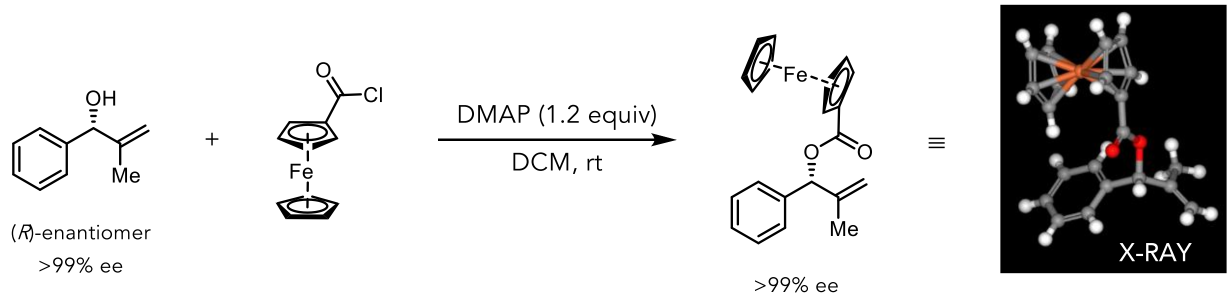

Benzaldehyde (1 equiv) was added dropwise to a THF (0.2 M) solution of isopropenylmagenesium bromide (1.2 equiv) at 0 °C. The resulting solution was stirred at 0 °C for 30 min, slowly warmed to room temperature, and stirred for 3 hours. The reaction was quenched with saturated ammonium chloride and extracted with diethyl ether (3 x 50 mL). The combined organic layers were washed with water and brine, dried over anhydrous Na_2SO_4, filtered, and concentrated using a rotary evaporator. The crude material was purified by column chromatography using ethyl acetate and hexanes to give the corresponding racemic alcohol products. Chiral supercritical fluid chromatography was performed by Lotus Separations, LLC to separate two enantiomers.

The enantiomer 1 (200 mg, 1.35 mmol, 1 equiv), which corresponds to the major enantiomer of **1a** after deprotection of the silyl group was dissolved in 4 mL of DCM and DMAP (1.2 equiv) was added. A solution of ferrocenoyl chloride (1.5 equiv) in 3 mL of DCM was added dropwise at room temperature and stirred overnight. The reaction mixture was quenched by adding silica gel, and the volatiles were removed using a rotary evaporator. The crude material was purified by silica gel chromatography to obtain 302 mg (64% yield) of orange solid. A benzene solution of the product was diluted with pentanes by vapor diffusion and cooled at -10 °C. A crystal suitable for X-ray diffraction was obtained as a pale orange, translucent block. The absolute configuration was determined based on the X-ray crystallography of **(*R*)-ferrocenyl 2-methyl-3-phenylprop-2-enoate**.

- **Crystallography Data**

Table S7. Sample and crystal data for (*R*)-ferrocenyl 2-methyl-3-phenylprop-2-enoate.

Chemical formula	$C_{21}H_{20}FeO_2$	
Formula weight	360.22 g/mol	
Temperature	100(2) K	
Wavelength	1.54178 Å	
Crystal size	0.382 mm x 0.025 mm x 0.018 mm	
Crystal system	Orthorhombic	
Space group	P 21 21 21	
Unit cell dimensions	a = 5.8817(2) Å	α = 90°
	b = 11.1589(4) Å	β = 90°
	c = 25.8643(9) Å	γ = 90°
Volume	1697.56(10) $Å^3$	
Z	4	
Density (calculated)	1.410 g/cm^3	
Absorption coefficient	7.189 mm^{-1}	
F(000)	752	

Table S8. Data collection and structure refinement for (*R*)-ferrocenyl 2-methyl-3-phenylprop-2-enoate.

Diffractometer	Bruker D8 Venture Photon 3
Radiation source	micro-focus sealed X-ray tube (CuKα, λ = 1.54178 Å)
Theta range for data collection	3.42 to 70.05 º
Index ranges	-7<=h<=6, -13<=k<=13, -31<=l<=31
Reflections collected	23183
Independent reflections	3221
Coverage of independent reflections	100.0%
Absorption correction	Multi-scan
Structure solution technique	Direct methods
Structure solution program	SHELXT (Sheldrick, 2016)
Refinement method	Full-matrix least-squares on F2
Refinement program	SHELX-2018/3 (Sheldrick, 2018)
Function minimized	$\Sigma w(Fo^2 - Fc^2)^2$
Data / restraints / parameters	3221 / 0 / 199
Goodness-of-fit on F^2	1.030
Δ/σ(max)	0.000
Final R indices	0.0332
.........all data	wR2 = 0.0792
Weighting scheme	w=1/[\s^2^(Fo^2^)+(0.0341P)^2^+0.4241P] where P=(Fo^2^+2Fc^2^)/3
Largest diff. peak and hole	0.331 and -0.337 eÅ-3

Table S9. Atomic coordinates and equivalent isotropic atomic parameters (Å²) for (*R*)-ferrocenyl 2-methyl-3-phenylprop-2-enoate. U(eq) is defined as one third of the trace of the orthogonalized Uij tensor.

Atom	x/a	y/b	z/c	U(eq)
Fe1	0.73849(9)	0.72778(4)	0.69998(2)	0.01797(15)
C2	0.6228(6)	0.8584(3)	0.65111(14)	0.0240(8)
H2	0.46016	0.881352	0.645762	0.029
C3	0.7725(7)	0.9087(3)	0.68823(13)	0.0246(8)
H3	0.733925	0.973132	0.713622	0.03
C4	0.9868(7)	0.8505(3)	0.68323(15)	0.0246(8)
H4	1.125337	0.86701	0.704497	0.029
C5	0.9686(6)	0.7636(3)	0.64301(14)	0.0224(7)
H5	1.092311	0.708788	0.630955	0.027
C6	0.7437(7)	0.7687(3)	0.62300(12)	0.0221(6)
H6	0.68117	0.71813	0.594426	0.026
C7	0.8177(6)	0.5615(3)	0.72678(14)	0.0216(8)
H7	0.945489	0.509644	0.714516	0.026
C8	0.8262(7)	0.6447(3)	0.76820(14)	0.0252(8)
H8	0.962989	0.663322	0.789714	0.03
C9	0.6083(7)	0.6994(3)	0.77307(14)	0.0248(8)
H9	0.566662	0.763282	0.798492	0.03
C10	0.4627(7)	0.6512(3)	0.73445(14)	0.0233(8)
H10	0.299809	0.672952	0.728506	0.028
C11	0.5919(6)	0.5640(3)	0.70622(14)	0.0194(7)
C12	0.5062(6)	0.5008(3)	0.66033(14)	0.0206(7)
O13	0.3193(4)	0.5145(2)	0.64175(11)	0.0300(6)
O14	0.6611(4)	0.4219(2)	0.64206(9)	0.0205(5)
C15	0.6076(6)	0.3671(3)	0.59182(14)	0.0200(7)
H15	0.439515	0.35469	0.589576	0.024
C16	0.7230(6)	0.2463(3)	0.59130(12)	0.0210(7)

C17	0.9764(6)	0.2437(3)	0.59785(15)	0.0276(8)
H17A	1.025762	0.161385	0.604894	0.041
H17B	1.019808	0.295456	0.626819	0.041
H17C	1.049285	0.272446	0.566115	0.041
C18	0.6004(7)	0.1485(3)	0.58397(16)	0.0300(9)
H18A	0.672408	0.072381	0.582666	0.036
H18B	0.440179	0.154371	0.580008	0.036
C19	0.6791(6)	0.4534(3)	0.54958(14)	0.0201(7)
C20	0.5307(6)	0.4757(3)	0.50878(15)	0.0252(8)
H20	0.389039	0.434975	0.507285	0.03
C21	0.5877(7)	0.5567(4)	0.47036(16)	0.0299(9)
H21	0.485133	0.57176	0.442729	0.036
C22	0.7951(7)	0.6160(3)	0.47233(14)	0.0271(9)
H22	0.834627	0.671456	0.44596	0.033
C23	0.9437(7)	0.5944(3)	0.51240(15)	0.0262(8)
H23	1.084937	0.635631	0.513717	0.031
C24	0.8878(7)	0.5125(3)	0.55108(15)	0.0233(8)
H24	0.991771	0.496918	0.578381	0.028

8. GPC Analysis

The reaction with enol ether **1** (0.2 mmol) was conducted under standard reaction conditions. The crude reaction mixture was filtered through a pipette silica plug eluting with ethyl acetate. The resulting mixture was concentrated under reduced pressure to remove volatiles and redissolved in THF. The reaction mixture was filtered through a syringe filter and subjected to room temperature GPC analysis.

The GPC analysis of the sample (blue line) showed new peaks corresponding to high molecular weight species (peaks at M_N 6,595 and M_N 3,683) that did not appear in the sample prepared from the control reaction conducted without blue LEDs (orange line).

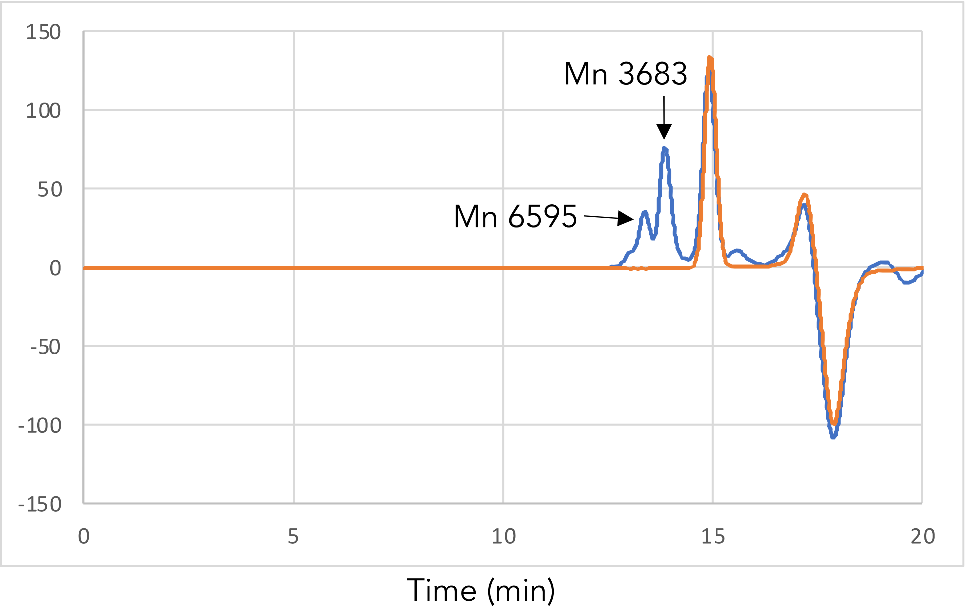

9. Mechanistic Studies

- **Kinetic Experiments**

General Procedure for Kinetic and KIE Experiments: inside a N_2-filled glove box, a stock solution (0.2 M to enol ether substrate) was prepared with $CrCl_2$ (5 mol%), **L1** (6 mol%), [Ir(dF(CF_3)ppy)$_2$(dtbbpy)]Cl (2 mol%), and either **CH_3OH** or **CD_3OD** (1 equiv) in degassed and anhydrous MeCN. An oven-dried 2-dram vial equipped with a magnetic stir bar was charged with enol ether **2** or **2-*d*$_6$** (161.3 mg, 0.5 mmol, 1.00 equiv), stock solution (2.5 mL), and trifluorotoluene (61 µL, 0.5 mmol, 1.00 equiv) as an internal standard. The vial was sealed with a cap fitted with a PTFE septum. Electrical tape was used to seal the sides of the cap. The vial was removed from the glovebox. With an N_2 inlet, the vial was placed approximately 2.5 cm away from 34W Kessil PR160-456 nm blue LED lamps (25% intensity) on a stir plate (see Figure S2). An aliquot (~20 µL) for time zero was taken with a degassed disposable syringe, diluted with MeCN-d_3 (0.1 mL) and MeCN (0.4 mL), and analyzed with quantitative ^{19}F NMR. The reaction mixture was irradiated. During irradiation, two rotary fans were placed adjacent to the lamps to cool the reaction setup. The temperature of the setup was measured to be 28±5 °C. Aliquots (~20 µL) were taken at various time points and analyzed similarly. For initial-rate measurements, the reactions were monitored until the product yield reached ~15%. Deuterium incorporation was determined by ^{1}H NMR analysis by comparing the integrations of the stereogenic proton to that of allylic or aromatic protons. NMR samples were prepared by passing reaction aliquots (~40 µL) through a short silica plug, followed by concentration and dilution with either $CDCl_3$ or CD_3CN. When necessary, the product was further purified by preparative TLC (100% hexanes) prior to analysis.

Note = Induction periods varied up to 1 h. To minimize errors, all KIE experiments were conducted using identical light setups. While absolute KIE values may be prone to error, the conclusion that both the KIEs for methanol and the substrate are primary is definitive and unaffected by the errors.

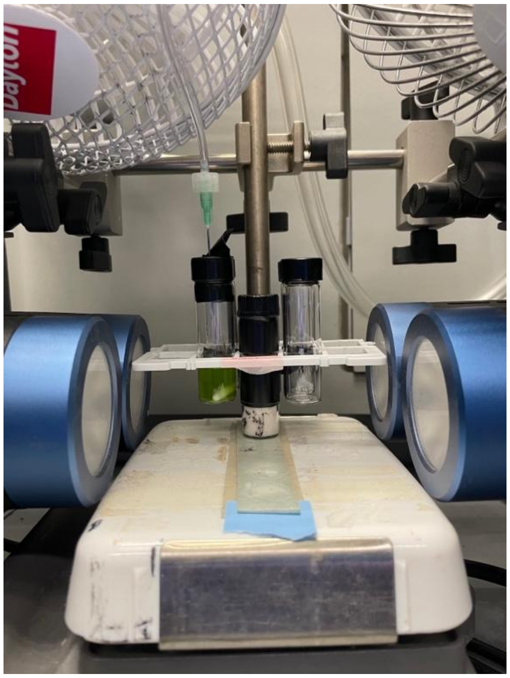

Figure S2. Typical reaction setup for kinetic experiments.

Table S10. Kinetics Data for Figure 3A.

Time (h)	[2] (M)	ln([2])	[2a] (M)	[2]+[2a] (M)
0	0.200	-1.609	0.000	0.200
1	0.198	-1.619	0.000	0.198
1.5	0.194	-1.638	0.000	0.194
2	0.192	-1.652	0.000	0.192
2.25	0.187	-1.674	0.000	0.187
**2.5**	**0.182**	**-1.704**	**0.001**	**0.183**
2.75	0.176	-1.737	0.002	0.178
3	0.171	-1.766	0.005	0.176
3.25	0.162	-1.817	0.009	0.171
3.5	0.158	-1.845	0.013	0.171
3.75	0.153	-1.877	0.017	0.170
4	0.145	-1.933	0.021	0.166
4.25	0.136	-1.999	0.025	0.160
4.5	0.134	-2.013	0.030	0.164
4.75	0.128	-2.059	0.034	0.162
5	0.124	-2.091	0.039	0.163
5.5	0.111	-2.201	0.047	0.157
6.5	0.092	-2.384	0.061	0.153
8	0.071	-2.640	0.078	0.150
10	0.050	-3.001	0.094	0.144
12	0.034	-3.393	0.106	0.139
14	0.022	-3.806	0.111	0.133
16	0.014	-4.269	0.121	0.135
18	0.008	-4.794	0.123	0.131
20	0.004	-5.468	0.124	0.128
22	0.002	-6.066	0.128	0.131
24	0.001	-7.323	0.129	0.130

- **KIE Experiments**

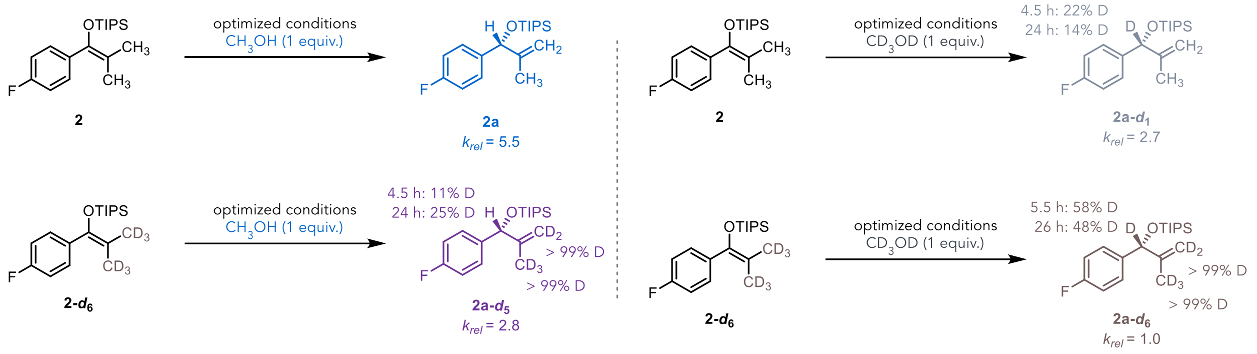

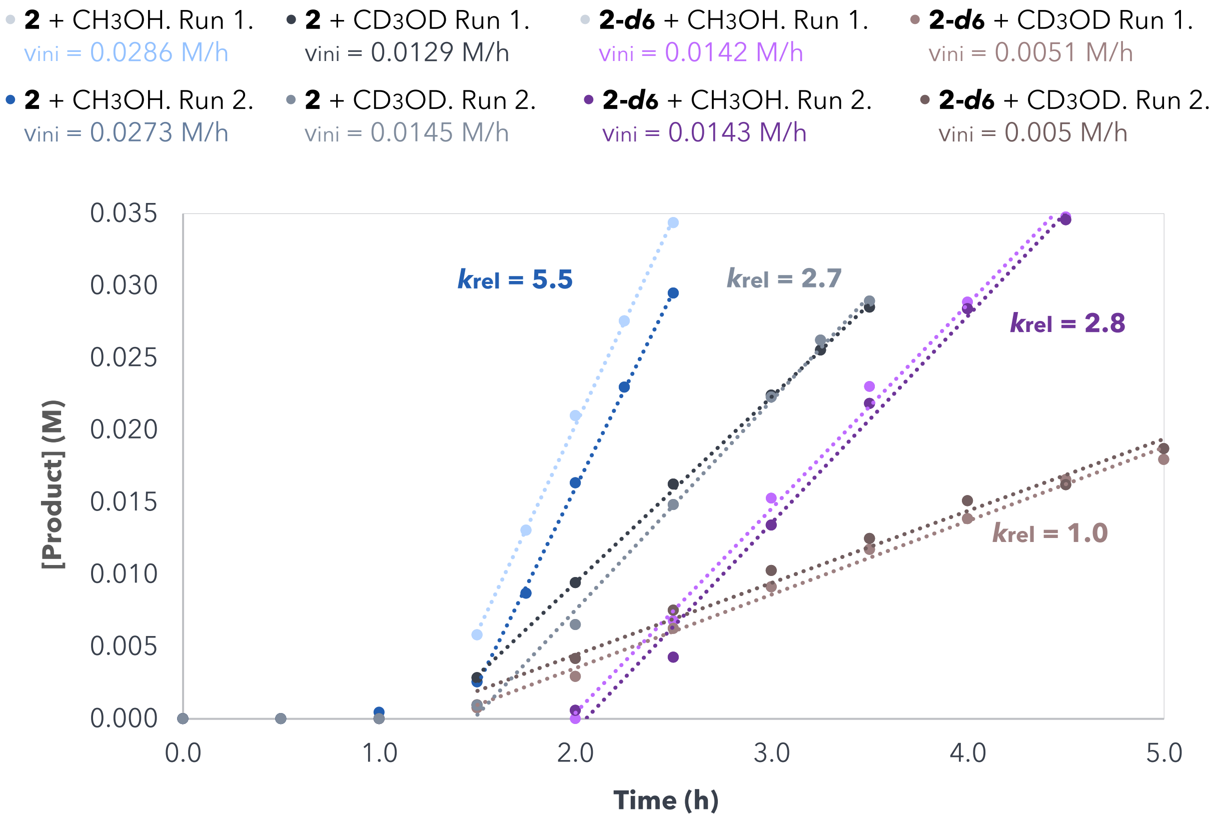

Figure S3. Kinetic traces corresponding to experiments shown in Figure 3B. Average v_{ini} values of duplicate runs were used to calculate k_{rel}. Deuterium incorporation data for "Run 2" of 2-d_6 + CD_3OD is shown.

Kinetics Data for Figure S3.

Table S11. Kinetics Data for 2 and CH_3OH (1 equiv). Run 1.

Time (h)	[2] (M)	[2a] (M)	[2]+[2a] (M)
0.00	0.20	0.000	0.20
0.50	0.20	0.000	0.20
1.00	0.20	0.000	0.20
**1.50**	**0.191**	**0.006**	**0.197**
1.75	0.177	0.013	0.190
2.00	0.170	0.021	0.191
2.25	0.161	0.028	0.188
2.50	0.154	0.034	0.188

Table S12. Kinetics Data for 2 and CH_3OH (1 equiv). Run 2.

Time (h)	[2] (M)	[2a] (M)	[2]+[2a] (M)
0.00	0.200	0.000	0.200
0.50	0.198	0.000	0.198
1.00	0.195	0.000	0.196
**1.50**	**0.176**	**0.002**	**0.179**
1.75	0.164	0.009	0.172
2.00	0.154	0.016	0.171
2.25	0.144	0.023	0.167
2.50	0.135	0.029	0.165

Table S13. Kinetics Data for 2 and CD_3OD (1 equiv). Run 1.

Time (h)	[2] (M)	[2a] (M)	[2]+[2a] (M)
0.00	0.200	0.000	0.200
0.50	0.200	0.000	0.200
1.00	0.192	0.000	0.192
**1.50**	**0.175**	**0.003**	**0.178**
2.00	0.160	0.009	0.169
2.50	0.145	0.016	0.161
3.00	0.138	0.022	0.160
3.25	0.134	0.026	0.159
3.50	0.131	0.029	0.160

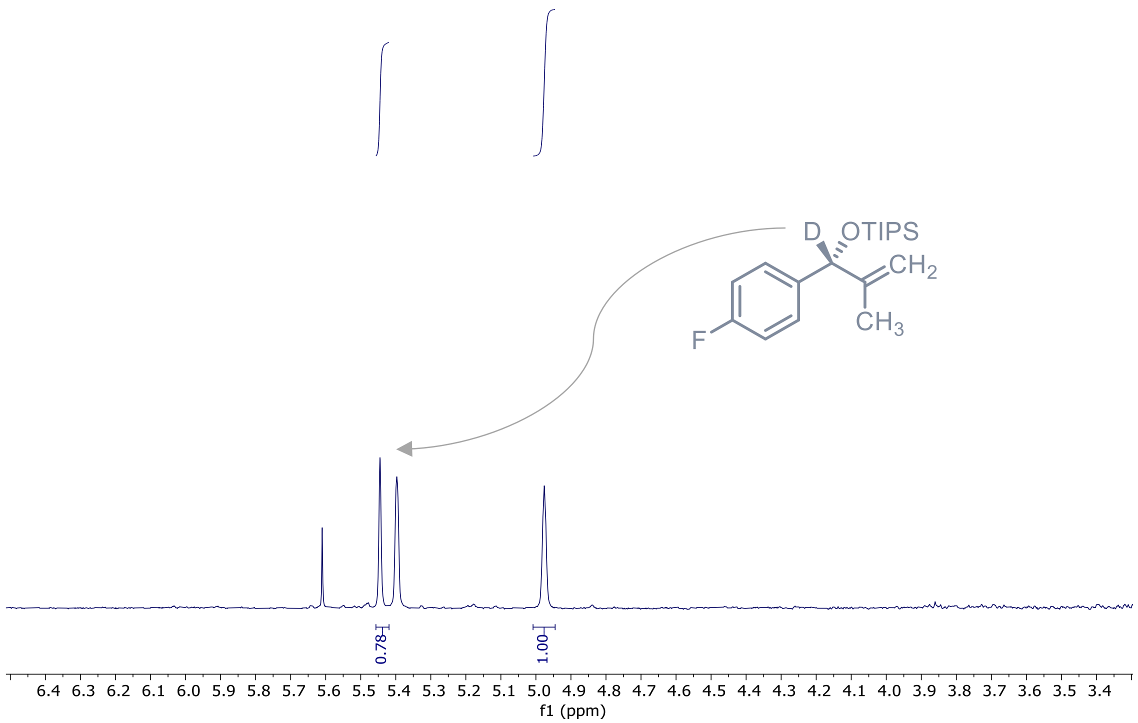

Figure S4. ^{1}H NMR (400 MHz, CD_3CN) at t = 4.5 h for determining D-incorporation (22%).

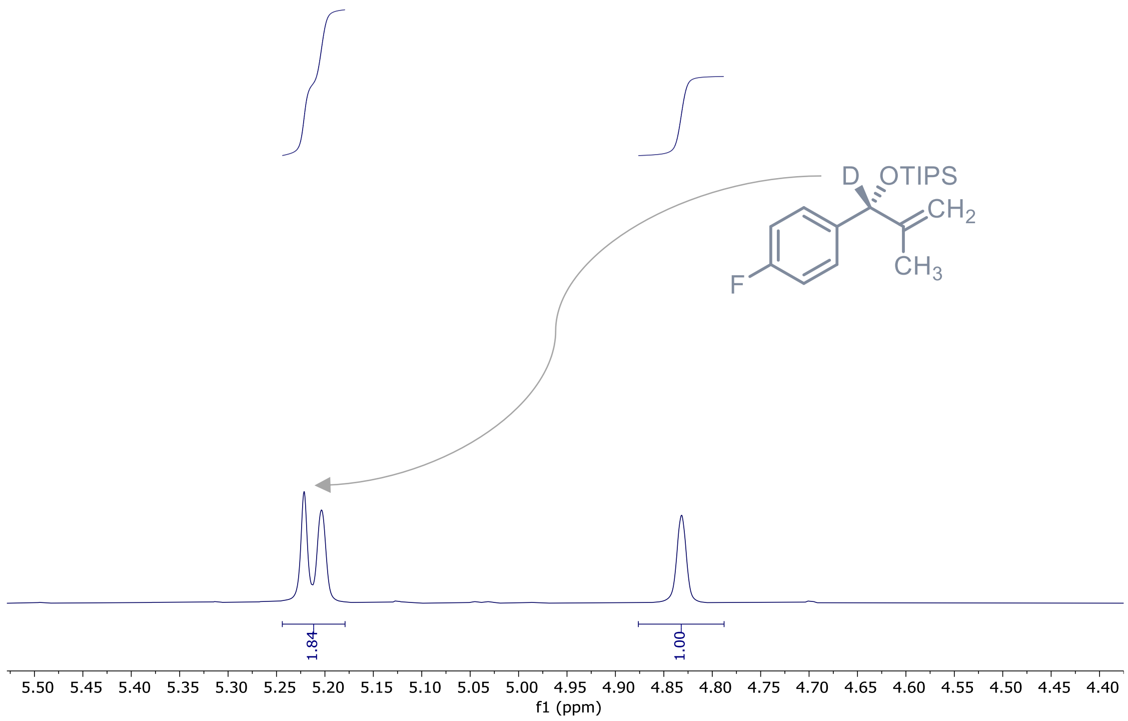

Figure S5. ^{1}H NMR (500 MHz, $CDCl_3$) at t = 24 h for determining D-incorporation (16%).

Table S14. Kinetics Data for 2 and CD_3OD (1 equiv). Run 2.

Time (h)	[2] (M)	[2a] (M)	[2]+[2a] (M)
0.00	0.200	0.000	0.200
0.50	0.200	0.000	0.200
1.00	0.199	0.000	0.199
**1.50**	**0.189**	**0.001**	**0.190**
2.00	0.170	0.007	0.177
2.50	0.156	0.015	0.171
3.00	0.143	0.022	0.165
3.25	0.139	0.026	0.165
3.50	0.133	0.029	0.162

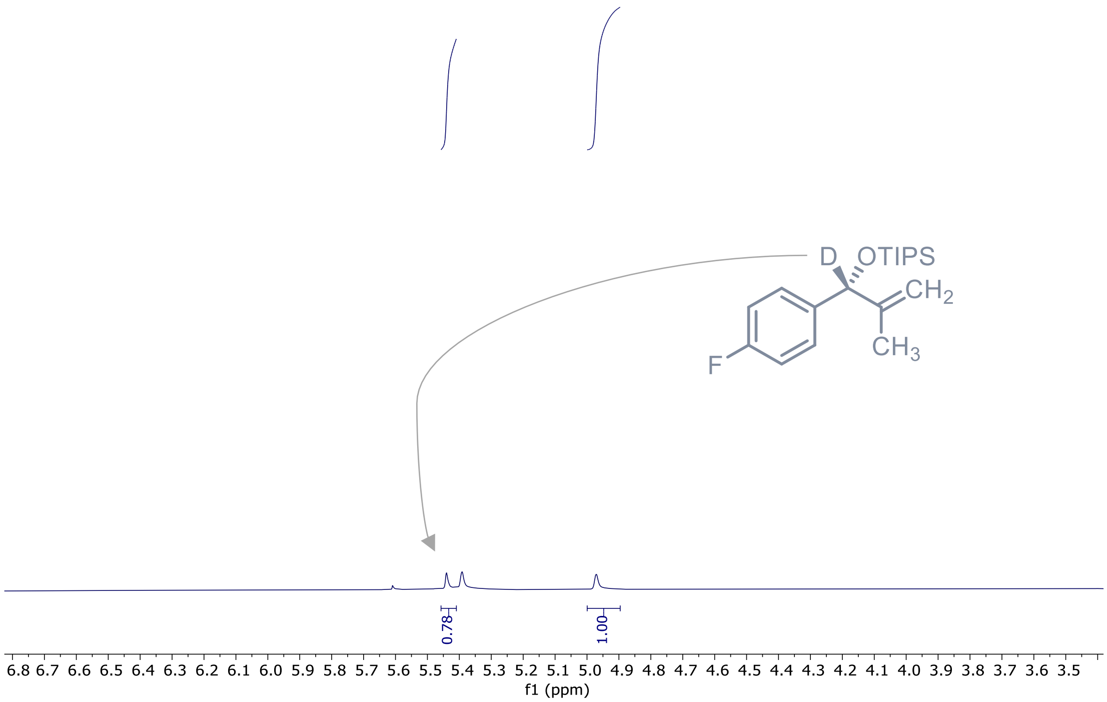

Figure S6. ^{1}H NMR (500 MHz, CD_3CN) at t = 4.5 h for determining D-incorporation (22%).

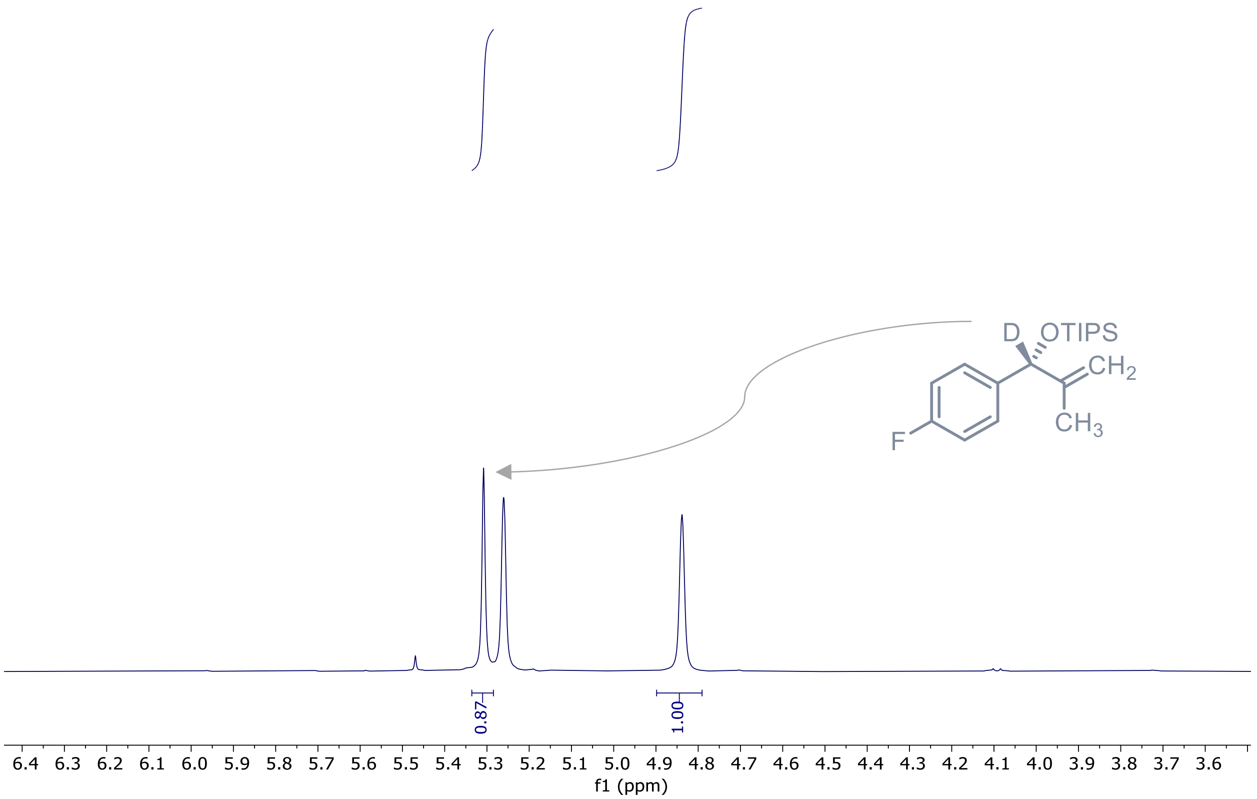

Figure S7. ^{1}H NMR (400 MHz, $CDCl_3$) at t = 24 h for determining D-incorporation (13%).

Table S15. Kinetics Data for 2-d_6 and CH_3OH (1 equiv). Run 1.

Time (h)	[2] (M)	[2a] (M)	[2]+[2a] (M)
0.000	0.200	0.000	0.200
1.000	0.196	0.000	0.196
**2.000**	**0.185**	**0.000**	**0.185**
2.500	0.166	0.007	0.173
3.000	0.148	0.015	0.163
3.500	0.138	0.023	0.161
4.000	0.126	0.029	0.155
4.500	0.119	0.035	0.154

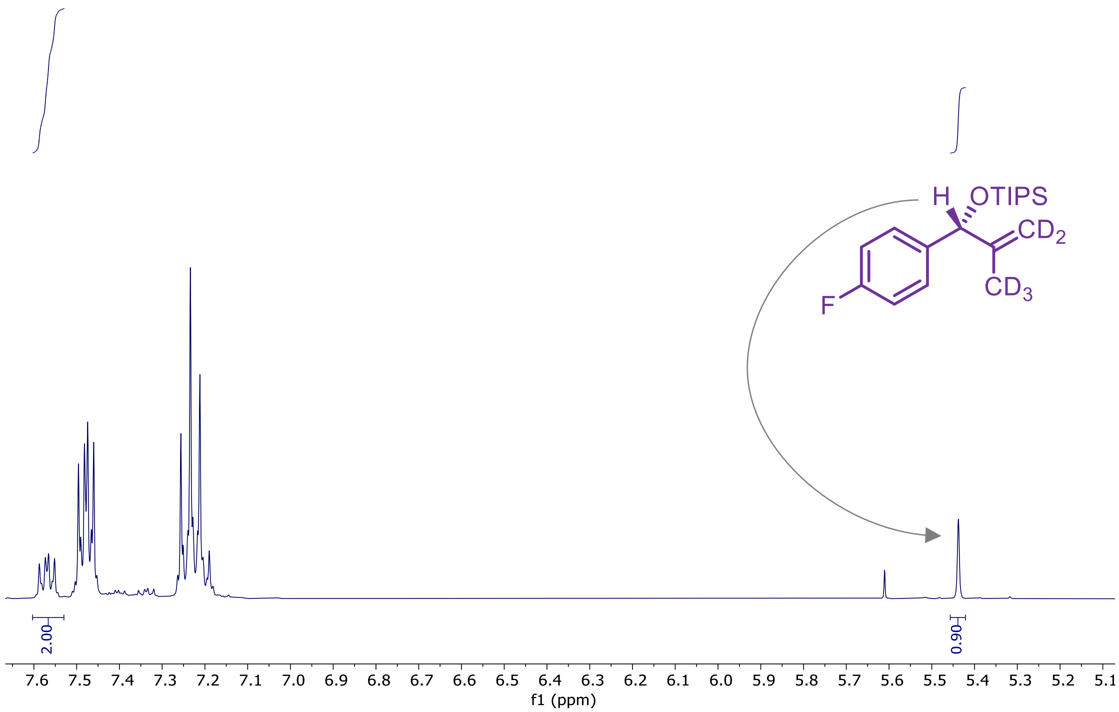

Figure S8. ^{1}H NMR (400 MHz, CD_3CN) at t = 4.5 h for determining D-incorporation (10%).

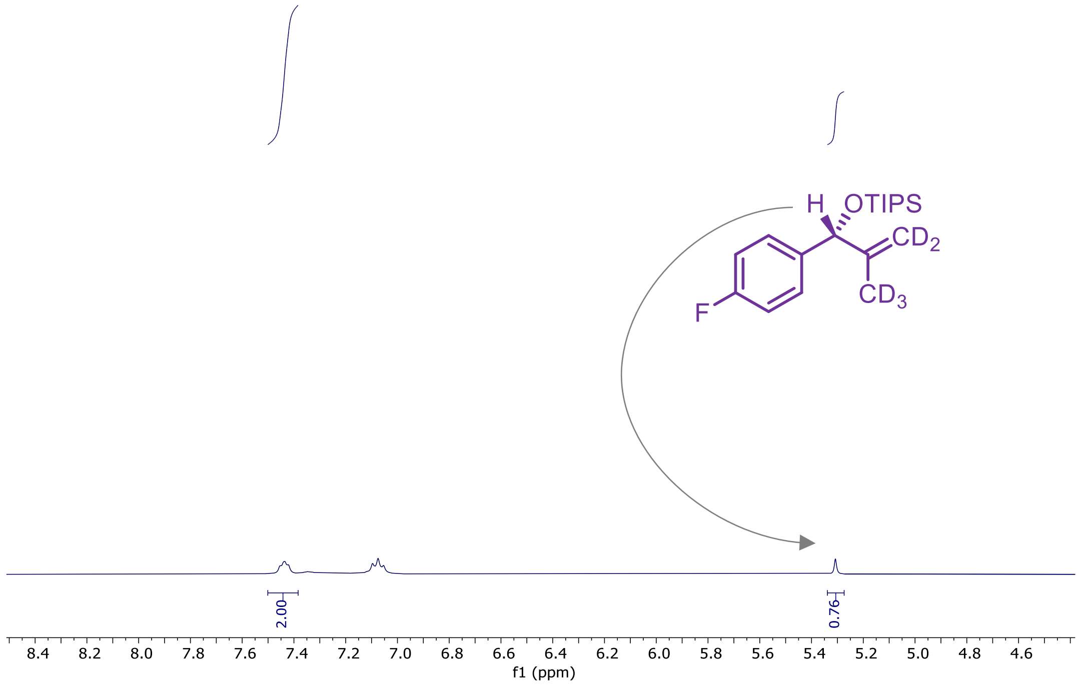

Figure S9. ^{1}H NMR (400 MHz, CD_3CN) at t = 24 h for determining D-incorporation (24%).

Table S16. Kinetics Data for 2-d_6 and CH_3OH (1 equiv). Run 2.

Time (h)	[2] (M)	[2a] (M)	[2]+[2a] (M)
0.000	0.200	0.000	0.200
1.000	0.200	0.000	0.200
**2.000**	**0.189**	**0.001**	**0.189**
2.500	0.172	0.004	0.177
3.000	0.153	0.013	0.167
3.500	0.144	0.022	0.166
4.000	0.132	0.028	0.160
4.500	0.123	0.035	0.157

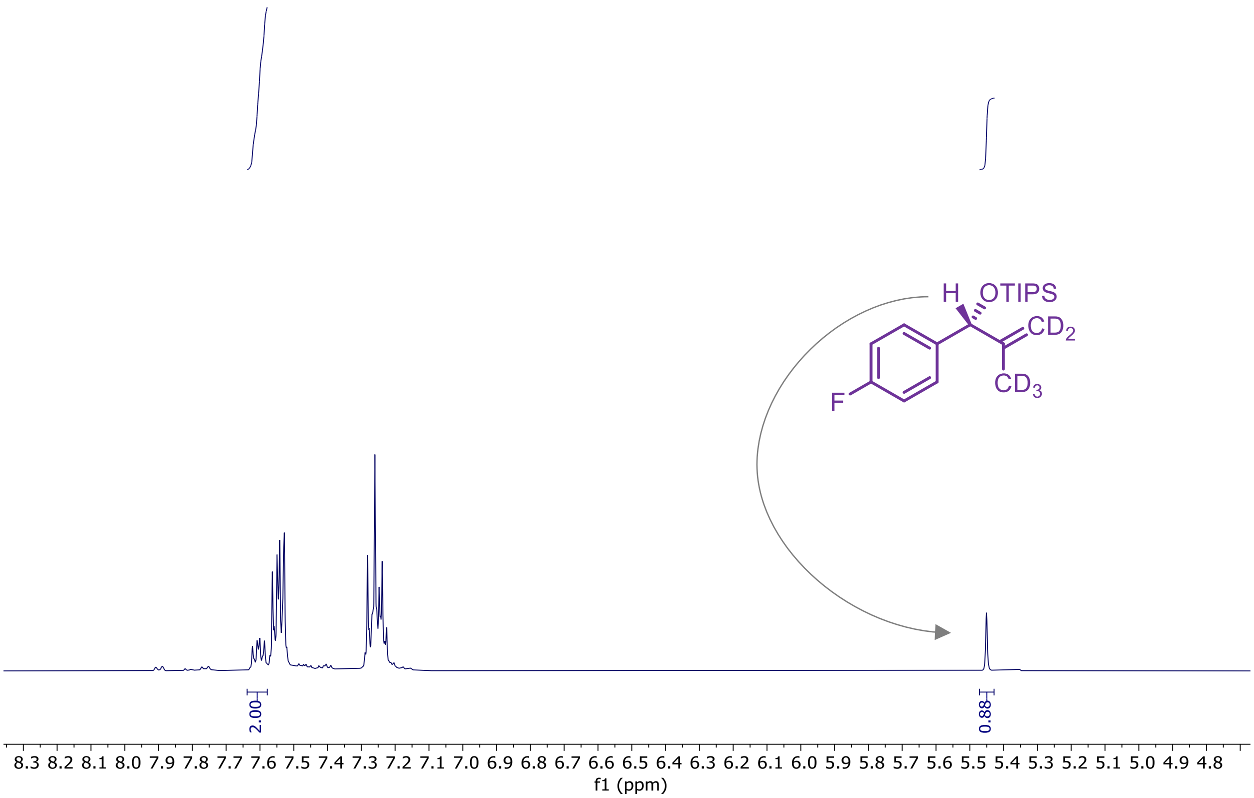

Figure S10. ^{1}H NMR (500 MHz, $CDCl_3$) at t = 4.5 h for determining D-incorporation (12%).

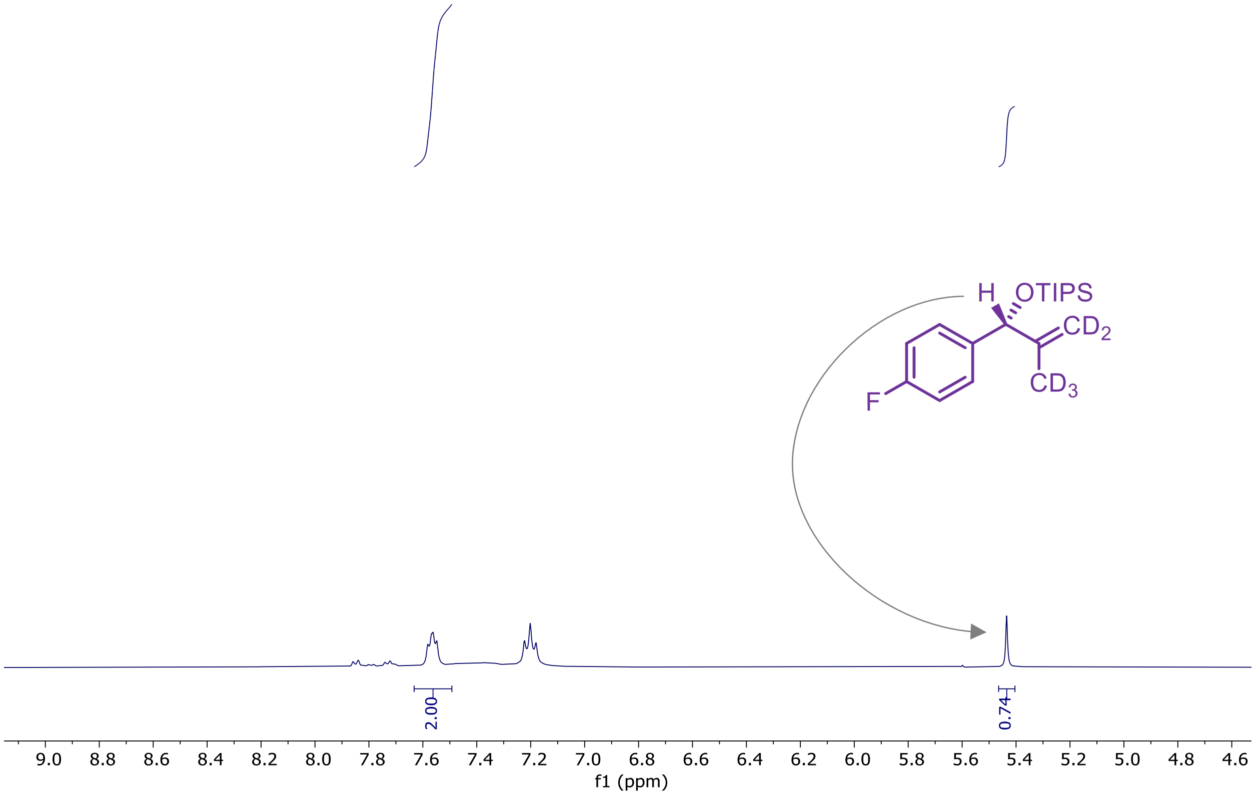

Figure S11. ^{1}H NMR (400 MHz, CD_3CN) at t = 24 h for determining D-incorporation (26%).

Table S17. Kinetics Data for 2-d_6 and CD_3OD (1 equiv). Run 1.

Time (h)	[2] (M)	[2a] (M)	[2]+[2a] (M)
0.00	0.200	0.000	0.200
1.00	0.194	0.000	0.194
1.50	0.184	0.001	0.185
2.00	0.171	0.003	0.174
2.50	0.160	0.006	0.166
3.00	0.153	0.009	0.162
3.50	0.148	0.012	0.160
4.00	0.145	0.014	0.159
4.50	0.142	0.017	0.159
5.00	0.138	0.018	0.156

Table S18. Kinetics Data for 2-d_6 and CD_3OD (1 equiv). Run 2.

Time (h)	[2] (M)	[2a] (M)	[2]+[2a] (M)
0.00	0.200	0.000	0.200
1.00	0.196	0.000	0.196
1.50	0.183	0.001	0.183
2.00	0.168	0.004	0.172
2.50	0.158	0.008	0.165
3.00	0.148	0.010	0.158
3.50	0.140	0.012	0.152
4.00	0.136	0.015	0.151
4.50	0.129	0.016	0.146
5.00	0.127	0.019	0.146

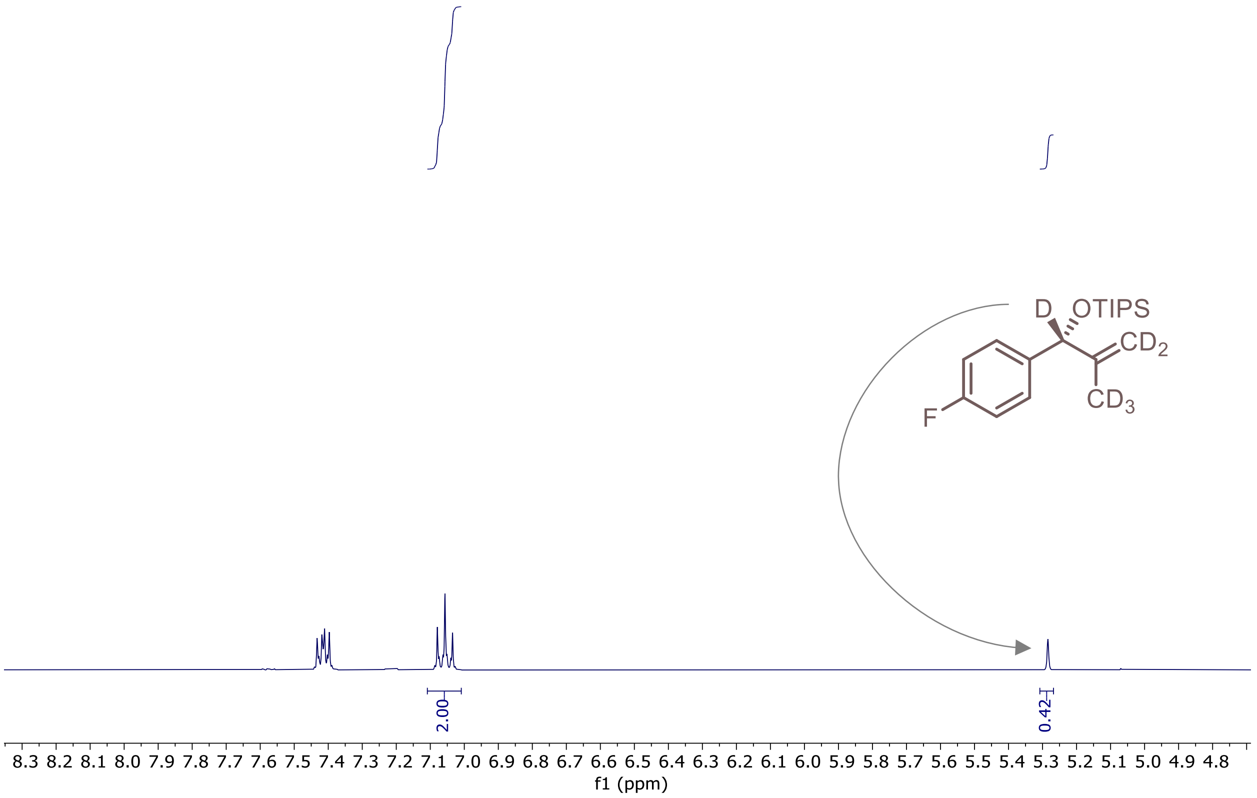

Figure S12. ^{1}H NMR (400 MHz, CD_3CN) at t = 5 h for determining D-incorporation (58%).

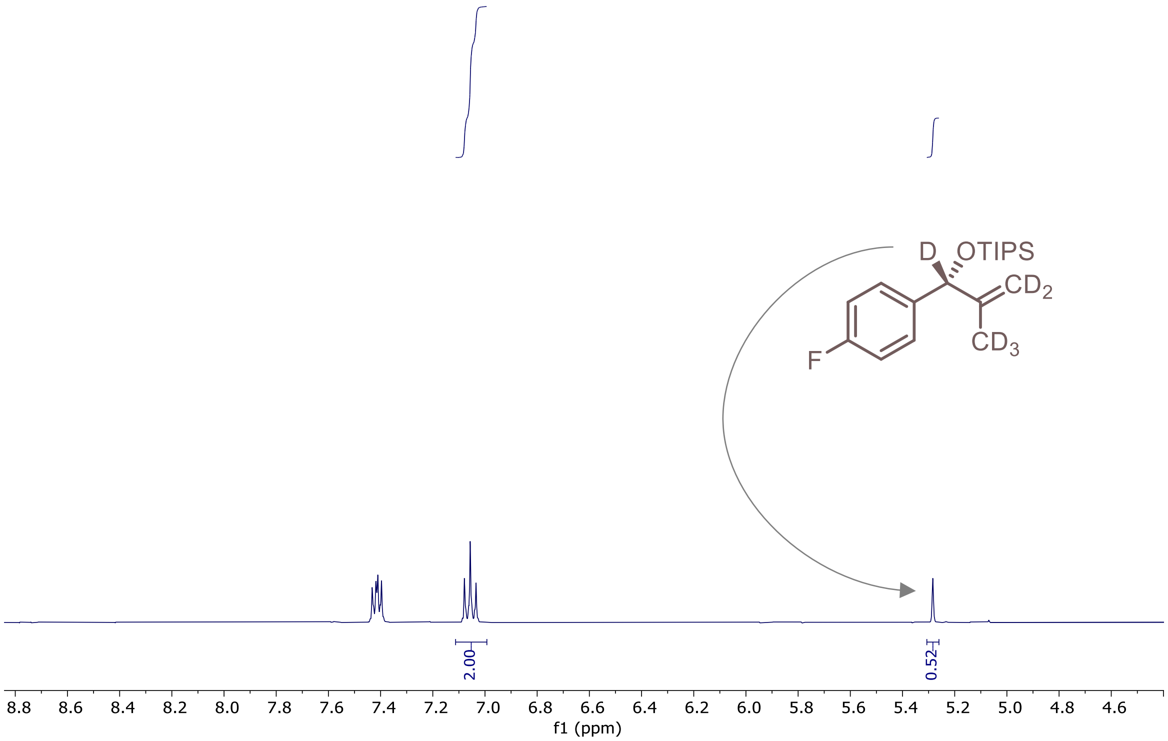

Figure S13. ^{1}H NMR (400 MHz, CD_3CN) at t = 24 h for determining D-incorporation (48%).

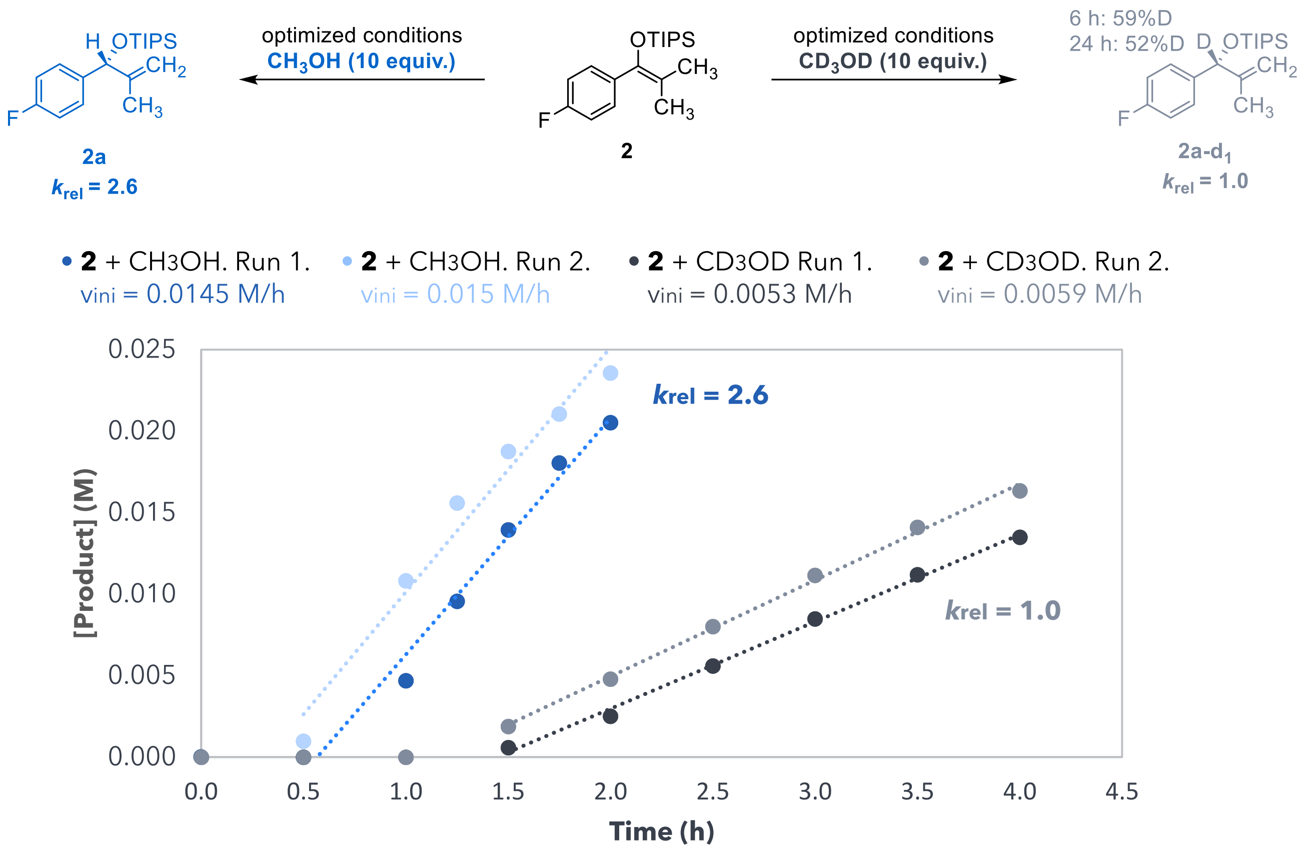

Figure S14. KIE experiments with 10 equiv CH_3OH or CD_3OD. Average v_{ini} values of duplicate runs were used to calculate k_{rel}.

Table S19. Kinetics Data for 2 and CH_3OH (10 equiv). Run 1.

Time (h)	[2] (M)	[2a] (M)	[2]+[2a] (M)
0.00	0.200	0.000	0.200
0.50	0.196	0.000	0.196
1.00	0.170	0.005	0.175
1.25	0.160	0.010	0.170
1.50	0.147	0.014	0.161
1.75	0.137	0.018	0.155
2.00	0.125	0.021	0.145

Table S20. Kinetics Data for 2 and CH_3OH (10 equiv). Run 2.

Time (h)	[2] (M)	[2a] (M)	[2]+[2a] (M)
0.00	0.200	0.000	0.200
0.50	0.191	0.001	0.192
1.00	0.158	0.011	0.169
1.25	0.146	0.016	0.161
1.50	0.132	0.019	0.151
1.75	0.124	0.021	0.145
2.00	0.117	0.024	0.141

Table S21. Kinetics Data for 2 and CD_3OD (10 equiv). Run 1.

Time (h)	[2] (M)	[2a] (M)	[2]+[2a] (M)
0.00	0.200	0.000	0.200
0.50	0.200	0.000	0.200
1.00	0.197	0.000	0.197
1.50	0.186	0.001	0.187
2.00	0.169	0.003	0.172
2.50	0.158	0.006	0.164
3.00	0.146	0.008	0.155
3.50	0.135	0.011	0.146
4.00	0.126	0.013	0.139

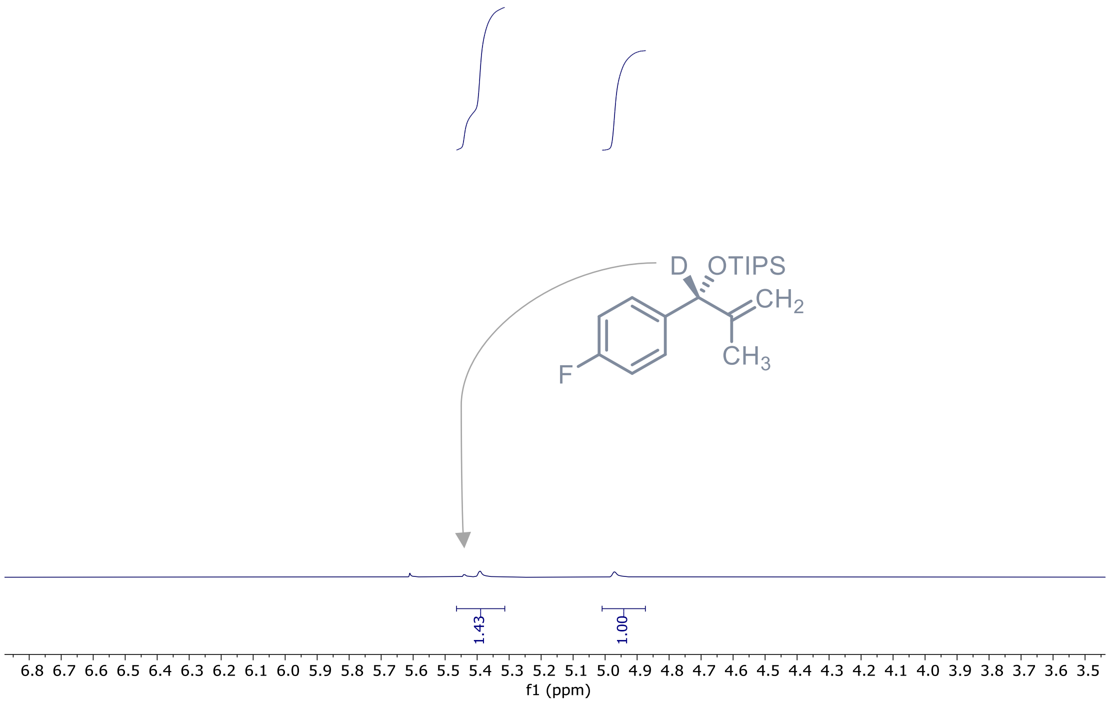

Figure S15. ^{1}H NMR (400 MHz, CD_3CN) at t = 6 h for determining D-incorporation (57%).

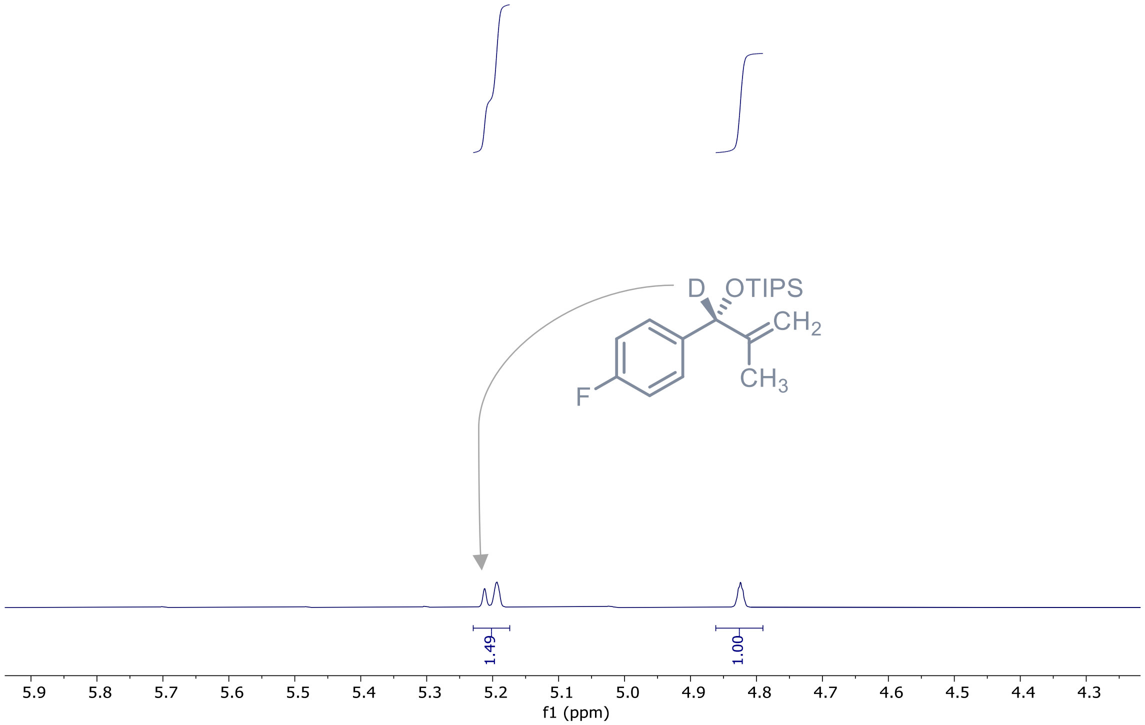

Figure S16. ^{1}H NMR (500 MHz, $CDCl_3$) at t = 24 h for determining D-incorporation (51%).

Table S22. Kinetics Data for 2 and CD_3OD (10 equiv). Run 2.

Time (h)	[2] (M)	[2a] (M)	[2]+[2a] (M)
0.00	0.200	0.000	0.200
0.50	0.197	0.000	0.197
1.00	0.191	0.000	0.191
1.50	0.172	0.002	0.174
2.00	0.155	0.005	0.160
2.50	0.146	0.008	0.154
3.00	0.135	0.011	0.146
3.50	0.128	0.014	0.142
4.00	0.118	0.016	0.135

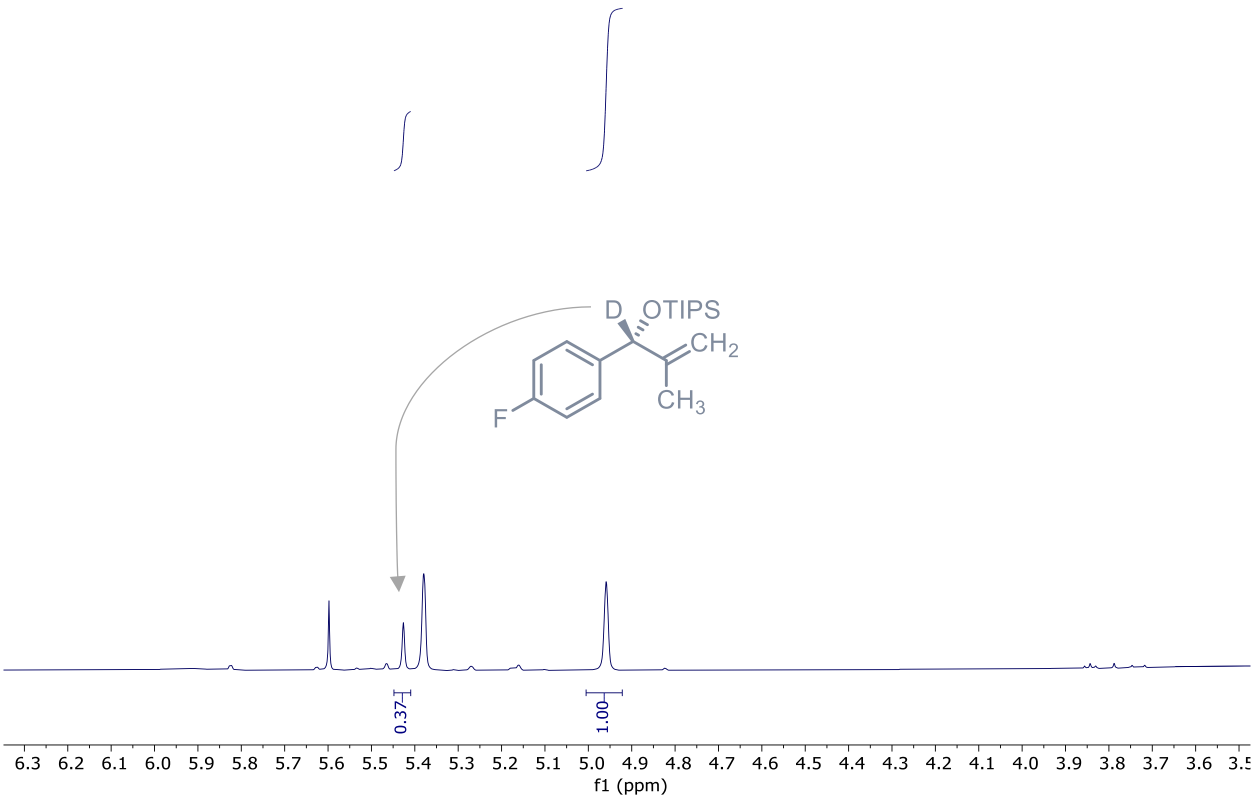

Figure S17. ^{1}H NMR (500 MHz, CD_3CN) at t = 6 h for determining D-incorporation (63%).

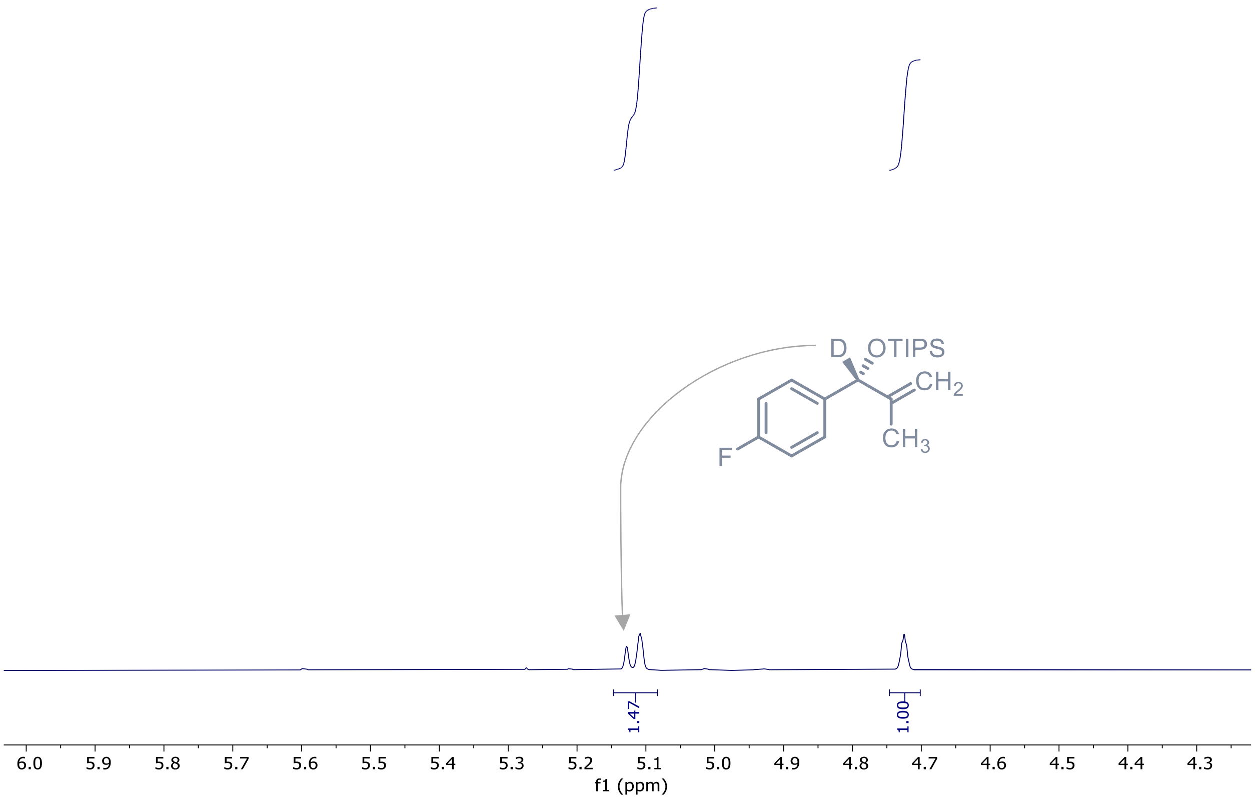

Figure S18. ^{1}H NMR (500 MHz, CD_3CN) at t = 24 h for determining D-incorporation (53%).

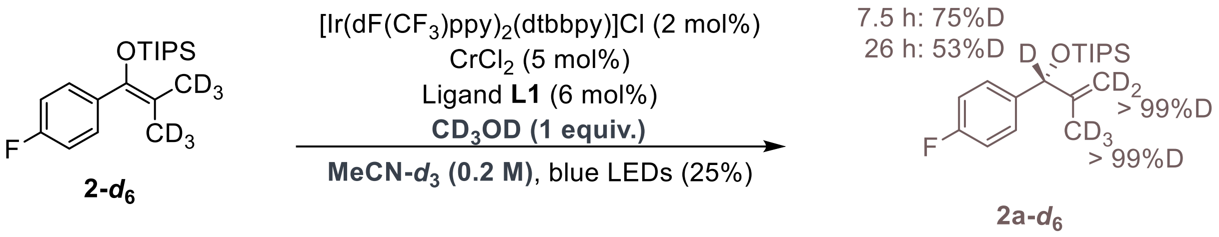

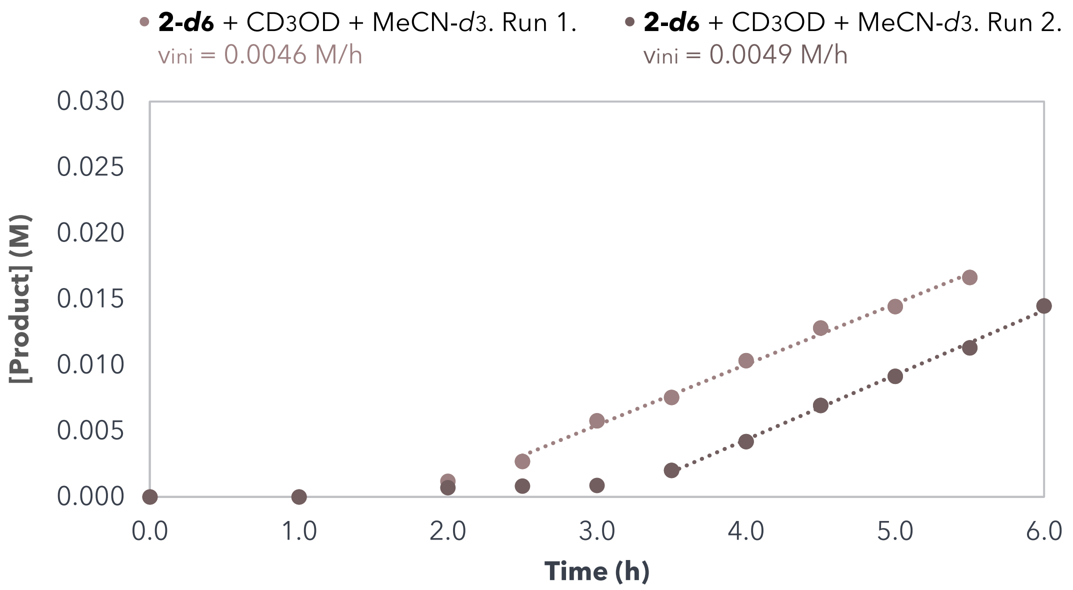

Figure S19. Kinetic traces corresponding to initial rate experiments using MeCN-d_3 solvent. Deuterium incorporation at t = 7.5 h was taken as the value from Figure S21.

Table S23. Kinetics Data for 2-d_6 and CD_3OD (1 equiv) using CD_3CN solvent. Run 1.

Time (h)	[2] (M)	[2a] (M)	[2]+[2a] (M)
0.00	0.200	0.000	0.200
1.00	0.154	0.000	0.154
2.00	0.128	0.001	0.129
2.50	0.118	0.003	0.121
3.00	0.110	0.006	0.116
3.50	0.101	0.008	0.108
4.00	0.095	0.010	0.106
4.50	0.090	0.013	0.103
5.00	0.085	0.014	0.100
5.50	0.080	0.017	0.097

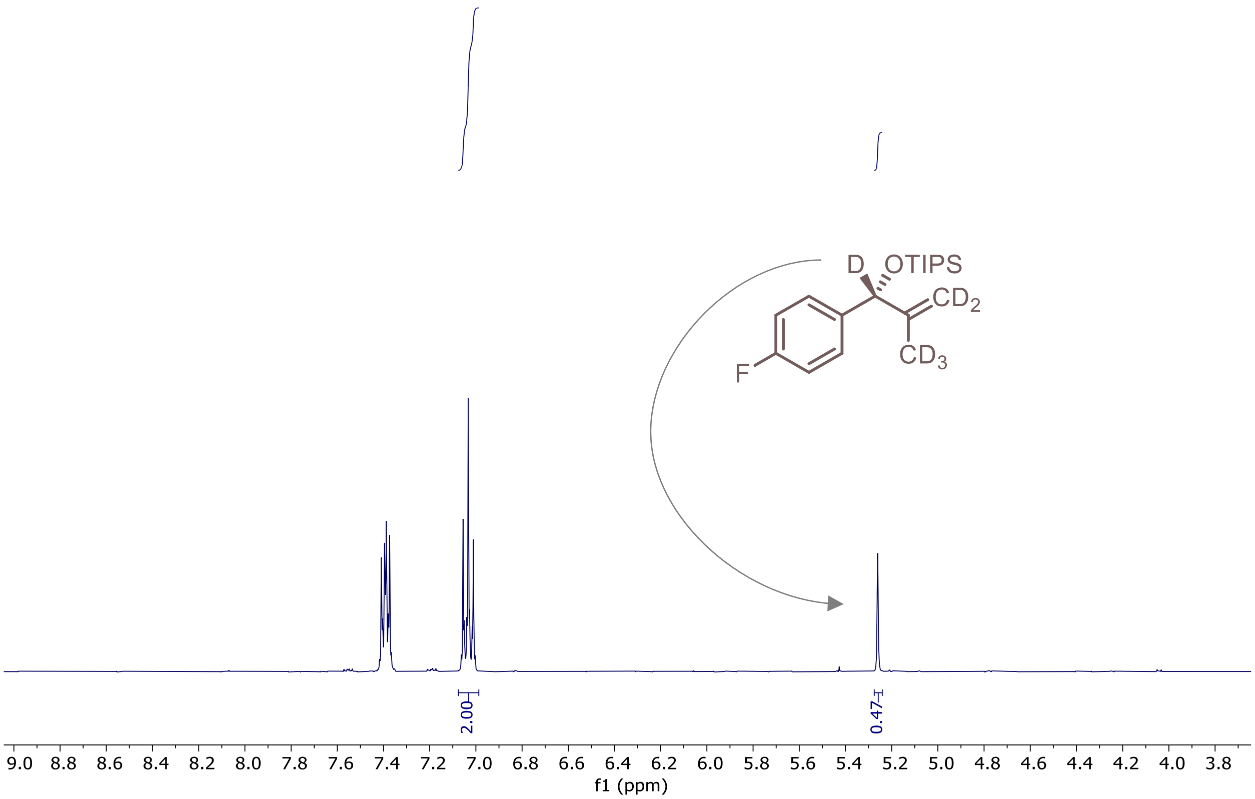

Figure S20. ^{1}H NMR (400 MHz, CD_3CN) at t = 26 h for determining D-incorporation (53%).

Table S24. Kinetics Data for 2-*d*$_6$ and CD_3OD (1 equiv) using CD_3CN solvent. Run 2.

Time (h)	[2] (M)	[2a] (M)	[2]+[2a] (M)
0.00	0.200	0.000	0.200
1.00	0.179	0.000	0.179
2.00	0.165	0.001	0.166
2.50	0.156	0.001	0.156
3.00	0.143	0.001	0.144
3.50	0.132	0.002	0.134
4.00	0.123	0.004	0.127
4.50	0.114	0.007	0.121
5.00	0.107	0.009	0.116
5.50	0.099	0.011	0.110
6.00	0.094	0.014	0.108

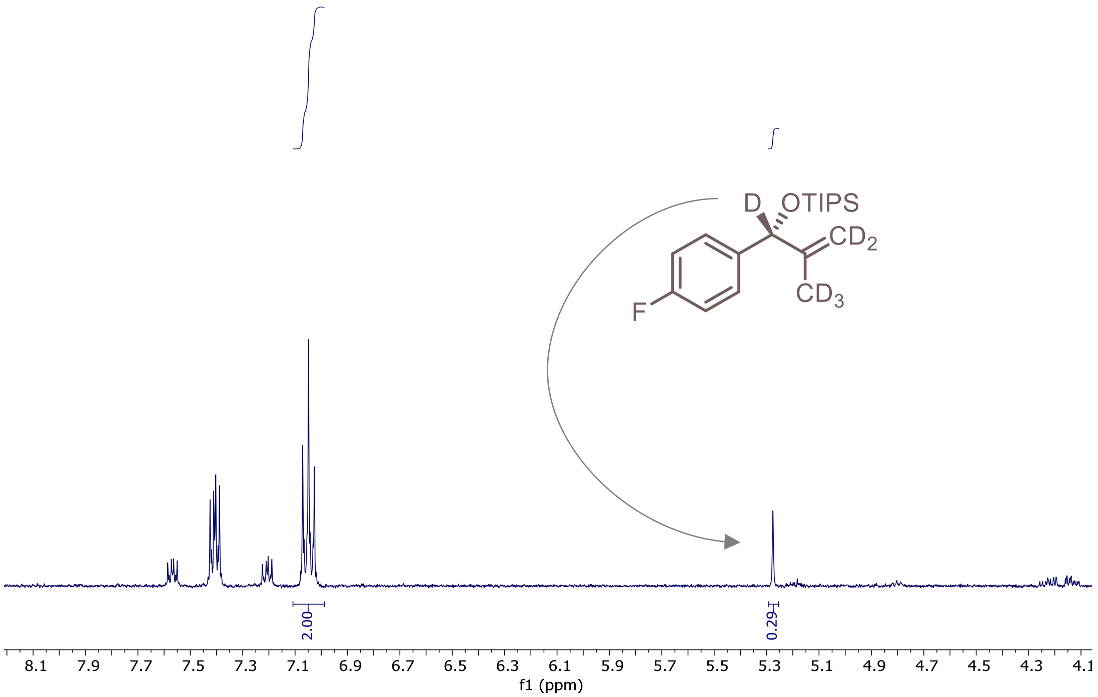

Figure S21. ^{1}H NMR (400 MHz, CD_3CN) at t = 7.5 h for determining D-incorporation (71%).

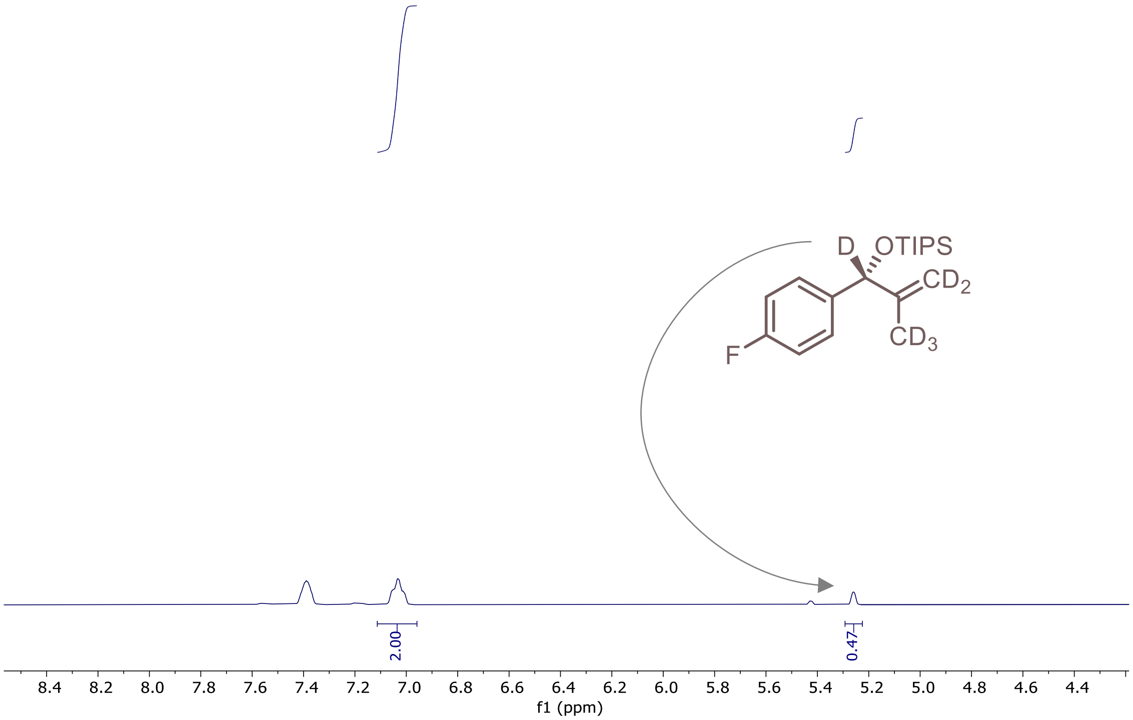

Figure S22. ^{1}H NMR (400 MHz, CD_3CN) at t = 26 h for determining D-incorporation (53%).

- **Non-Linear Effects (NLE) Study**

Inside a N_2-filled glove box, a stock solution ((*S*)-stock) was prepared with $CrCl_2$ (4.3mg, 5 mol%), (*S*,*S*)-**L1** (13.5 mg, 6 mol%), [Ir(dF(CF_3)ppy)$_2$(dtbbpy)]Cl (14.2 mg, 2 mol%), and MeOH (28.4 µL, 1 equiv) in degassed and anhydrous MeCN (3.5 mL). Another stock solution ((*R*)-stock) was prepared similarly with (*R*,*R*)-**L1**. Six oven-dried 2-dram vials equipped with magnetic stir bars were each charged with enol ether **1** (0.1 mmol, 1 equiv) and scalemic mixtures of the two stock solutions according to the following table. The ee of **L1** was calculated based on the ratios of the stock solutions added. The vials were sealed with caps fitted with PTFE septa. Electrical tape was used to seal the sides of the caps. The vials were removed from the glovebox and placed approximately 2.5 cm away from 34W Kessil PR160-456 nm blue LED lamps (25% intensity) on a stir plate (see Figure S1). The reaction mixture was stirred and irradiated for 24 hours. During irradiation, two rotary fans were placed adjacent to the lamps to cool the reaction setup. The temperature of the setup was measured to be 28±5 °C. The crude reaction mixture was diluted with ethyl acetate and filtered through a pipette silica plug eluting with ethyl acetate. The filtrate was concentrated under reduced pressure. Yields were assessed by quantitative ^{1}H NMR spectroscopy using 1,3,5-trimethoxybenzene as an internal standard. Samples were purified by preparatory TLC. The enantioenrichment was determined by HPLC on a chiral stationary phase.

L1 ee	(*S*)-Stock (µL)	(*R*)-Stock (µL)	1a ee
0%	250	250	–0.8%
20%	300	200	13.6%
40%	350	150	32.4%
60%	400	100	50.2%
80%	450	50	67.2%
100%	500	0	87.9%

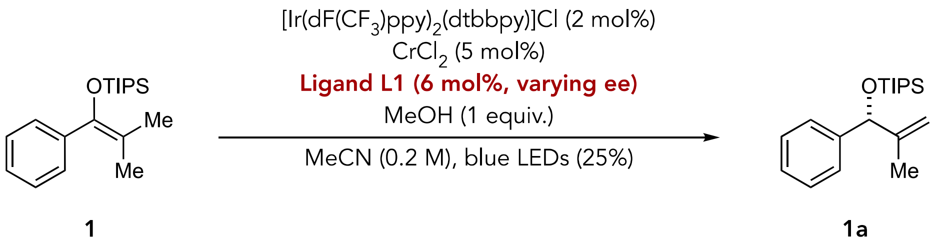

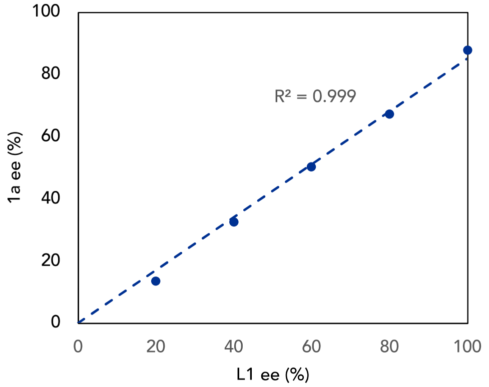

Figure S23. Outcome of the NLE study.

- **Cyclic Voltammetry Experiments**

Cyclic voltammetry experiments were performed on a CH Instruments 600E potentiostat using CHI600E software. Experiments were carried at a scan rate of 0.1 V/s in a 25 mL three-neck flask equipped with glassy carbon as the working electrode, platinum mesh as the counter electrode, and Ag wire as the reference electrode. The voltammogram was collected with ~10 mg substrate, 0.1 M NBu_4PF_6 as electrolyte in anhydrous MeCN under nitrogen. All values are referenced to Fc^+/Fc.

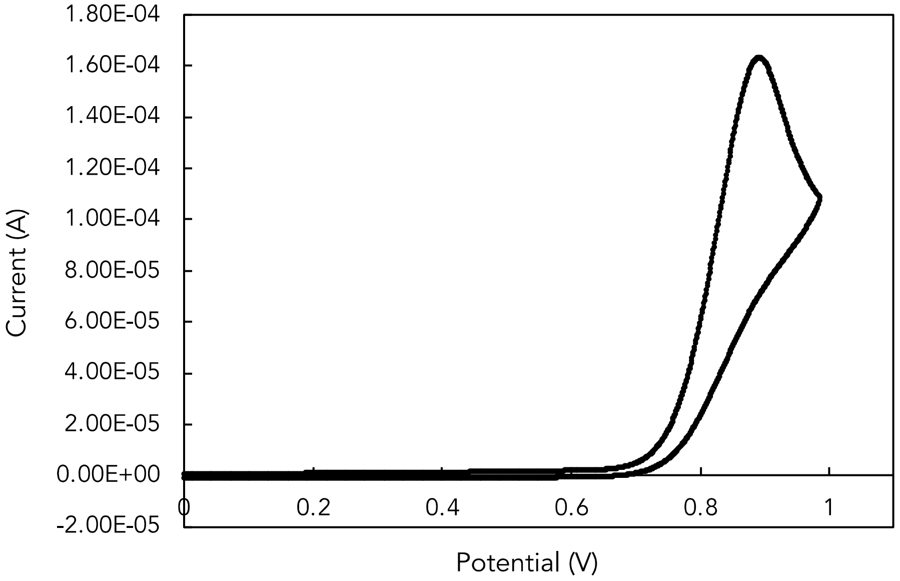

Figure S24. Cyclic voltammogram of **1**. $E_p = 0.89$ V vs Fc^+/Fc. $E_{p/2} = 0.82$ V vs Fc^+/Fc.

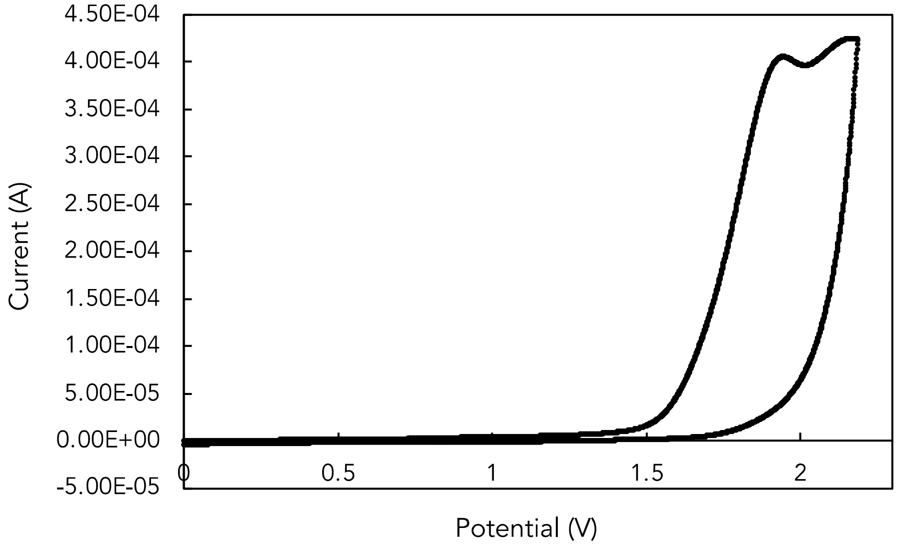

Figure S25. Cyclic voltammogram of **1a**. $E_p = 1.94$ V vs Fc^+/Fc. $E_{p/2} = 1.76$ V vs Fc^+/Fc.

10.Computational Studies

Conformational searches were performed either by manually perturbing the structures or using MacroModel version 11.8[14] and the OPLS3 forcefield.[15] All DFT computations were performed using Gaussian 16 Rev. A.03.[16] Frequency calculations were performed on all structures and confirmed to contain no imaginary frequency for ground states.

Geometry optimizations and frequency calculations for all ground-state structures were performed using the B3LYP-D3(BJ)/6-311G(d,p)-SDD(Cr) level of theory. The single-point energy (SPE) of these structures were calculated at B3LYP-D3(BJ)/6-311+G(d,p)-SDD(Cr)/SMD(MeCN) level of theory. Thermal free energy corrections to the SPE were carried out using "Goodvibes" Python code.

- **pKa Calculation**

The BDFE of the allylic C–H bond of **1** was calculated using the following isodesmic reaction with a BDFE value of 79.1 kcal/mol for the allylic C–H bond in propene.

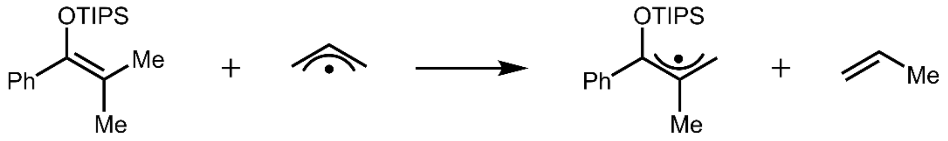

1 S1• 1• S1

BDFE (C-H(1)) = [G(1•) - (G(1)] - [G(S1•) - G(S1)] + 79.1 kcal/mol
= 75.1 kcal/mol

The G values are reported below along with the calculated coordinates

The pKa of the radical cation is estimated using the following thermodynamic cycle[17]:

$$\text{BDFE (C–H)} = 1.37\text{pKa} + 23.06\ E^{o} + 52.6$$

pKa of the radical cation is calculated to be 2.6 with BDFE (C–H (**1**)) = 75.1 kcal/mol and E^{o} = 0.82 V (**Figure S24**).

Coordinates of Ground States

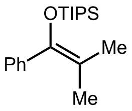

1

E = -1108.427343 Hartree; G (298.15 K) = -1108.015306 Hartree

E_SPE = -1108.446455 Hartree; G_SPE (298.15 K) = -1108.034419 Hartree

0 1

C	2.86166900	2.88325900	-0.31291000
H	2.87353600	3.45283800	0.62470600
H	3.60808200	2.09433000	-0.23887400
C	1.48239900	2.36011400	-0.61199400
C	1.17571600	1.05286200	-0.60981700
C	0.46087900	3.40859500	-0.95729200
H	-0.48376100	2.96831700	-1.26943700
H	0.27395000	4.06726600	-0.10004300
H	0.82869800	4.04737600	-1.76809300
O	-0.07747000	0.62299900	-1.00000700
Si	-1.33512900	0.03162500	-0.02712600
C	-1.59026600	1.28303300	1.38272500
H	-1.51200000	2.25423300	0.87687000
C	-0.85750800	-1.69158300	0.61015100
H	0.15373600	-1.56028100	1.01468300
C	-2.81248400	0.05979400	-1.21750700
H	-3.14826000	1.10666200	-1.20272200
C	-0.76676000	-2.73149000	-0.52011300
H	-0.15636600	-2.37984400	-1.35247100
H	-0.31878200	-3.66075600	-0.15251100
H	-1.75738200	-2.97853800	-0.91146600
C	-1.74854900	-2.21509600	1.75078700
H	-2.78630600	-2.34010900	1.43073500
H	-1.39459700	-3.19473900	2.09013000
H	-1.74706400	-1.55043400	2.61680500
C	-0.48033700	1.23160600	2.44662500
H	0.50426800	1.39370800	2.00627500
H	-0.63713300	2.00694400	3.20483800
H	-0.45970000	0.26930800	2.96540500
C	-2.98275200	1.22641900	2.03542400
H	-3.07800100	2.00732200	2.79788100
H	-3.78220600	1.38167200	1.30743600
H	-3.16513900	0.26895600	2.53036900
C	-2.41707400	-0.27251400	-2.66728800
H	-2.10031500	-1.31466000	-2.76219500
H	-3.26983300	-0.12754500	-3.33972500
H	-1.59478800	0.35577000	-3.01183300
C	-3.99643200	-0.81395800	-0.76608500
H	-4.32260700	-0.59225300	0.25231600
H	-4.85677400	-0.66471500	-1.42721500
H	-3.74048200	-1.87579100	-0.80620300
C	2.10967200	-0.05737300	-0.29020200
C	2.25534100	-1.11266400	-1.19917400
C	2.81170400	-0.11319900	0.91842200
C	3.08389400	-2.19004100	-0.91069900
H	1.70879100	-1.07598700	-2.13331700
C	3.63868300	-1.19490800	1.21104400
H	2.69406500	0.68546400	1.64007300
C	3.77670500	-2.23698000	0.29840800
H	3.18873000	-2.99606600	-1.62789800
H	4.16808800	-1.22645300	2.15645500
H	4.41764600	-3.08048700	0.52696900
H	3.17647300	3.57649800	-1.10115700

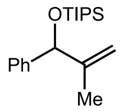

1a

E = -1108.415915 Hartree; G (298.15 K) = -1108.004886 Hartree

E_SPE = -1108.436649 Hartree; G_SPE (298.15 K) = -1108.025620 Hartree

0 1

C	1.43125900	3.27842300	-0.80096600
H	1.95317600	4.20113000	-1.02904300
H	0.50101300	3.07783000	-1.31364800
C	1.91628100	2.41976300	0.09118000
C	1.19869700	1.12482000	0.42670600
C	3.19794200	2.64841700	0.84560100
H	3.93785000	1.87762600	0.61004900
H	3.62498200	3.62309000	0.60635100
H	3.03025400	2.60441700	1.92795200
O	-0.01859000	1.03462800	-0.29041600
H	1.00036800	1.14220600	1.50775400
Si	-1.39624900	0.13525200	0.06858100
C	-1.82224200	0.43811700	1.90420700
H	-1.51446900	1.48142300	2.06464600
C	-1.05664200	-1.70058200	-0.27674300
H	-0.15218900	-1.92149000	0.30461000
C	-2.69739600	0.92336300	-1.06839500
H	-2.99060600	1.84361600	-0.54355000
C	-0.72504900	-1.96604100	-1.75521700
H	0.05210700	-1.29595600	-2.12441600
H	-0.36981500	-2.99293600	-1.89324200
H	-1.60906400	-1.83890000	-2.38671500
C	-2.16486600	-2.65465400	0.20424000
H	-3.09234200	-2.50757600	-0.35424600
H	-1.86134100	-3.69702500	0.05753600
H	-2.39641400	-2.52902000	1.26383900
C	-1.02511000	-0.43921600	2.88640000
H	0.05126400	-0.39617700	2.71222200
H	-1.20518500	-0.12403300	3.92005200
H	-1.31883400	-1.48915900	2.81237800
C	-3.32531000	0.35671500	2.22432800
H	-3.50766400	0.60118400	3.27661400
H	-3.91229700	1.05108500	1.61952100
H	-3.72007900	-0.64908200	2.05389800
C	-2.12245700	1.34148900	-2.43366900
H	-1.83822000	0.46920800	-3.02817400
H	-2.86962100	1.89982700	-3.00854900
H	-1.23612600	1.96619600	-2.32330800
C	-3.96423000	0.07205900	-1.26451500
H	-4.41643800	-0.24169200	-0.32155800
H	-4.72104400	0.63421900	-1.82215600
H	-3.74426000	-0.82984400	-1.84176400
C	2.07893000	-0.08151700	0.13313500
C	2.43377800	-0.36504700	-1.18757700
C	2.52831000	-0.91911400	1.15129200
C	3.20975600	-1.47880000	-1.48414300
H	2.09055500	0.29487900	-1.97543100
C	3.30945800	-2.03675800	0.85818600
H	2.26567900	-0.70046000	2.18065200
C	3.64734900	-2.32086600	-0.46100400
H	3.47510900	-1.69370400	-2.51297900
H	3.64852000	-2.68403700	1.65881500
H	4.25058700	-3.19082400	-0.69319000

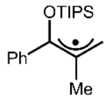

1•

E = -1107.784707 Hartree; G (298.15 K) = -1107.385692 Hartree
E_SPE = -1107.804990 Hartree; G_SPE (298.15 K) = -1107.405975 Hartree

0 2

C	2.55536200	3.16577900	-0.18631500
H	2.61124400	4.23649500	-0.03714800
H	3.48759900	2.63695900	-0.32154600
C	1.33879100	2.54158400	-0.25553200
C	1.17642000	1.14071400	-0.43859300
C	0.07755200	3.37652600	-0.20075500
H	-0.59395500	3.04259900	0.59465500
H	0.31988500	4.42497900	-0.02497400
H	-0.47899900	3.30221300	-1.13734100
O	-0.06235100	0.70723800	-0.81548800
Si	-1.28527900	-0.08034700	0.07029400
C	-0.87578900	0.14719500	1.91320200
H	-0.41219000	1.14204100	1.95843000
C	-1.26062900	-1.90452600	-0.43933500
H	-0.23440400	-2.22137800	-0.21477800
C	-2.85396800	0.86325200	-0.43362600
H	-2.81756700	1.78197600	0.16842500
C	-1.48966500	-2.10042400	-1.94733800
H	-0.84743400	-1.45310700	-2.54939400
H	-1.29114700	-3.13718500	-2.23978800
H	-2.52530800	-1.87932900	-2.22019700
C	-2.20850700	-2.80152100	0.37668500
H	-3.25671100	-2.56392000	0.18230300
H	-2.06026900	-3.85390100	0.11165000
H	-2.04371400	-2.71030600	1.45240600
C	0.16278800	-0.86345200	2.43283100
H	1.06464400	-0.88713100	1.82035600
H	0.46395800	-0.60894300	3.45469600
H	-0.24678200	-1.87669400	2.45940400
C	-2.11081200	0.17631100	2.83014700
H	-1.81238300	0.36933600	3.86610500
H	-2.82112100	0.95473700	2.54205200
H	-2.64303200	-0.77948500	2.82177400
C	-2.84549800	1.28669300	-1.91364400
H	-2.94364000	0.42032200	-2.57307700
H	-3.68755700	1.95450600	-2.12535000
H	-1.92272900	1.80078000	-2.18320300
C	-4.16755000	0.13602000	-0.09666800
H	-4.22019900	-0.18714900	0.94495000
H	-5.02582800	0.79026600	-0.28298600
H	-4.29654800	-0.74900400	-0.72525000
C	2.21463900	0.12315800	-0.35258300
C	2.12080500	-1.03461900	-1.15020200
C	3.28875600	0.21057700	0.55572900
C	3.06294400	-2.04898300	-1.05364300
H	1.30627900	-1.11451500	-1.85637000
C	4.22549400	-0.80976600	0.65092800
H	3.35517600	1.06249700	1.21848800
C	4.12281000	-1.94461500	-0.15330200
H	2.97325700	-2.92441800	-1.68696000
H	5.03295100	-0.72677600	1.36961400
H	4.85557600	-2.73893400	-0.07508300

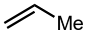

E = -117.952094 Hartree; G (298.15 K) = -117.897770 Hartree

E_SPE = -117.956890 Hartree; G_SPE (298.15 K) = -117.902566 Hartree

0 1

C	-1.27900800	-0.22074100	-0.00000400
H	-1.29683900	-1.30661700	-0.00004200
H	-2.23812200	0.28426400	0.00008400
C	-0.13447800	0.45471300	-0.00001500
H	-0.16643800	1.54284700	0.00001400
C	1.23213300	-0.16252100	-0.00001100
H	1.80575400	0.15212900	-0.87866100
H	1.80579500	0.15222500	0.87865300
H	1.17797100	-1.25355900	0.00012700

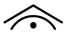

E = -117.303381 Hartree; G (298.15 K) = -117.262674 Hartree

E_SPE = -117.308401 Hartree; G_SPE (298.15 K) = -117.267694 Hartree

0 2

C	-1.22638600	-0.19598800	0.00001600
H	-1.29183800	-1.27847000	0.00014800
H	-2.15402100	0.36135700	0.00001500
C	0.00002700	0.44252100	-0.00007500
H	-0.00016200	1.53057400	0.00005500
C	1.22633200	-0.19590100	0.00002800
H	2.15417500	0.36114200	0.00019500
H	1.29200800	-1.27839400	-0.00022000

11. NMR Spectra

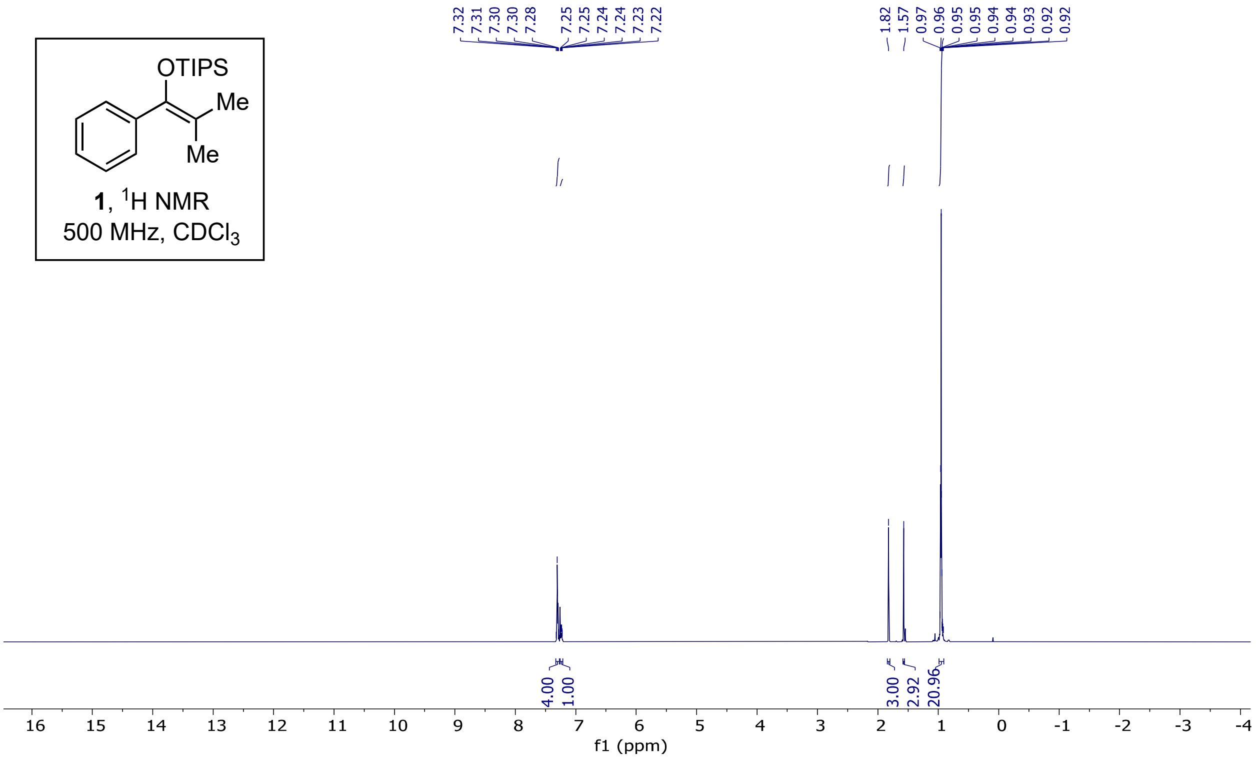

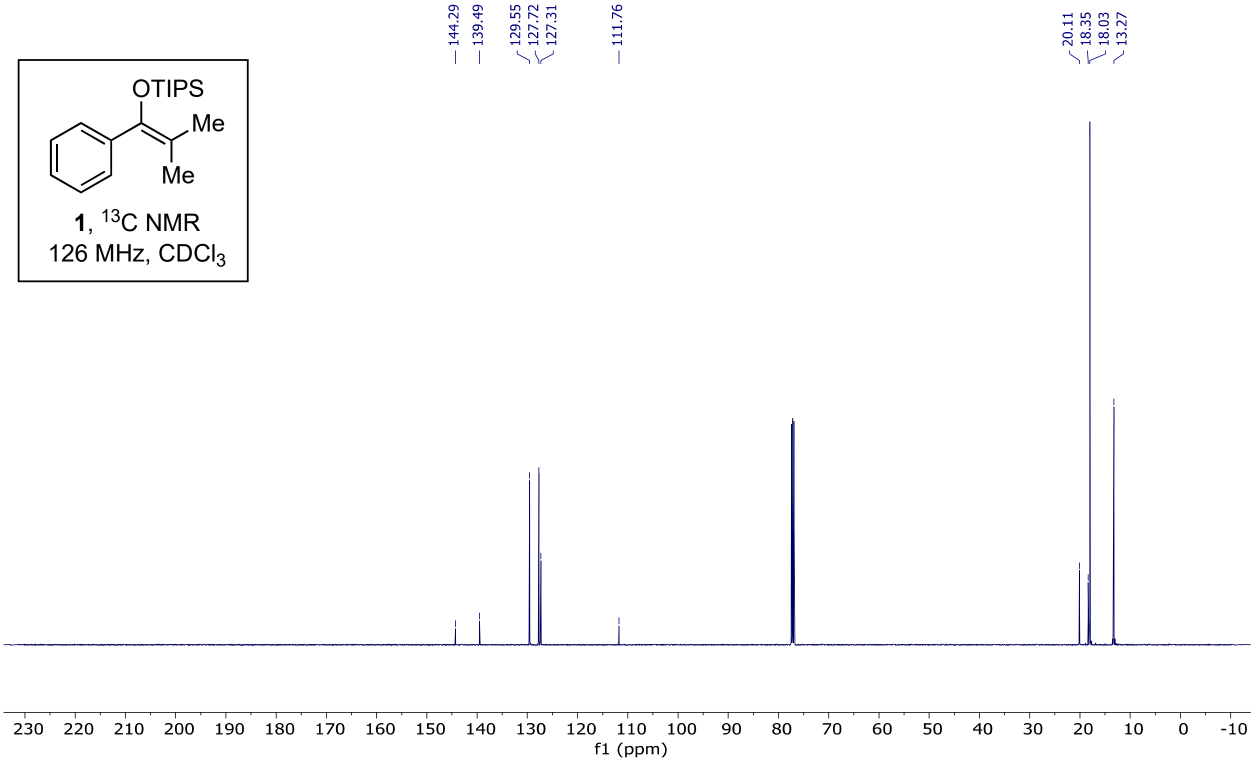

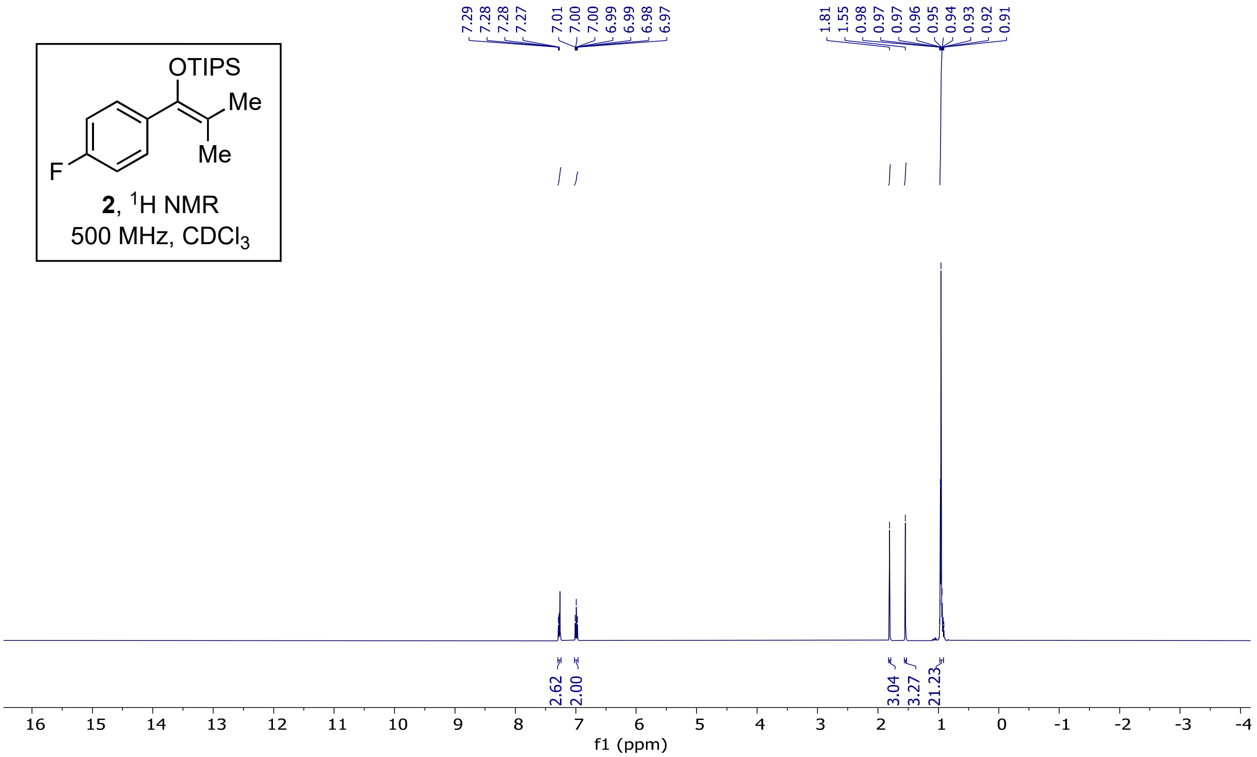
OTIPS
Me
Me
F
2, ^{1}H NMR
500 MHz, $CDCl_3$
7.29
7.28
7.28
7.27
7.01
7.00
7.00
6.99
6.99
6.98
6.97
1.81
1.55
0.98
0.97
0.97
0.96
0.95
0.94
0.93
0.92
0.91
2.62
2.00
3.04
3.27
21.23
f1 (ppm)

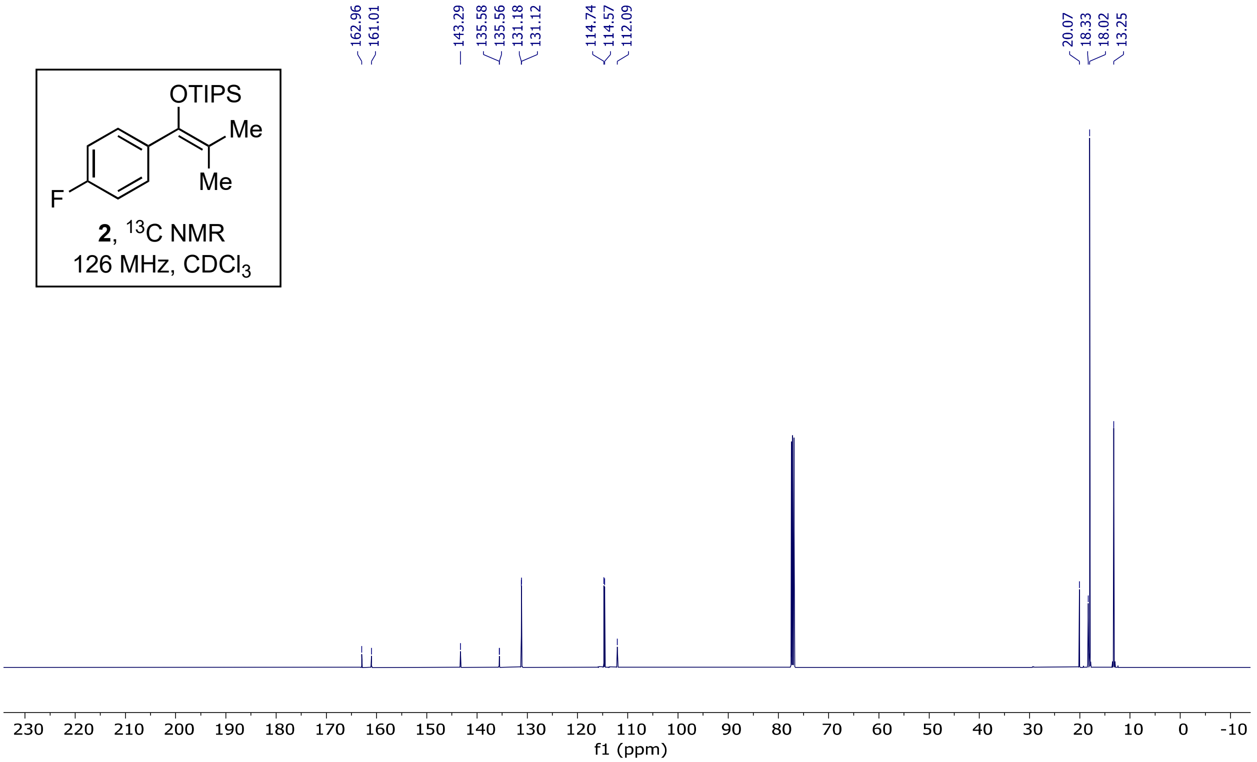
OTIPS
Me
Me
F
2, ^{13}C NMR
126 MHz, $CDCl_3$
162.96
161.01
143.29
135.58
135.56
131.18
131.12
114.74
114.57
112.09
20.07
18.33
18.02
13.25
f1 (ppm)

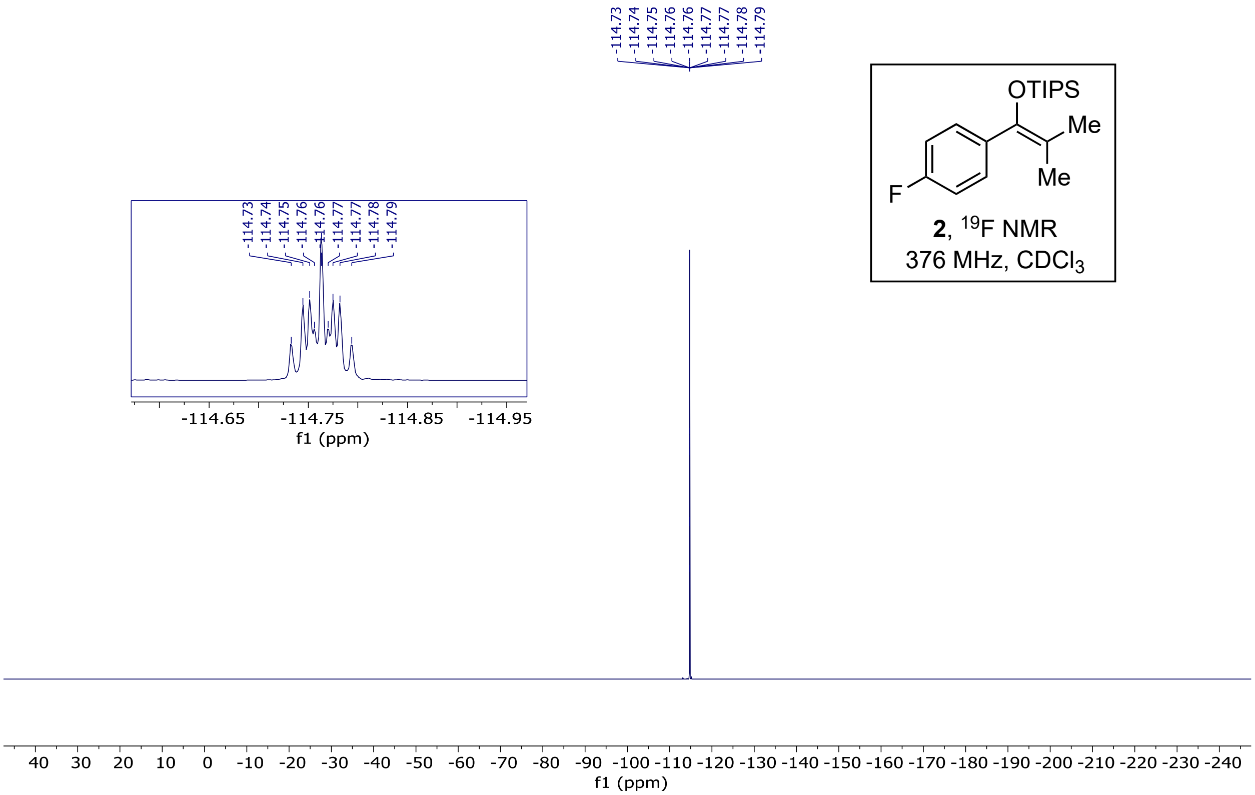
-114.73
-114.74
-114.75
-114.76
-114.76
-114.77
-114.77
-114.78
-114.79
OTIPS
Me
Me
F
2, ^{19}F NMR
376 MHz, $CDCl_3$
-114.65
-114.75
-114.85
-114.95
f1 (ppm)
40 30 20 10 0 -10 -20 -30 -40 -50 -60 -70 -80 -90 -100 -110 -120 -130 -140 -150 -160 -170 -180 -190 -200 -210 -220 -230 -240
f1 (ppm)

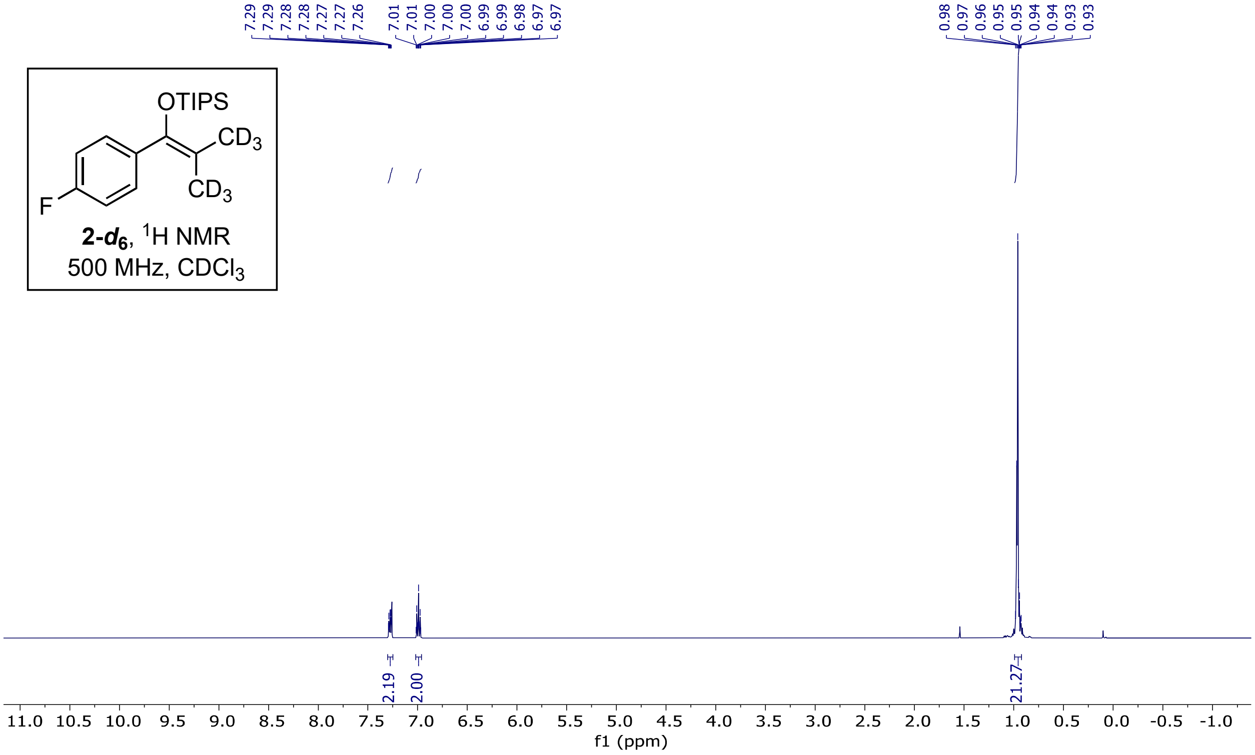
OTIPS
CD3
CD3
F
2-d6, 1H NMR
500 MHz, CDCl3
f1 (ppm)

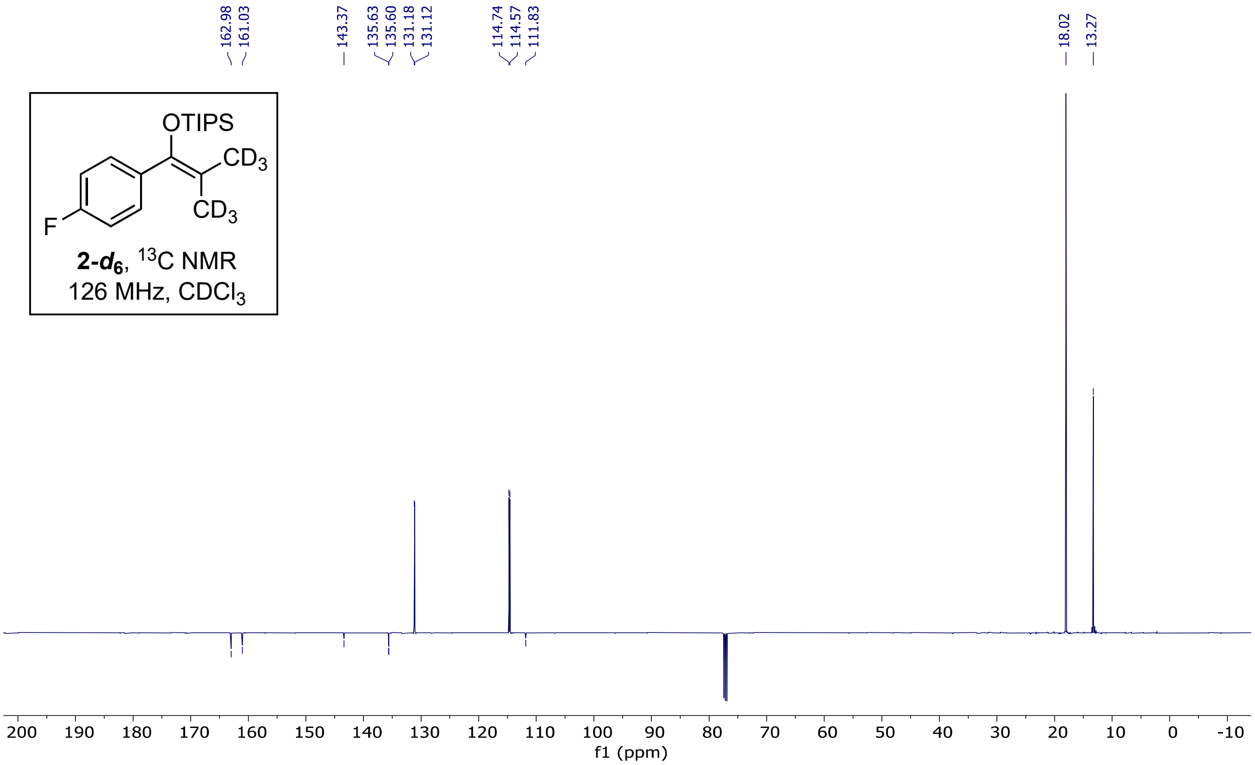
OTIPS
CD3
CD3
F
2-d6, 13C NMR
126 MHz, CDCl3
f1 (ppm)

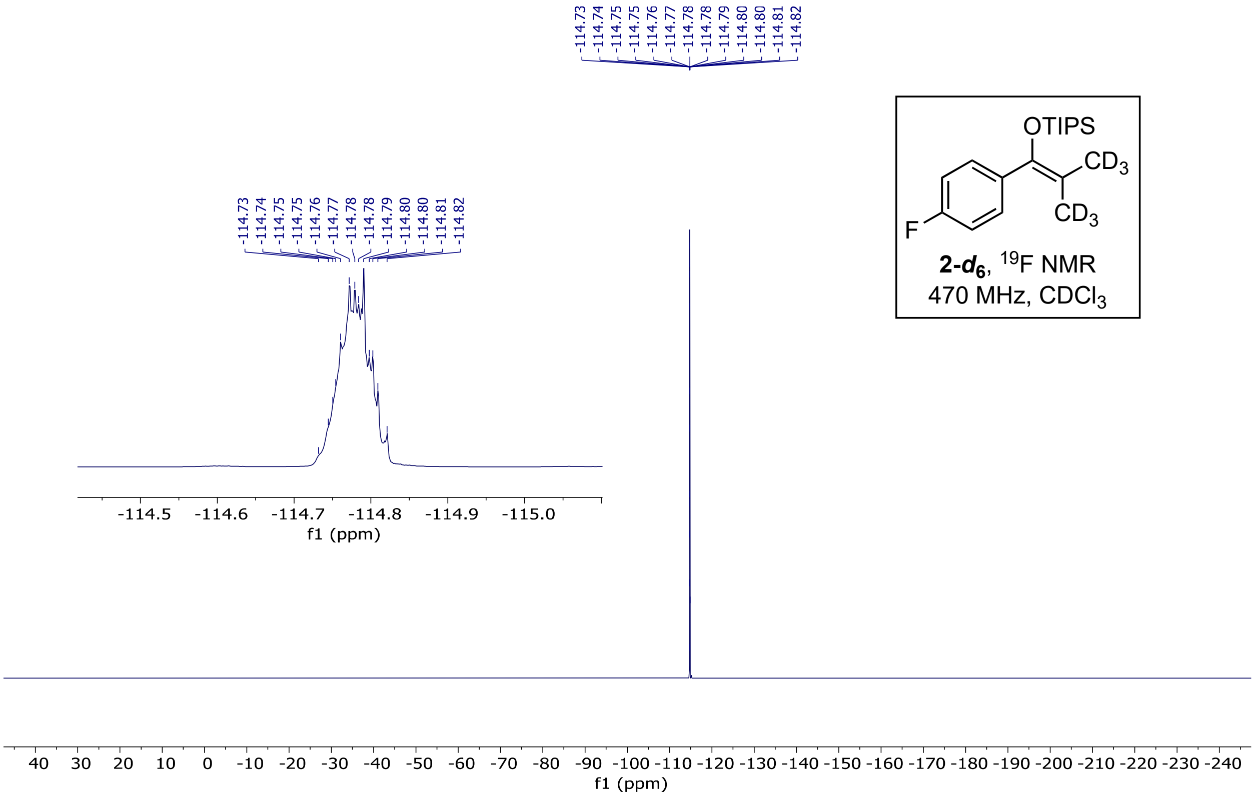

-114.73
-114.74
-114.75
-114.75
-114.76
-114.77
-114.78
-114.78
-114.79
-114.80
-114.80
-114.81
-114.82
-114.5 -114.6 -114.7 -114.8 -114.9 -115.0
f1 (ppm)
OTIPS
CD3
CD3
F
2-d6, 19F NMR
470 MHz, CDCl3
40 30 20 10 0 -10 -20 -30 -40 -50 -60 -70 -80 -90 -100 -110 -120 -130 -140 -150 -160 -170 -180 -190 -200 -210 -220 -230 -240
f1 (ppm)

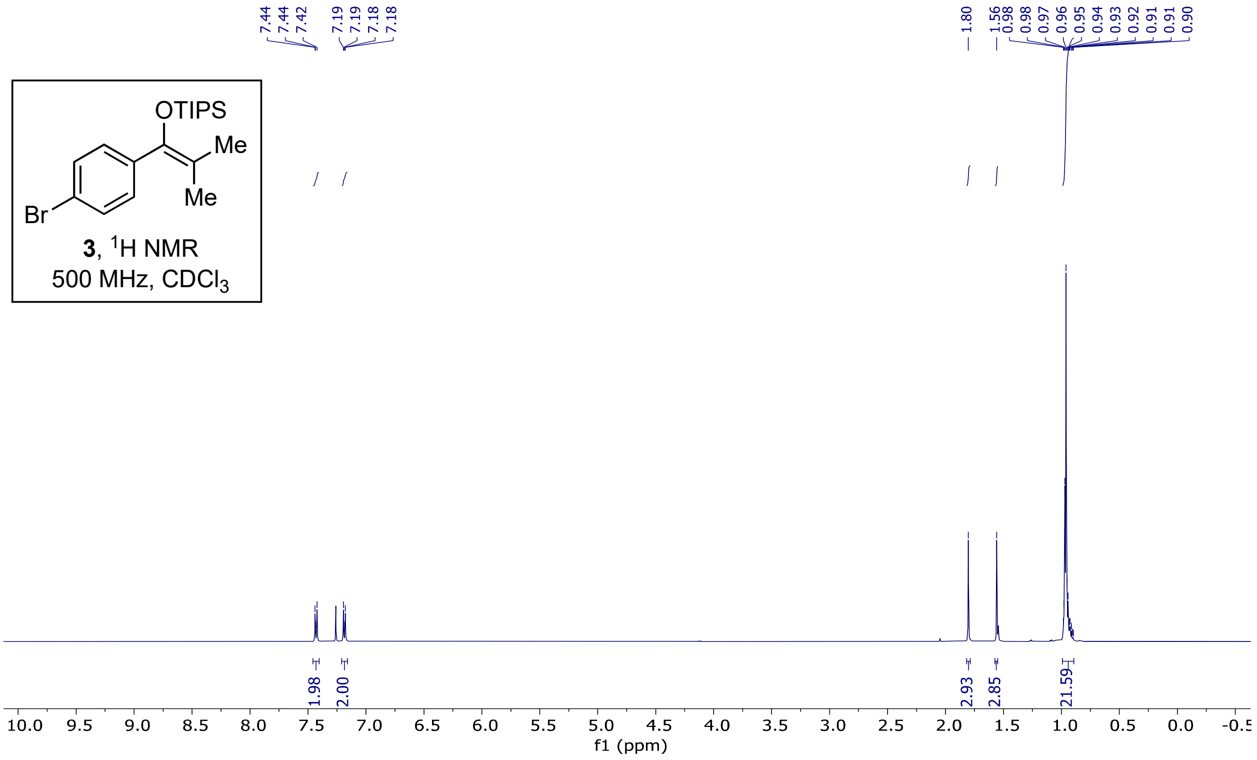

OTIPS
Me
Me
Br
3, ^{1}H NMR
500 MHz, CDCl$_3$
7.44
7.44
7.42
7.19
7.19
7.18
7.18
1.80
1.56
0.98
0.98
0.97
0.96
0.95
0.94
0.93
0.92
0.91
0.91
0.90
1.98
2.00
2.93
2.85
21.59
f1 (ppm)

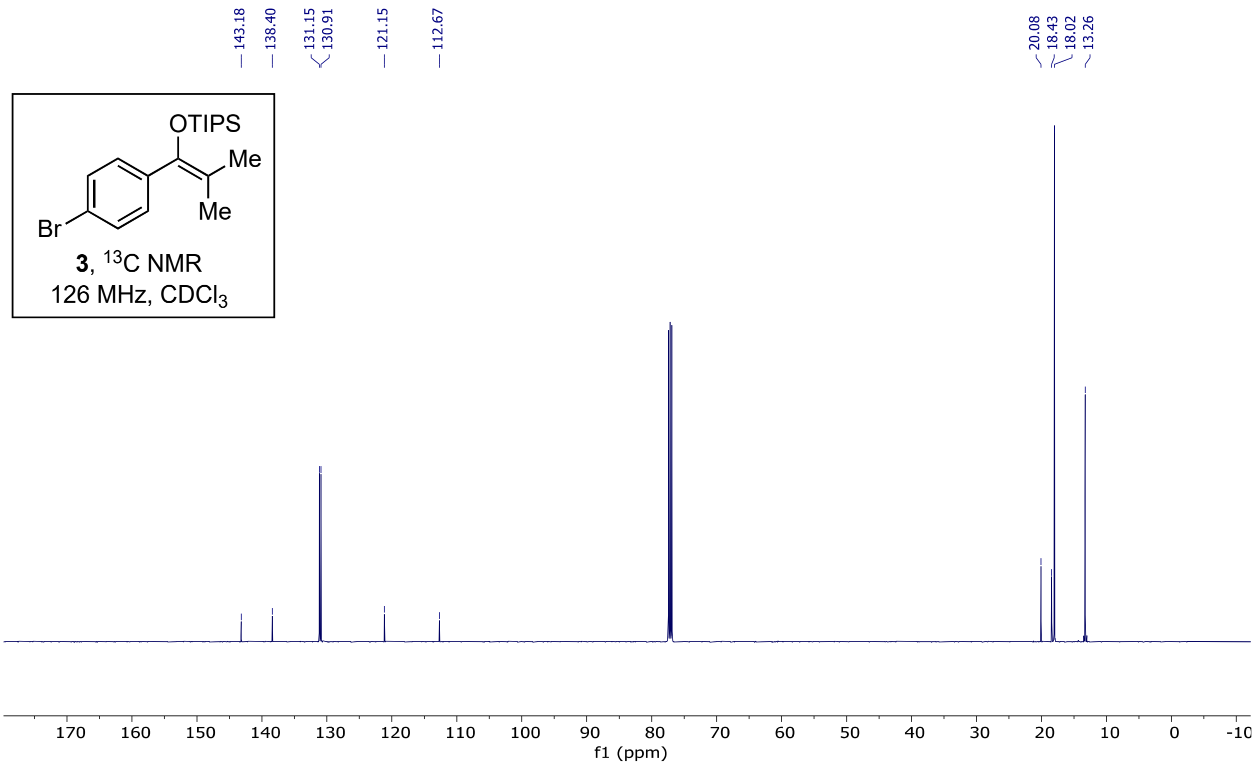

OTIPS
Me
Me
Br
3, ^{13}C NMR
126 MHz, CDCl$_3$
143.18
138.40
131.15
130.91
121.15
112.67
20.08
18.43
18.02
13.26
f1 (ppm)

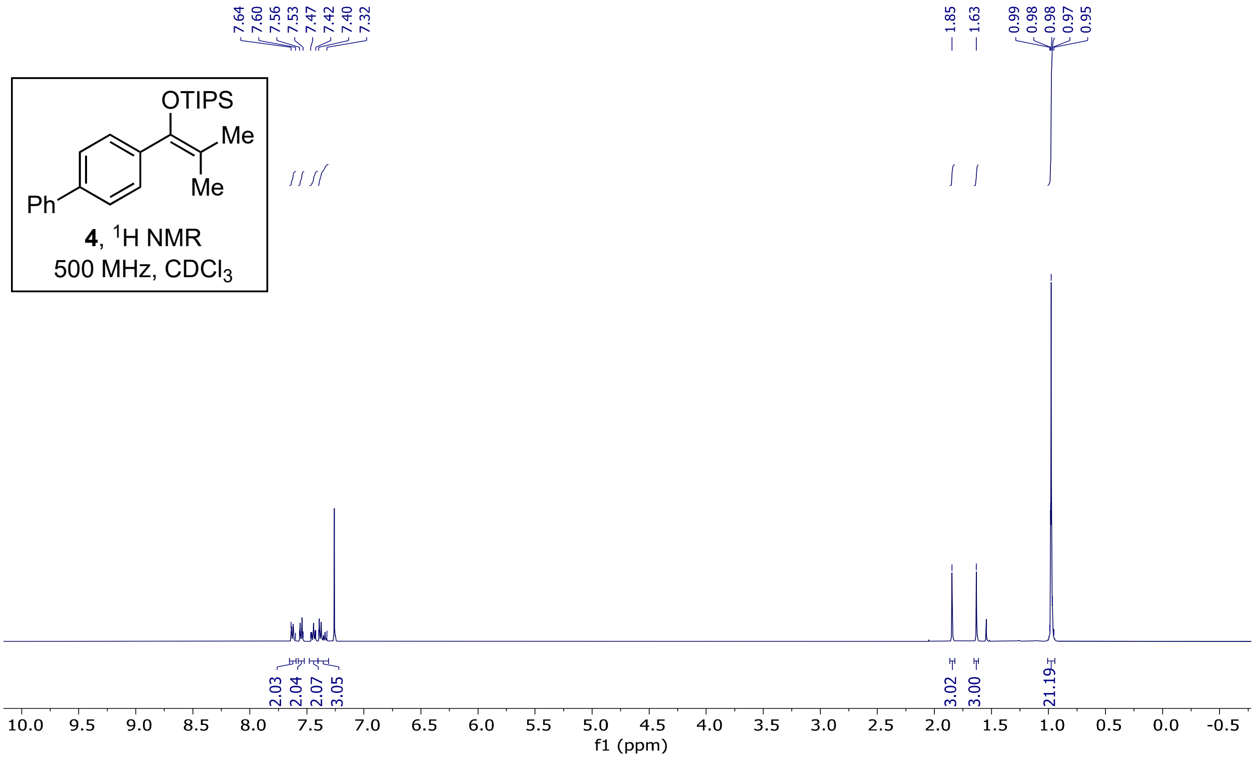
7.64
7.60
7.56
7.53
7.47
7.42
7.40
7.32
1.85
1.63
0.99
0.98
0.98
0.97
0.95
OTIPS
Me
Me
Ph
4, ¹H NMR
500 MHz, CDCl3
2.03
2.04
2.07
3.05
3.02
3.00
21.19
10.0 9.5 9.0 8.5 8.0 7.5 7.0 6.5 6.0 5.5 5.0 4.5 4.0 3.5 3.0 2.5 2.0 1.5 1.0 0.5 0.0 -0.5
f1 (ppm)

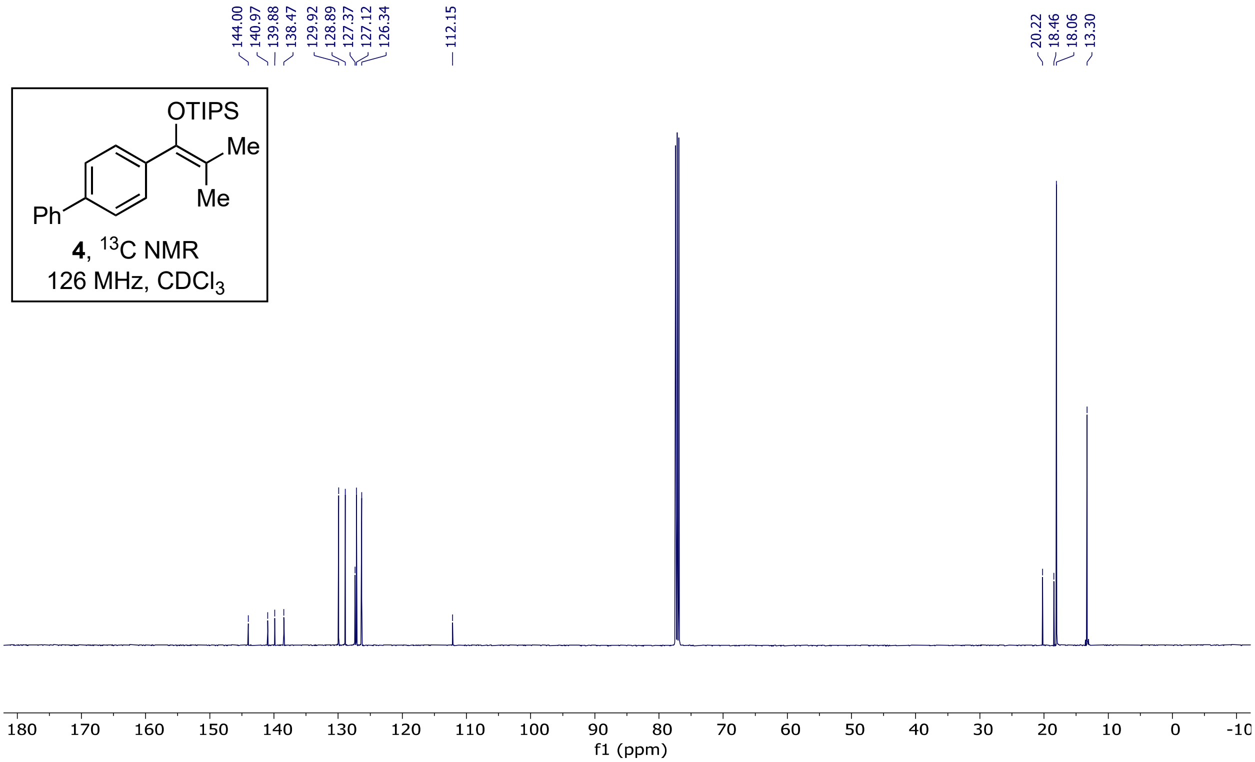
144.00
140.97
139.88
138.47
129.92
128.89
127.37
127.12
126.34
112.15
20.22
18.46
18.06
13.30
OTIPS
Me
Me
Ph
4, ¹³C NMR
126 MHz, CDCl3
180 170 160 150 140 130 120 110 100 90 80 70 60 50 40 30 20 10 0 -10
f1 (ppm)

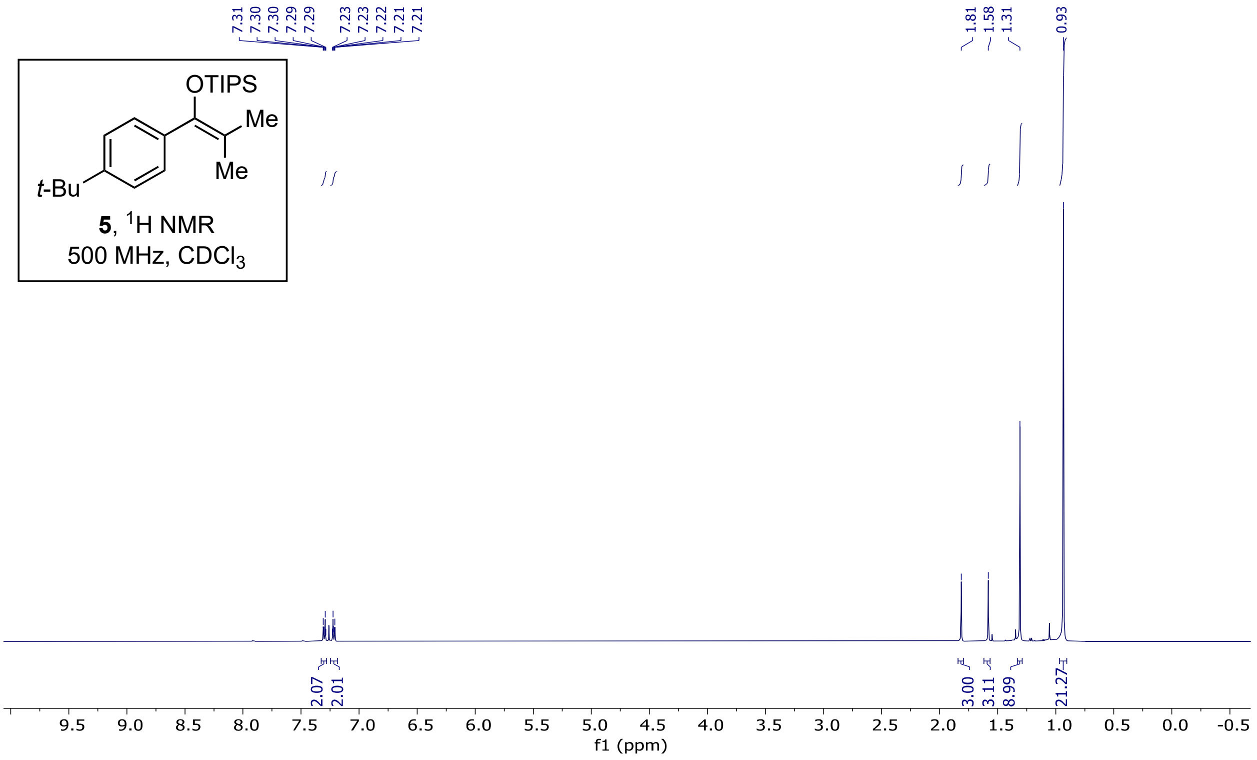
OTIPS
Me
Me
t-Bu
5, 1H NMR
500 MHz, CDCl3
7.31
7.30
7.30
7.29
7.29
7.23
7.23
7.22
7.21
7.21
1.81
1.58
1.31
0.93
2.07
2.01
3.00
3.11
8.99
21.27
9.5
9.0
8.5
8.0
7.5
7.0
6.5
6.0
5.5
5.0
4.5
4.0
3.5
3.0
2.5
2.0
1.5
1.0
0.5
0.0
-0.5
f1 (ppm)

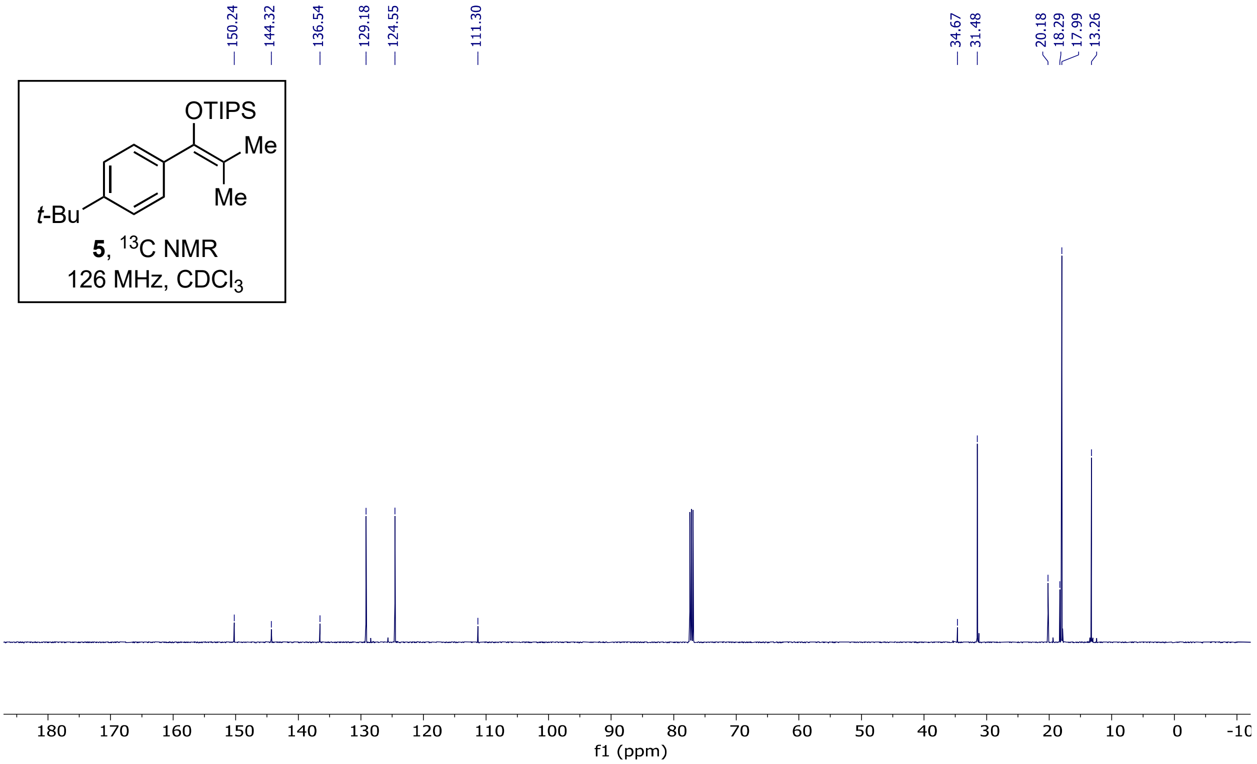
OTIPS
Me
Me
t-Bu
5, 13C NMR
126 MHz, CDCl3
150.24
144.32
136.54
129.18
124.55
111.30
34.67
31.48
20.18
18.29
17.99
13.26
180
170
160
150
140
130
120
110
100
90
80
70
60
50
40
30
20
10
0
-10
f1 (ppm)

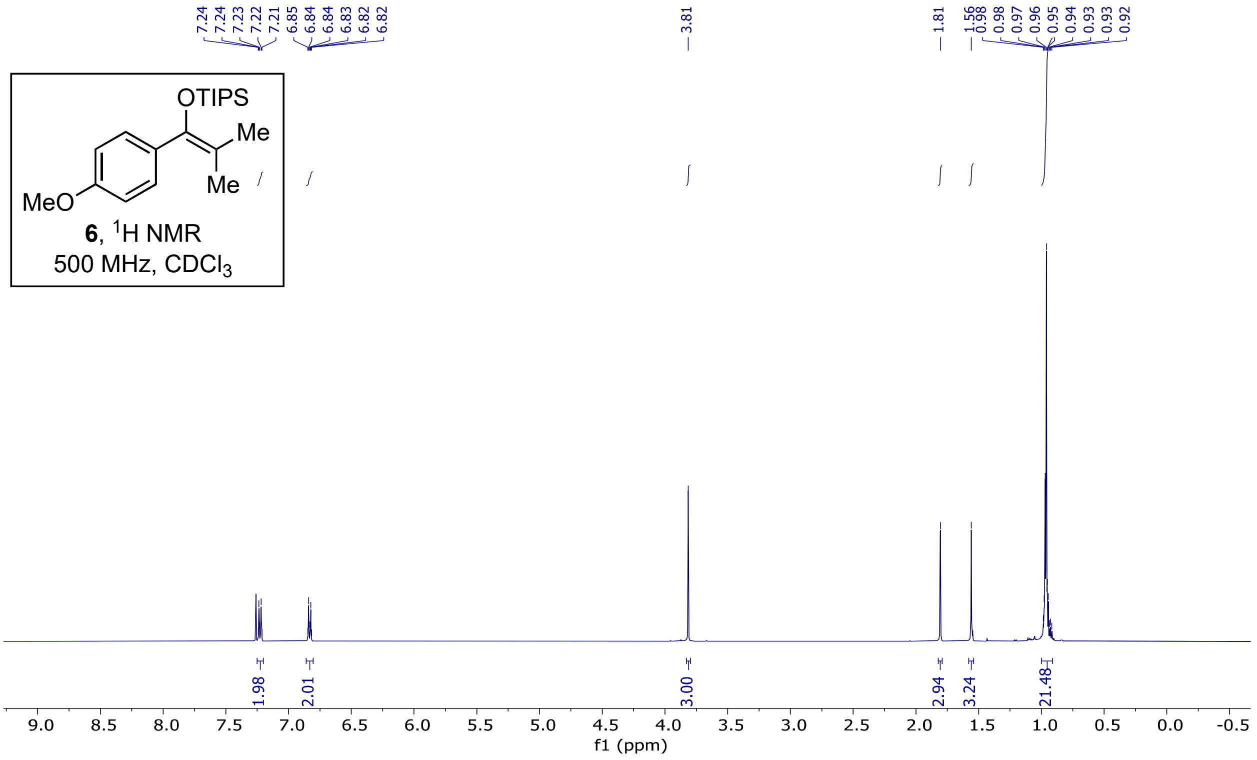
OTIPS
Me
Me
MeO
6, ^{1}H NMR
500 MHz, $CDCl_3$
f1 (ppm)

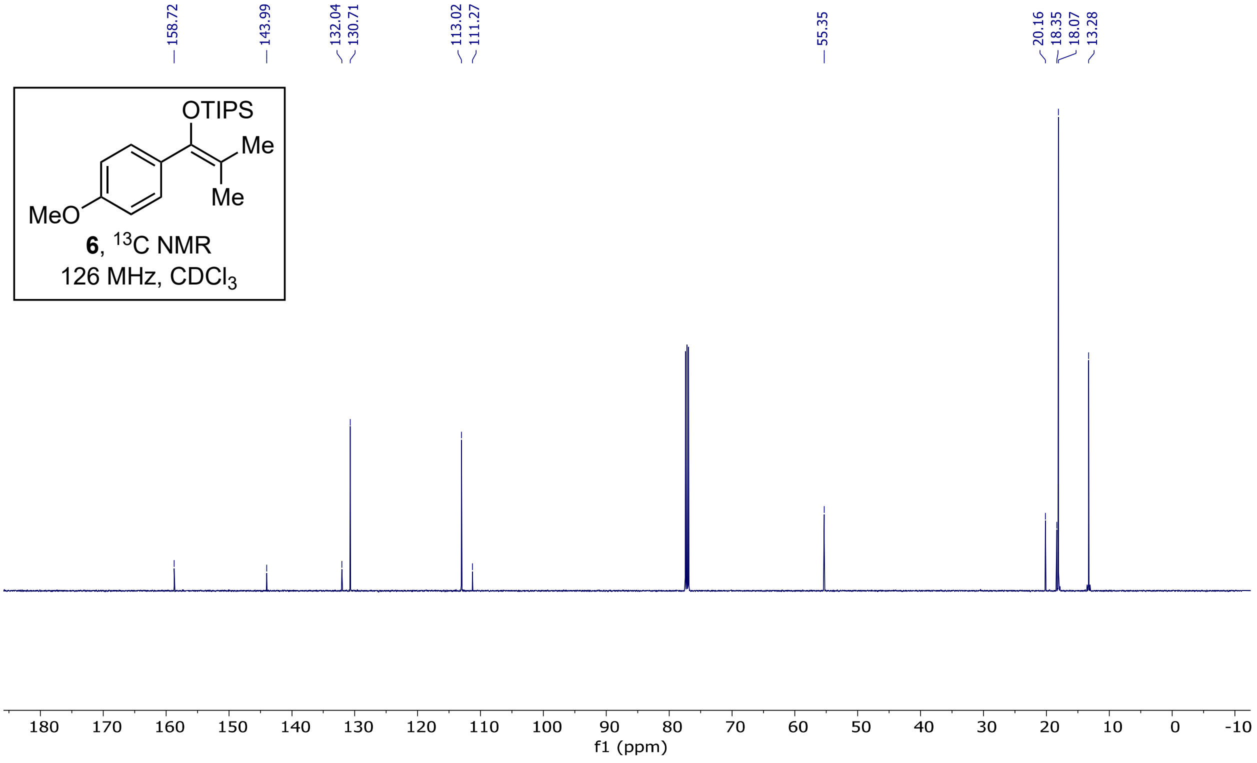
OTIPS
Me
Me
MeO
6, ^{13}C NMR
126 MHz, $CDCl_3$
f1 (ppm)

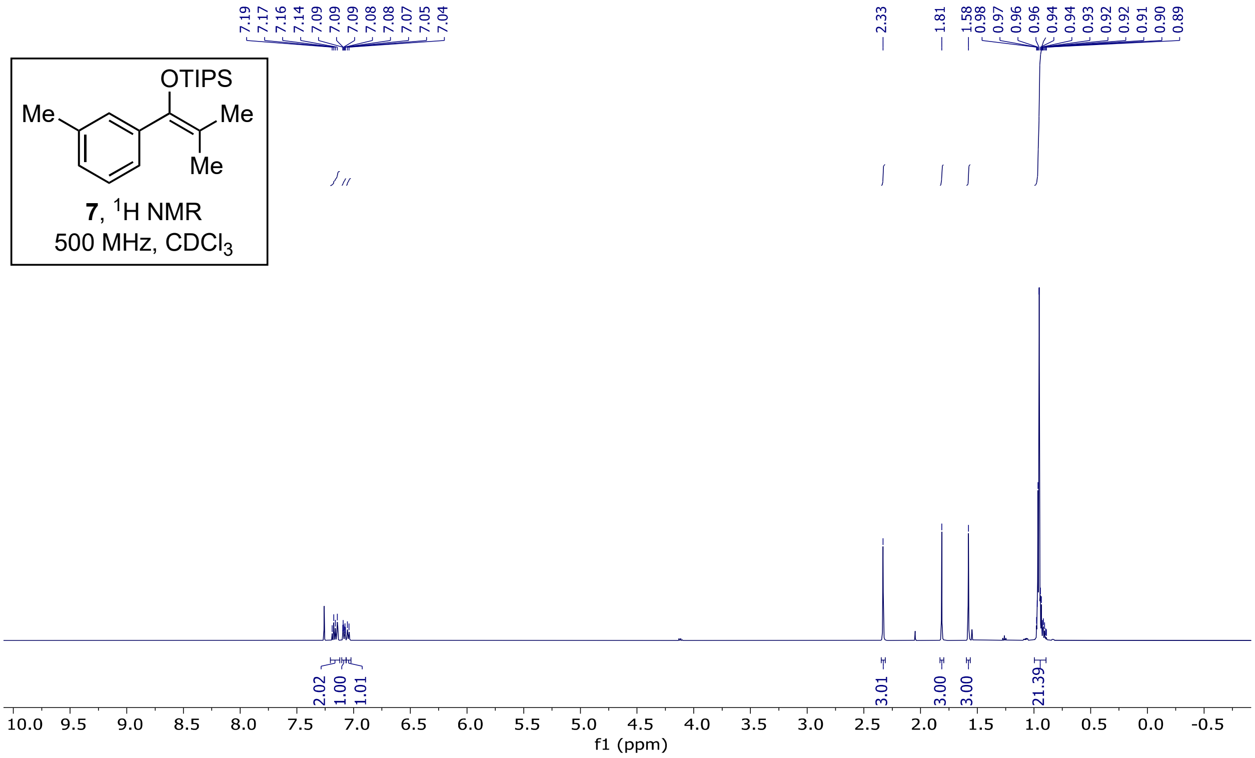
OTIPS
Me
Me
Me
7, ^{1}H NMR
500 MHz, $CDCl_3$
7.19
7.17
7.16
7.14
7.09
7.09
7.09
7.08
7.08
7.07
7.05
7.04
2.33
1.81
1.58
0.98
0.97
0.96
0.96
0.94
0.94
0.93
0.92
0.92
0.91
0.90
0.89
2.02
1.00
1.01
3.01
3.00
3.00
21.39
10.0 9.5 9.0 8.5 8.0 7.5 7.0 6.5 6.0 5.5 5.0 4.5 4.0 3.5 3.0 2.5 2.0 1.5 1.0 0.5 0.0 -0.5
f1 (ppm)

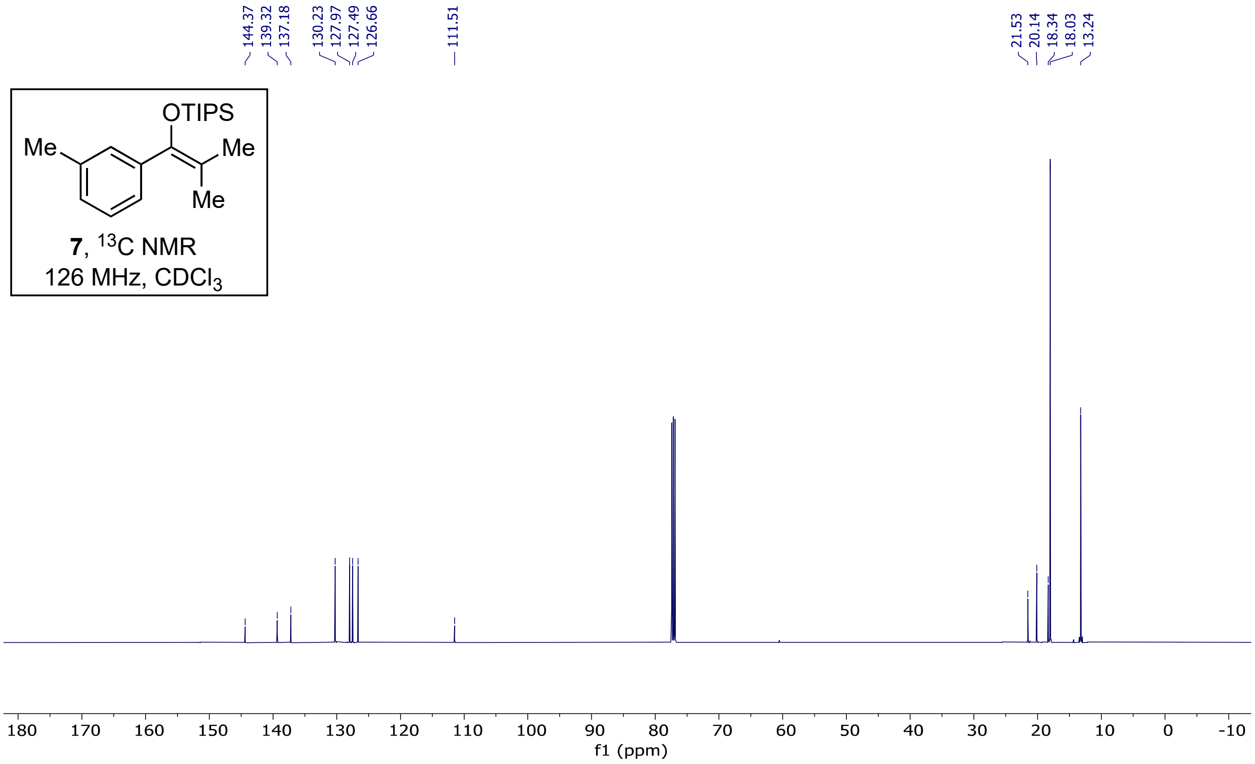
OTIPS
Me
Me
Me
7, ^{13}C NMR
126 MHz, $CDCl_3$
144.37
139.32
137.18
130.23
127.97
127.49
126.66
111.51
21.53
20.14
18.34
18.03
13.24
180 170 160 150 140 130 120 110 100 90 80 70 60 50 40 30 20 10 0 -10
f1 (ppm)

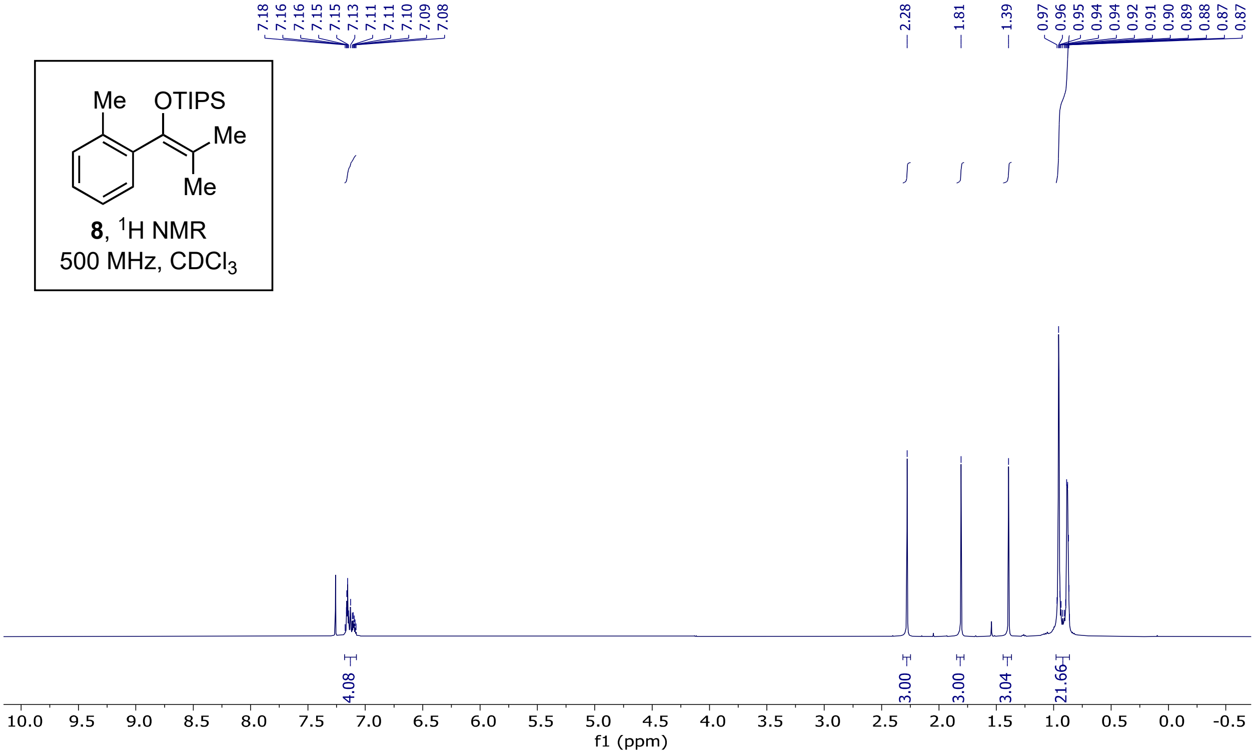

7.18 7.16 7.16 7.15 7.15 7.13 7.11 7.11 7.10 7.09 7.08
2.28
1.81
1.39
0.97 0.96 0.95 0.94 0.94 0.92 0.91 0.90 0.89 0.88 0.87 0.87
Me
OTIPS
Me
Me
8, 1H NMR
500 MHz, CDCl3
4.08
3.00
3.00
3.04
21.66
10.0 9.5 9.0 8.5 8.0 7.5 7.0 6.5 6.0 5.5 5.0 4.5 4.0 3.5 3.0 2.5 2.0 1.5 1.0 0.5 0.0 -0.5
f1 (ppm)

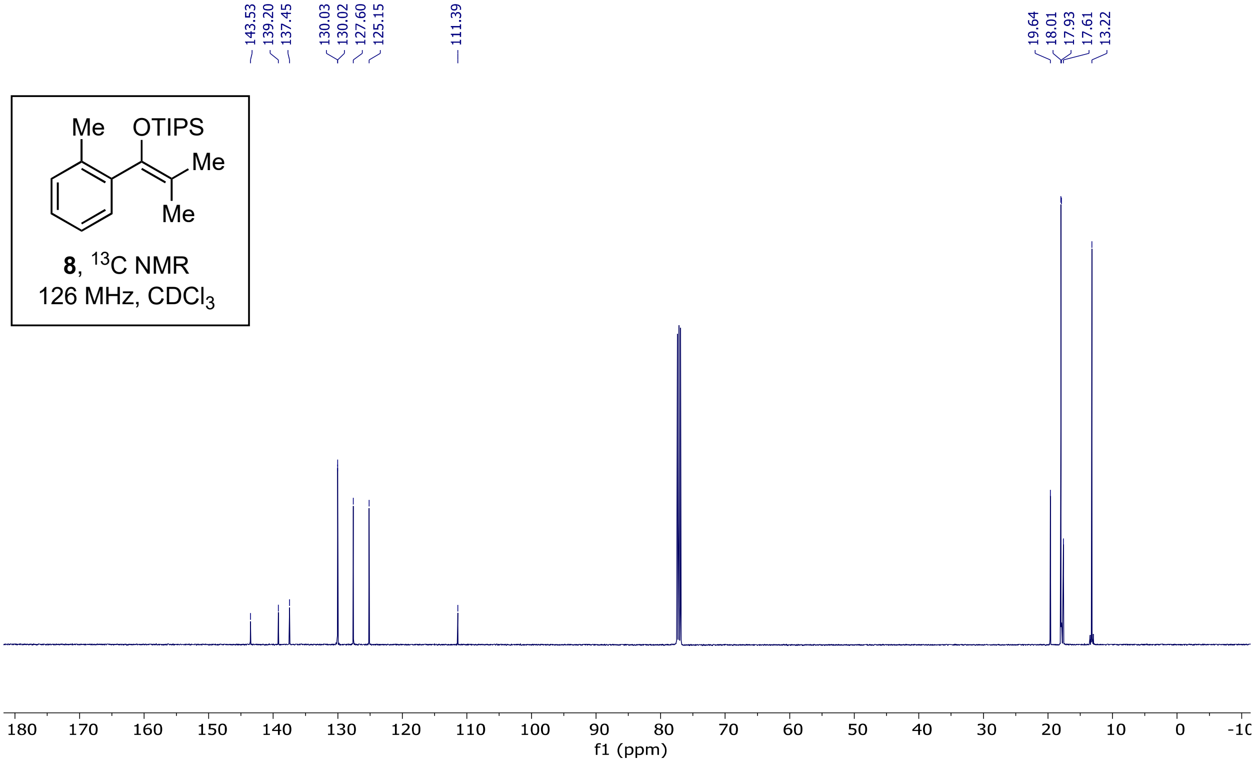

143.53 139.20 137.45
130.03 130.02 127.60 125.15
111.39
19.64 18.01 17.93 17.61 13.22
Me
OTIPS
Me
Me
8, 13C NMR
126 MHz, CDCl3
180 170 160 150 140 130 120 110 100 90 80 70 60 50 40 30 20 10 0 -10
f1 (ppm)

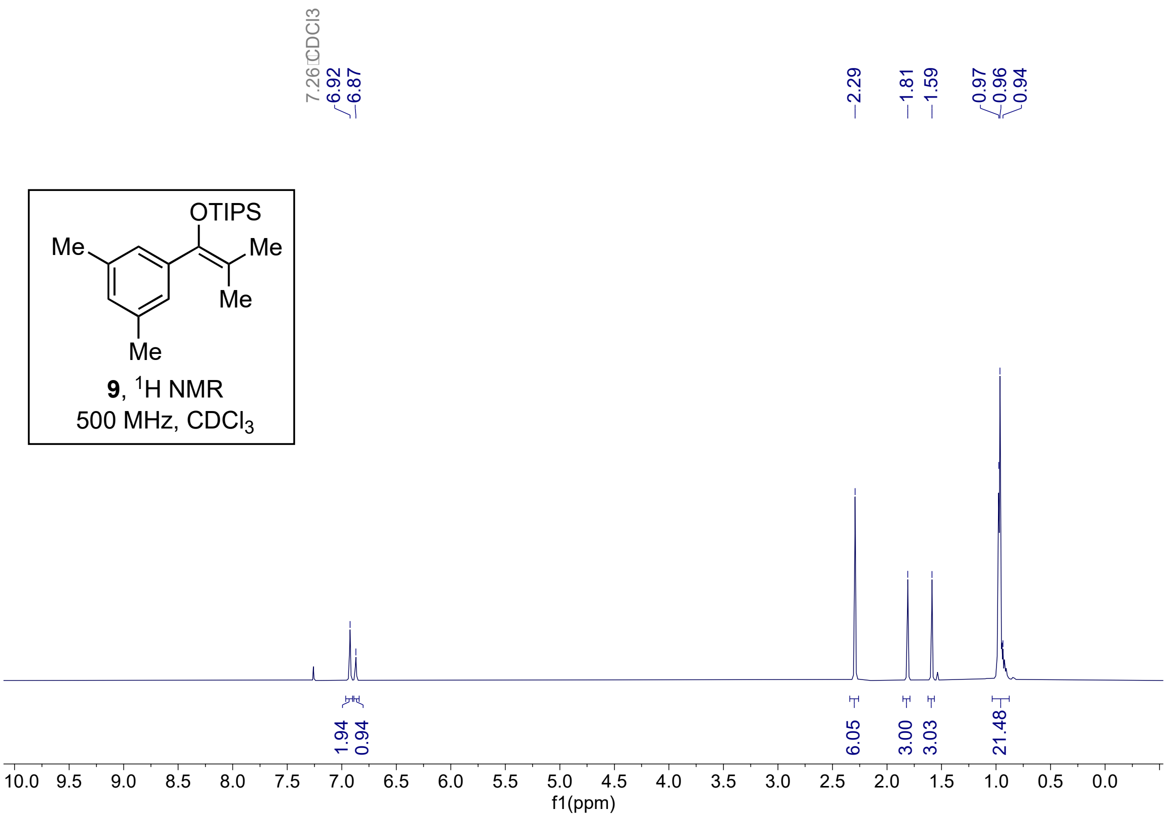
7.26 CDCl3
6.92
6.87
2.29
1.81
1.59
0.97
0.96
0.94
OTIPS
Me
Me
Me
Me
9, 1H NMR
500 MHz, CDCl3
1.94
0.94
6.05
3.00
3.03
21.48
10.0 9.5 9.0 8.5 8.0 7.5 7.0 6.5 6.0 5.5 5.0 4.5 4.0 3.5 3.0 2.5 2.0 1.5 1.0 0.5 0.0
f1(ppm)

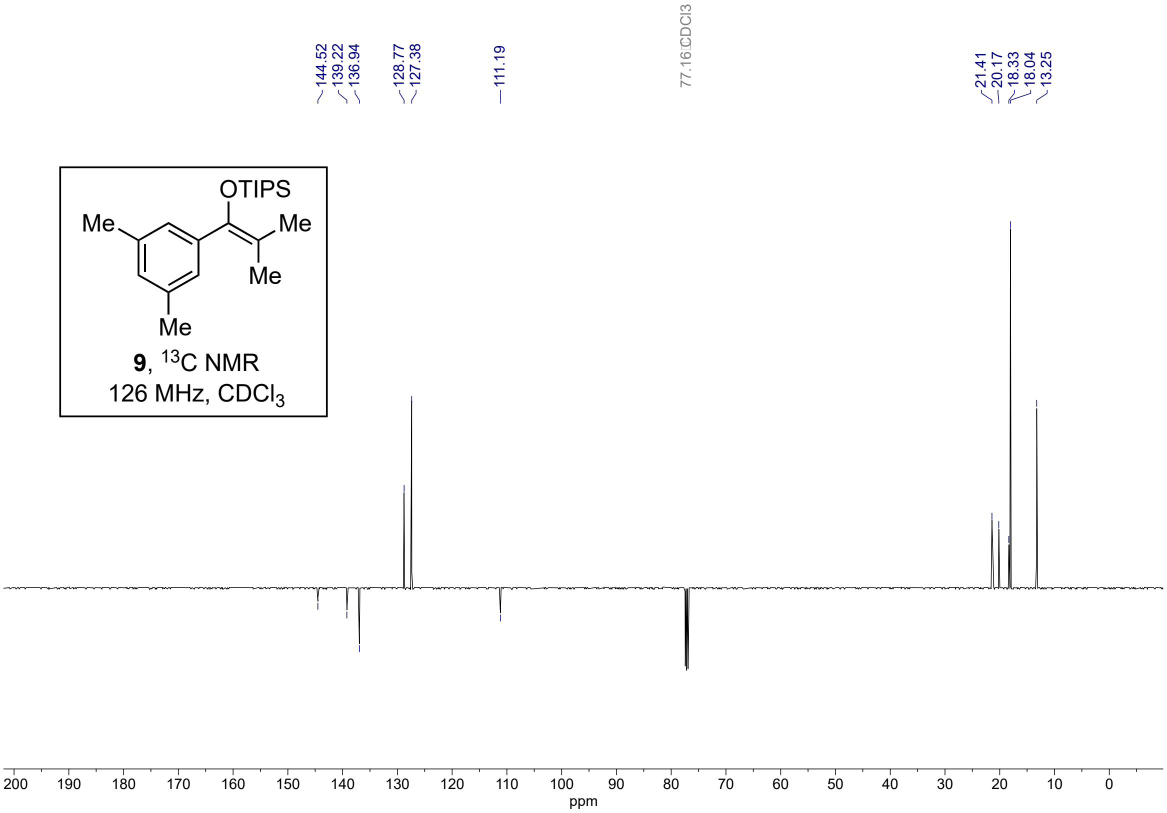
144.52
139.22
136.94
128.77
127.38
111.19
77.16 CDCl3
21.41
20.17
18.33
18.04
13.25
OTIPS
Me
Me
Me
Me
9, 13C NMR
126 MHz, CDCl3
200 190 180 170 160 150 140 130 120 110 100 90 80 70 60 50 40 30 20 10 0
ppm

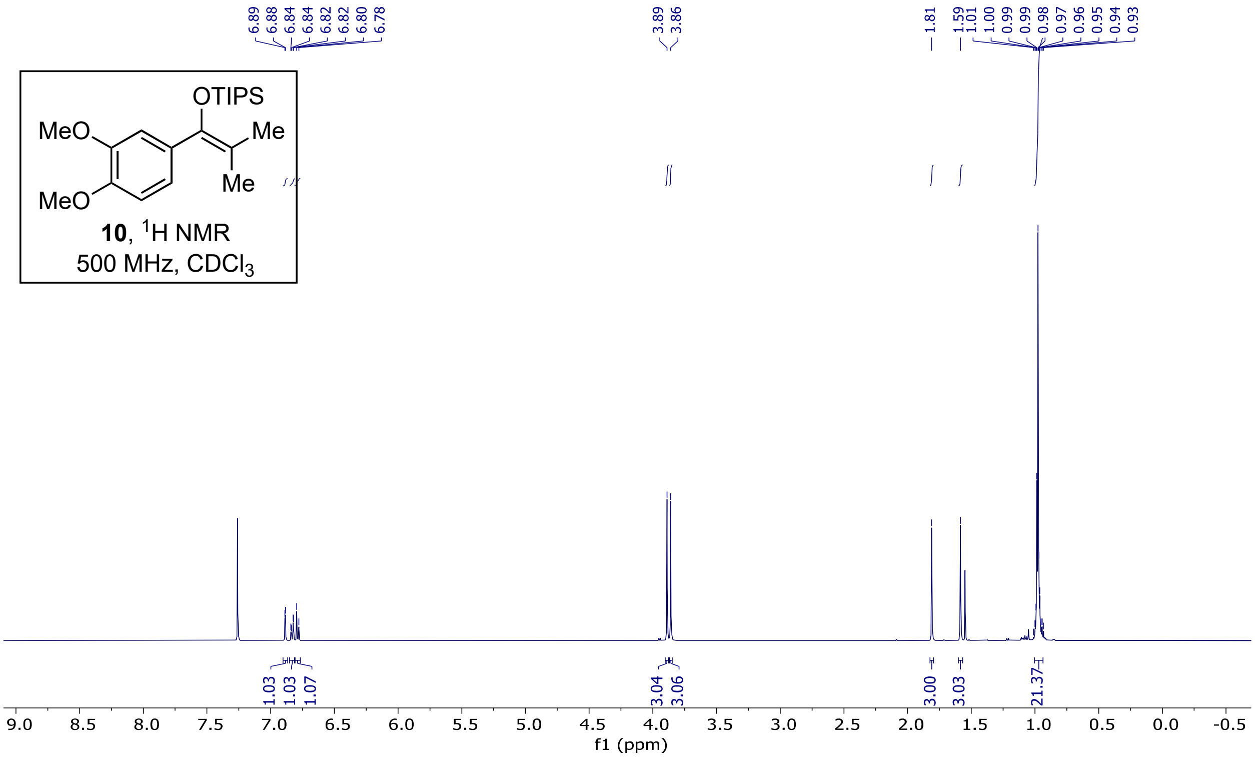
6.89
6.88
6.84
6.84
6.82
6.82
6.80
6.78
3.89
3.86
1.81
1.59
1.01
1.00
0.99
0.99
0.98
0.97
0.96
0.95
0.94
0.93
OTIPS
MeO
Me
Me
MeO
10, 1H NMR
500 MHz, CDCl3
1.03
1.03
1.07
3.04
3.06
3.00
3.03
21.37
f1 (ppm)

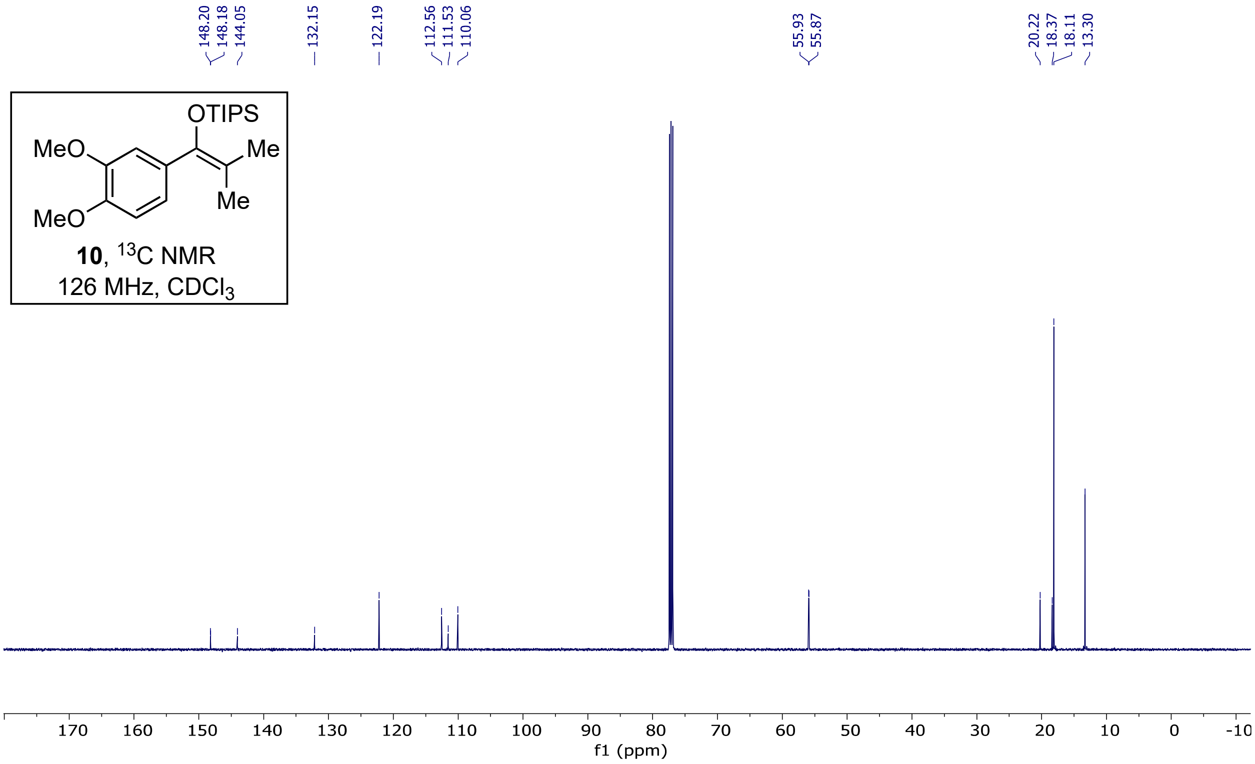
148.20
148.18
144.05
132.15
122.19
112.56
111.53
110.06
55.93
55.87
20.22
18.37
18.11
13.30
OTIPS
MeO
Me
Me
MeO
10, 13C NMR
126 MHz, CDCl3
f1 (ppm)

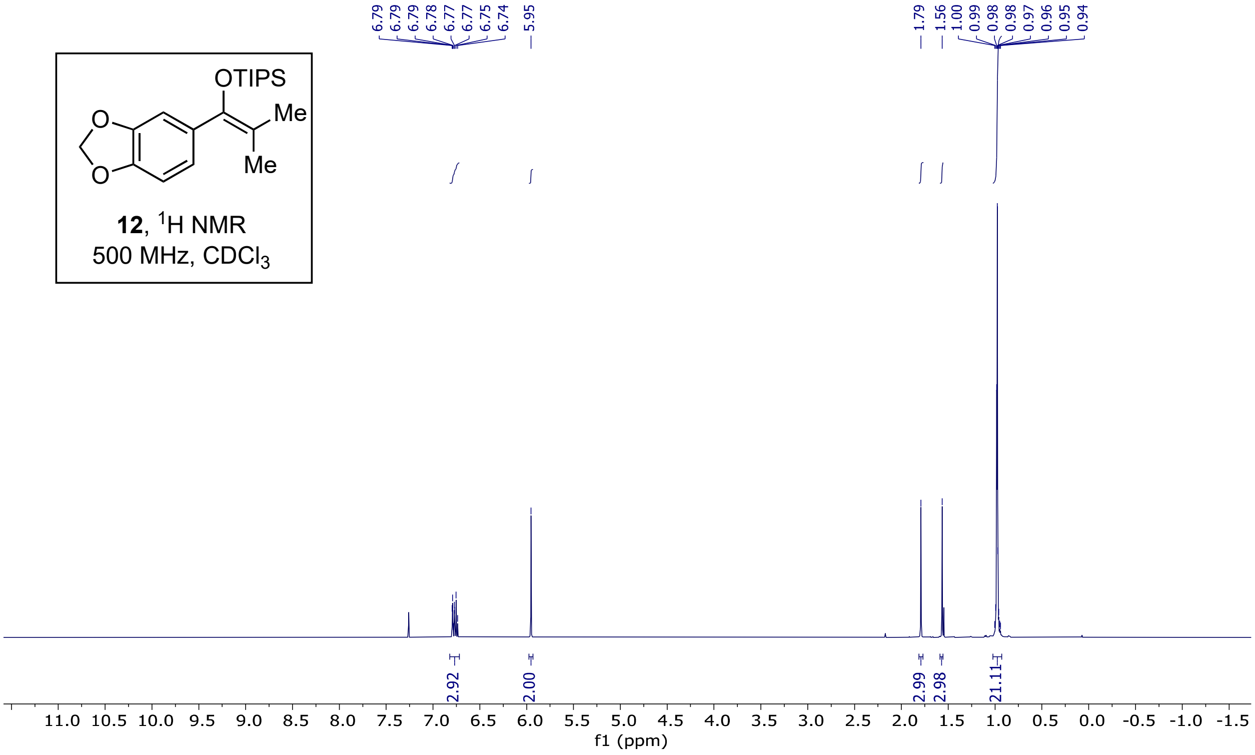
OTIPS
Me
Me
O
O
12, ^{1}H NMR
500 MHz, $CDCl_3$
6.79 6.79 6.79 6.78 6.77 6.77 6.75 6.74 5.95
1.79 1.56 1.00 0.99 0.98 0.98 0.97 0.96 0.95 0.94
2.92 2.00 2.99 2.98 21.11
11.0 10.5 10.0 9.5 9.0 8.5 8.0 7.5 7.0 6.5 6.0 5.5 5.0 4.5 4.0 3.5 3.0 2.5 2.0 1.5 1.0 0.5 0.0 -0.5 -1.0 -1.5
f1 (ppm)

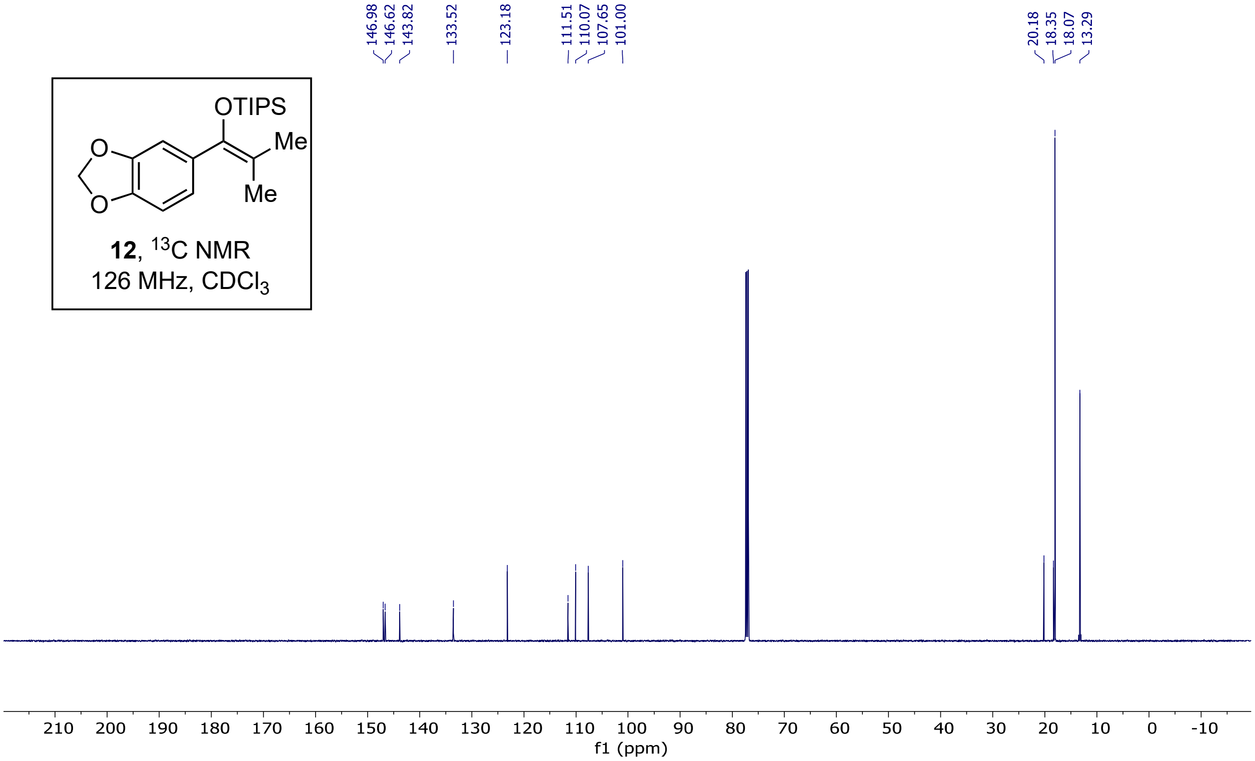
OTIPS
Me
Me
O
O
12, ^{13}C NMR
126 MHz, $CDCl_3$
146.98 146.62 143.82 133.52 123.18 111.51 110.07 107.65 101.00
20.18 18.35 18.07 13.29
210 200 190 180 170 160 150 140 130 120 110 100 90 80 70 60 50 40 30 20 10 0 -10
f1 (ppm)

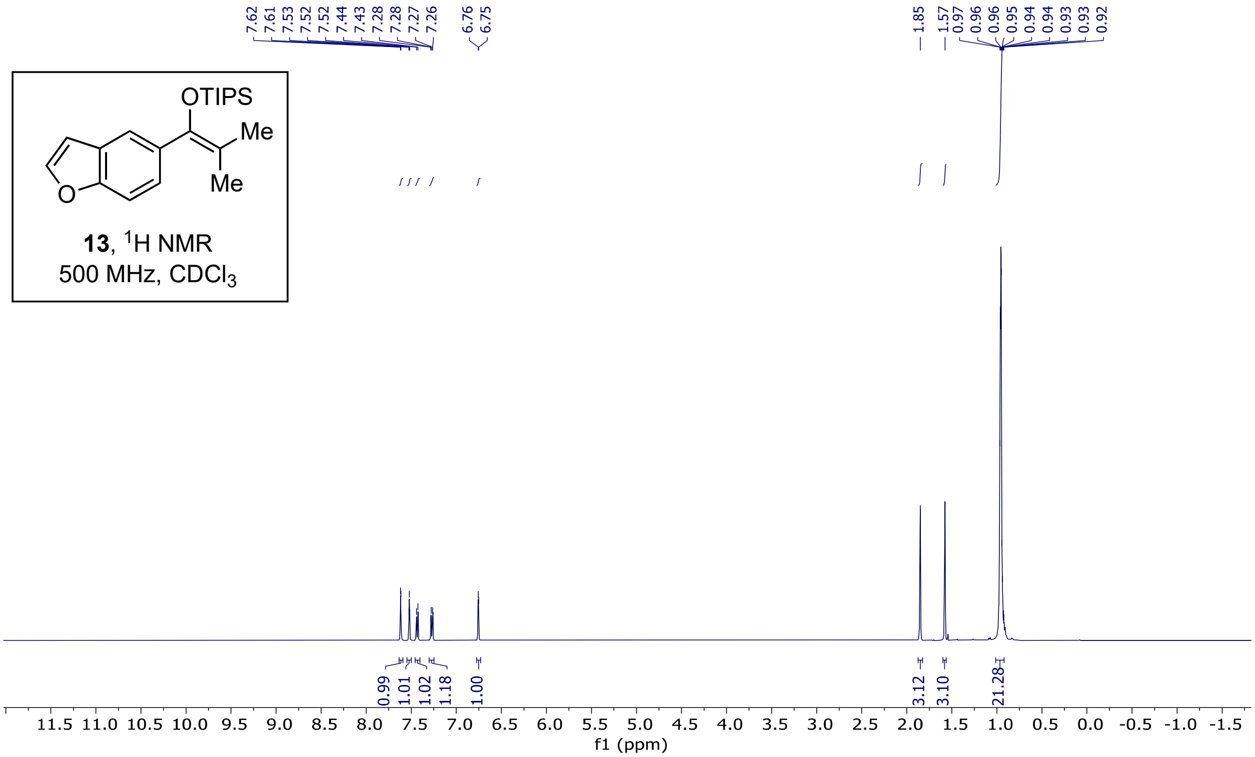
7.62 7.61 7.53 7.52 7.52 7.44 7.43 7.28 7.28 7.27 7.26
6.76 6.75
1.85 1.57 0.97 0.96 0.96 0.96 0.95 0.94 0.94 0.93 0.93 0.92
OTIPS
Me
Me
O
13, ^{1}H NMR
500 MHz, $CDCl_3$
0.99 1.01 1.02 1.18 1.00 3.12 3.10 21.28
f1 (ppm)

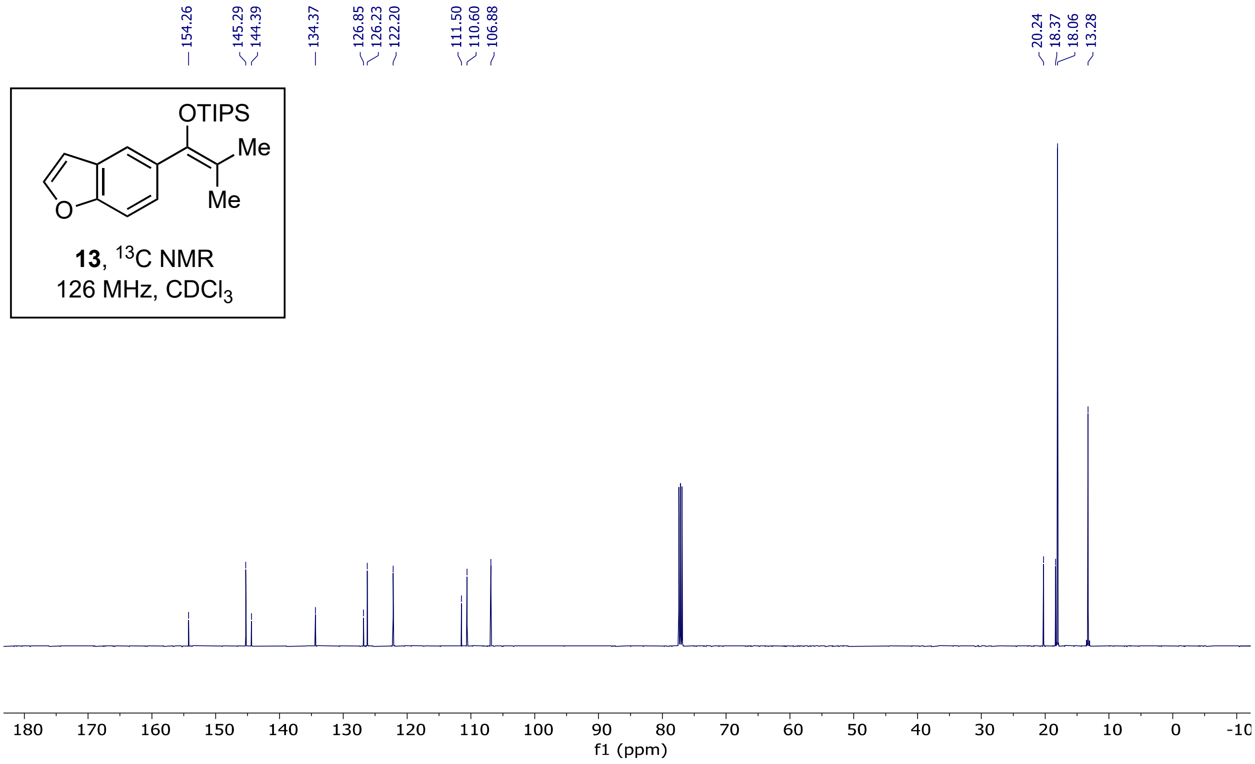
154.26 145.29 144.39 134.37 126.85 126.23 122.20 111.50 110.60 106.88
20.24 18.37 18.06 13.28
OTIPS
Me
Me
O
13, ^{13}C NMR
126 MHz, $CDCl_3$
f1 (ppm)

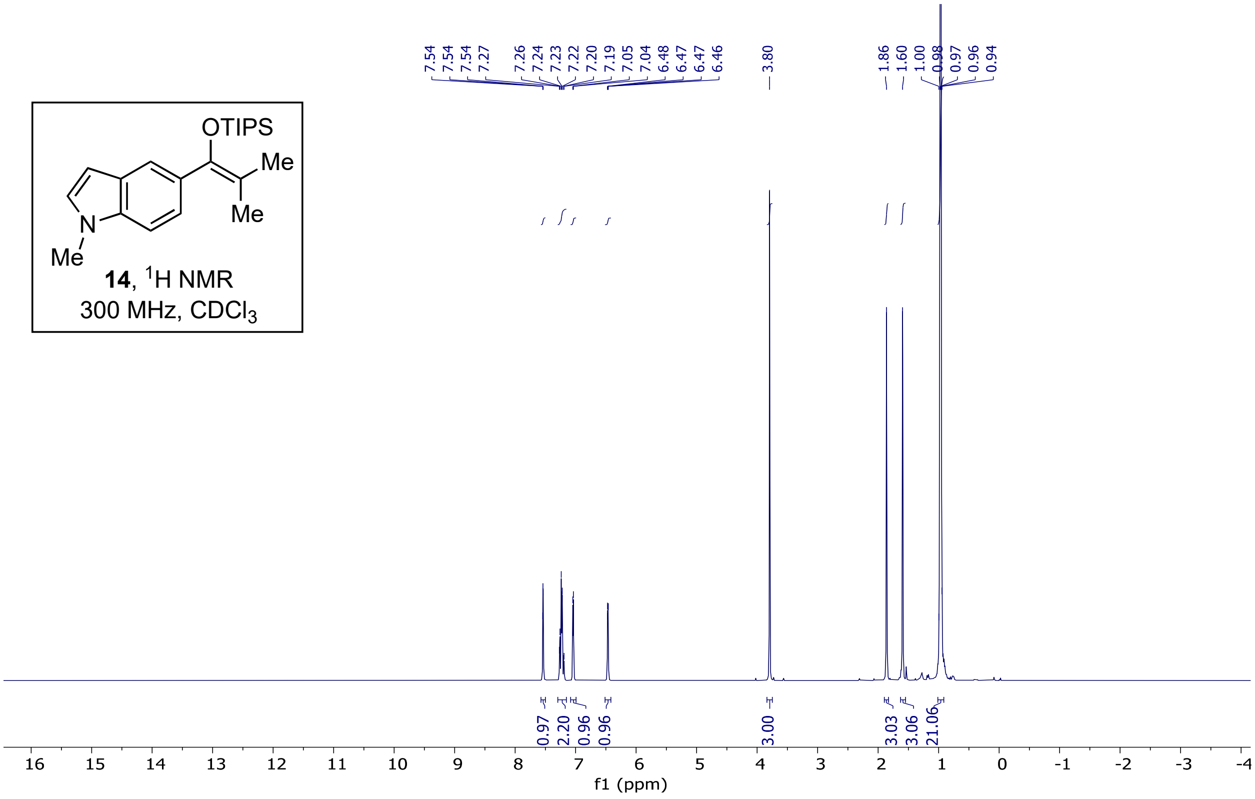
OTIPS
Me
Me
N
Me
14, ¹H NMR
300 MHz, CDCl₃
7.54
7.54
7.54
7.27
7.26
7.24
7.23
7.22
7.20
7.19
7.05
7.04
6.48
6.47
6.47
6.46
3.80
1.86
1.60
1.00
0.98
0.97
0.96
0.94
0.97
2.20
0.96
0.96
3.00
3.03
3.06
21.06
f1 (ppm)

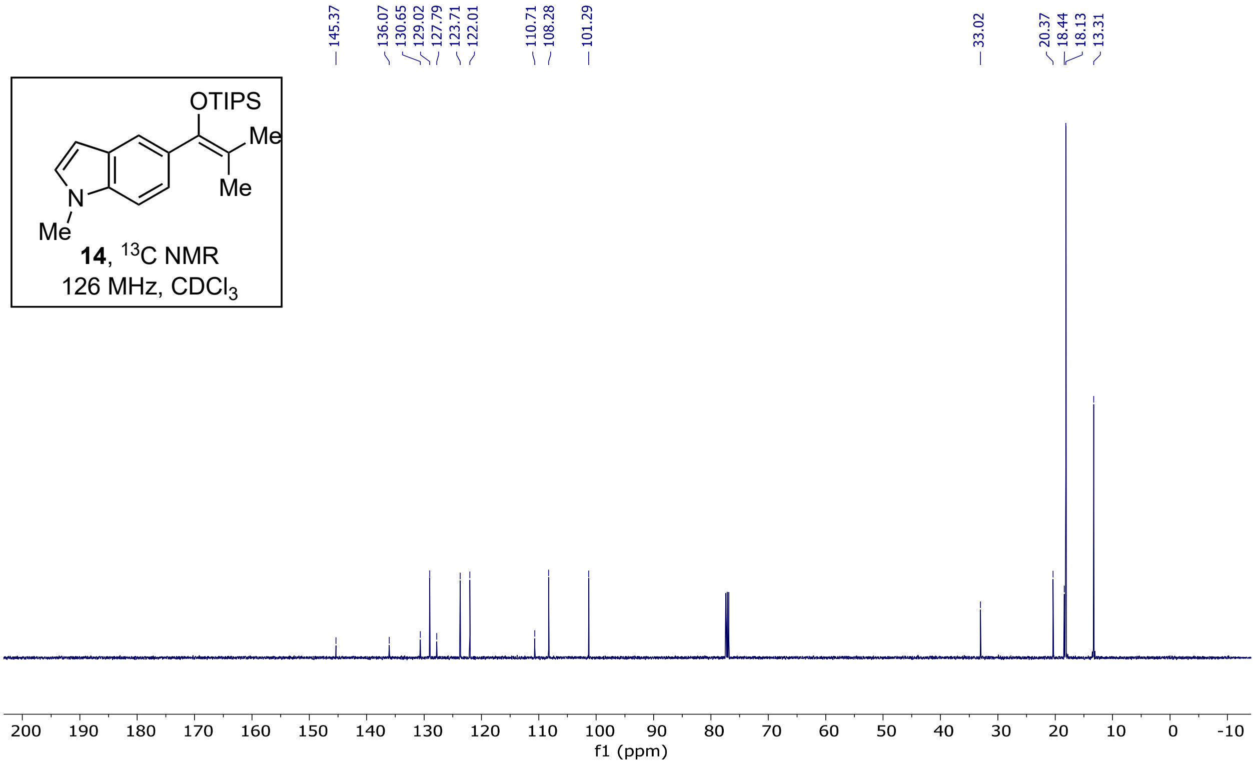
OTIPS
Me
Me
N
Me
14, ¹³C NMR
126 MHz, CDCl₃
145.37
136.07
130.65
129.02
127.79
123.71
122.01
110.71
108.28
101.29
33.02
20.37
18.44
18.13
13.31
f1 (ppm)

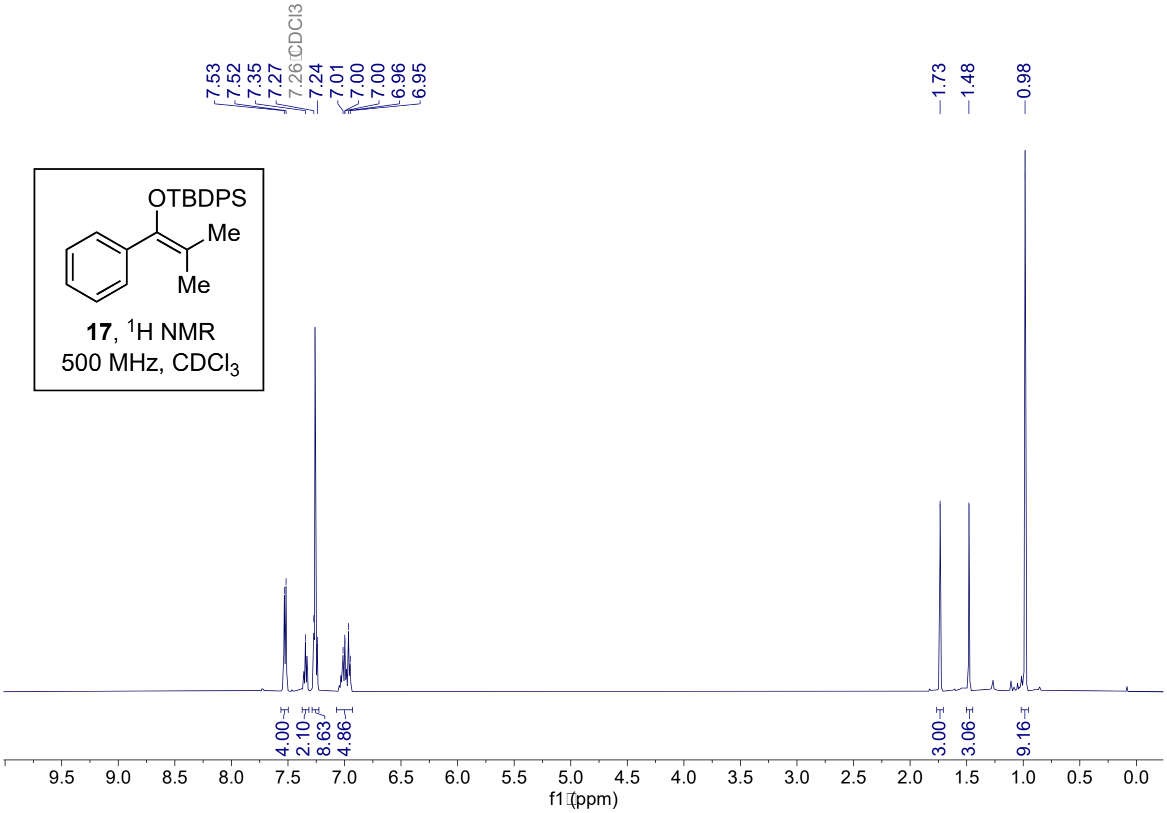
OTBDPS
Me
Me
17, ^{1}H NMR
500 MHz, $CDCl_3$
7.53 7.52 7.35 7.27 7.26 CDCl3 7.24 7.01 7.00 7.00 6.96 6.95
1.73 1.48 0.98
4.00 2.10 8.63 4.86 3.00 3.06 9.16
9.5 9.0 8.5 8.0 7.5 7.0 6.5 6.0 5.5 5.0 4.5 4.0 3.5 3.0 2.5 2.0 1.5 1.0 0.5 0.0
f1 (ppm)

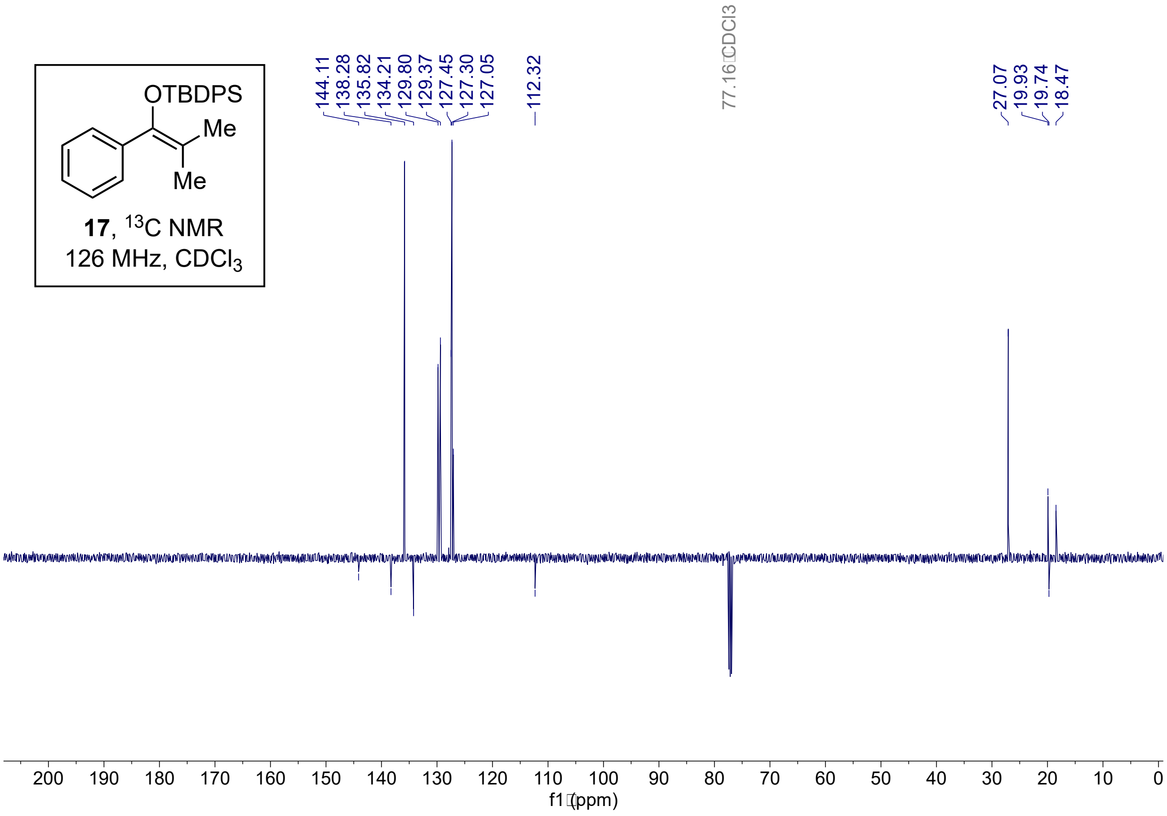
OTBDPS
Me
Me
17, ^{13}C NMR
126 MHz, $CDCl_3$
144.11 138.28 135.82 134.21 129.80 129.37 127.45 127.30 127.05
112.32
77.16 CDCl3
27.07 19.93 19.74 18.47
200 190 180 170 160 150 140 130 120 110 100 90 80 70 60 50 40 30 20 10 0
f1 (ppm)

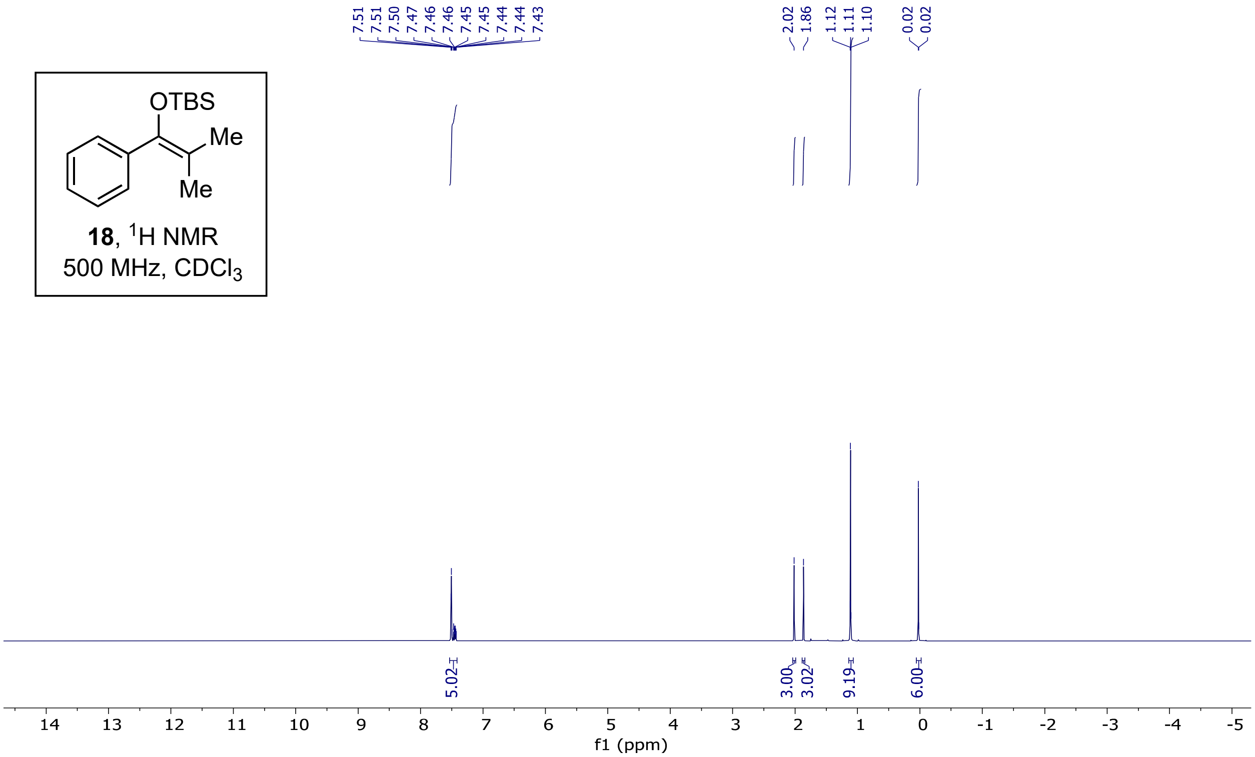
OTBS
Me
Me
18, ^{1}H NMR
500 MHz, $CDCl_3$
7.51 7.51 7.50 7.47 7.46 7.46 7.45 7.45 7.44 7.44 7.43
2.02 1.86 1.12 1.11 1.10 0.02 0.02
5.02 3.00 3.02 9.19 6.00
f1 (ppm)

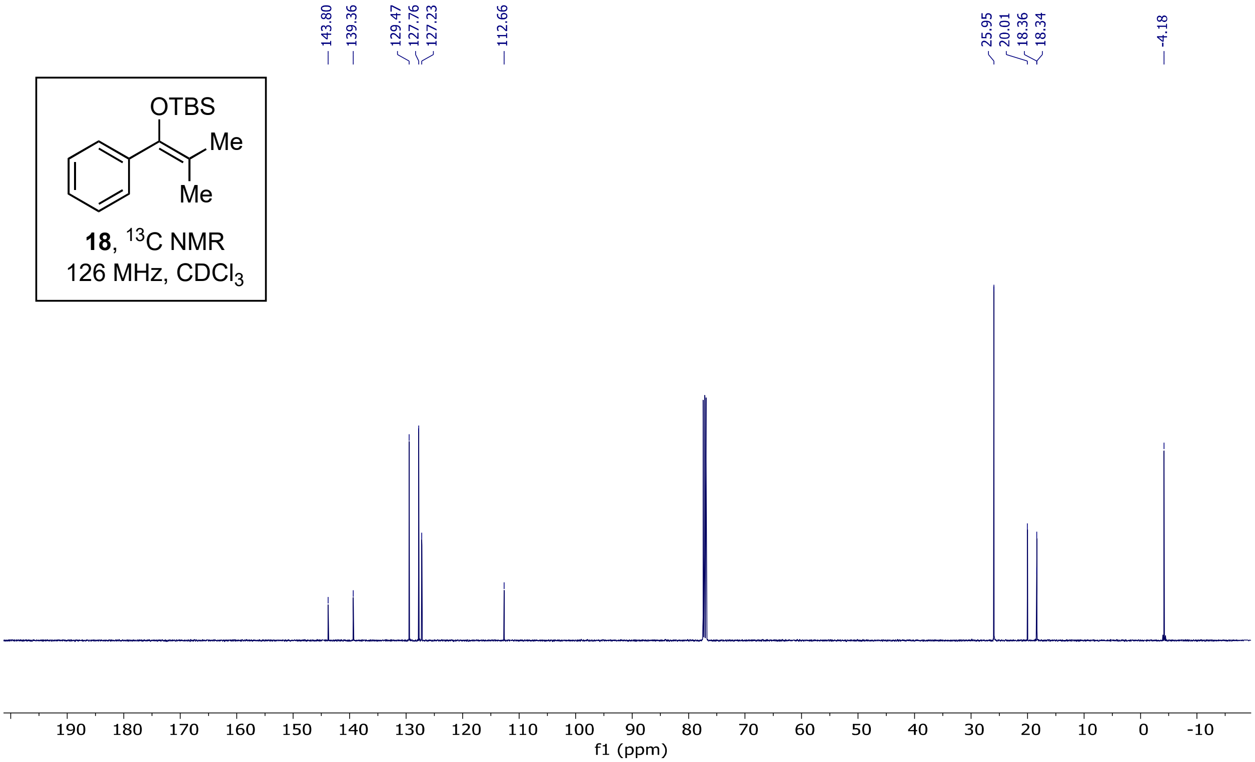
OTBS
Me
Me
18, ^{13}C NMR
126 MHz, $CDCl_3$
143.80 139.36 129.47 127.76 127.23 112.66 25.95 20.01 18.36 18.34 -4.18
f1 (ppm)

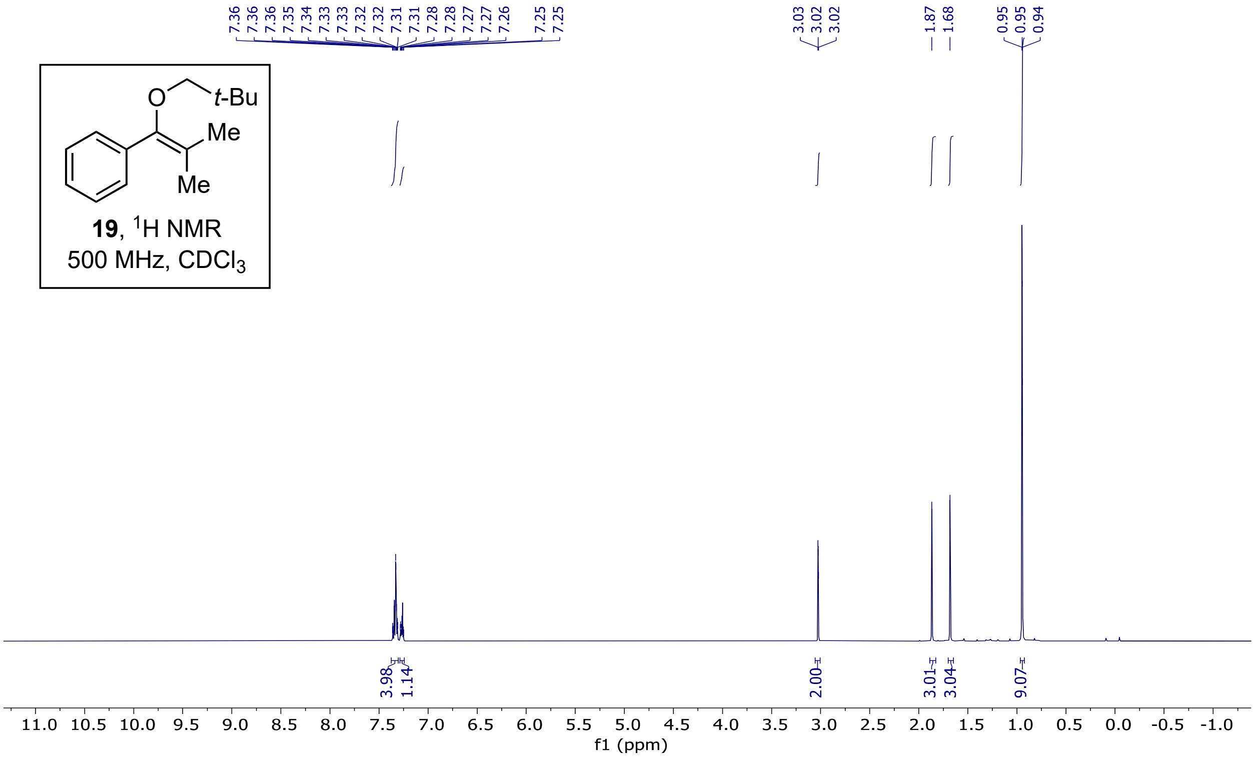

t-Bu
Me
Me
O
19, 1H NMR
500 MHz, CDCl3
7.36 7.36 7.36 7.35 7.34 7.33 7.33 7.32 7.32 7.31 7.31 7.28 7.28 7.27 7.27 7.26 7.25 7.25
3.03 3.02 3.02
1.87
1.68
0.95 0.95 0.94
3.98
1.14
2.00
3.01
3.04
9.07
11.0 10.5 10.0 9.5 9.0 8.5 8.0 7.5 7.0 6.5 6.0 5.5 5.0 4.5 4.0 3.5 3.0 2.5 2.0 1.5 1.0 0.5 0.0 -0.5 -1.0
f1 (ppm)

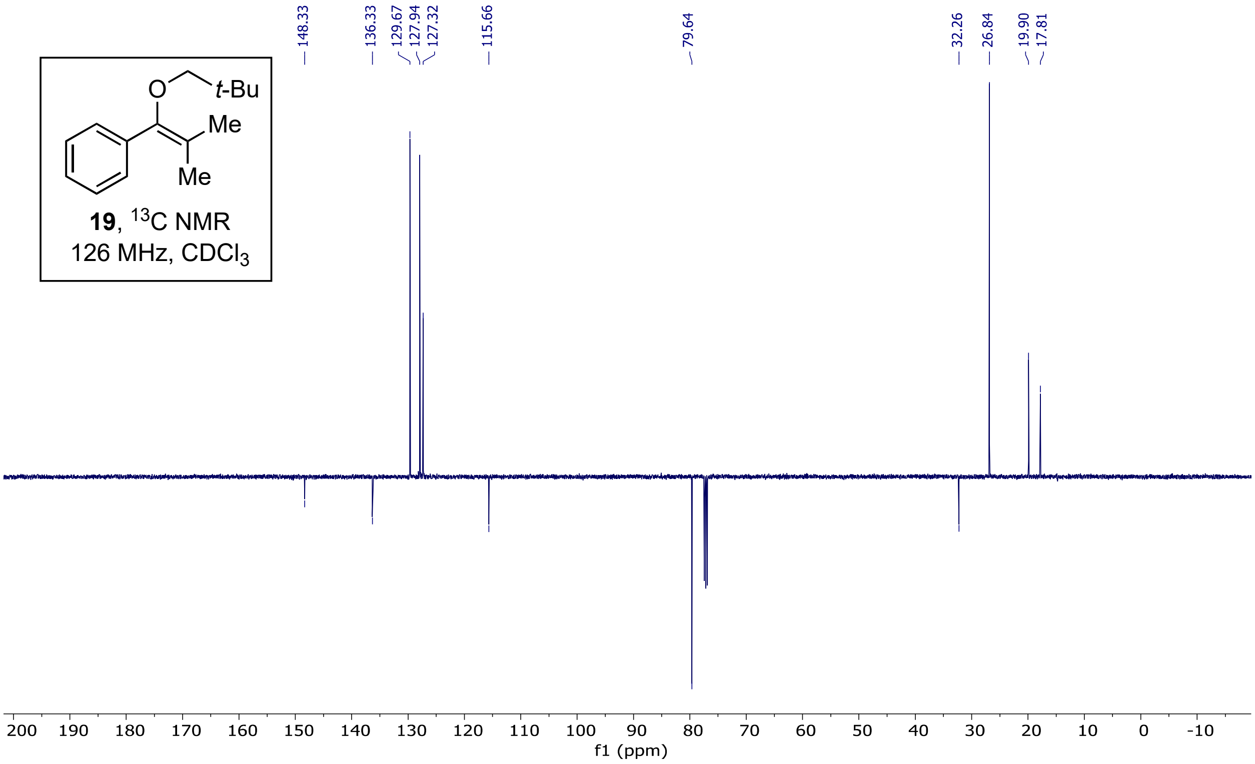

t-Bu
Me
Me
O
19, 13C NMR
126 MHz, CDCl3
148.33
136.33
129.67
127.94
127.32
115.66
79.64
32.26
26.84
19.90
17.81
200 190 180 170 160 150 140 130 120 110 100 90 80 70 60 50 40 30 20 10 0 -10
f1 (ppm)

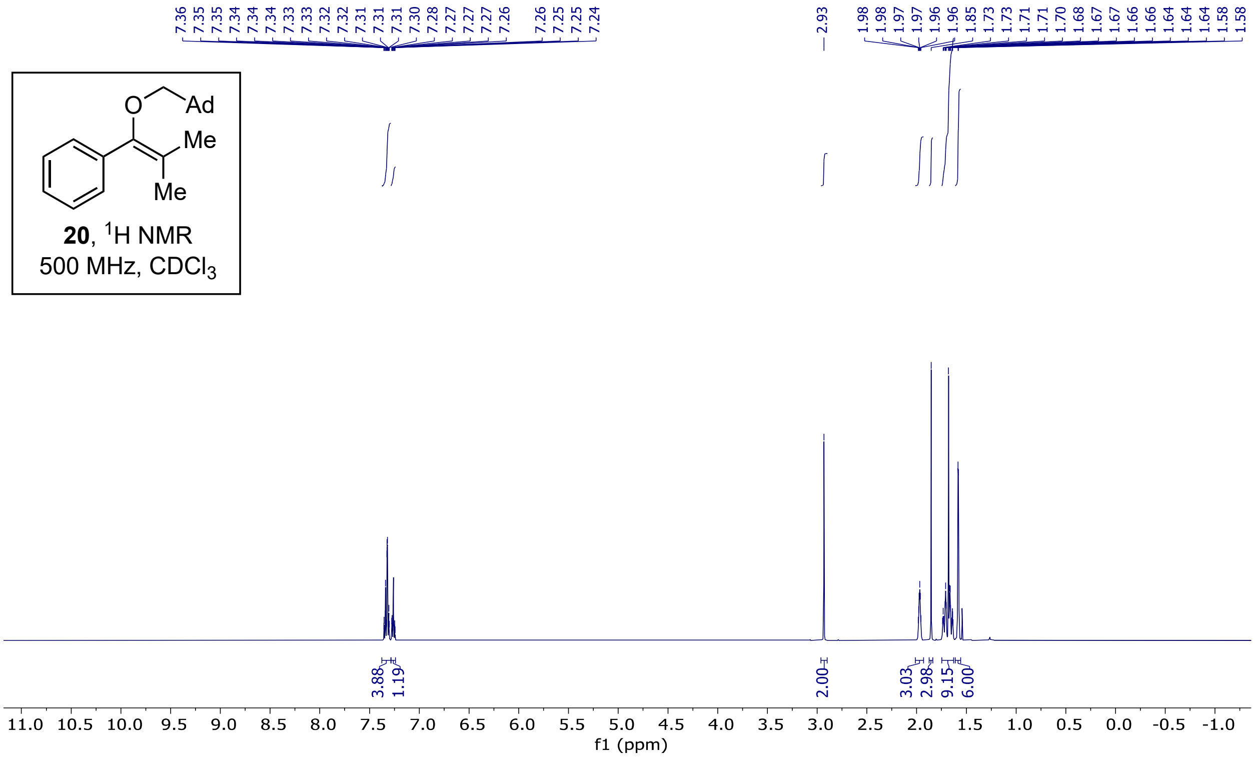
7.36 7.35 7.35 7.34 7.34 7.34 7.33 7.33 7.32 7.32 7.31 7.31 7.31 7.30 7.28 7.27 7.27 7.27 7.26 7.26 7.25 7.25 7.24
2.93
1.98 1.98 1.97 1.97 1.96 1.96 1.85 1.73 1.73 1.71 1.71 1.70 1.68 1.67 1.67 1.66 1.64 1.64 1.64 1.58 1.58
O
Ad
Me
Me
20, 1H NMR
500 MHz, CDCl3
3.88
1.19
2.00
3.03
2.98
9.15
6.00
f1 (ppm)

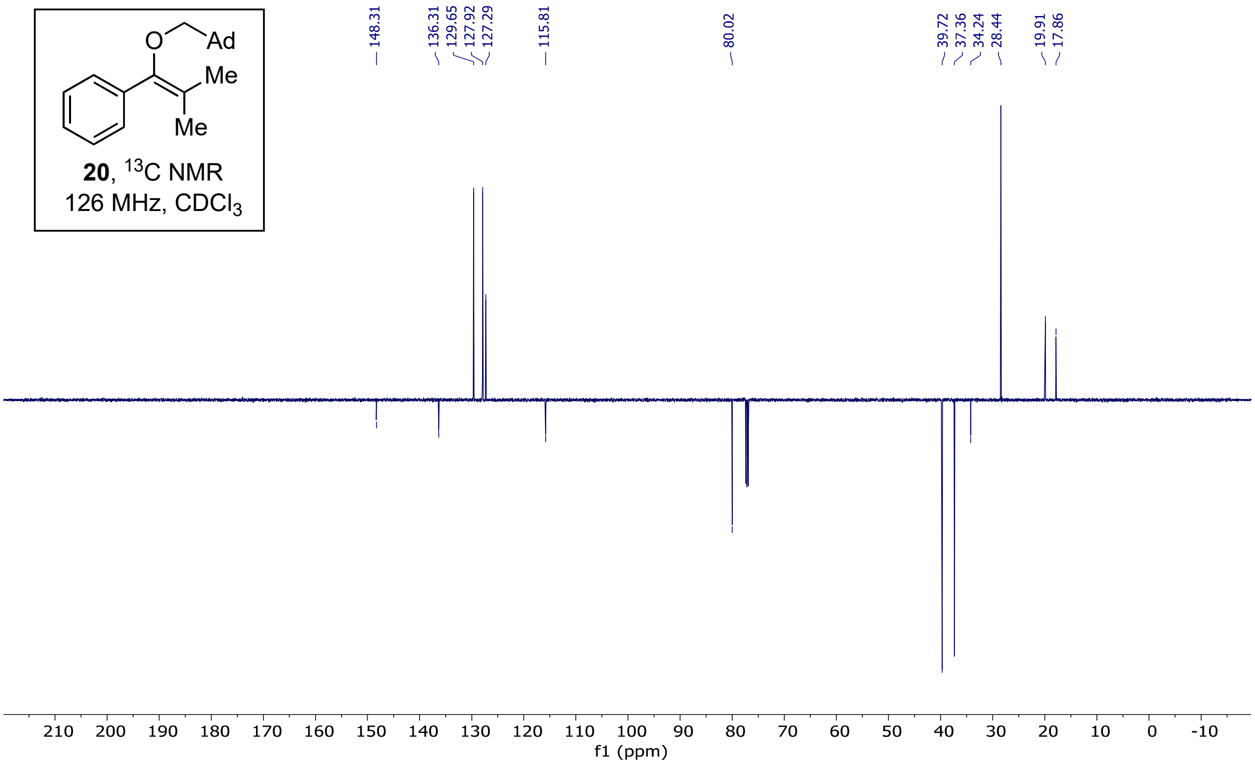
148.31
136.31
129.65
127.92
127.29
115.81
80.02
39.72
37.36
34.24
28.44
19.91
17.86
O
Ad
Me
Me
20, 13C NMR
126 MHz, CDCl3
f1 (ppm)

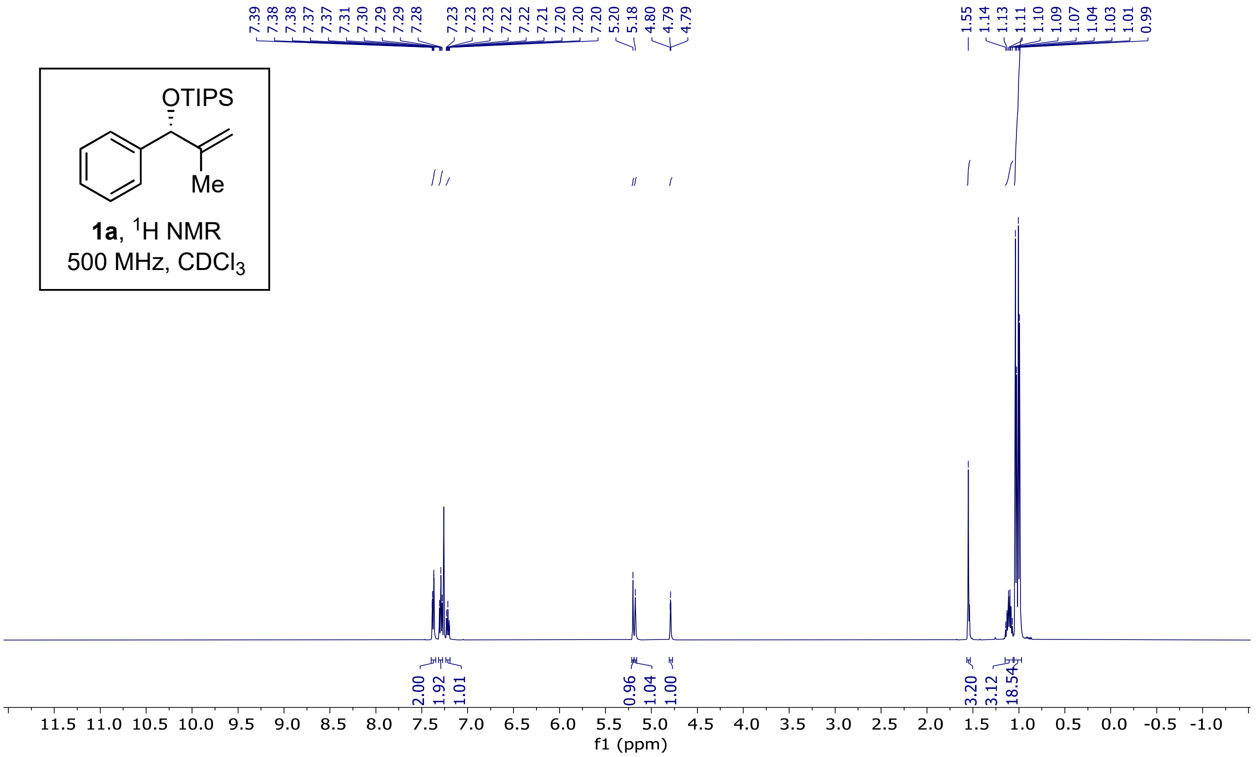
OTIPS
Me
1a, ¹H NMR
500 MHz, CDCl3
7.39 7.38 7.38 7.37 7.37 7.31 7.30 7.29 7.29 7.28 7.23 7.23 7.23 7.22 7.22 7.21 7.21 7.20 7.20 7.20 5.20 5.18 4.80 4.79 4.79
1.55 1.14 1.13 1.11 1.10 1.09 1.07 1.04 1.03 1.01 0.99
2.00 1.92 1.01 0.96 1.04 1.00 3.20 3.12 18.54
11.5 11.0 10.5 10.0 9.5 9.0 8.5 8.0 7.5 7.0 6.5 6.0 5.5 5.0 4.5 4.0 3.5 3.0 2.5 2.0 1.5 1.0 0.5 0.0 -0.5 -1.0
f1 (ppm)

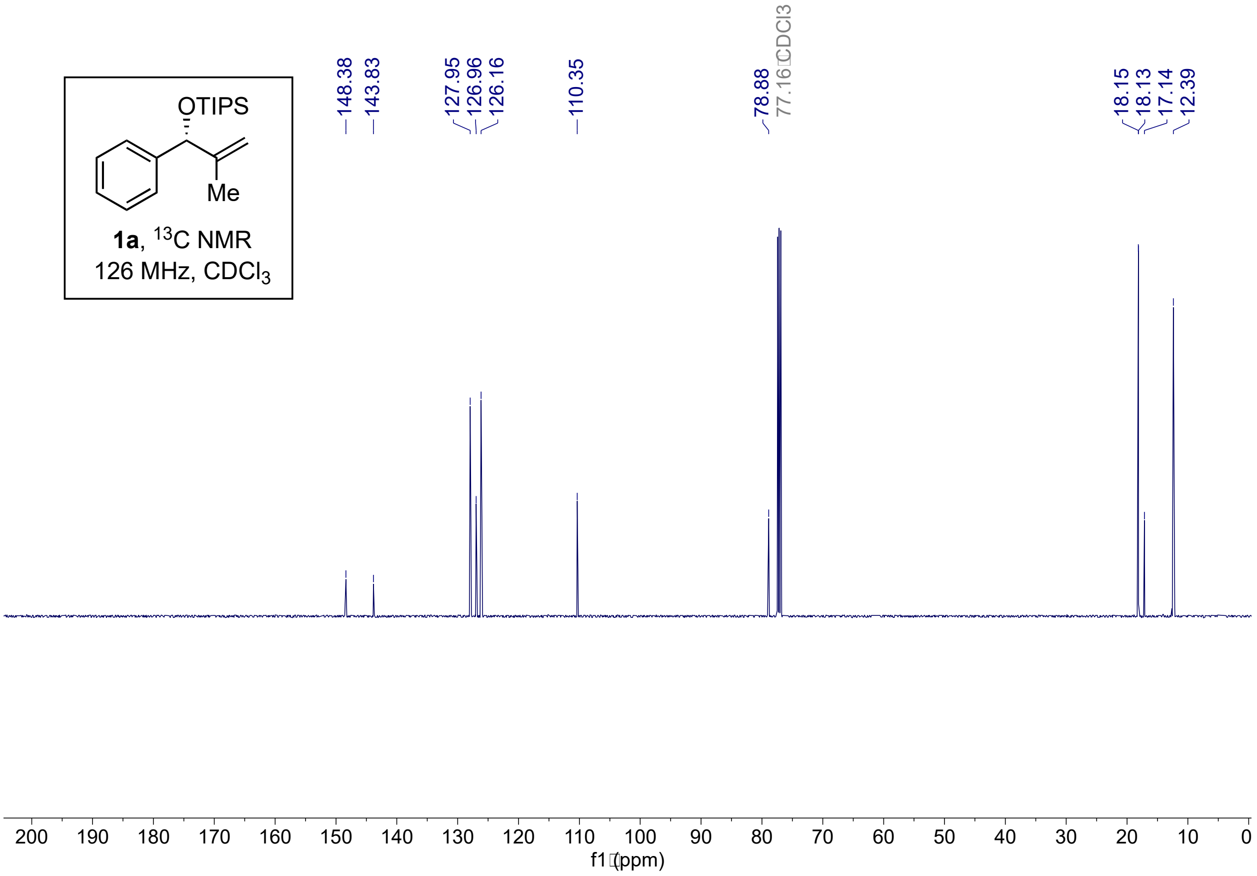
OTIPS
Me
1a, ¹³C NMR
126 MHz, CDCl3
148.38 143.83 127.95 126.96 126.16 110.35 78.88 77.16 CDCl3 18.15 18.13 17.14 12.39
200 190 180 170 160 150 140 130 120 110 100 90 80 70 60 50 40 30 20 10 0
f1 (ppm)

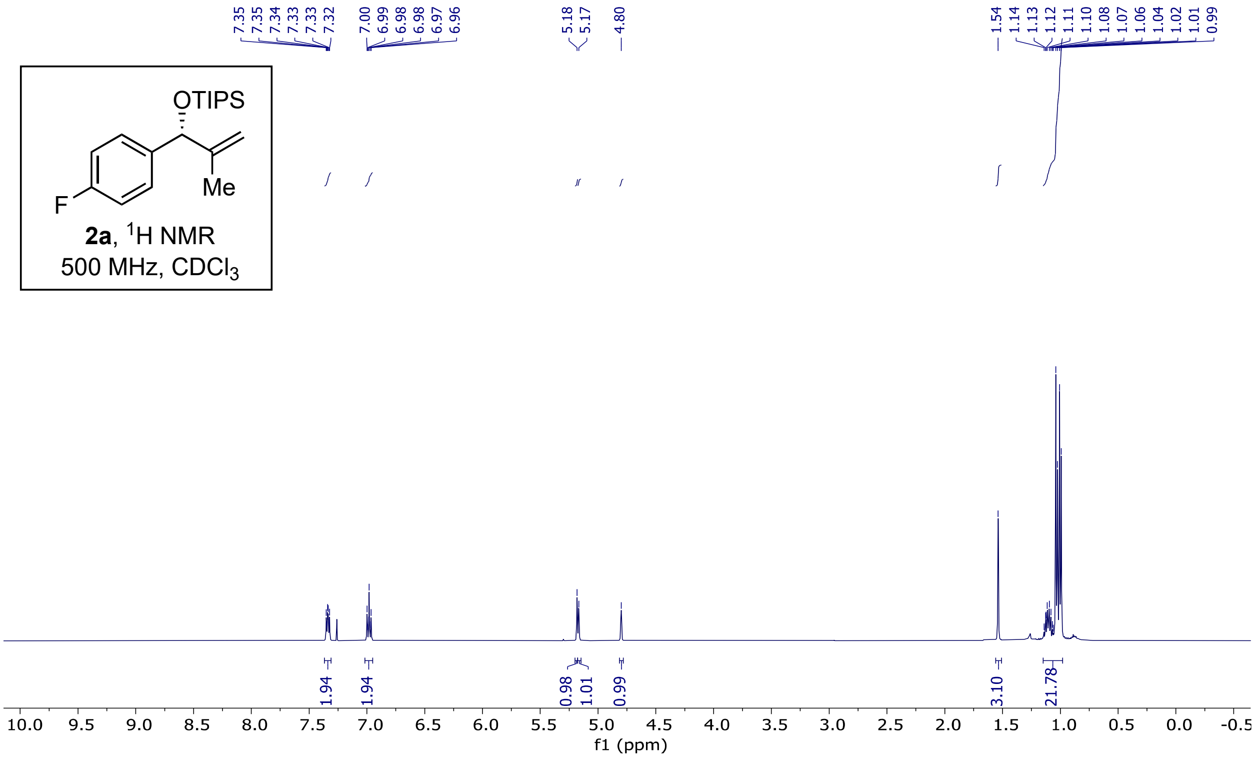
7.35
7.35
7.34
7.33
7.33
7.32
7.00
6.99
6.98
6.98
6.97
6.96
5.18
5.17
4.80
1.54
1.14
1.13
1.12
1.11
1.10
1.08
1.07
1.06
1.04
1.02
1.01
0.99
OTIPS
Me
F
2a, 1H NMR
500 MHz, CDCl3
1.94
1.94
0.98
1.01
0.99
3.10
21.78
10.0 9.5 9.0 8.5 8.0 7.5 7.0 6.5 6.0 5.5 5.0 4.5 4.0 3.5 3.0 2.5 2.0 1.5 1.0 0.5 0.0 -0.5
f1 (ppm)

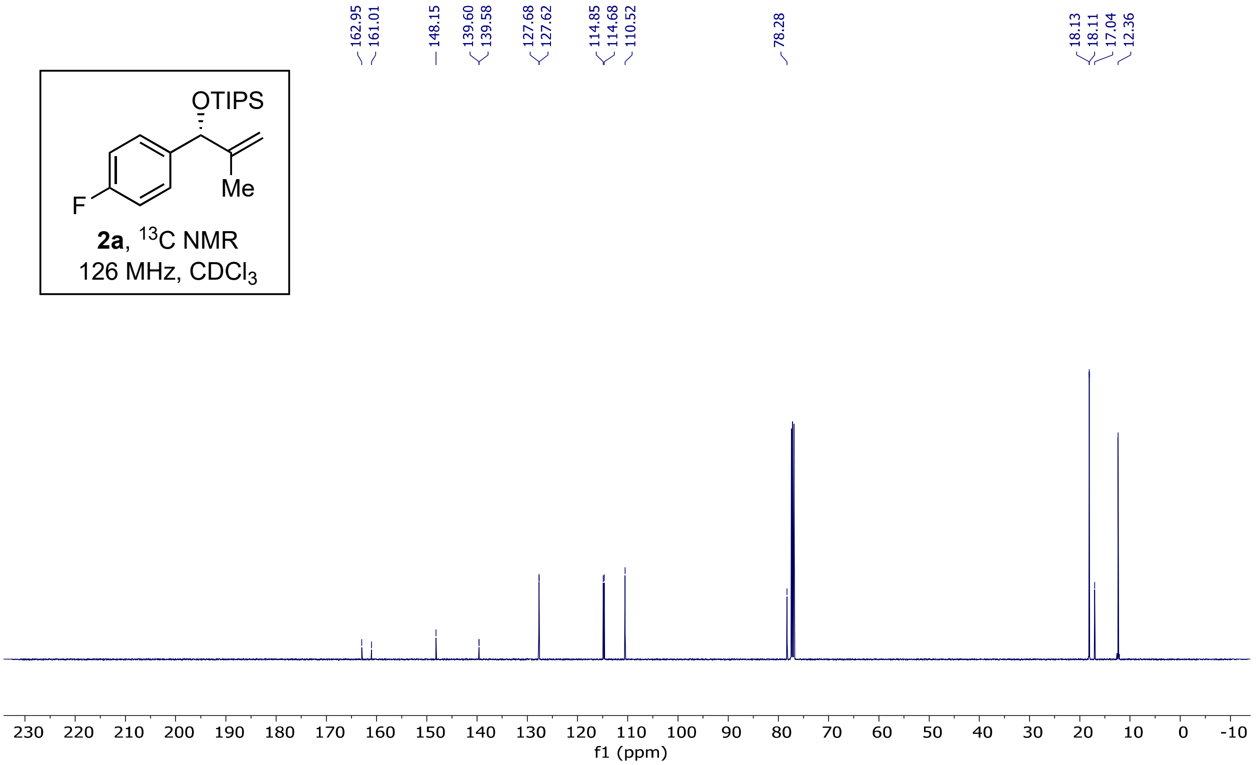
162.95
161.01
148.15
139.60
139.58
127.68
127.62
114.85
114.68
110.52
78.28
18.13
18.11
17.04
12.36
OTIPS
Me
F
2a, 13C NMR
126 MHz, CDCl3
230 220 210 200 190 180 170 160 150 140 130 120 110 100 90 80 70 60 50 40 30 20 10 0 -10
f1 (ppm)

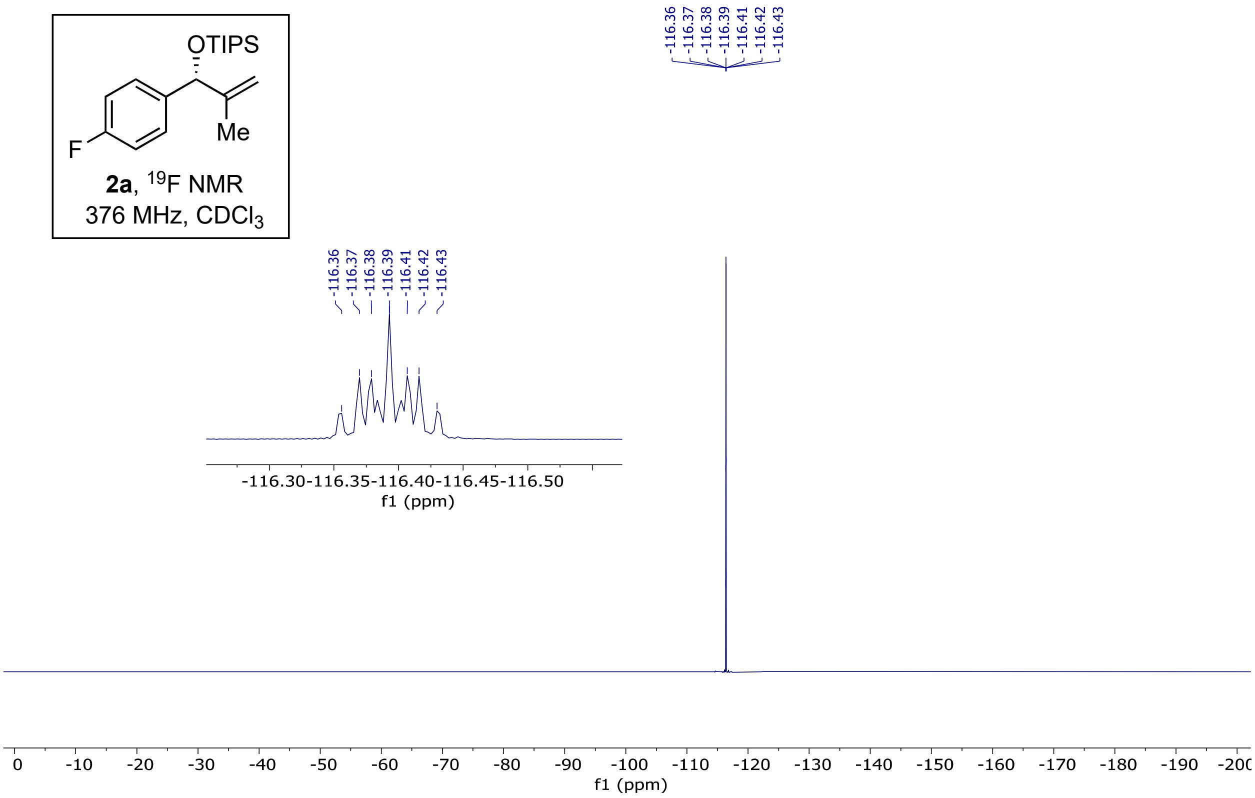
OTIPS
F
Me
2a, 19F NMR
376 MHz, CDCl3
-116.36
-116.37
-116.38
-116.39
-116.41
-116.42
-116.43
-116.30
-116.35
-116.40
-116.45
-116.50
f1 (ppm)
0
-10
-20
-30
-40
-50
-60
-70
-80
-90
-100
-110
-120
-130
-140
-150
-160
-170
-180
-190
-200
f1 (ppm)

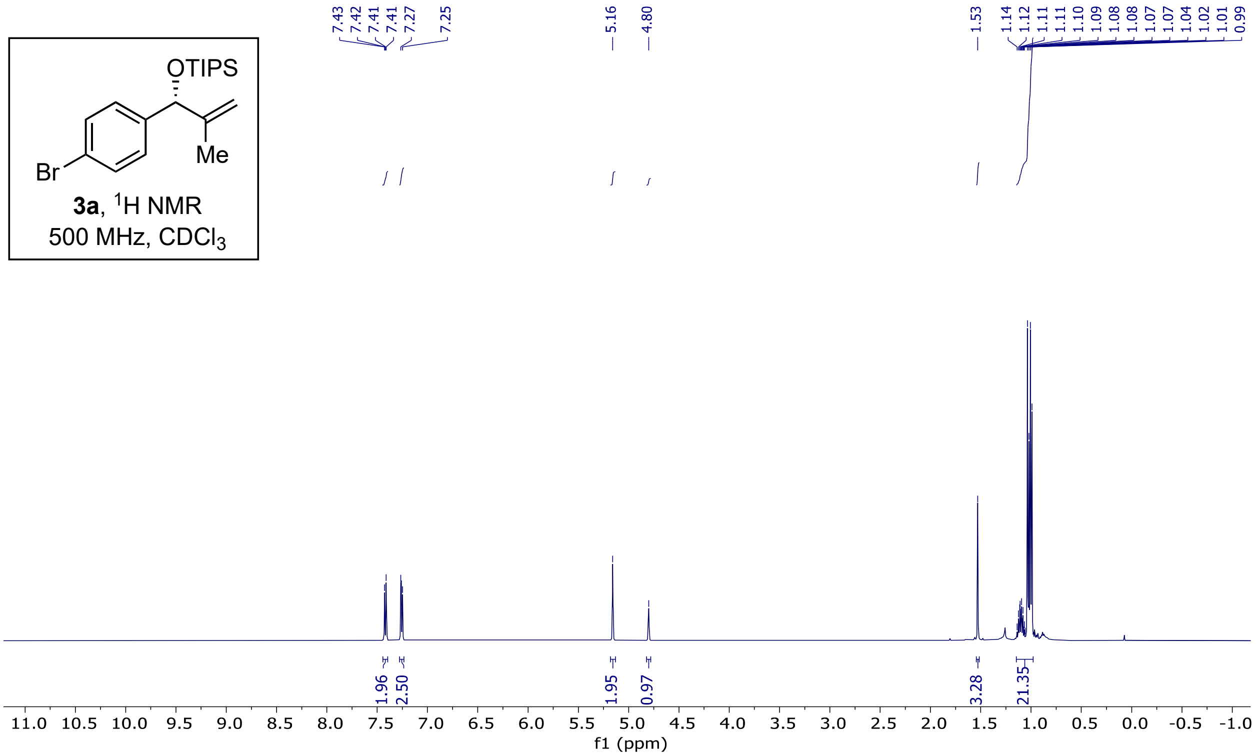
OTIPS
Br
Me
3a, 1H NMR
500 MHz, CDCl3
7.43
7.42
7.41
7.41
7.27
7.25
5.16
4.80
1.53
1.14
1.12
1.11
1.11
1.10
1.09
1.08
1.07
1.07
1.04
1.02
1.01
0.99
1.96
2.50
1.95
0.97
3.28
21.35
11.0 10.5 10.0 9.5 9.0 8.5 8.0 7.5 7.0 6.5 6.0 5.5 5.0 4.5 4.0 3.5 3.0 2.5 2.0 1.5 1.0 0.5 0.0 -0.5 -1.0
f1 (ppm)

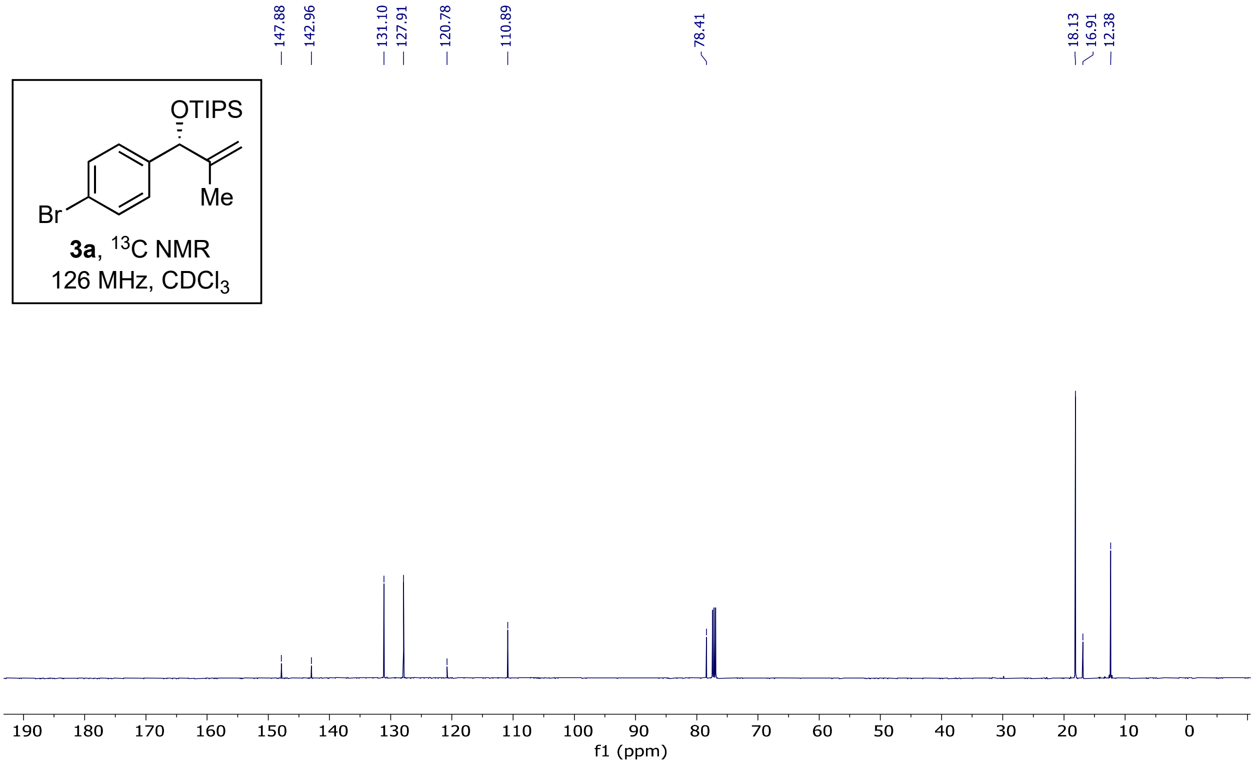
OTIPS
Br
Me
3a, 13C NMR
126 MHz, CDCl3
147.88
142.96
131.10
127.91
120.78
110.89
78.41
18.13
16.91
12.38
190 180 170 160 150 140 130 120 110 100 90 80 70 60 50 40 30 20 10 0
f1 (ppm)

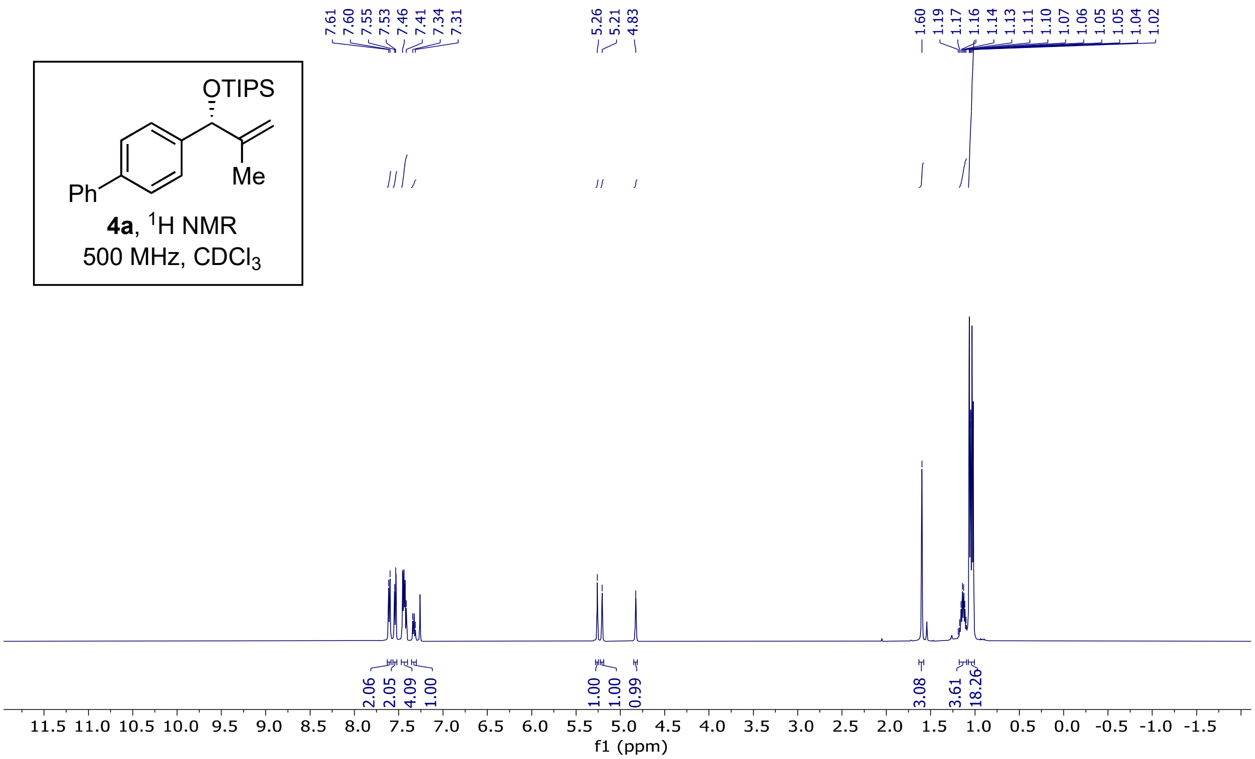
OTIPS
Me
Ph
4a, 1H NMR
500 MHz, CDCl3
7.61 7.60 7.55 7.53 7.46 7.41 7.34 7.31
5.26 5.21 4.83
1.60 1.19 1.17 1.16 1.14 1.13 1.11 1.10 1.07 1.06 1.05 1.05 1.04 1.02
2.06 2.05 4.09 1.00
1.00 1.00 0.99
3.08 3.61 18.26
f1 (ppm)

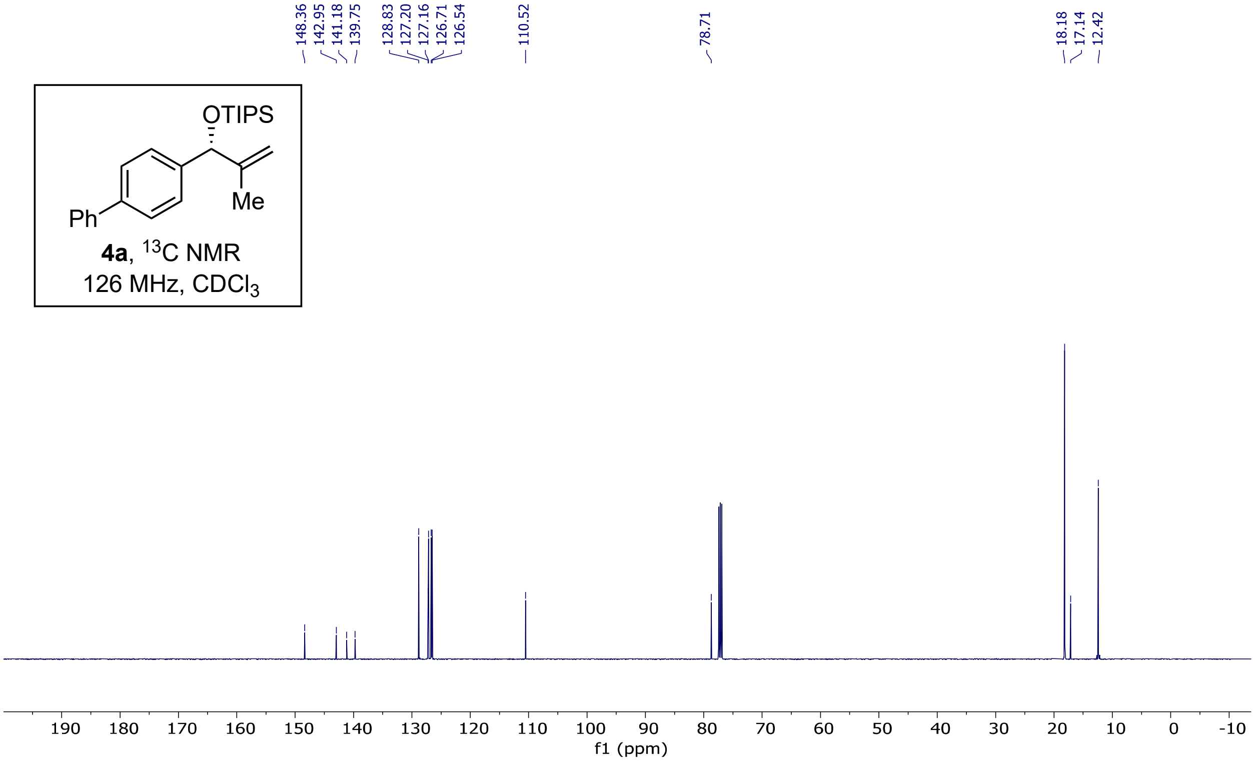
OTIPS
Me
Ph
4a, 13C NMR
126 MHz, CDCl3
148.36 142.95 141.18 139.75
128.83 127.20 127.16 126.71 126.54
110.52
78.71
18.18 17.14 12.42
f1 (ppm)

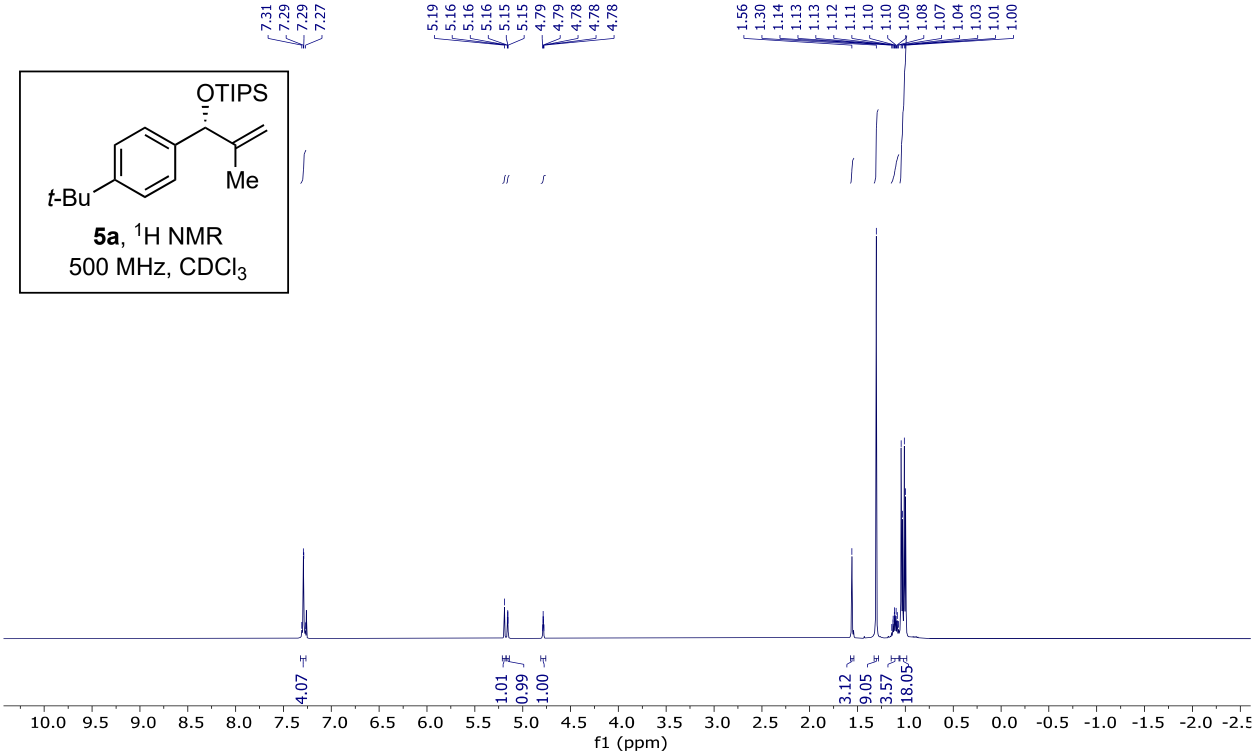
OTIPS
t-Bu
Me
5a, ¹H NMR
500 MHz, CDCl₃
f1 (ppm)

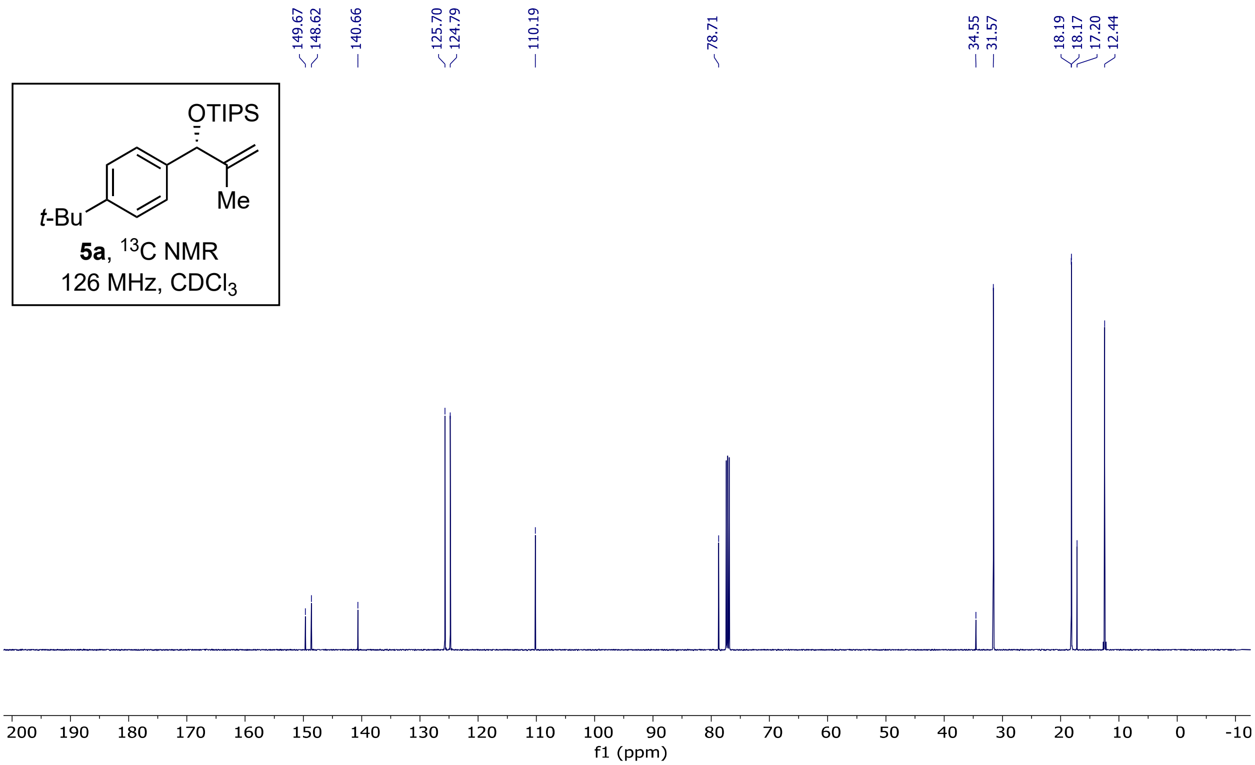
OTIPS
t-Bu
Me
5a, ¹³C NMR
126 MHz, CDCl₃
f1 (ppm)

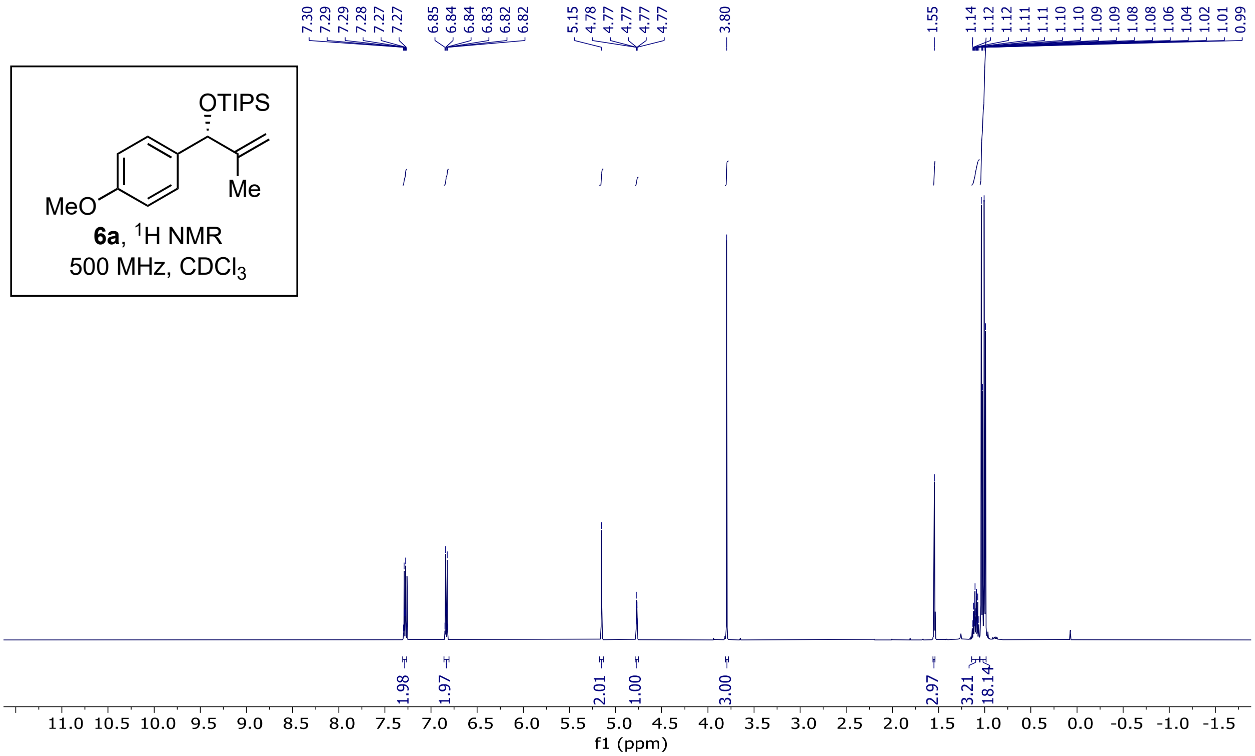

OTIPS
Me
MeO
6a, ¹H NMR
500 MHz, CDCl3
f1 (ppm)

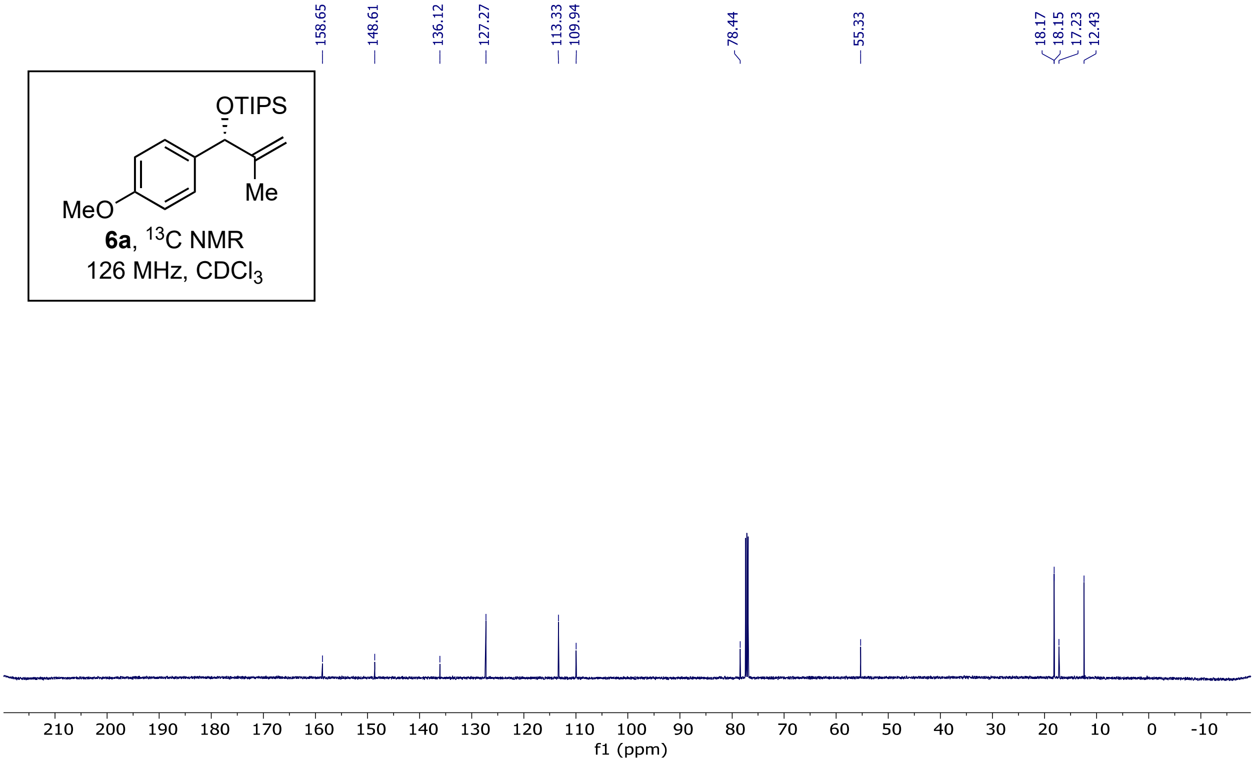

OTIPS
Me
MeO
6a, ¹³C NMR
126 MHz, CDCl3
f1 (ppm)

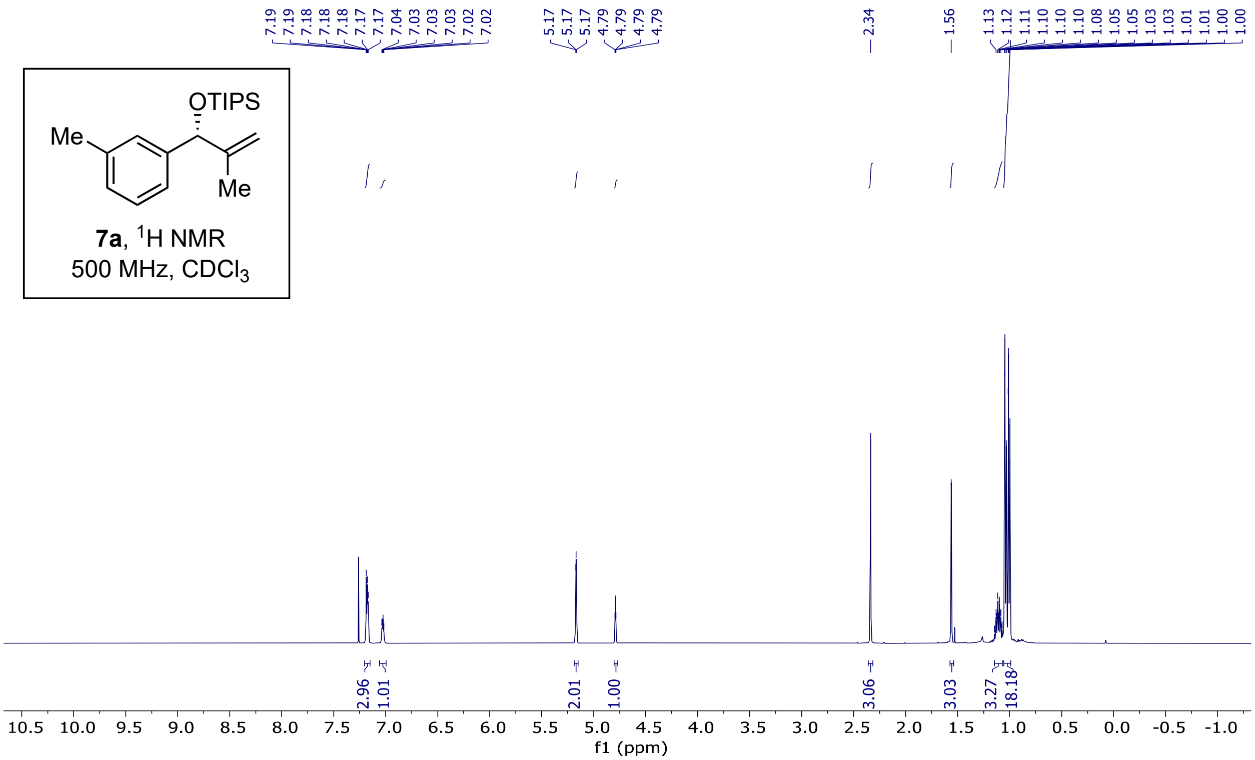
7.19 7.19 7.18 7.18 7.18 7.17 7.17 7.04 7.03 7.03 7.03 7.02 7.02
5.17 5.17 5.17 4.79 4.79 4.79 4.79
2.34
1.56
1.13 1.12 1.11 1.10 1.10 1.10 1.08 1.05 1.03 1.03 1.01 1.01 1.00 1.00
OTIPS
Me
Me
7a, 1H NMR
500 MHz, CDCl3
2.96 1.01 2.01 1.00 3.06 3.03 3.27 18.18
10.5 10.0 9.5 9.0 8.5 8.0 7.5 7.0 6.5 6.0 5.5 5.0 4.5 4.0 3.5 3.0 2.5 2.0 1.5 1.0 0.5 0.0 -0.5 -1.0
f1 (ppm)

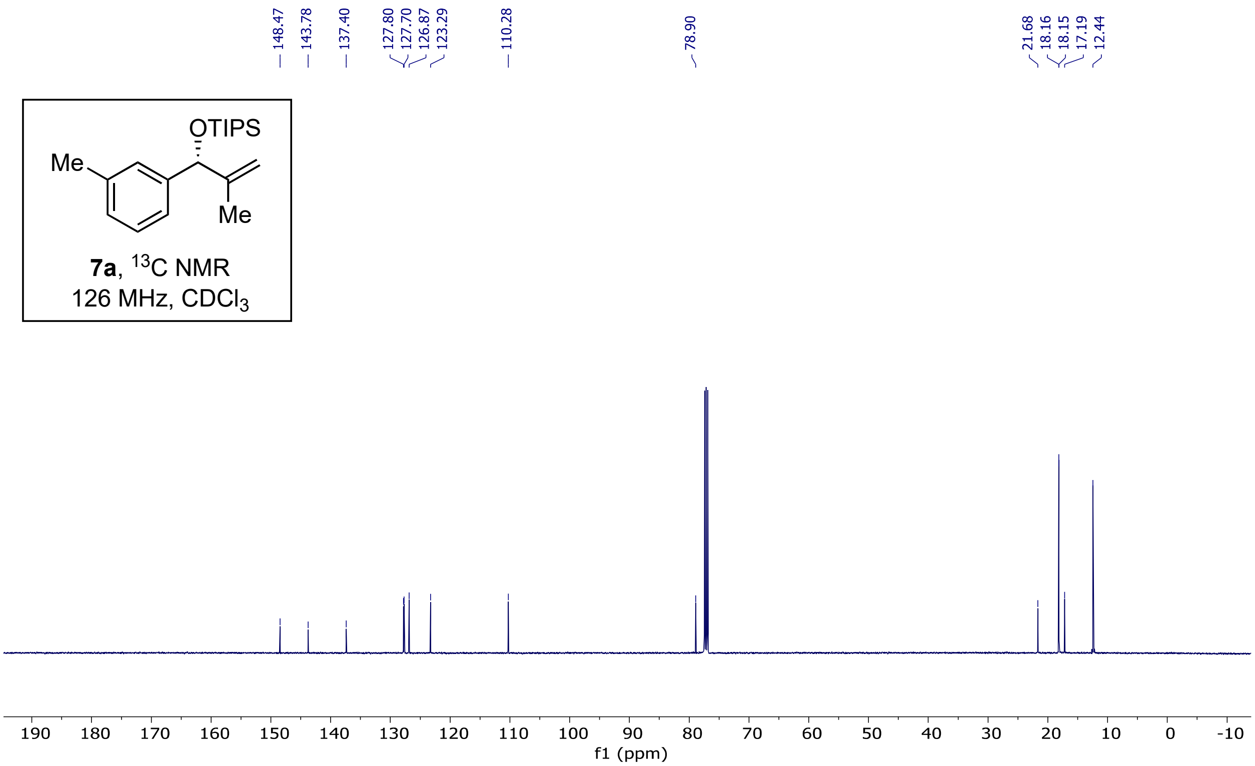
148.47
143.78
137.40
127.80 127.70 126.87 123.29
110.28
78.90
21.68 18.16 18.15 17.19 12.44
OTIPS
Me
Me
7a, 13C NMR
126 MHz, CDCl3
190 180 170 160 150 140 130 120 110 100 90 80 70 60 50 40 30 20 10 0 -10
f1 (ppm)

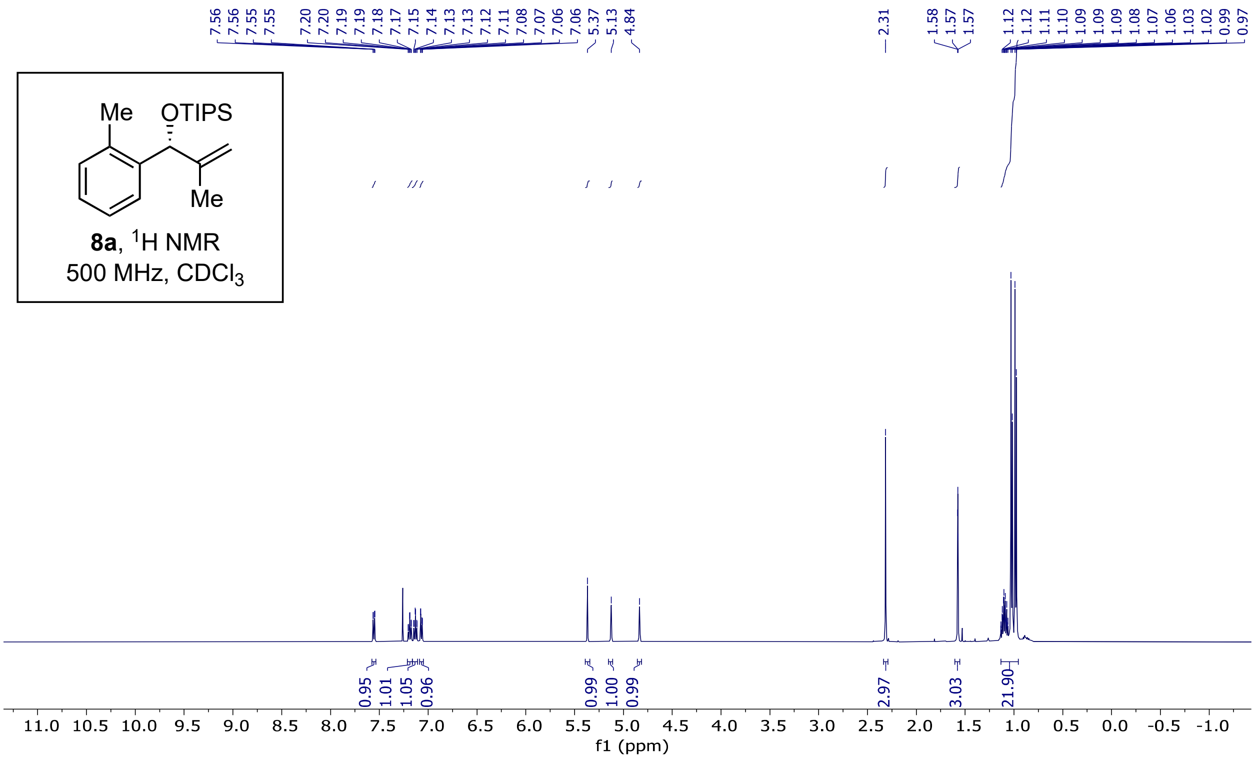
Me
OTIPS
Me
8a, ¹H NMR
500 MHz, CDCl3
7.56 7.56 7.55 7.55 7.20 7.20 7.19 7.19 7.18 7.17 7.15 7.14 7.13 7.13 7.12 7.11 7.08 7.07 7.06 7.06 5.37 5.13 4.84
2.31 1.58 1.57 1.57 1.12 1.12 1.11 1.10 1.09 1.09 1.09 1.08 1.07 1.06 1.03 1.02 0.99 0.97
0.95 1.01 1.05 0.96 0.99 1.00 0.99 2.97 3.03 21.90
11.0 10.5 10.0 9.5 9.0 8.5 8.0 7.5 7.0 6.5 6.0 5.5 5.0 4.5 4.0 3.5 3.0 2.5 2.0 1.5 1.0 0.5 0.0 -0.5 -1.0
f1 (ppm)

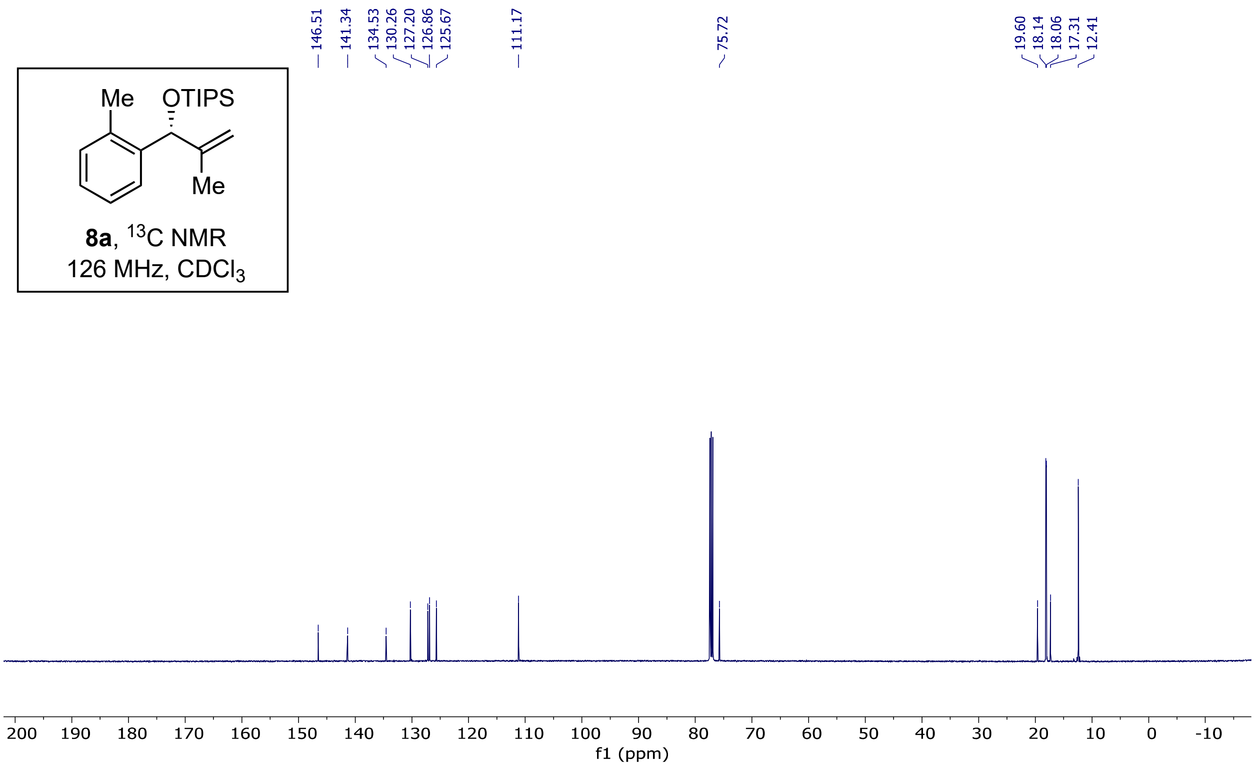
Me
OTIPS
Me
8a, ¹³C NMR
126 MHz, CDCl3
146.51 141.34 134.53 130.26 127.20 126.86 125.67 111.17 75.72 19.60 18.14 18.06 17.31 12.41
200 190 180 170 160 150 140 130 120 110 100 90 80 70 60 50 40 30 20 10 0 -10
f1 (ppm)

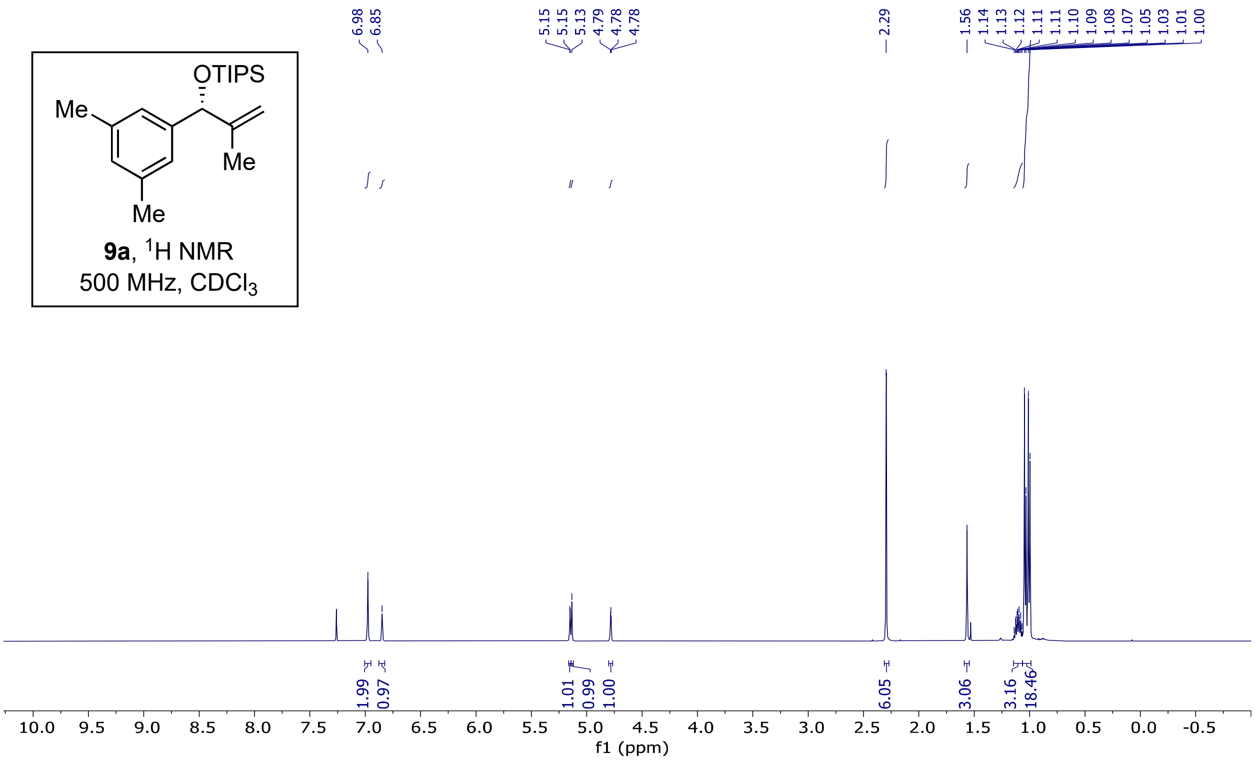

OTIPS
Me
Me
Me
9a, 1H NMR
500 MHz, CDCl3
6.98
6.85
5.15
5.15
5.13
4.79
4.78
4.78
2.29
1.56
1.14
1.13
1.12
1.11
1.11
1.10
1.09
1.08
1.07
1.05
1.03
1.01
1.00
1.99
0.97
1.01
0.99
1.00
6.05
3.06
3.16
18.46
f1 (ppm)

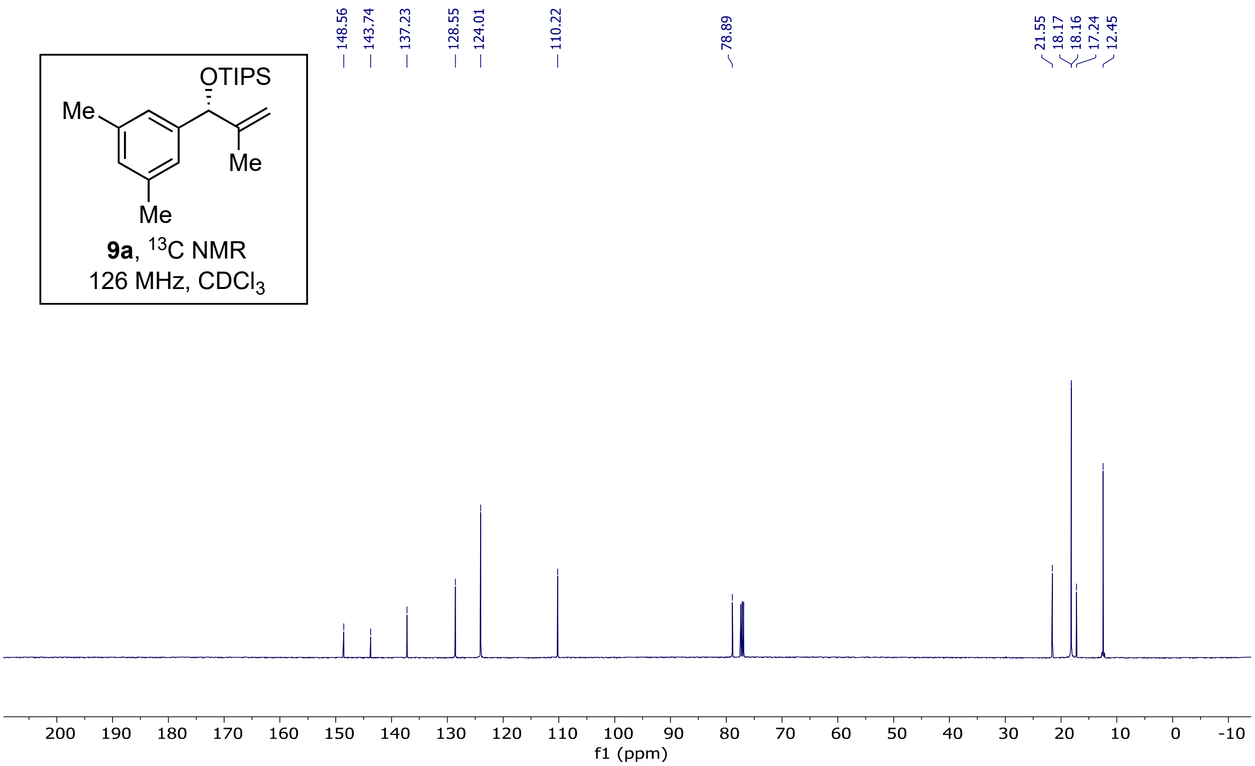

OTIPS
Me
Me
Me
9a, 13C NMR
126 MHz, CDCl3
148.56
143.74
137.23
128.55
124.01
110.22
78.89
21.55
18.17
18.16
17.24
12.45
f1 (ppm)

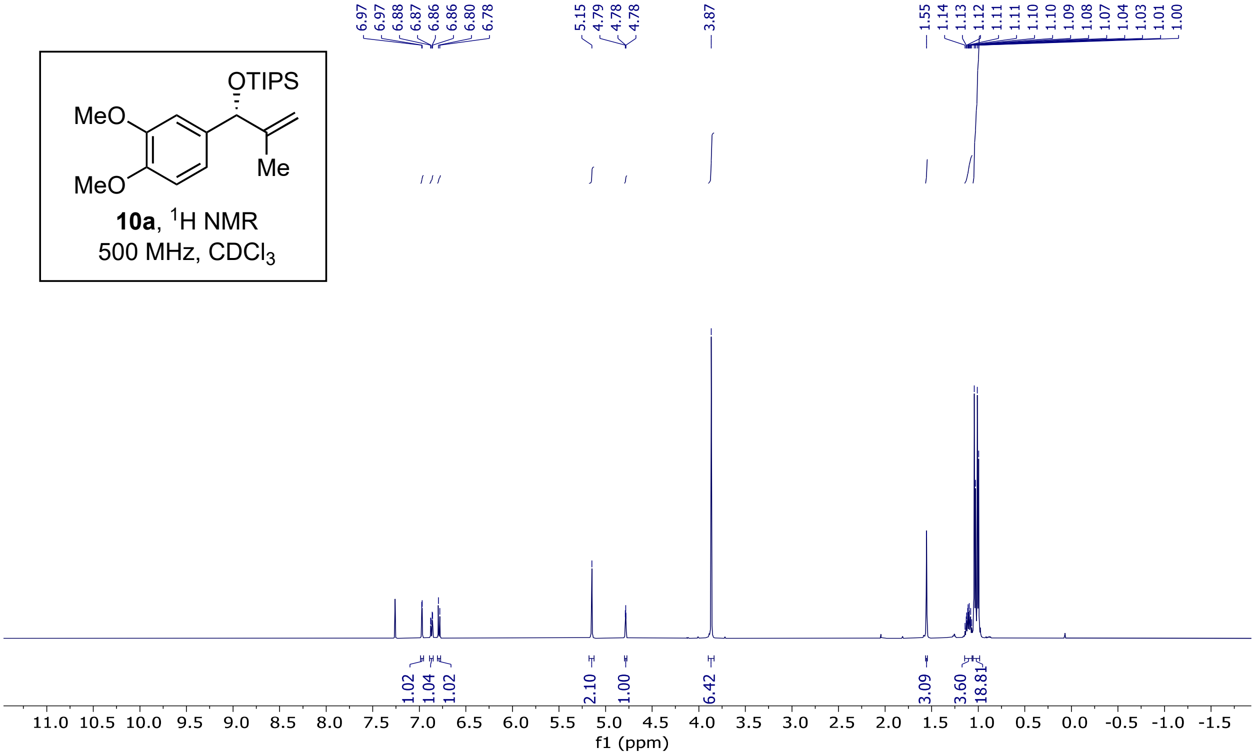
OTIPS
MeO
MeO
Me
10a, ¹H NMR
500 MHz, CDCl3
6.97 6.97 6.88 6.87 6.86 6.86 6.80 6.78
5.15 4.79 4.78 4.78
3.87
1.55 1.14 1.13 1.12 1.11 1.11 1.10 1.10 1.09 1.08 1.07 1.04 1.03 1.01 1.00
1.02 1.04 1.02 2.10 1.00 6.42 3.09 3.60 18.81
f1 (ppm)

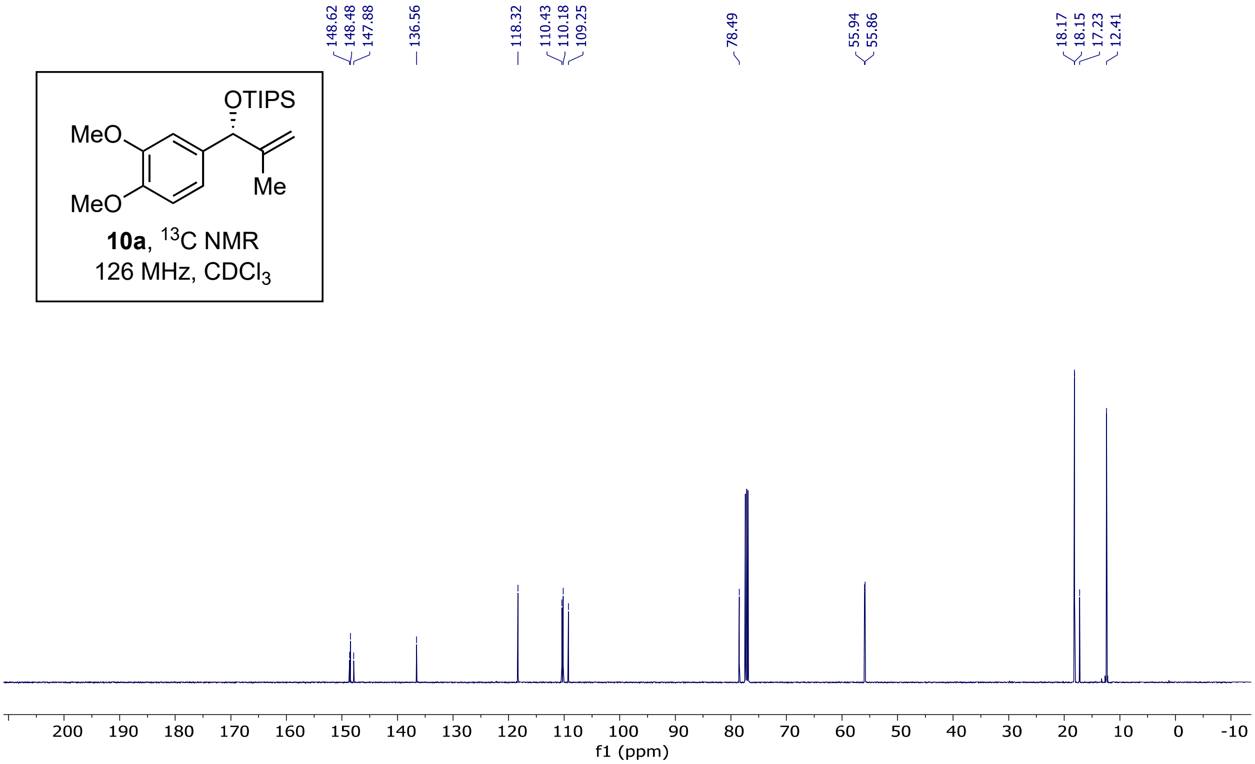
OTIPS
MeO
MeO
Me
10a, ¹³C NMR
126 MHz, CDCl3
148.62 148.48 147.88 136.56 118.32 110.43 110.18 109.25 78.49 55.94 55.86 18.17 18.15 17.23 12.41
f1 (ppm)

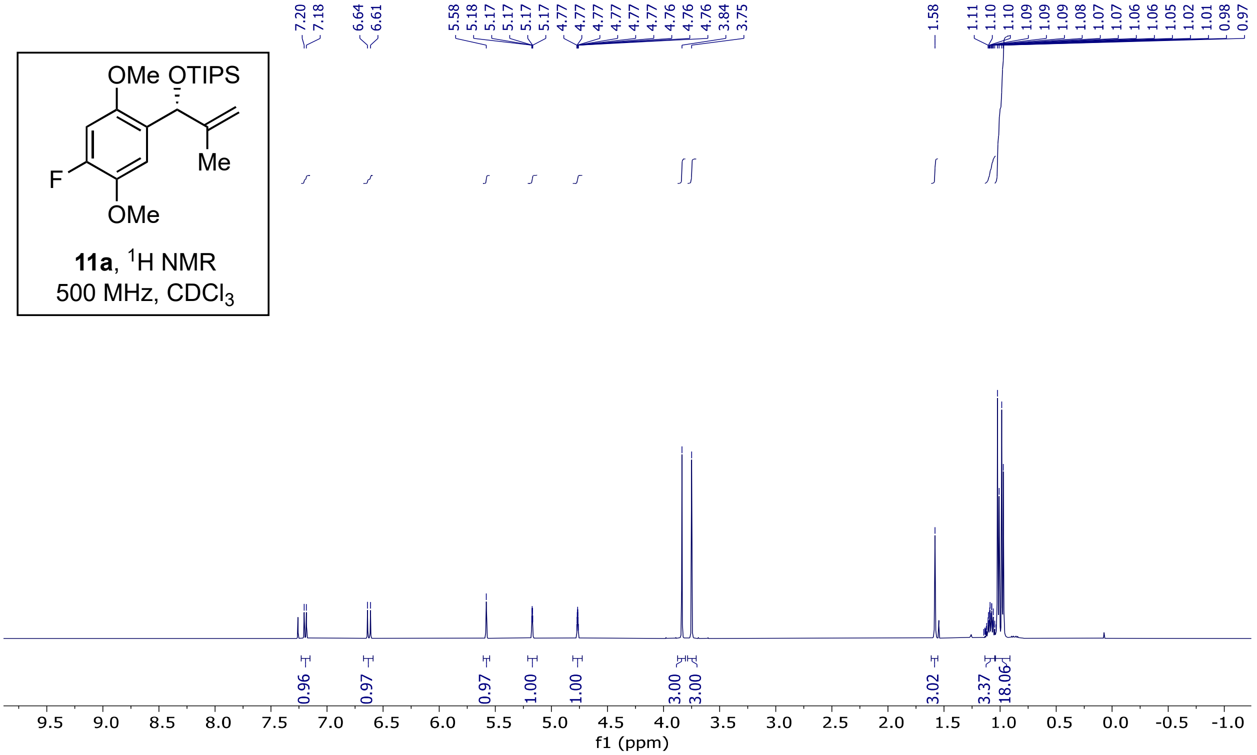
OMe OTIPS
Me
F
OMe
11a, ¹H NMR
500 MHz, CDCl3
7.20
7.18
6.64
6.61
5.58
5.18
5.17
5.17
5.17
5.17
4.77
4.77
4.77
4.77
4.77
4.77
4.76
4.76
4.76
3.84
3.75
1.58
1.11
1.10
1.10
1.09
1.09
1.09
1.08
1.07
1.07
1.06
1.06
1.05
1.02
1.01
0.98
0.97
0.96
0.97
0.97
1.00
1.00
3.00
3.00
3.02
3.37
18.06
9.5 9.0 8.5 8.0 7.5 7.0 6.5 6.0 5.5 5.0 4.5 4.0 3.5 3.0 2.5 2.0 1.5 1.0 0.5 0.0 -0.5 -1.0
f1 (ppm)

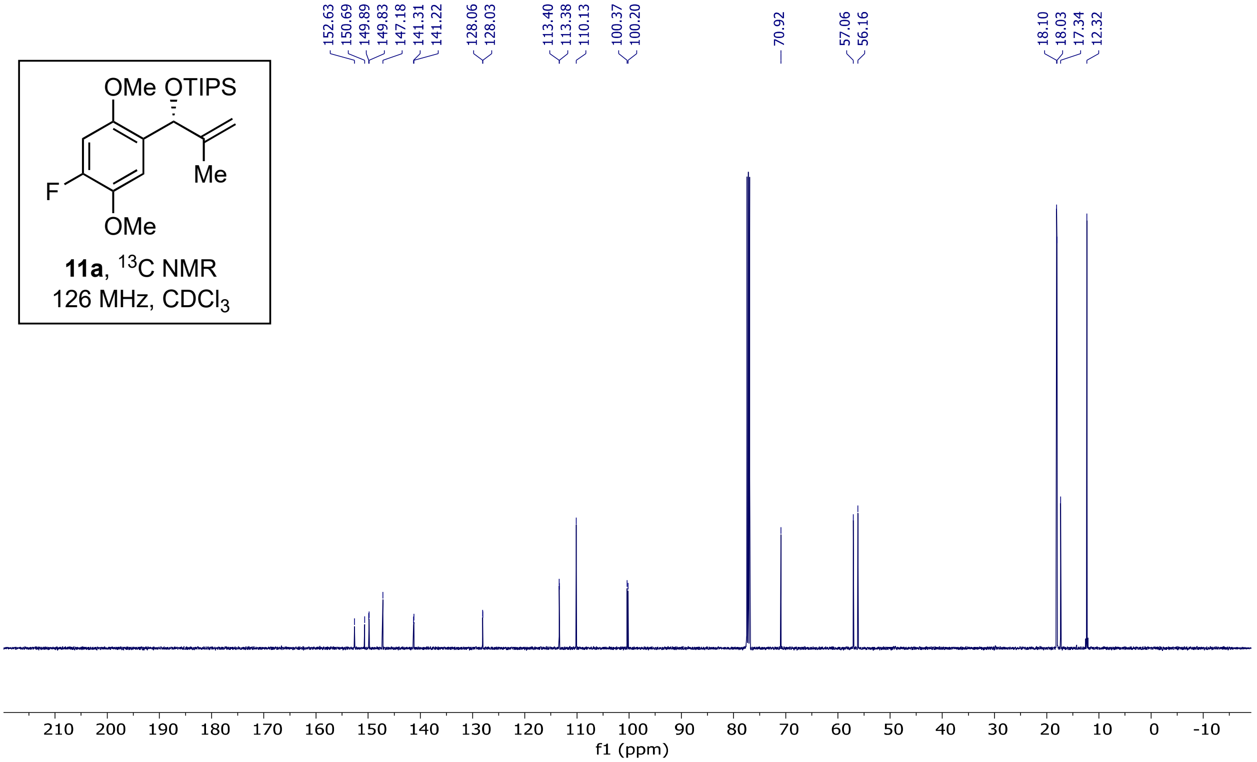
OMe OTIPS
Me
F
OMe
11a, ¹³C NMR
126 MHz, CDCl3
152.63
150.69
149.89
149.83
147.18
141.31
141.22
128.06
128.03
113.40
113.38
110.13
100.37
100.20
70.92
57.06
56.16
18.10
18.03
17.34
12.32
210 200 190 180 170 160 150 140 130 120 110 100 90 80 70 60 50 40 30 20 10 0 -10
f1 (ppm)

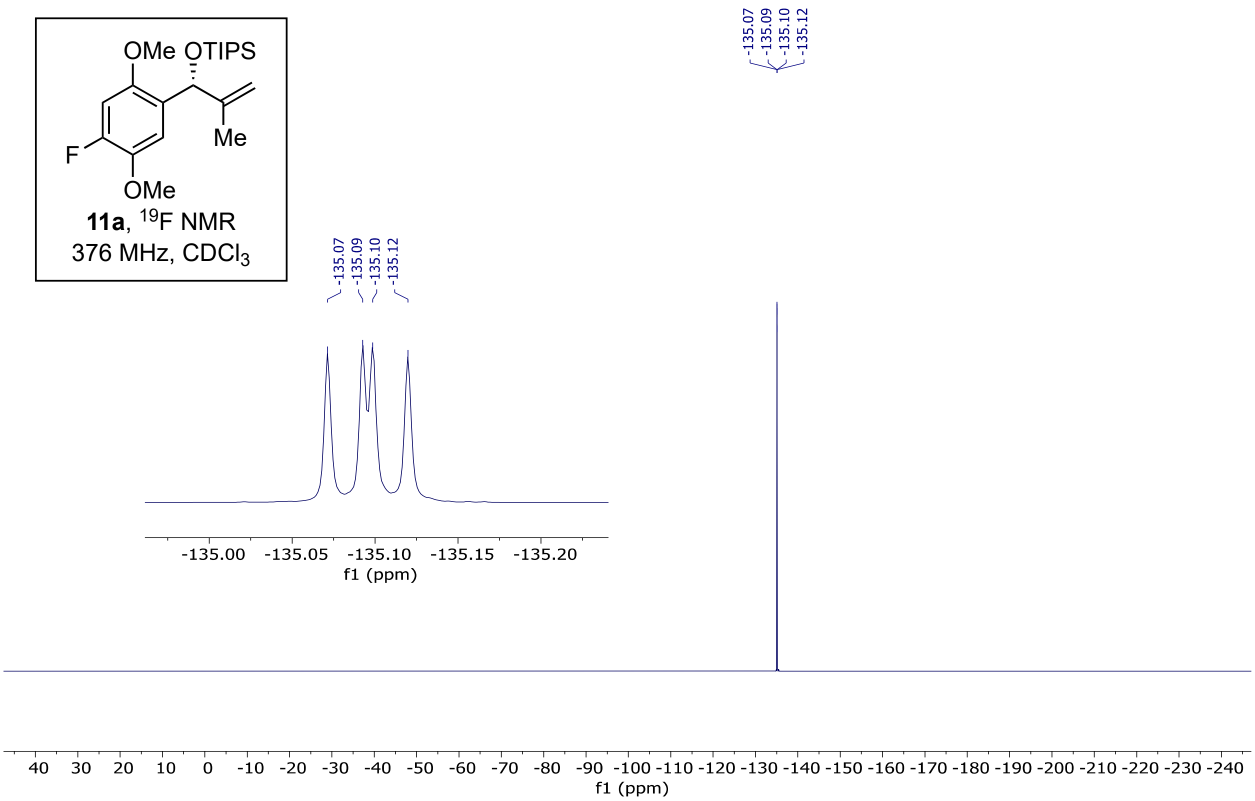
OMe OTIPS
F
Me
OMe
11a, 19F NMR
376 MHz, CDCl3
-135.07
-135.09
-135.10
-135.12
-135.00 -135.05 -135.10 -135.15 -135.20
f1 (ppm)
40 30 20 10 0 -10 -20 -30 -40 -50 -60 -70 -80 -90 -100 -110 -120 -130 -140 -150 -160 -170 -180 -190 -200 -210 -220 -230 -240
f1 (ppm)

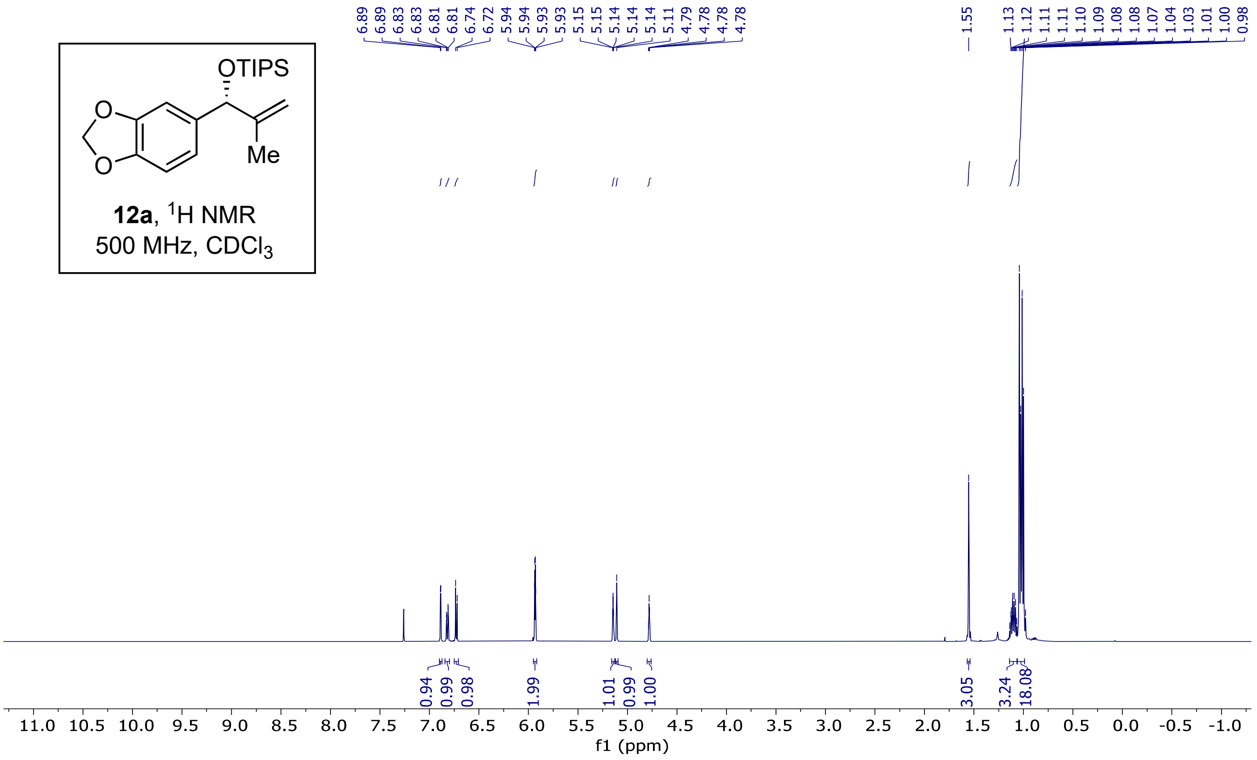
OTIPS
Me
12a, ¹H NMR
500 MHz, CDCl₃
f1 (ppm)

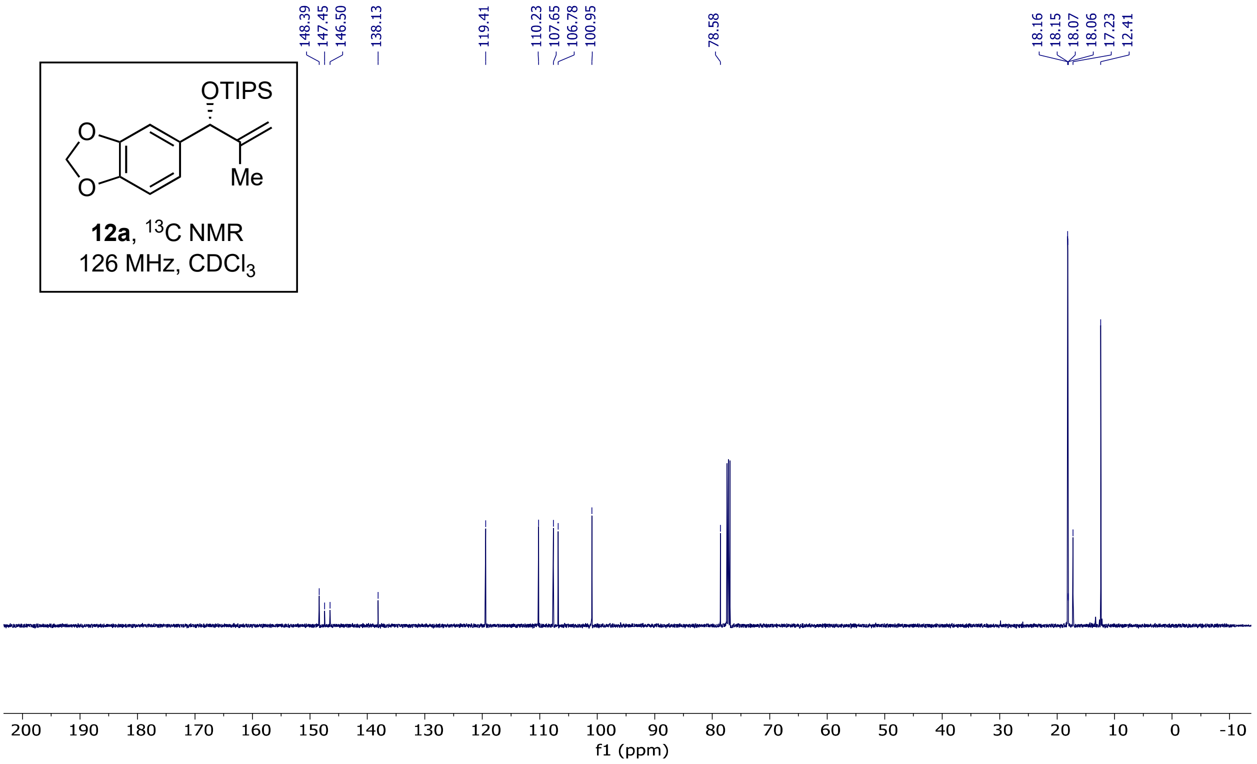
OTIPS
Me
12a, ¹³C NMR
126 MHz, CDCl₃
f1 (ppm)

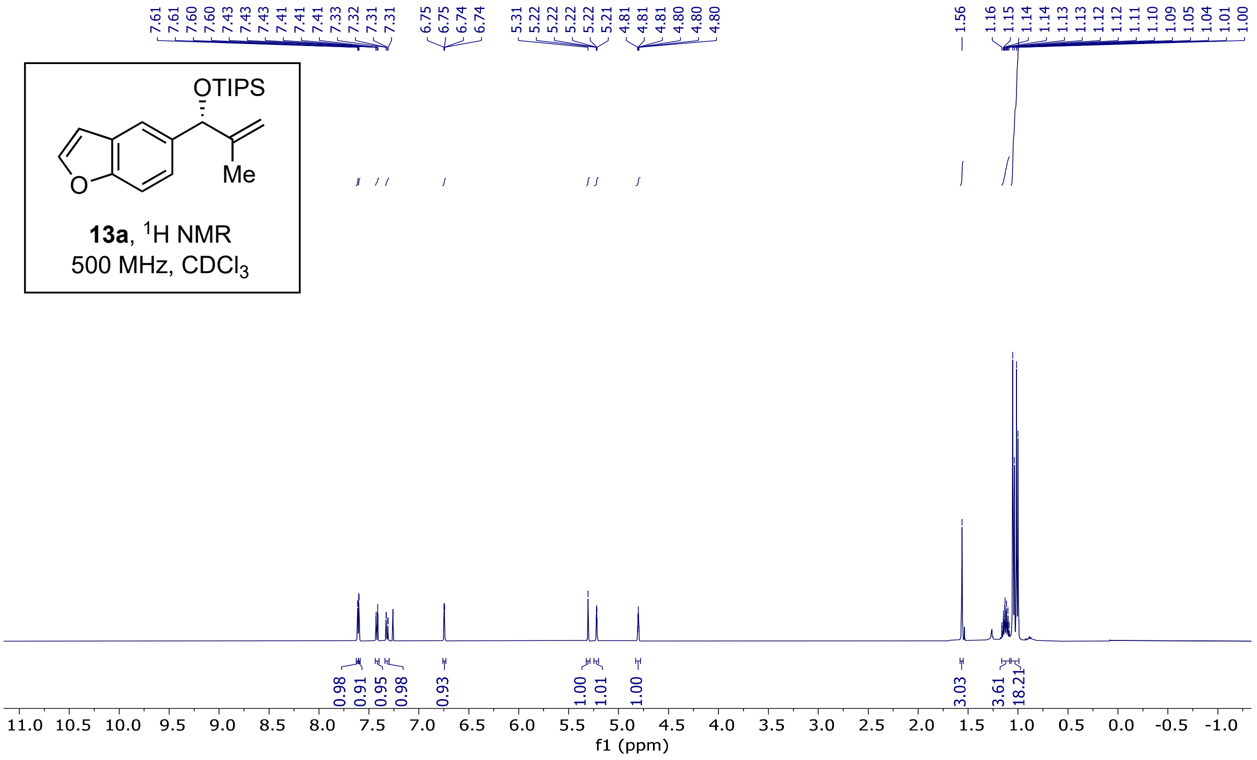
OTIPS
Me
O
13a, 1H NMR
500 MHz, CDCl3
f1 (ppm)

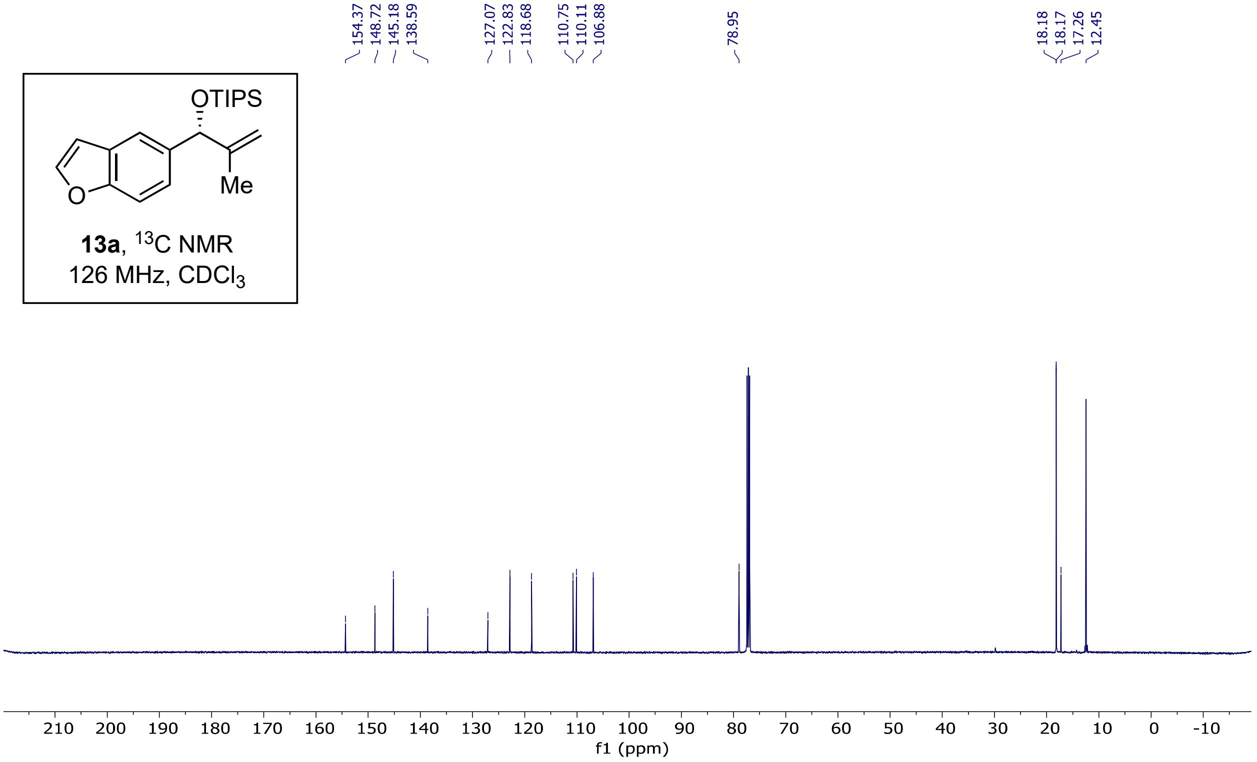
OTIPS
Me
O
13a, 13C NMR
126 MHz, CDCl3
f1 (ppm)

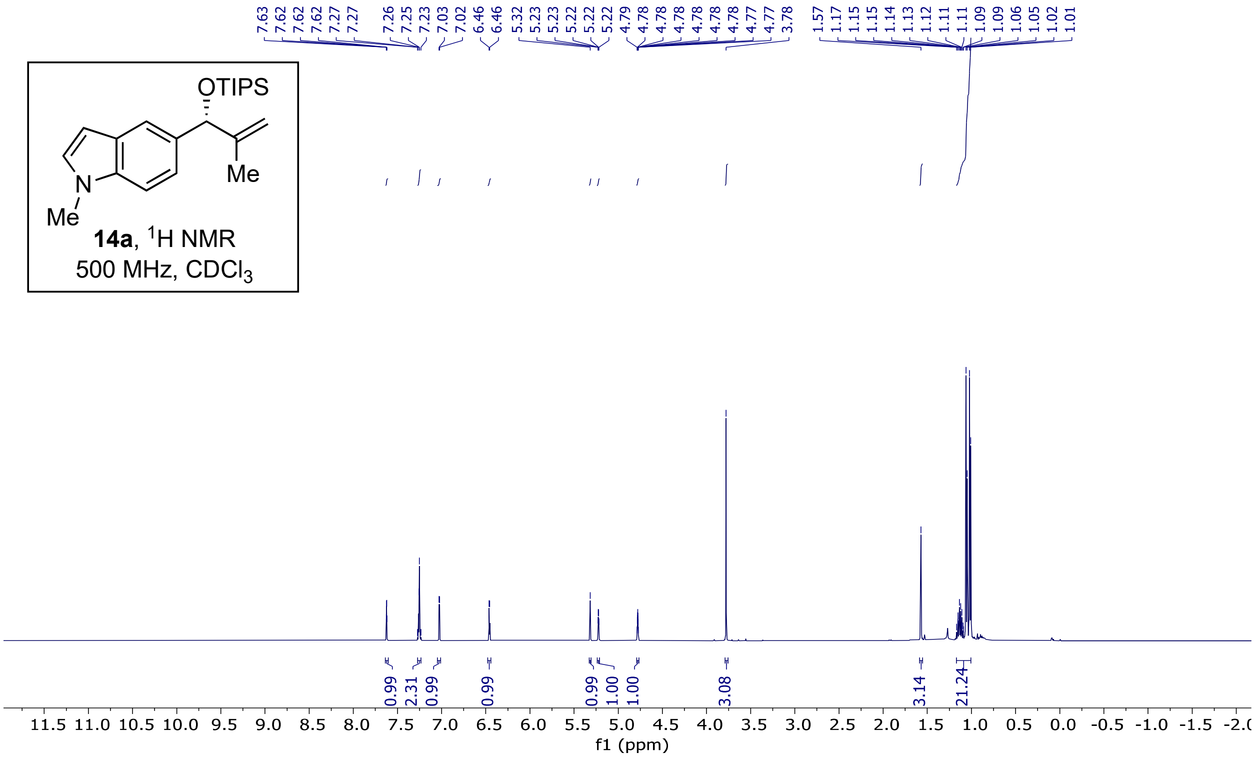
OTIPS
Me
N
Me
14a, ¹H NMR
500 MHz, CDCl₃
7.63 7.62 7.62 7.27 7.27 7.26 7.25 7.23 7.03 7.02 6.46 6.46 5.32 5.23 5.23 5.22 5.22 5.22 4.79 4.78 4.78 4.78 4.78 4.78 4.78 4.77 4.77 3.78 1.57 1.17 1.15 1.15 1.14 1.13 1.12 1.11 1.11 1.09 1.09 1.06 1.05 1.02 1.01
0.99 2.31 0.99 0.99 0.99 1.00 1.00 3.08 3.14 21.24
f1 (ppm)

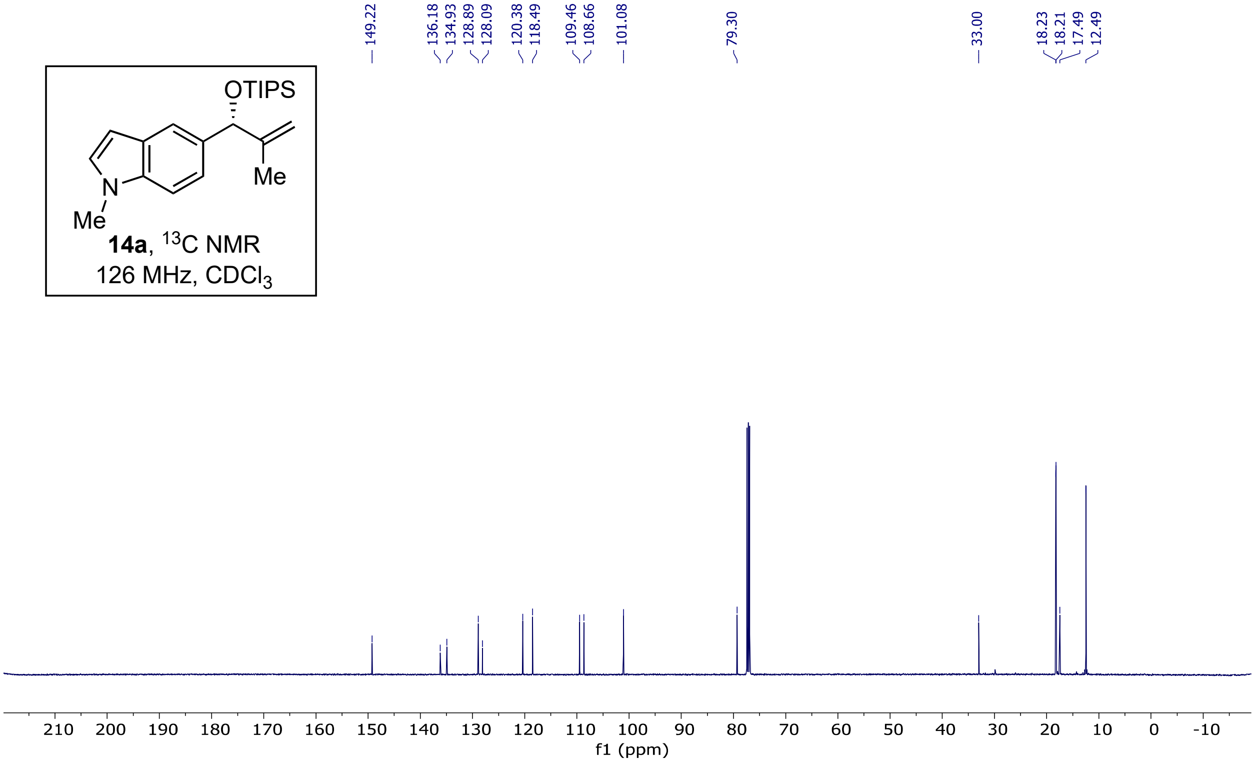
OTIPS
Me
N
Me
14a, ¹³C NMR
126 MHz, CDCl₃
149.22 136.18 134.93 128.89 128.09 120.38 118.49 109.46 108.66 101.08 79.30 33.00 18.23 18.21 17.49 12.49
f1 (ppm)

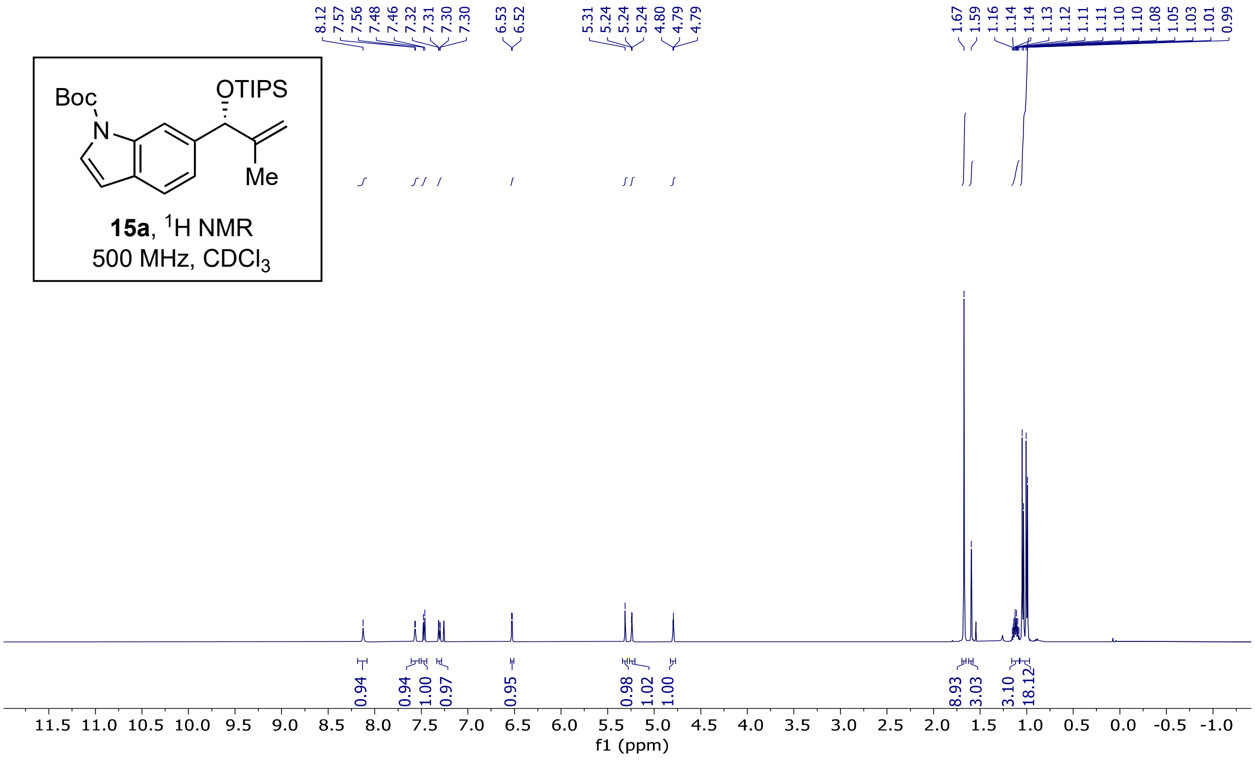
8.12 7.57 7.56 7.48 7.46 7.32 7.31 7.30 7.30 6.53 6.52 5.31 5.24 5.24 5.24 4.80 4.79 4.79 1.67 1.59 1.16 1.14 1.14 1.13 1.12 1.11 1.11 1.10 1.10 1.08 1.05 1.03 1.01 0.99
Boc
N
OTIPS
Me
15a, 1H NMR
500 MHz, CDCl3
0.94 0.94 1.00 0.97 0.95 0.98 1.02 1.00 8.93 3.03 3.10 18.12
11.5 11.0 10.5 10.0 9.5 9.0 8.5 8.0 7.5 7.0 6.5 6.0 5.5 5.0 4.5 4.0 3.5 3.0 2.5 2.0 1.5 1.0 0.5 0.0 -0.5 -1.0
f1 (ppm)

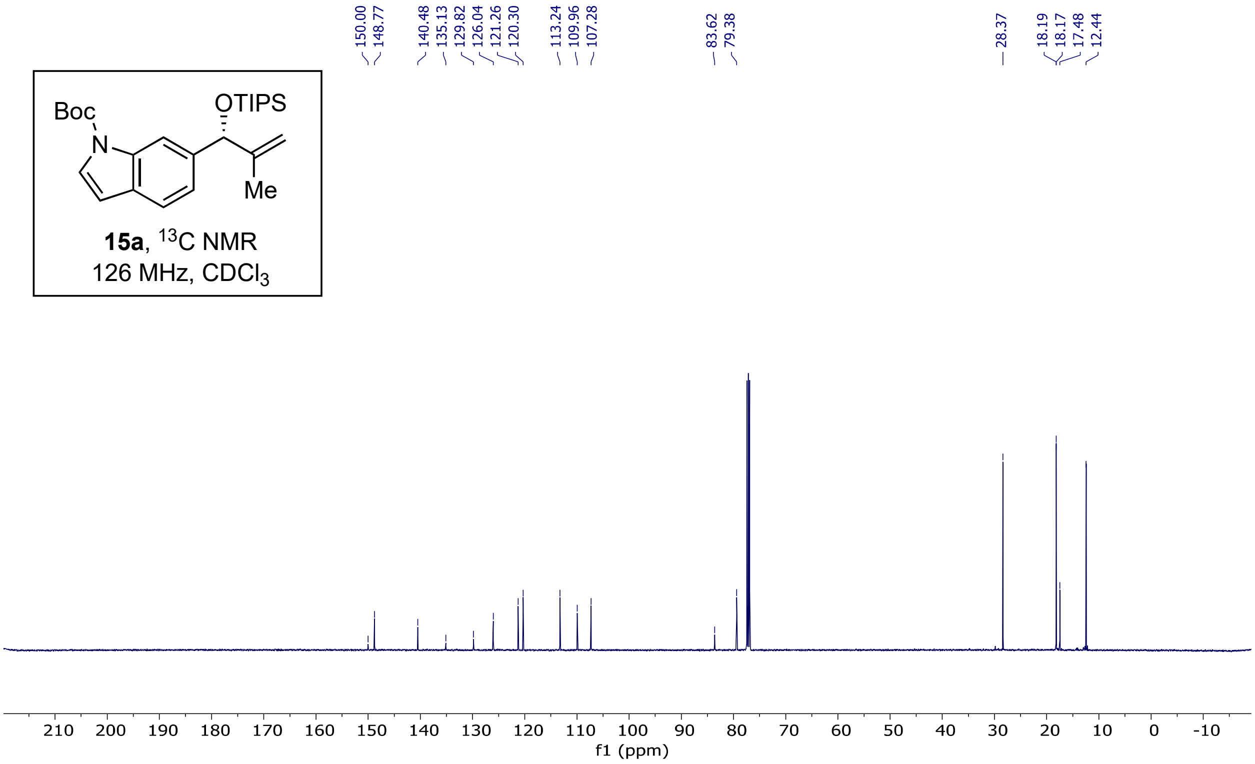
150.00 148.77 140.48 135.13 129.82 126.04 121.26 120.30 113.24 109.96 107.28 83.62 79.38 28.37 18.19 18.17 17.48 12.44
Boc
N
OTIPS
Me
15a, 13C NMR
126 MHz, CDCl3
210 200 190 180 170 160 150 140 130 120 110 100 90 80 70 60 50 40 30 20 10 0 -10
f1 (ppm)

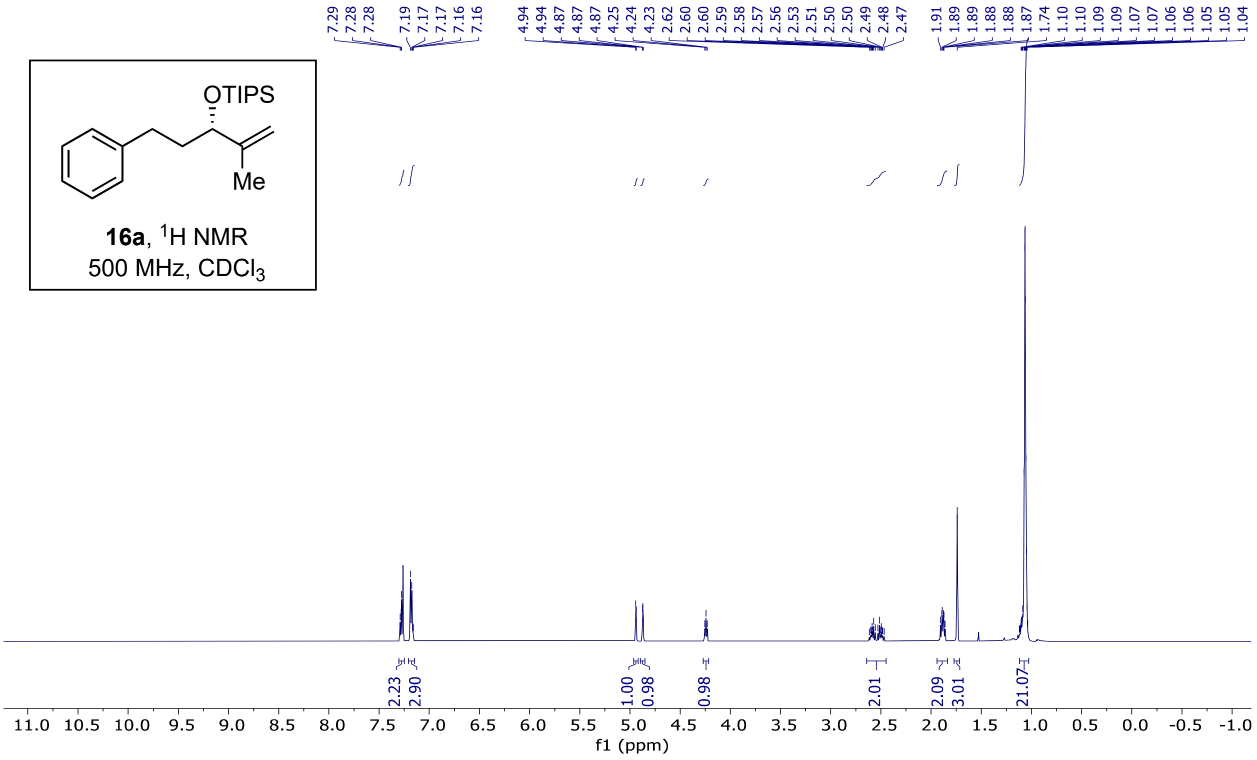
OTIPS
Me
16a, 1H NMR
500 MHz, CDCl3
f1 (ppm)

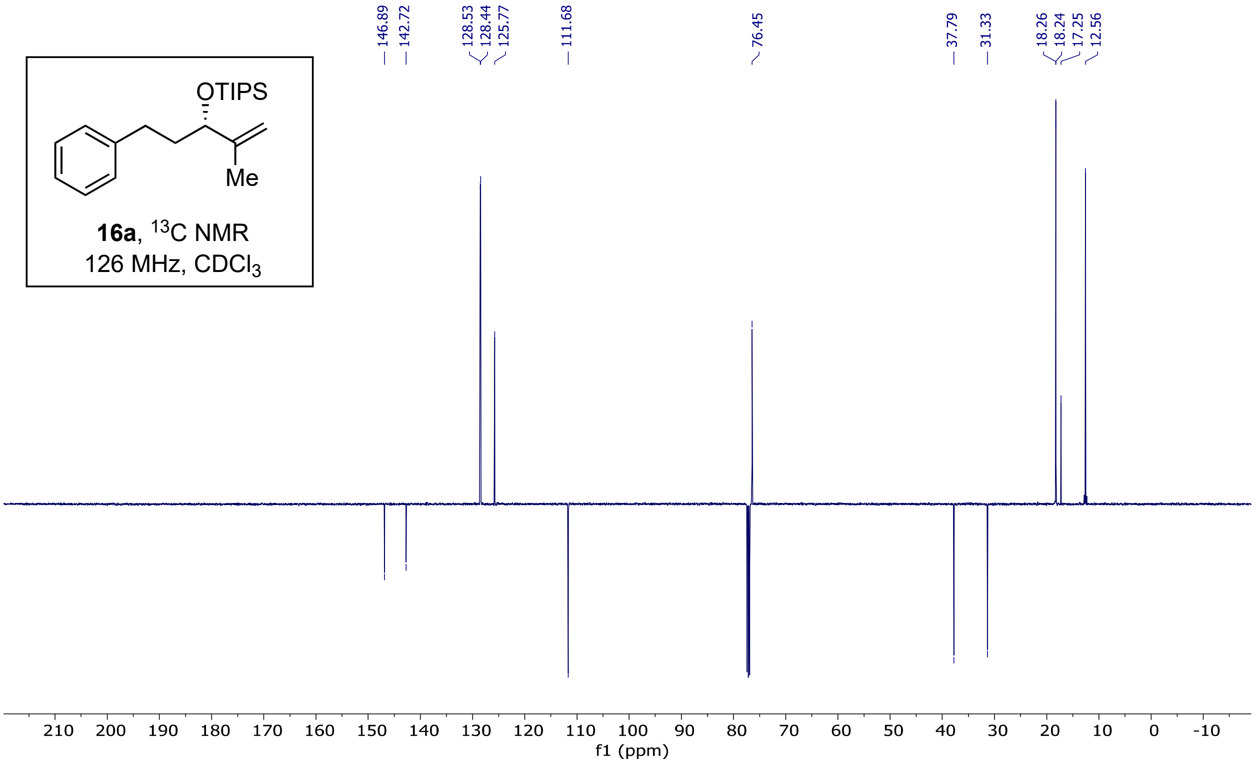
OTIPS
Me
16a, 13C NMR
126 MHz, CDCl3
f1 (ppm)

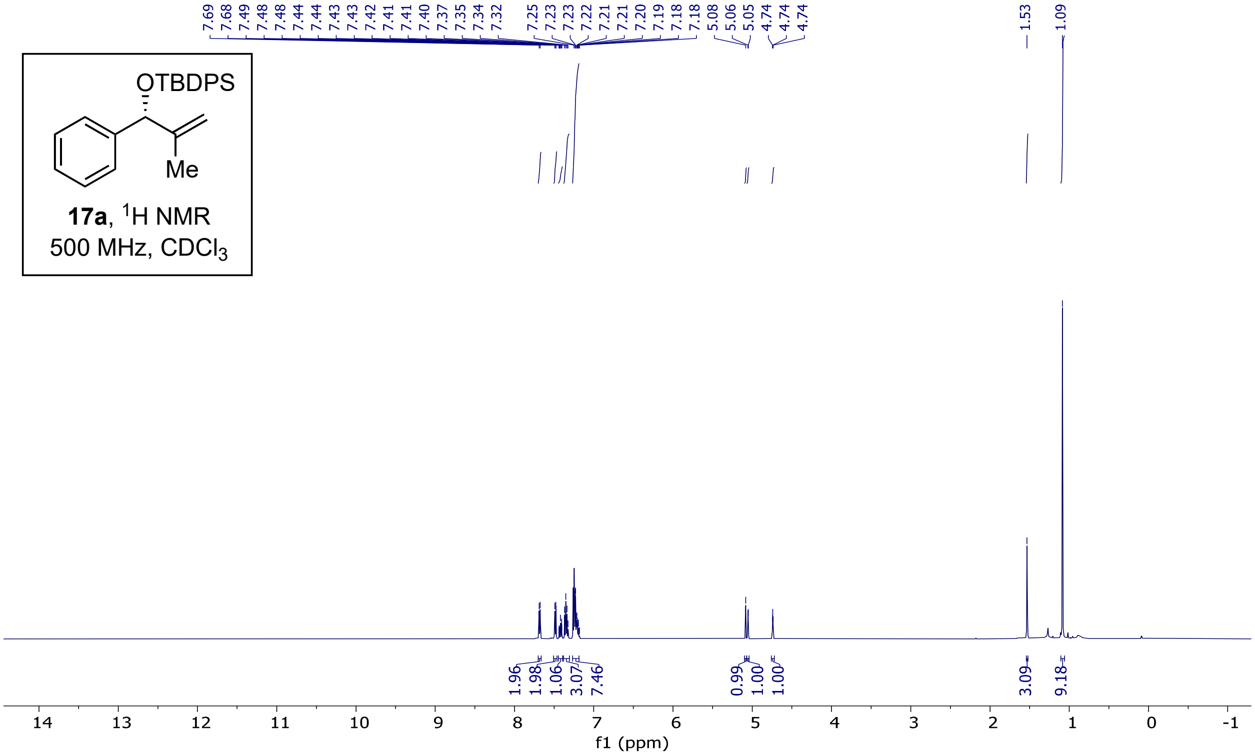
7.69 7.68 7.49 7.48 7.48 7.44 7.44 7.43 7.43 7.42 7.41 7.41 7.40 7.37 7.35 7.34 7.32 7.25 7.23 7.23 7.22 7.21 7.21 7.20 7.19 7.18 7.18 5.08 5.06 5.05 4.74 4.74 4.74
1.53
1.09
OTBDPS
Me
17a, 1H NMR
500 MHz, CDCl3
1.96 1.98 1.06 3.07 7.46
0.99 1.00 1.00
3.09
9.18
14 13 12 11 10 9 8 7 6 5 4 3 2 1 0 -1
f1 (ppm)

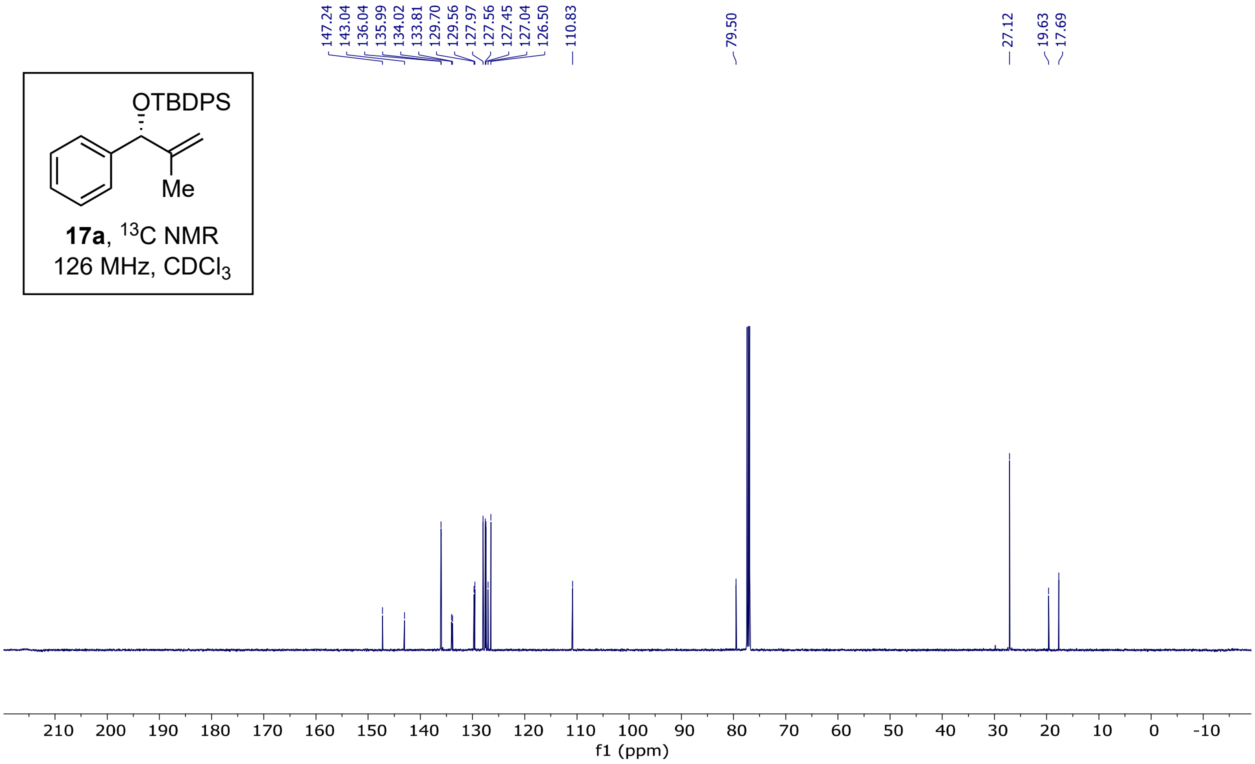
147.24 143.04 136.04 135.99 134.02 133.81 129.70 129.56 127.97 127.56 127.45 127.04 126.50
110.83
79.50
27.12
19.63 17.69
OTBDPS
Me
17a, 13C NMR
126 MHz, CDCl3
210 200 190 180 170 160 150 140 130 120 110 100 90 80 70 60 50 40 30 20 10 0 -10
f1 (ppm)

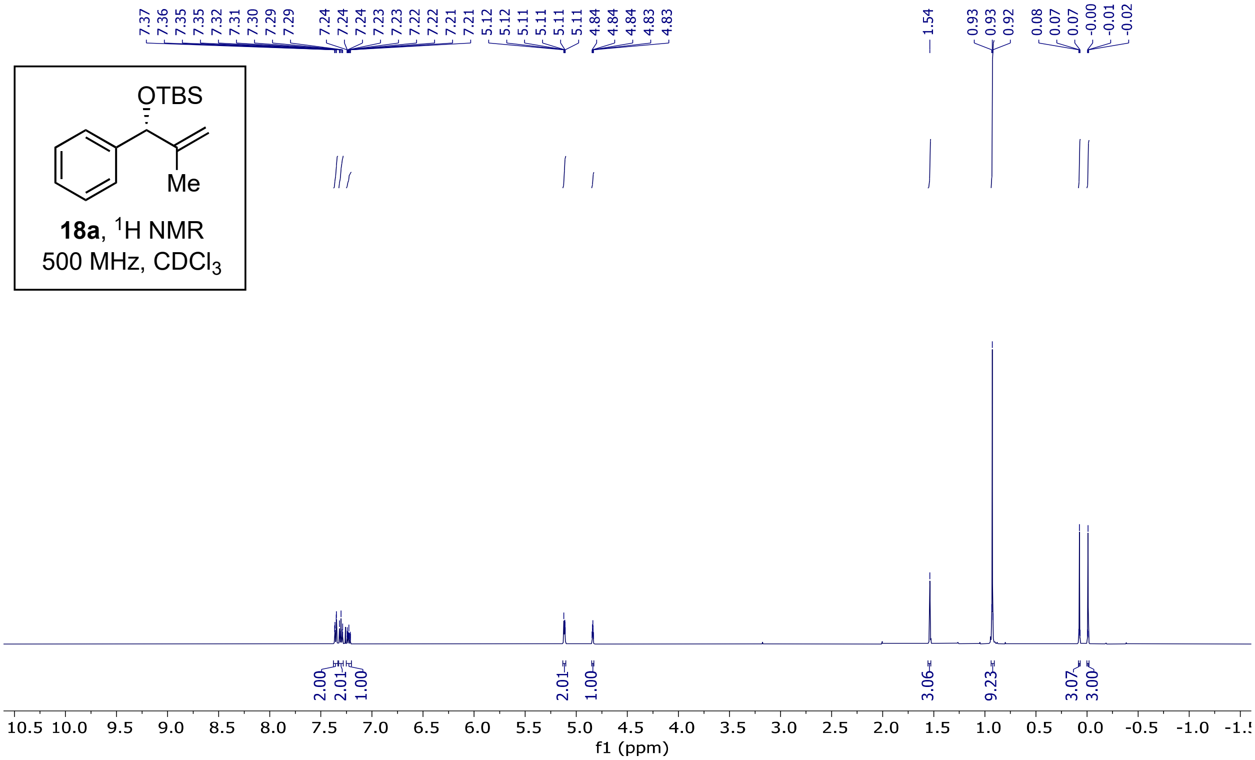

OTBS
Me
18a, 1H NMR
500 MHz, CDCl3
7.37 7.36 7.35 7.35 7.32 7.31 7.30 7.29 7.29 7.24 7.24 7.24 7.23 7.23 7.22 7.22 7.21 7.21 5.12 5.12 5.11 5.11 5.11 5.11 4.84 4.84 4.84 4.83 4.83
1.54
0.93 0.93 0.92
0.08 0.07 0.07 -0.00 -0.01 -0.02
2.00 2.01 1.00
2.01 1.00
3.06
9.23
3.07 3.00
f1 (ppm)

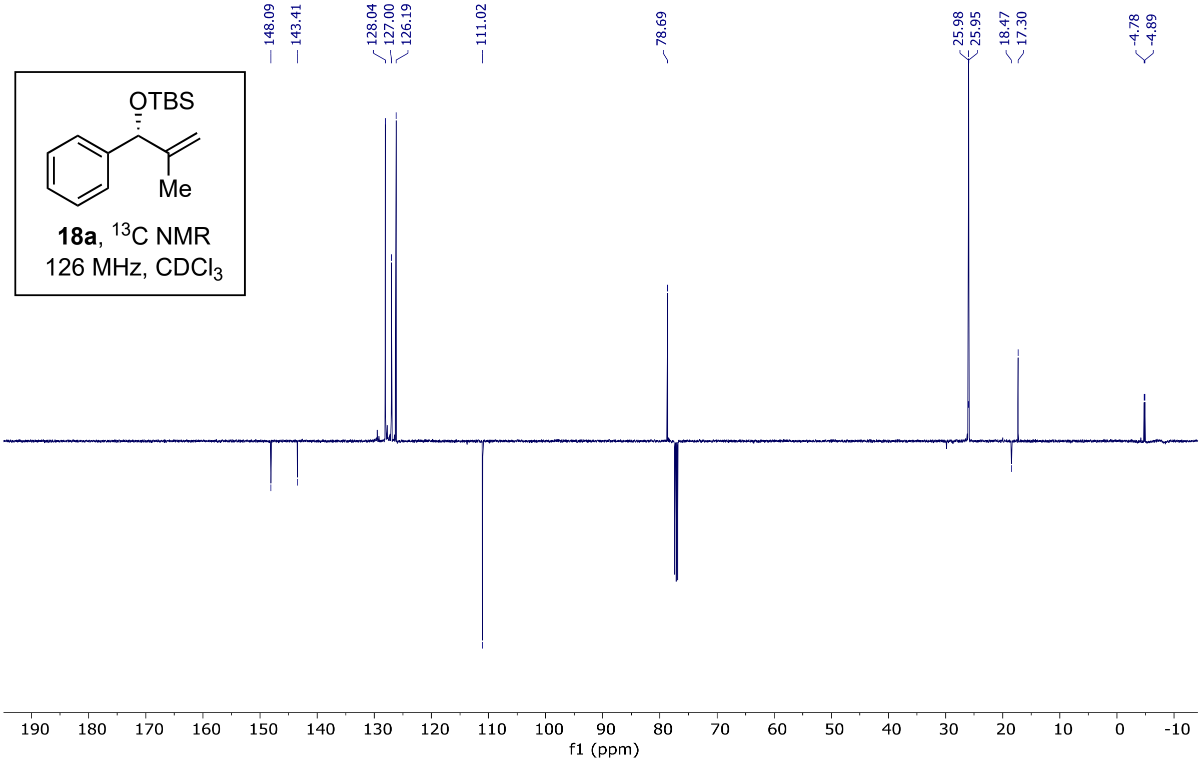

OTBS
Me
18a, 13C NMR
126 MHz, CDCl3
148.09
143.41
128.04 127.00 126.19
111.02
78.69
25.98 25.95
18.47 17.30
-4.78 -4.89
f1 (ppm)

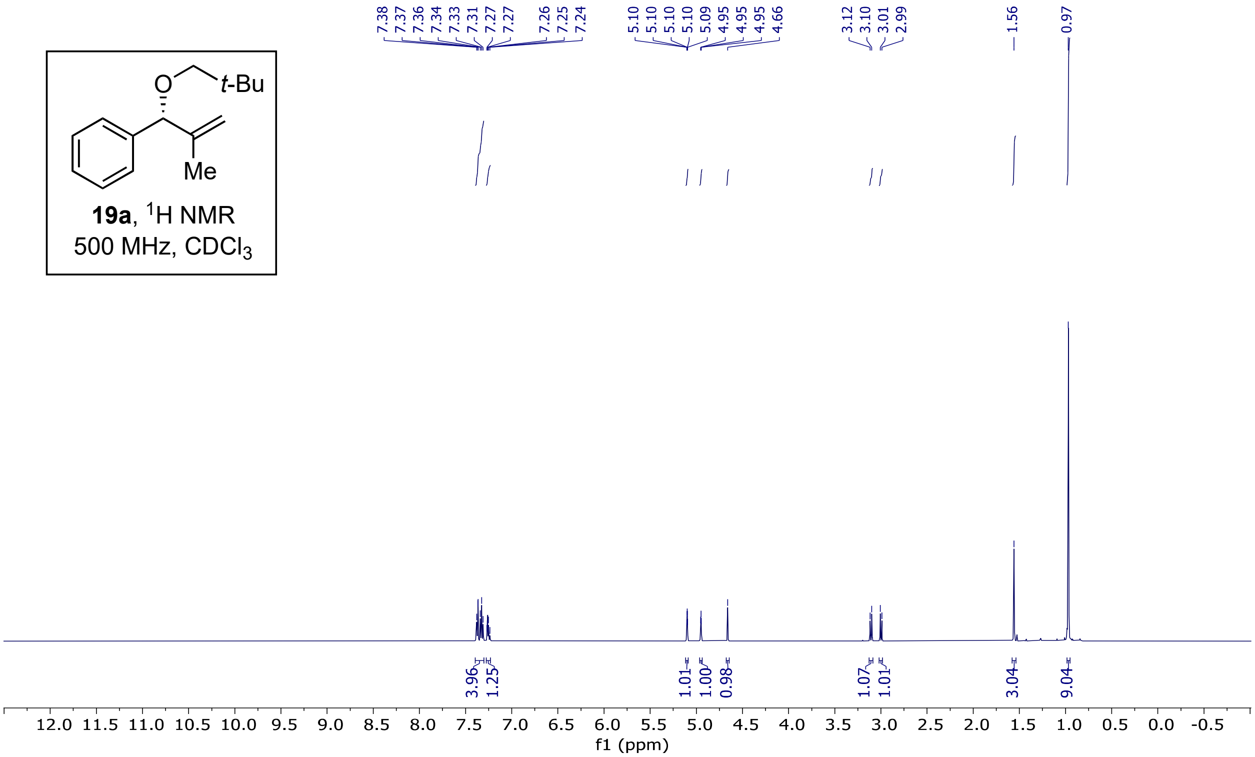
t-Bu
O
Me
19a, 1H NMR
500 MHz, CDCl3
7.38 7.37 7.36 7.34 7.33 7.31 7.27 7.27 7.27 7.26 7.25 7.24
5.10 5.10 5.10 5.10 5.09 4.95 4.95 4.95 4.95 4.66
3.12 3.10 3.01 2.99
1.56
0.97
3.96 1.25 1.01 1.00 0.98 1.07 1.01 3.04 9.04
f1 (ppm)

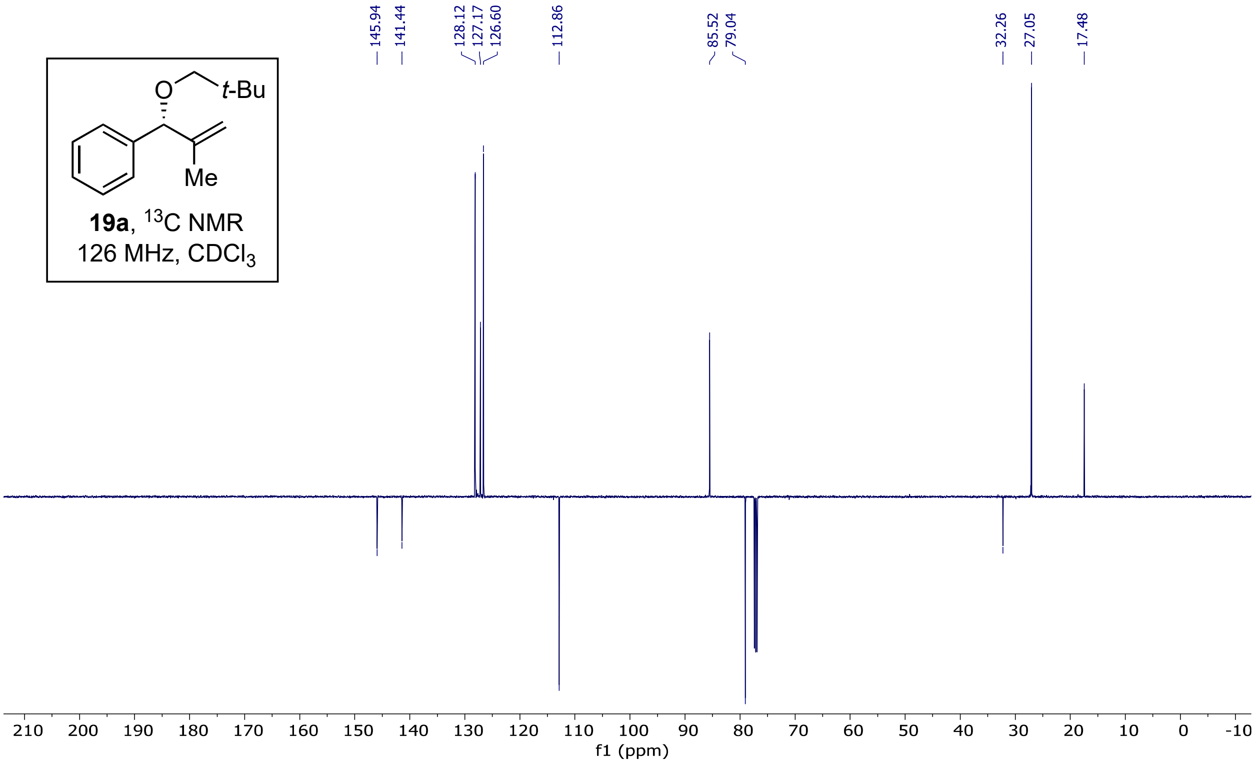
t-Bu
O
Me
19a, 13C NMR
126 MHz, CDCl3
145.94 141.44 128.12 127.17 126.60 112.86 85.52 79.04 32.26 27.05 17.48
f1 (ppm)

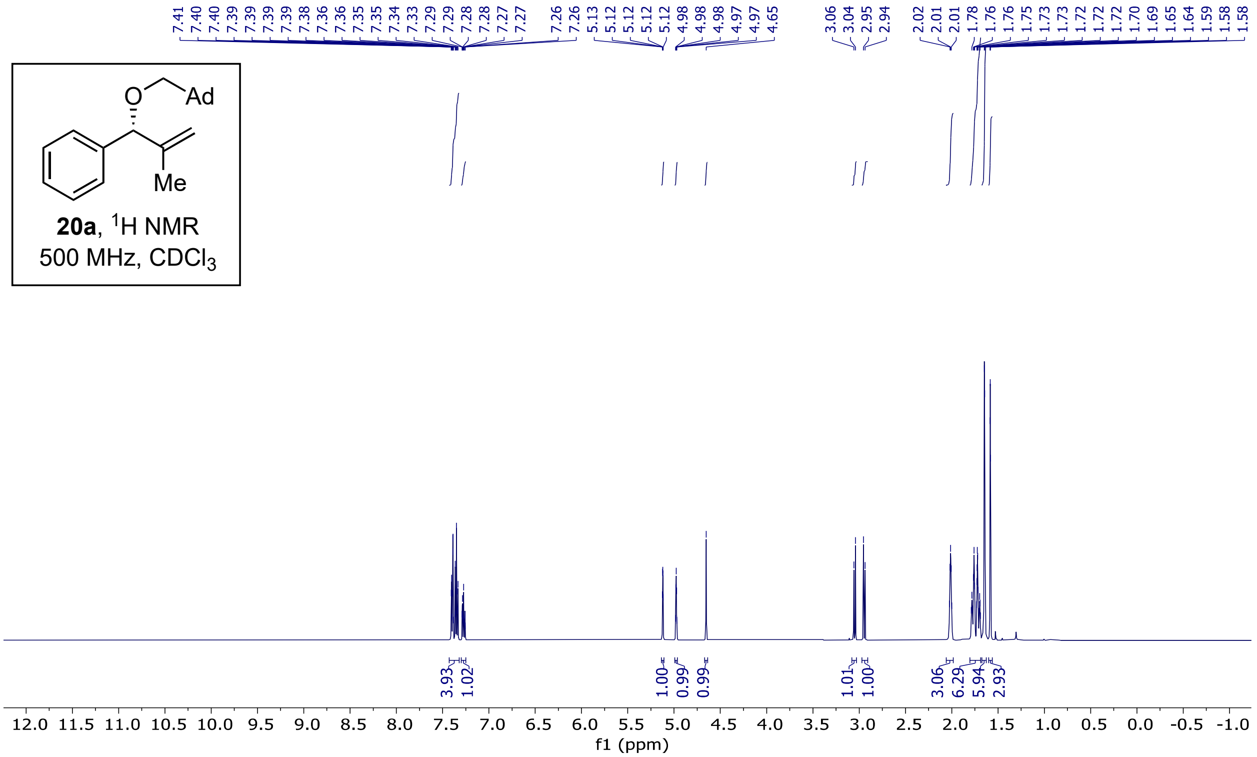
Ad
O
Me
20a, 1H NMR
500 MHz, CDCl3
7.41 7.40 7.40 7.39 7.39 7.39 7.39 7.38 7.36 7.36 7.35 7.35 7.34 7.33 7.29 7.29 7.28 7.28 7.27 7.27 7.26 7.26 5.13 5.12 5.12 5.12 5.12 4.98 4.98 4.98 4.97 4.97 4.65 3.06 3.04 2.95 2.94 2.02 2.01 2.01 1.78 1.76 1.76 1.75 1.73 1.73 1.72 1.72 1.70 1.69 1.65 1.64 1.59 1.58 1.58
3.93 1.02 1.00 0.99 0.99 1.01 1.00 3.06 6.29 5.94 2.93
f1 (ppm)

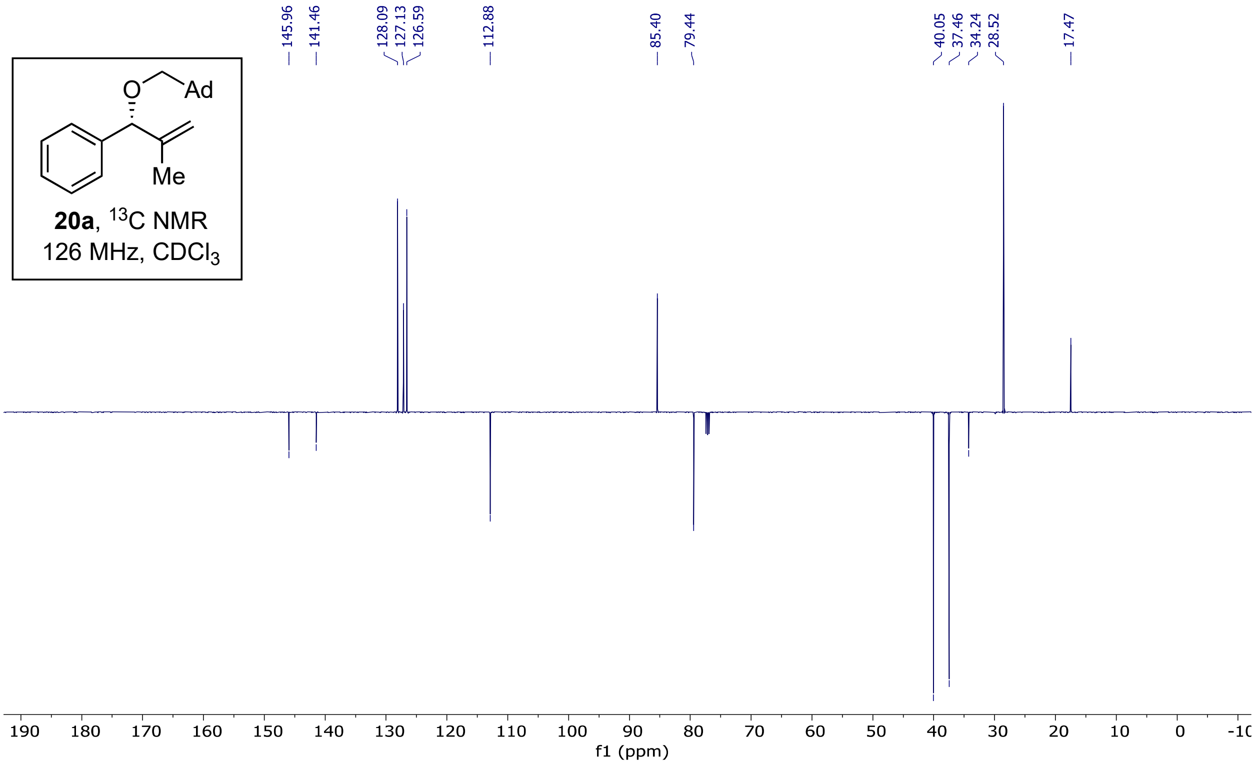
Ad
O
Me
20a, 13C NMR
126 MHz, CDCl3
145.96 141.46 128.09 127.13 126.59 112.88 85.40 79.44 40.05 37.46 34.24 28.52 17.47
f1 (ppm)

12.HPLC Traces

Desilylation procedure for ee determination of silyl ethers (1a-10a, 12a-18a)

A 2-dram vial equipped with a magnetic stir bar was charged with the corresponding silyl ether (~10 mg) is dissolved in THF (1 mL), tetrabutylammonium fluoride (1M in THF, 0.2 mL) was added. The resulting solution was stirred at room temperature for 1 h and then concentrated under reduced pressure. The crude mixture was diluted with 10% *i*-PrOH/hexanes and filtered through a pipette silica plug eluting with 10% *i*-PrOH/hexanes. The resulting solution was analyzed using HPLC.

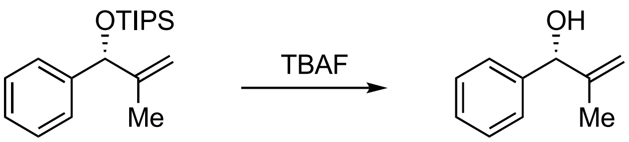

1a

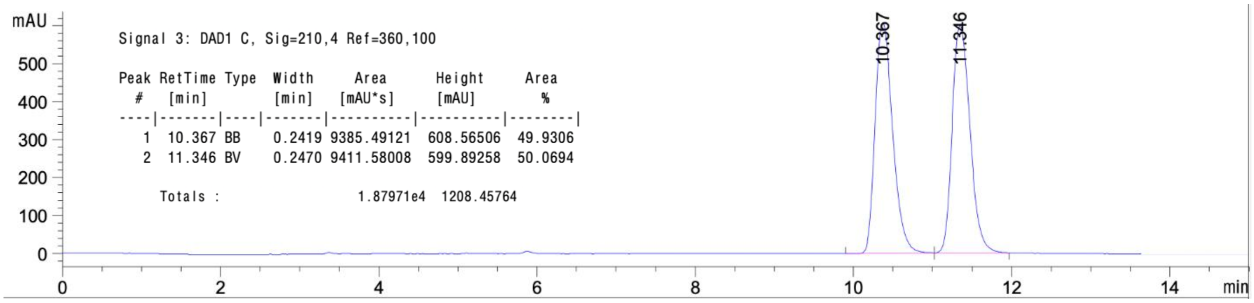

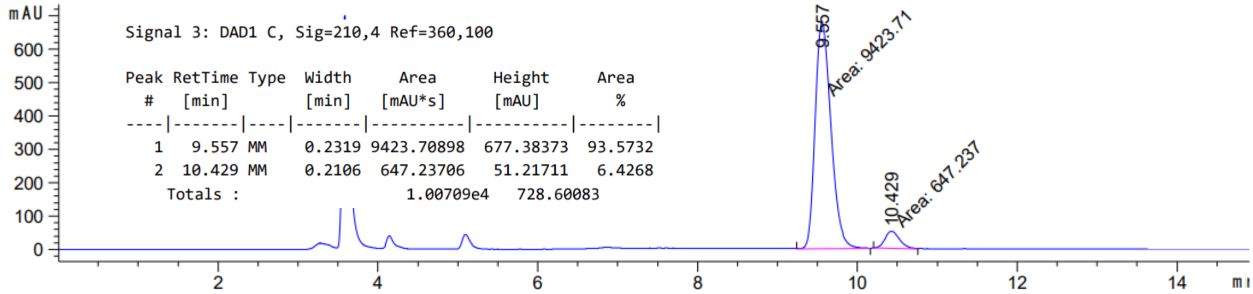

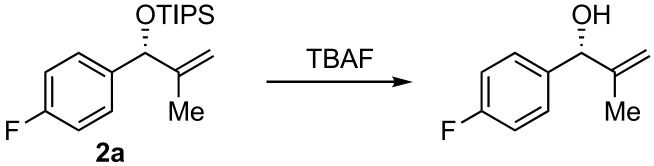

2a

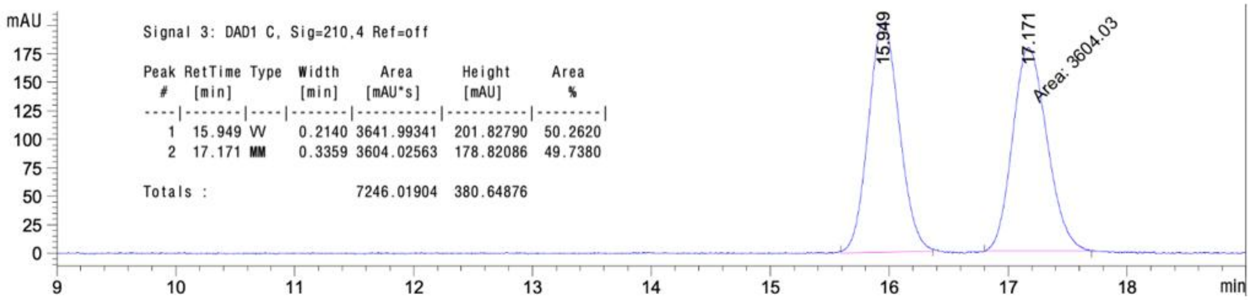

Peak #	RetTime [min]	Type	Width [min]	Area [mAU*s]	Height [mAU]	Area %
1	15.949	VV	0.2140	3641.99341	201.82790	50.2620
2	17.171	MM	0.3359	3604.02563	178.82086	49.7380
Totals :				7246.01904	380.64876	

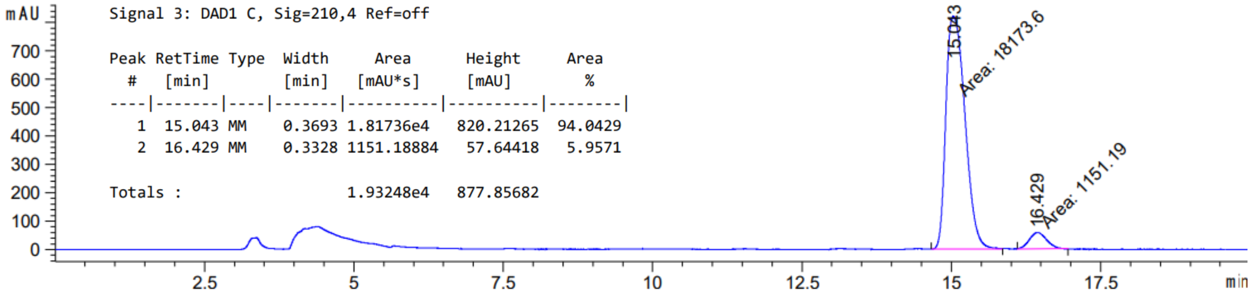

Peak #	RetTime [min]	Type	Width [min]	Area [mAU*s]	Height [mAU]	Area %
1	15.043	MM	0.3693	1.81736e4	820.21265	94.0429
2	16.429	MM	0.3328	1151.18884	57.64418	5.9571
Totals :				1.93248e4	877.85682	

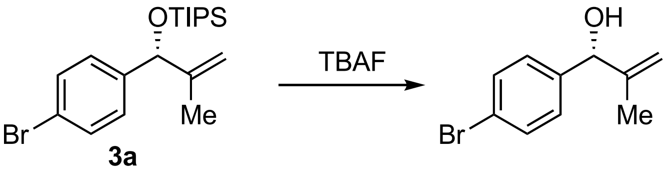

3a

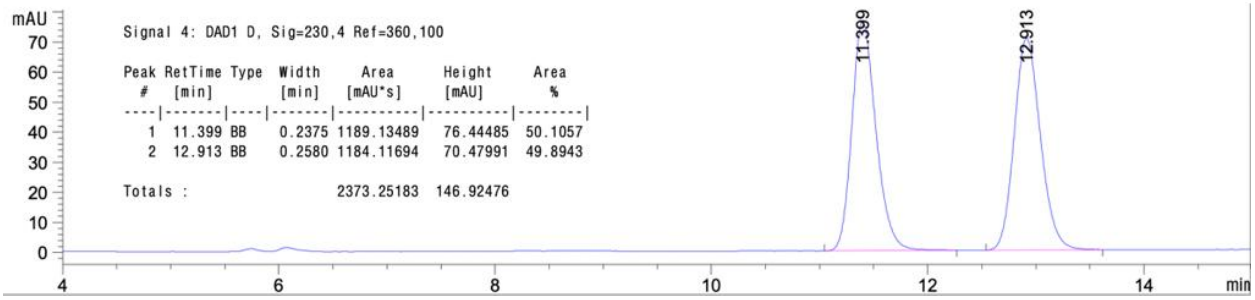

Peak #	RetTime [min]	Type	Width [min]	Area [mAU*s]	Height [mAU]	Area %
1	11.399	BB	0.2375	1189.13489	76.44485	50.1057
2	12.913	BB	0.2580	1184.11694	70.47991	49.8943
Totals :				2373.25183	146.92476	

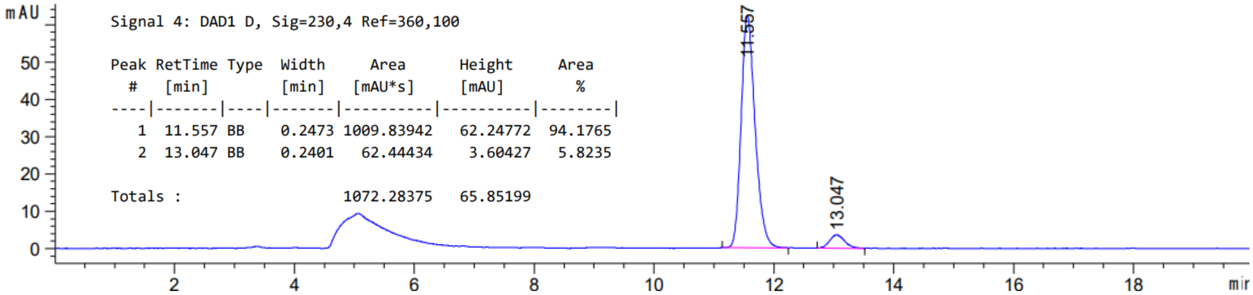

Peak #	RetTime [min]	Type	Width [min]	Area [mAU*s]	Height [mAU]	Area %
1	11.557	BB	0.2473	1009.83942	62.24772	94.1765
2	13.047	BB	0.2401	62.44434	3.60427	5.8235
Totals :				1072.28375	65.85199	

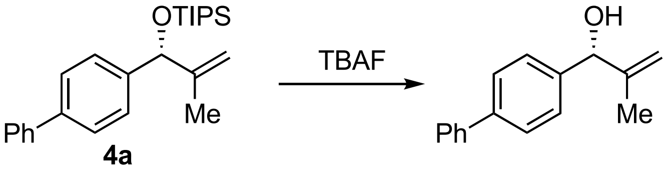
OTIPS
TBAF
OH
Ph
Me
Ph
Me

4a

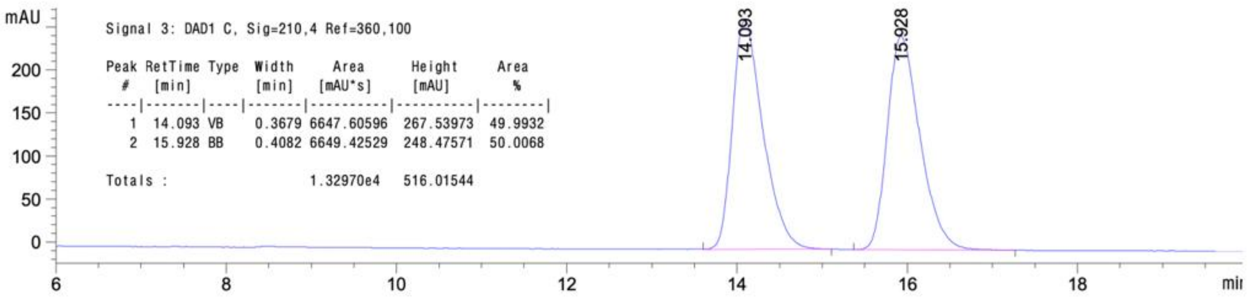
Signal 3: DAD1 C, Sig=210,4 Ref=360,100
Peak RetTime Type Width Area Height Area
[min] [min] [mAU*s] [mAU] %
1 14.093 VB 0.3679 6647.60596 267.53973 49.9932
2 15.928 BB 0.4082 6649.42529 248.47571 50.0068
Totals : 1.32970e4 516.01544
14.093
15.928
mAU
min

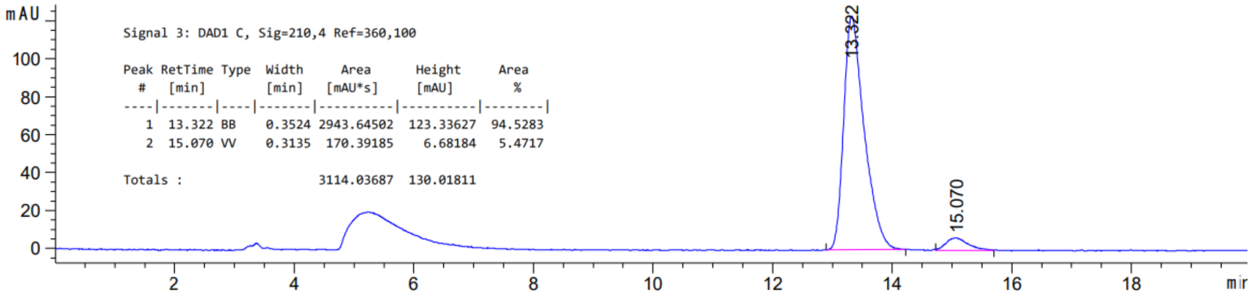
Signal 3: DAD1 C, Sig=210,4 Ref=360,100
Peak RetTime Type Width Area Height Area
[min] [min] [mAU*s] [mAU] %
1 13.322 BB 0.3524 2943.64502 123.33627 94.5283
2 15.070 VV 0.3135 170.39185 6.68184 5.4717
Totals : 3114.03687 130.01811
13.322
15.070
mAU

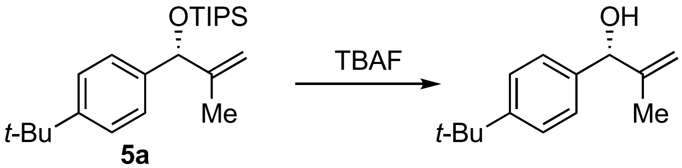
OTIPS
TBAF
OH
t-Bu
Me
t-Bu
Me

5a

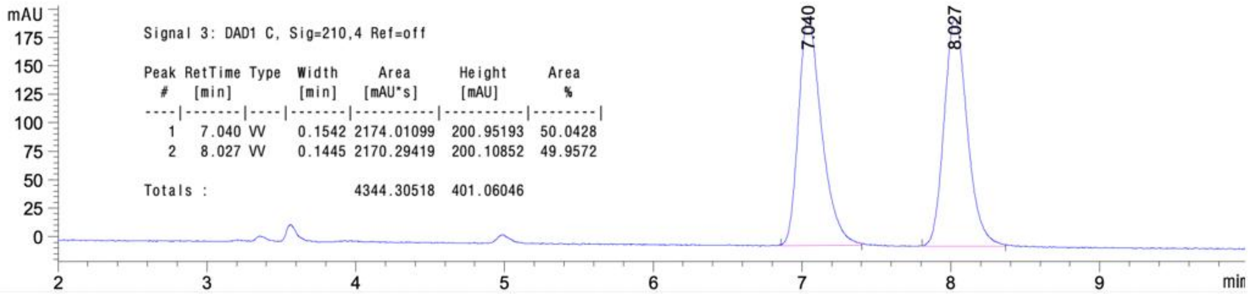
Signal 3: DAD1 C, Sig=210,4 Ref=off
Peak RetTime Type Width Area Height Area
[min] [min] [mAU*s] [mAU] %
1 7.040 VV 0.1542 2174.01099 200.95193 50.0428
2 8.027 VV 0.1445 2170.29419 200.10852 49.9572
Totals : 4344.30518 401.06046
7.040
8.027
mAU
min

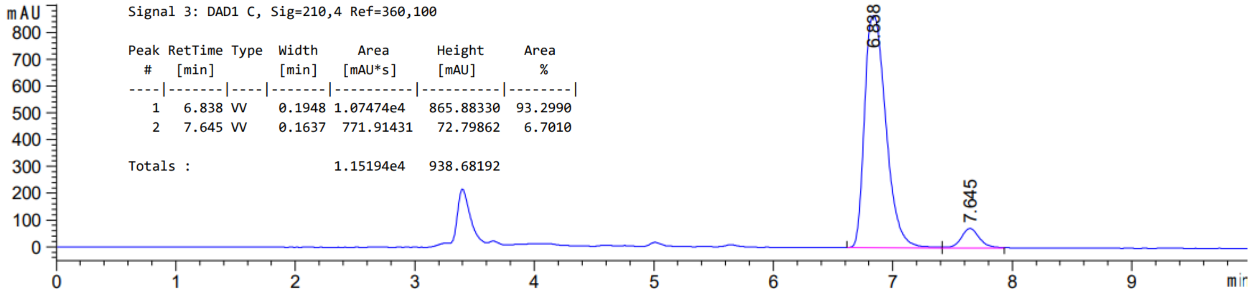
Signal 3: DAD1 C, Sig=210,4 Ref=360,100
Peak RetTime Type Width Area Height Area
[min] [min] [mAU*s] [mAU] %
1 6.838 VV 0.1948 1.07474e4 865.88330 93.2990
2 7.645 VV 0.1637 771.91431 72.79862 6.7010
Totals : 1.15194e4 938.68192
6.838
7.645
mAU

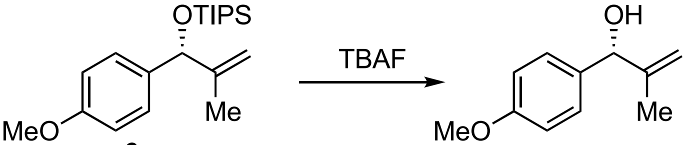
OTIPS
MeO
Me
TBAF
OH
MeO
Me

6a

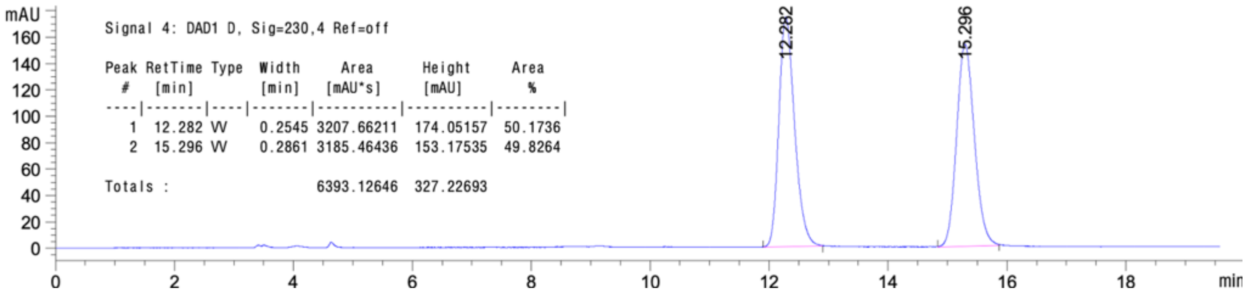
Signal 4: DAD1 D, Sig=230,4 Ref=off
Peak RetTime Type Width Area Height Area
[min] [min] [mAU*s] [mAU] %
1 12.282 VV 0.2545 3207.66211 174.05157 50.1736
2 15.296 VV 0.2861 3185.46436 153.17535 49.8264
Totals : 6393.12646 327.22693
12.282
15.296
mAU
min

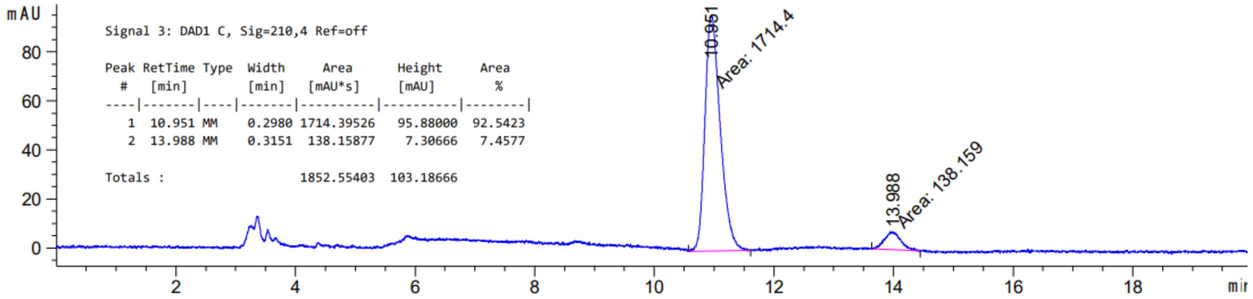
Signal 3: DAD1 C, Sig=210,4 Ref=off
Peak RetTime Type Width Area Height Area
[min] [min] [mAU*s] [mAU] %
1 10.951 MM 0.2980 1714.39526 95.88000 92.5423
2 13.988 MM 0.3151 138.15877 7.30666 7.4577
Totals : 1852.55403 103.18666
10.951
Area: 1714.4
13.988
Area: 138.159
mAU
min

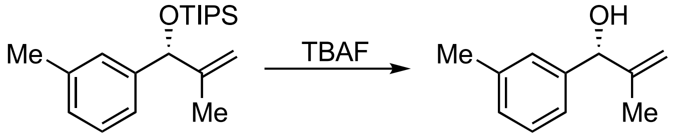
OTIPS
Me
Me
TBAF
OH
Me
Me

7a

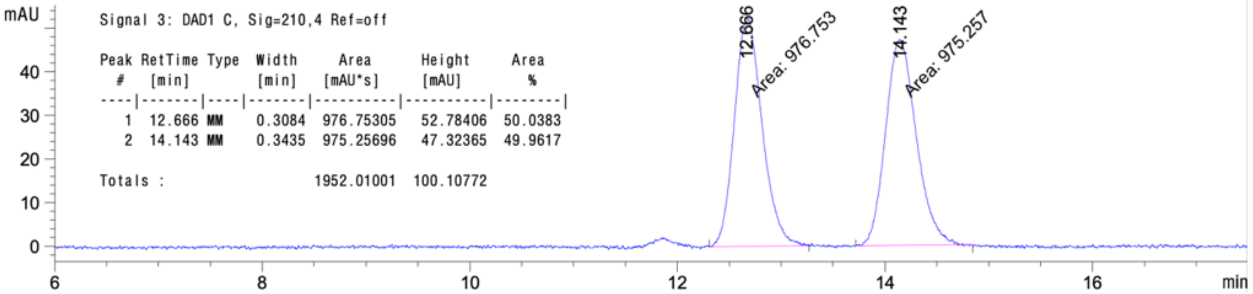
Signal 3: DAD1 C, Sig=210,4 Ref=off
Peak RetTime Type Width Area Height Area
[min] [min] [mAU*s] [mAU] %
1 12.666 MM 0.3084 976.75305 52.78406 50.0383
2 14.143 MM 0.3435 975.25696 47.32365 49.9617
Totals : 1952.01001 100.10772
12.666
Area: 976.753
14.143
Area: 975.257
mAU
min

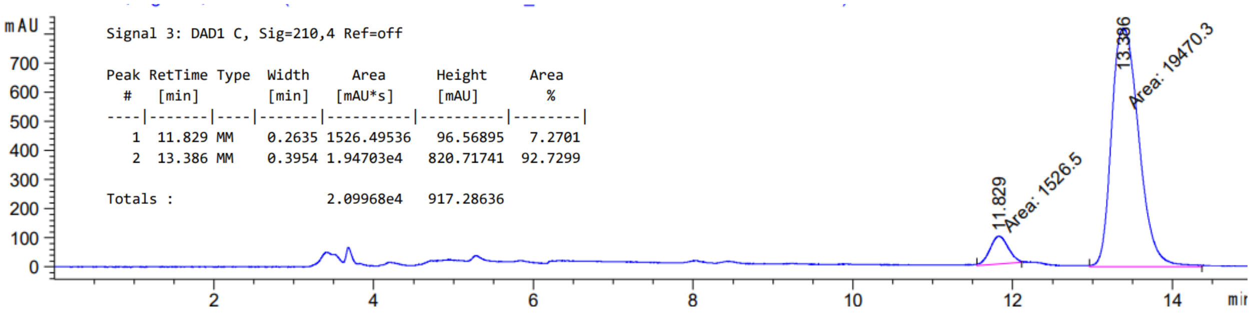
Signal 3: DAD1 C, Sig=210,4 Ref=off
Peak RetTime Type Width Area Height Area
[min] [min] [mAU*s] [mAU] %
1 11.829 MM 0.2635 1526.49536 96.56895 7.2701
2 13.386 MM 0.3954 1.94703e4 820.71741 92.7299
Totals : 2.09968e4 917.28636
11.829
Area: 1526.5
13.386
Area: 19470.3
mAU
min

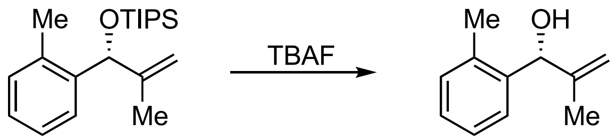

8a

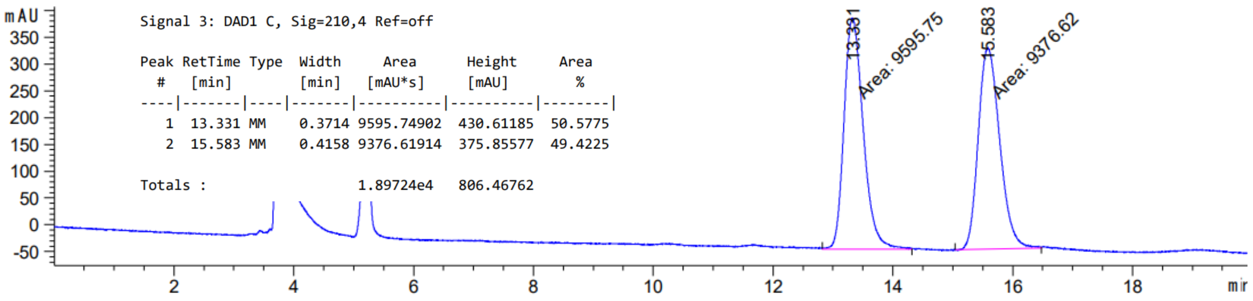

Signal 3: DAD1 C, Sig=210,4 Ref=off

Peak #	RetTime [min]	Type	Width [min]	Area [mAU*s]	Height [mAU]	Area %
1	13.331	MM	0.3714	9595.74902	430.61185	50.5775
2	15.583	MM	0.4158	9376.61914	375.85577	49.4225
Totals :				1.89724e4	806.46762	

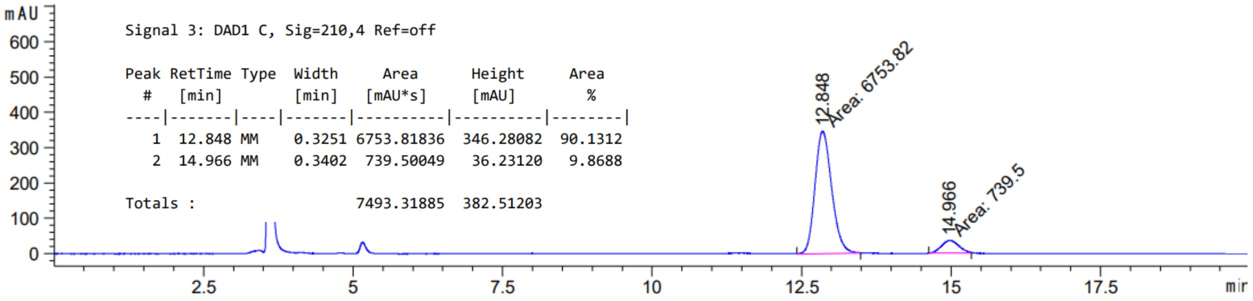

Signal 3: DAD1 C, Sig=210,4 Ref=off

Peak #	RetTime [min]	Type	Width [min]	Area [mAU*s]	Height [mAU]	Area %
1	12.848	MM	0.3251	6753.81836	346.28082	90.1312
2	14.966	MM	0.3402	739.50049	36.23120	9.8688
Totals :				7493.31885	382.51203	

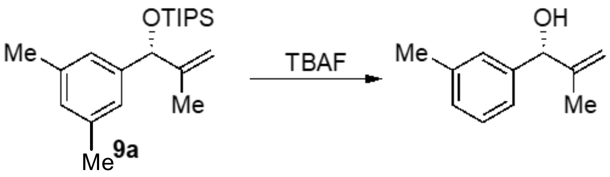

9a

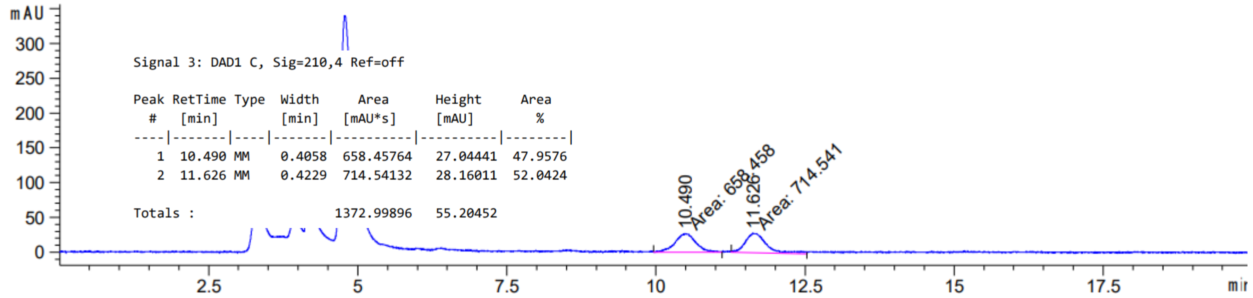

Signal 3: DAD1 C, Sig=210,4 Ref=off

Peak #	RetTime [min]	Type	Width [min]	Area [mAU*s]	Height [mAU]	Area %
1	10.490	MM	0.4058	658.45764	27.04441	47.9576
2	11.626	MM	0.4229	714.54132	28.16011	52.0424
Totals :				1372.99896	55.20452	

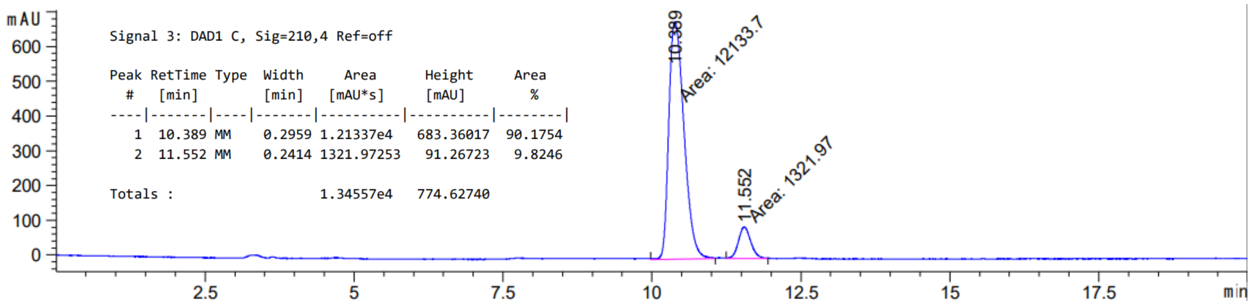

Signal 3: DAD1 C, Sig=210,4 Ref=off

Peak #	RetTime [min]	Type	Width [min]	Area [mAU*s]	Height [mAU]	Area %
1	10.389	MM	0.2959	1.21337e4	683.36017	90.1754
2	11.552	MM	0.2414	1321.97253	91.26723	9.8246
Totals :				1.34557e4	774.62740	

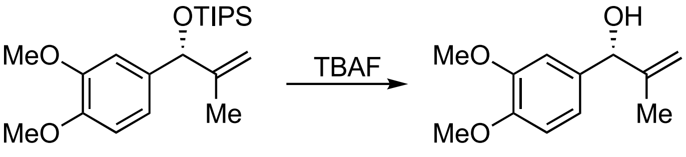

10a

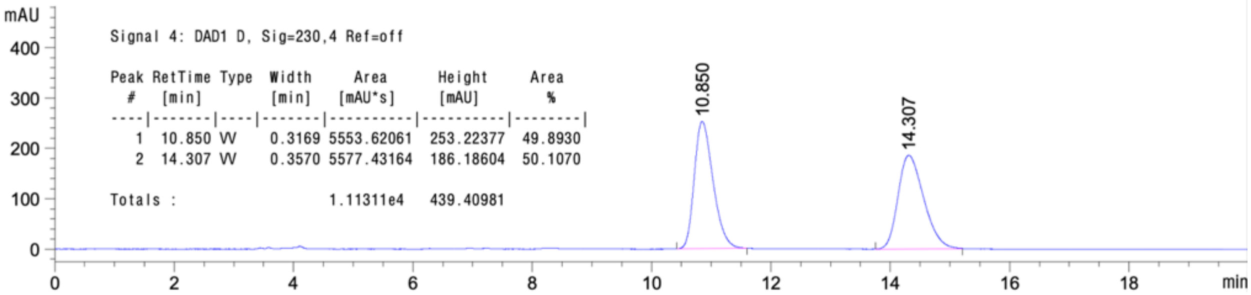

Signal 4: DAD1 D, Sig=230,4 Ref=off

Peak #	RetTime [min]	Type	Width [min]	Area [mAU*s]	Height [mAU]	Area %
1	10.850	VV	0.3169	5553.62061	253.22377	49.8930
2	14.307	VV	0.3570	5577.43164	186.18604	50.1070
Totals :				1.11311e4	439.40981	

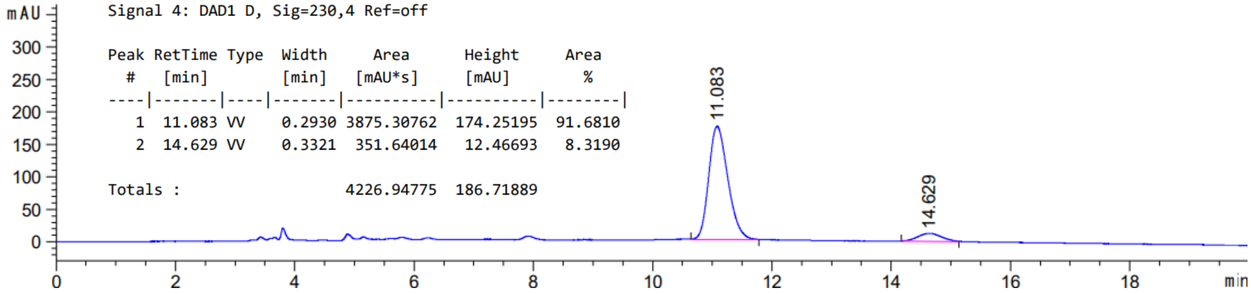

Signal 4: DAD1 D, Sig=230,4 Ref=off

Peak #	RetTime [min]	Type	Width [min]	Area [mAU*s]	Height [mAU]	Area %
1	11.083	VV	0.2930	3875.30762	174.25195	91.6810
2	14.629	VV	0.3321	351.64014	12.46693	8.3190
Totals :				4226.94775	186.71889	

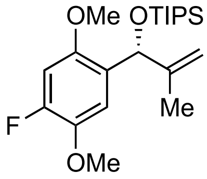

11a

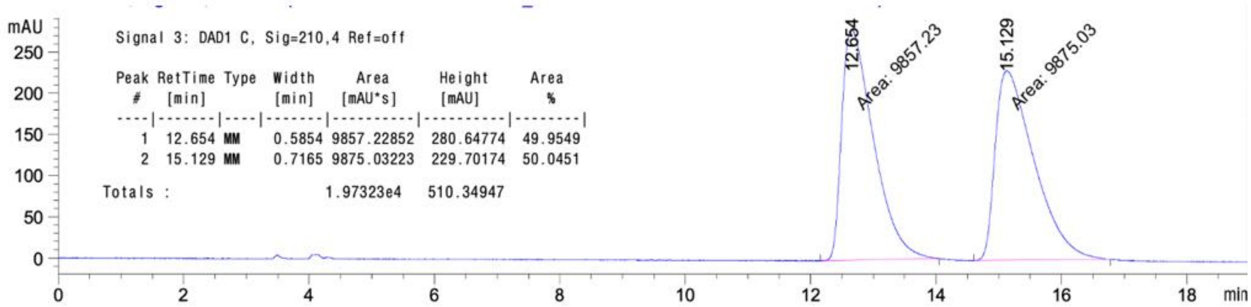

Signal 3: DAD1 C, Sig=210,4 Ref=off

Peak #	RetTime [min]	Type	Width [min]	Area [mAU*s]	Height [mAU]	Area %
1	12.654	MM	0.5854	9857.22852	280.64774	49.9549
2	15.129	MM	0.7165	9875.03223	229.70174	50.0451
Totals :				1.97323e4	510.34947	

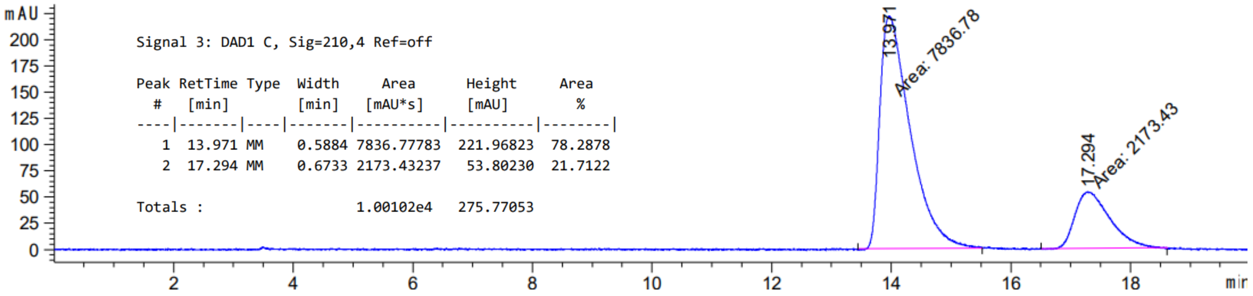

Signal 3: DAD1 C, Sig=210,4 Ref=off

Peak #	RetTime [min]	Type	Width [min]	Area [mAU*s]	Height [mAU]	Area %
1	13.971	MM	0.5884	7836.77783	221.96823	78.2878
2	17.294	MM	0.6733	2173.43237	53.80230	21.7122

Totals : 1.00102e4 275.77053

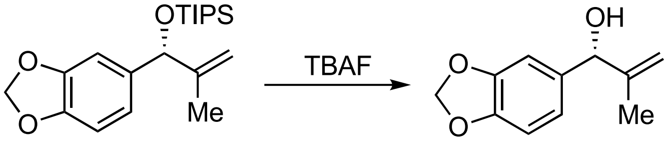

12a

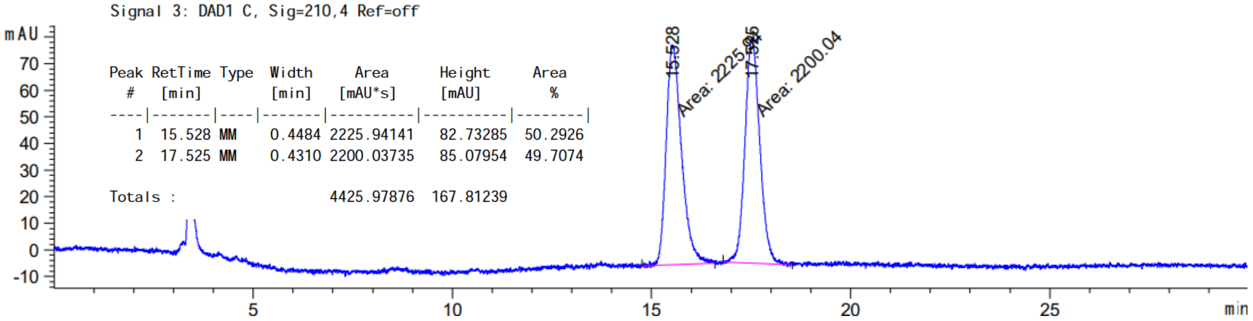

Signal 3: DAD1 C, Sig=210,4 Ref=off

Peak #	RetTime [min]	Type	Width [min]	Area [mAU*s]	Height [mAU]	Area %
1	15.528	MM	0.4484	2225.94141	82.73285	50.2926
2	17.525	MM	0.4310	2200.03735	85.07954	49.7074

Totals : 4425.97876 167.81239

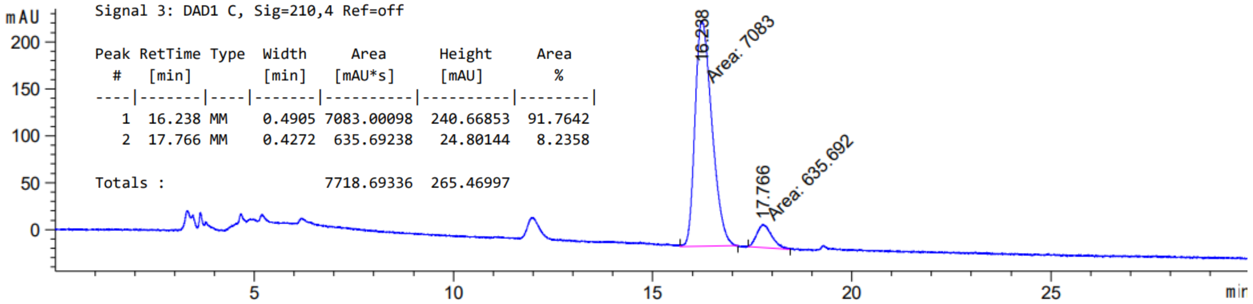

Signal 3: DAD1 C, Sig=210,4 Ref=off

Peak #	RetTime [min]	Type	Width [min]	Area [mAU*s]	Height [mAU]	Area %
1	16.238	MM	0.4905	7083.00098	240.66853	91.7642
2	17.766	MM	0.4272	635.69238	24.80144	8.2358

Totals : 7718.69336 265.46997

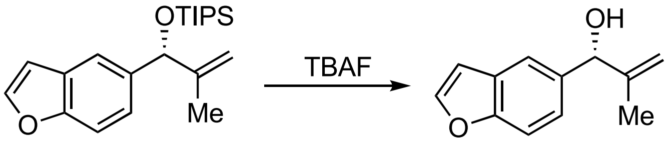

13a

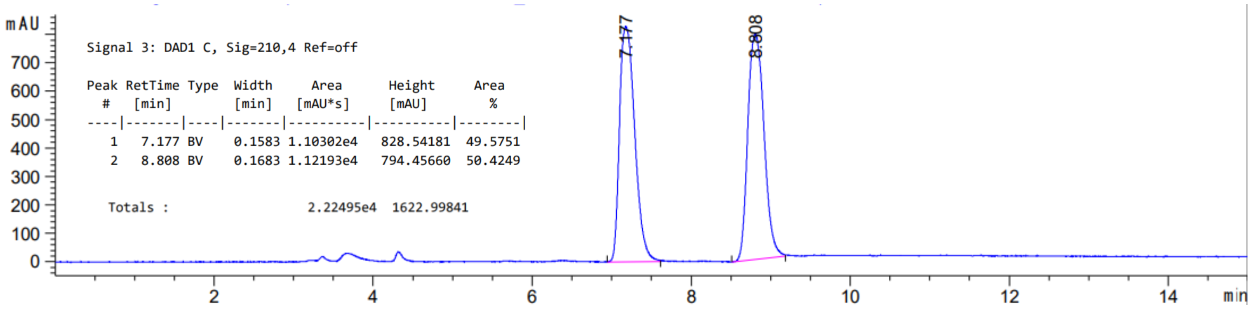

Signal 3: DAD1 C, Sig=210,4 Ref=off

Peak #	RetTime [min]	Type	Width [min]	Area [mAU*s]	Height [mAU]	Area %
1	7.177	BV	0.1583	1.10302e4	828.54181	49.5751
2	8.808	BV	0.1683	1.12193e4	794.45660	50.4249

Totals : 2.22495e4 1622.99841

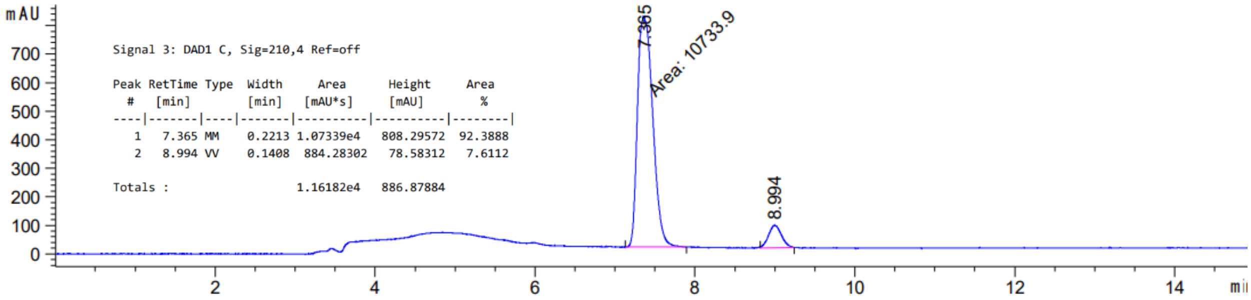
mAU
Signal 3: DAD1 C, Sig=210,4 Ref=off
Peak RetTime Type Width Area Height Area
[min] [min] [mAU*s] [mAU] %
1 7.365 MM 0.2213 1.07339e4 808.29572 92.3888
2 8.994 VV 0.1408 884.28302 78.58312 7.6112
Totals : 1.16182e4 886.87884
7.365
Area: 10733.9
8.994
min

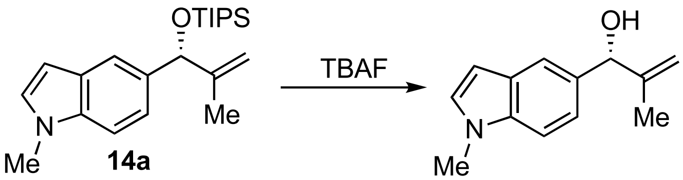
OTIPS
N
Me
Me
TBAF
OH
N
Me
Me

14a

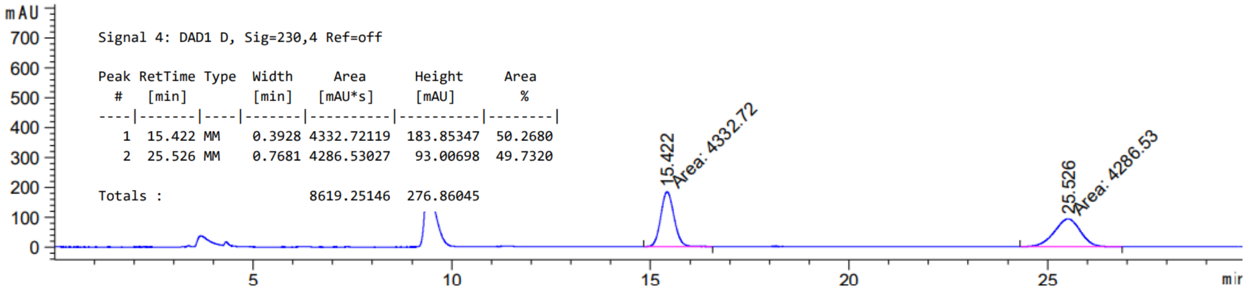
mAU
Signal 4: DAD1 D, Sig=230,4 Ref=off
Peak RetTime Type Width Area Height Area
[min] [min] [mAU*s] [mAU] %
1 15.422 MM 0.3928 4332.72119 183.85347 50.2680
2 25.526 MM 0.7681 4286.53027 93.00698 49.7320
Totals : 8619.25146 276.86045
15.422
Area: 4332.72
25.526
Area: 4286.53
min

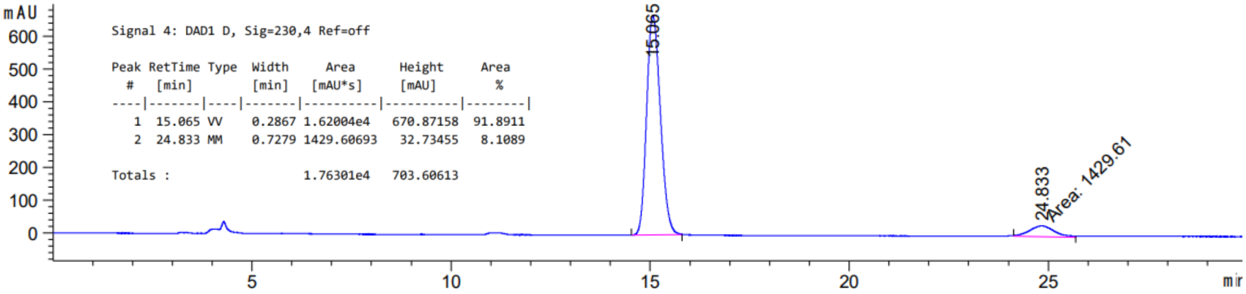
mAU
Signal 4: DAD1 D, Sig=230,4 Ref=off
Peak RetTime Type Width Area Height Area
[min] [min] [mAU*s] [mAU] %
1 15.065 VV 0.2867 1.62004e4 670.87158 91.8911
2 24.833 MM 0.7279 1429.60693 32.73455 8.1089
Totals : 1.76301e4 703.60613
15.065
24.833
Area: 1429.61
min

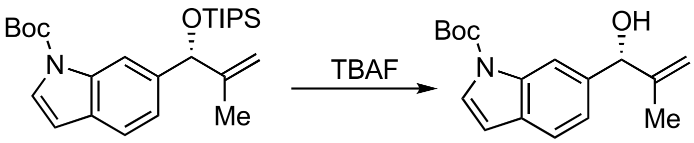
Boc
N
OTIPS
Me
TBAF
Boc
N
OH
Me

15a

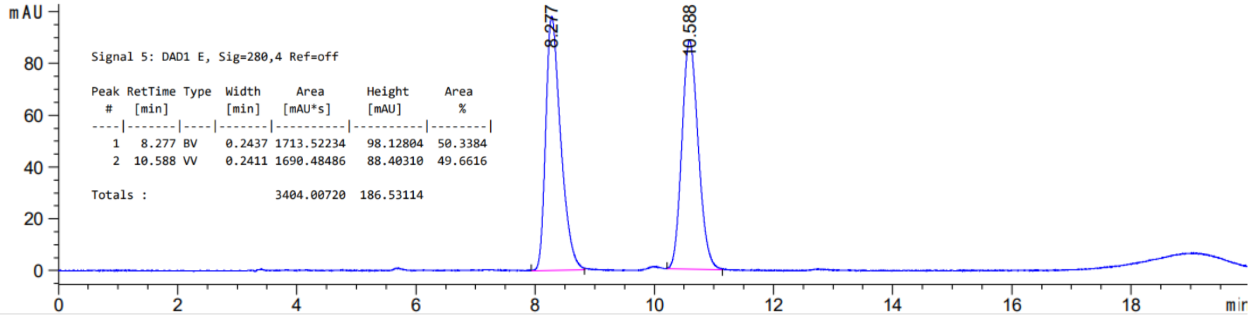
mAU
Signal 5: DAD1 E, Sig=280,4 Ref=off
Peak RetTime Type Width Area Height Area
[min] [min] [mAU*s] [mAU] %
1 8.277 BV 0.2437 1713.52234 98.12804 50.3384
2 10.588 VV 0.2411 1690.48486 88.40310 49.6616
Totals : 3404.00720 186.53114
8.277
10.588
min

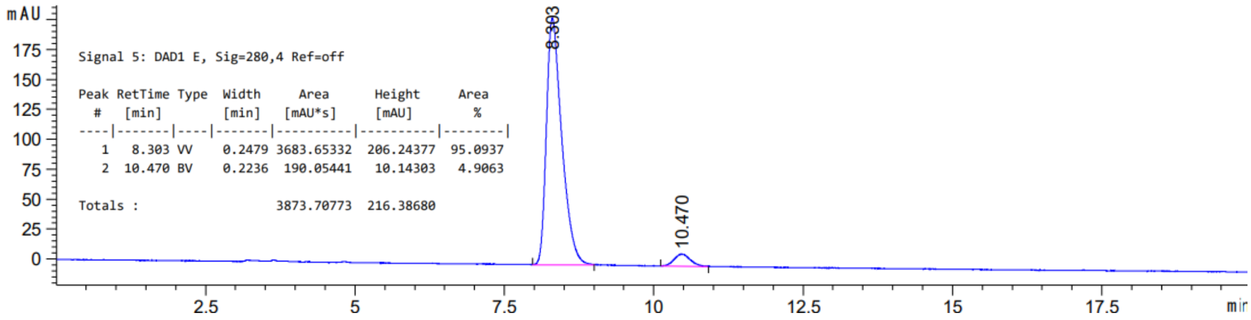
mAU
Signal 5: DAD1 E, Sig=280,4 Ref=off
Peak RetTime Type Width Area Height Area
[min] [min] [mAU*s] [mAU] %
1 8.303 VV 0.2479 3683.65332 206.24377 95.0937
2 10.470 BV 0.2236 190.05441 10.14303 4.9063
Totals : 3873.70773 216.38680
8.303
10.470
min

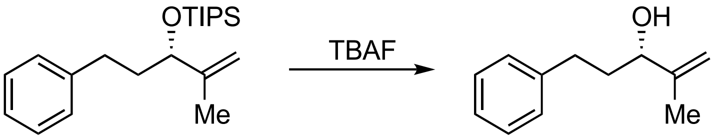
OTIPS
Me
TBAF
OH
Me

16a

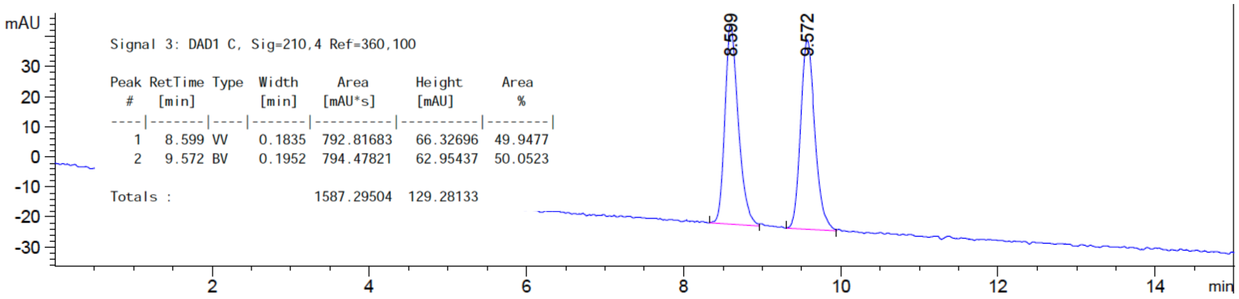
mAU
Signal 3: DAD1 C, Sig=210,4 Ref=360,100
Peak RetTime Type Width Area Height Area
[min] [min] [mAU*s] [mAU] %
1 8.599 VV 0.1835 792.81683 66.32696 49.9477
2 9.572 BV 0.1952 794.47821 62.95437 50.0523
Totals : 1587.29504 129.28133
8.599
9.572
min

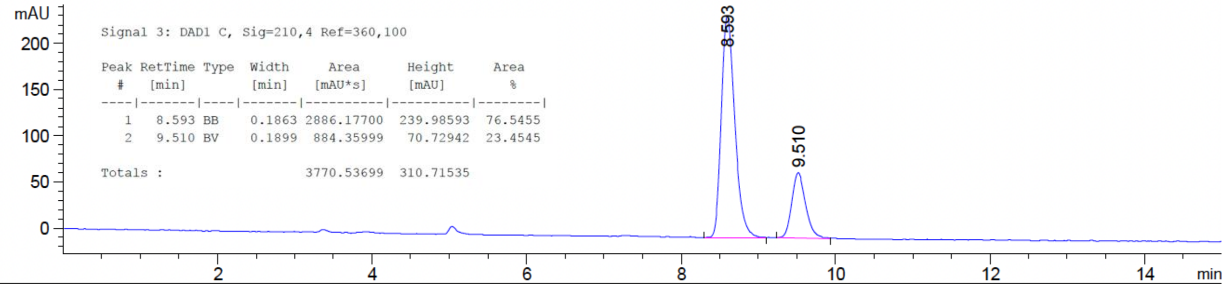
mAU
Signal 3: DAD1 C, Sig=210,4 Ref=360,100
Peak RetTime Type Width Area Height Area
[min] [min] [mAU*s] [mAU] %
1 8.593 BB 0.1863 2886.17700 239.98593 76.5455
2 9.510 BV 0.1899 884.35999 70.72942 23.4545
Totals : 3770.53699 310.71535
8.593
9.510
min

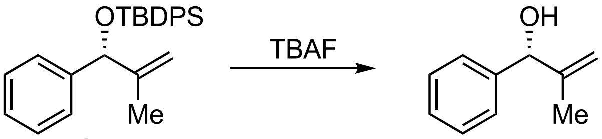
OTBDPS
Me
TBAF
OH
Me

17a

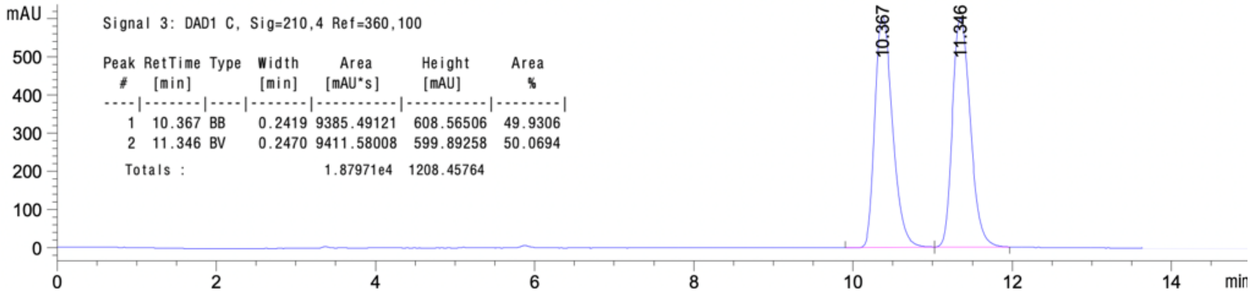
mAU
Signal 3: DAD1 C, Sig=210,4 Ref=360,100
Peak RetTime Type Width Area Height Area
[min] [min] [mAU*s] [mAU] %
1 10.367 BB 0.2419 9385.49121 608.56506 49.9306
2 11.346 BV 0.2470 9411.58008 599.89258 50.0694
Totals : 1.87971e4 1208.45764
10.367
11.346
min

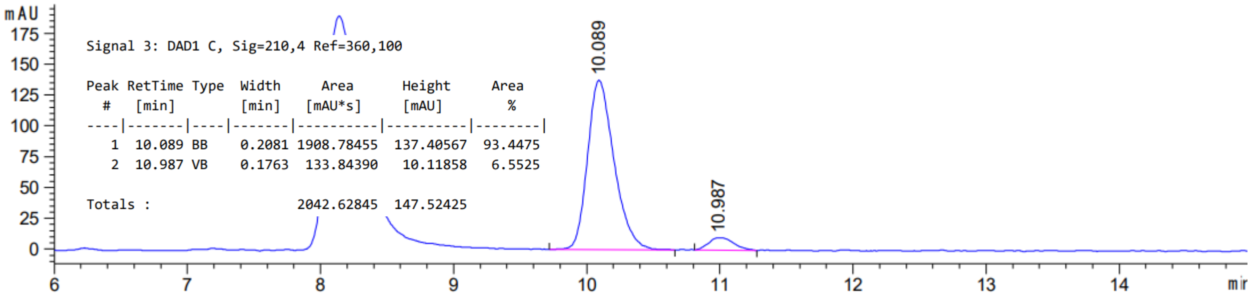
mAU
Signal 3: DAD1 C, Sig=210,4 Ref=360,100
Peak RetTime Type Width Area Height Area
[min] [min] [mAU*s] [mAU] %
1 10.089 BB 0.2081 1908.78455 137.40567 93.4475
2 10.987 VB 0.1763 133.84390 10.11858 6.5525
Totals : 2042.62845 147.52425
10.089
10.987
min

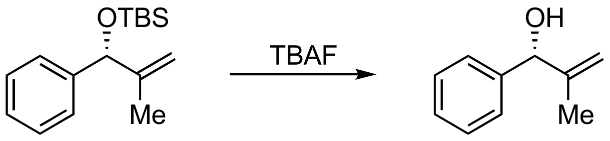
OTBS
TBAF
OH
Me
Me

18a

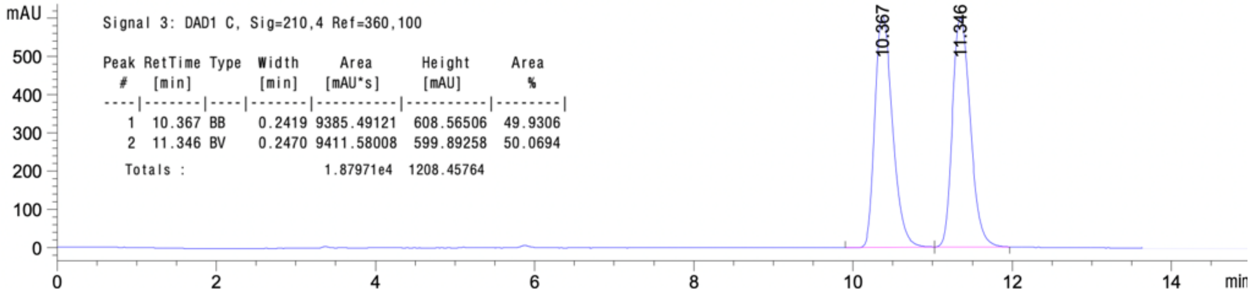
mAU
Signal 3: DAD1 C, Sig=210,4 Ref=360,100
Peak RetTime Type Width Area Height Area
[min] [min] [mAU*s] [mAU] %
1 10.367 BB 0.2419 9385.49121 608.56506 49.9306
2 11.346 BV 0.2470 9411.58008 599.89258 50.0694
Totals : 1.87971e4 1208.45764
10.367
11.346
min

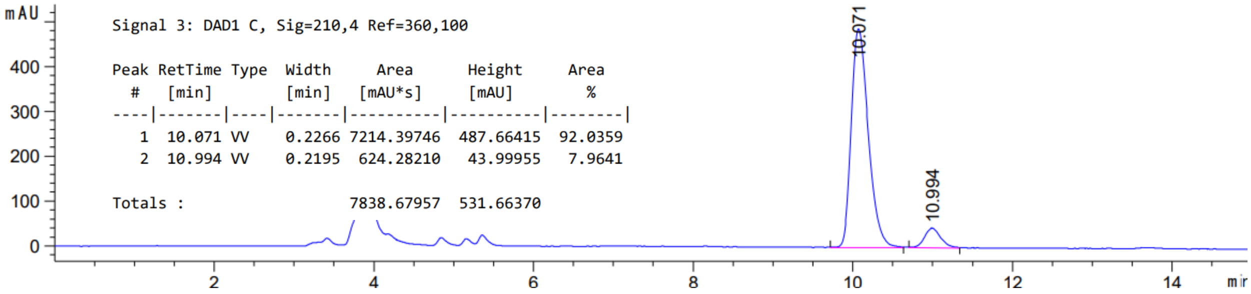
mAU
Signal 3: DAD1 C, Sig=210,4 Ref=360,100
Peak RetTime Type Width Area Height Area
[min] [min] [mAU*s] [mAU] %
1 10.071 VV 0.2266 7214.39746 487.66415 92.0359
2 10.994 VV 0.2195 624.28210 43.99955 7.9641
Totals : 7838.67957 531.66370
10.071
10.994
min

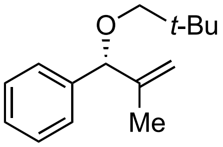
O
t-Bu
Me

19a

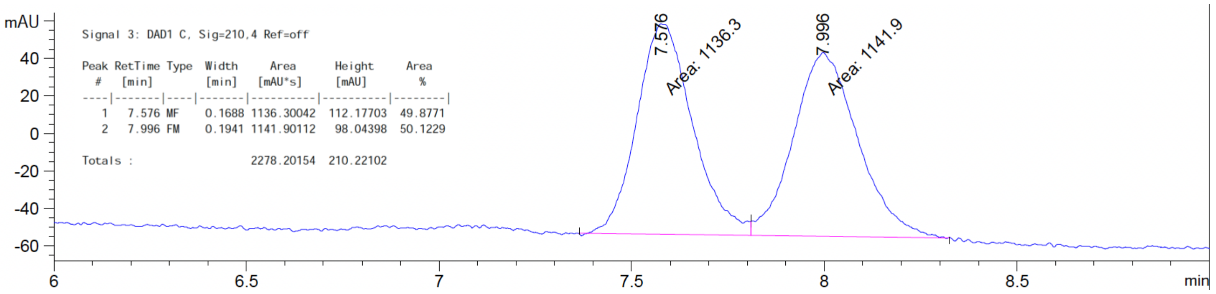
mAU
Signal 3: DAD1 C, Sig=210,4 Ref=off
Peak RetTime Type Width Area Height Area
[min] [min] [mAU*s] [mAU] %
1 7.576 MF 0.1688 1136.30042 112.17703 49.8771
2 7.996 FM 0.1941 1141.90112 98.04398 50.1229
Totals : 2278.20154 210.22102
7.576
Area: 1136.3
7.996
Area: 1141.9
min

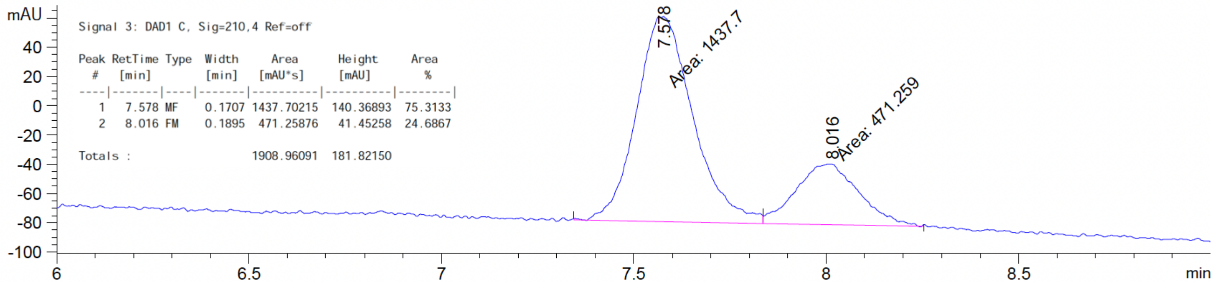
mAU
Signal 3: DAD1 C, Sig=210,4 Ref=off
Peak RetTime Type Width Area Height Area
[min] [min] [mAU*s] [mAU] %
1 7.578 MF 0.1707 1437.70215 140.36893 75.3133
2 8.016 FM 0.1895 471.25876 41.45258 24.6867
Totals : 1908.96091 181.82150
7.578
Area: 1437.7
8.016
Area: 471.259
min

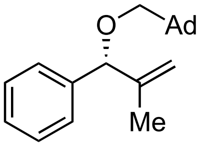

20a

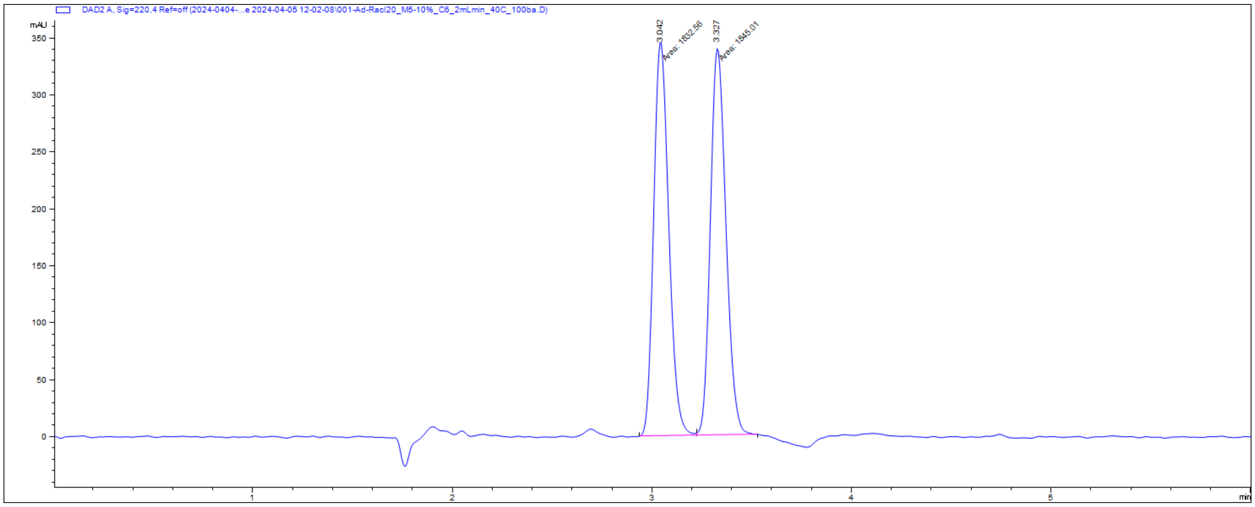

#	Time	Type	Area	Height	Width	Area%
1	3.042	MF	1832.6	348.4	0.0877	49.831
2	3.327	FM	1845	341.2	0.0901	50.169

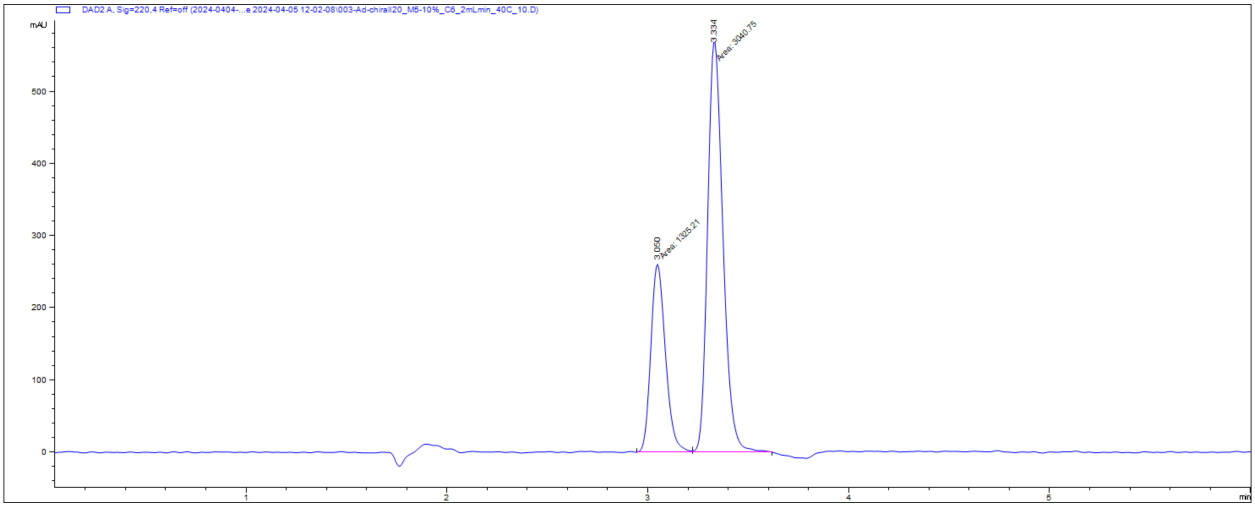

#	Time	Type	Area	Height	Width	Area%	Symmetry
1	3.05	MF	1325.2	261.4	0.0845	30.353	0.787
2	3.334	FM	3040.7	572.1	0.0886	69.647	0.758

13.References

(1) Smith, A. J.; Dimitrova, D.; Arokianathar, J. N.; Clark, K. F.; Poole, D. L.; Leach, S. G.; Murphy, J. A., Et3SiH + KOtBu provide multiple reactive intermediates that compete in the reactions and rearrangements of benzylnitriles and indolenines. *Chem. Sci.* **2020,** *11* (45), 12364-12370.

(2) Chakrabarti, K.; Maji, M.; Panja, D.; Paul, B.; Shee, S.; Das, G. K.; Kundu, S., Utilization of MeOH as a C1 Building Block in Tandem Three-Component Coupling Reaction. *Org. Lett.* **2017,** *19* (18), 4750-4753.

(3) Latham, D. E.; Polidano, K.; Williams, J. M. J.; Morrill, L. C., One-Pot Conversion of Allylic Alcohols to α-Methyl Ketones via Iron-Catalyzed Isomerization–Methylation. *Org. Lett.* **2019,** *21* (19), 7914-7918.

(4) Peng, J. B.; Chen, B.; Qi, X.; Ying, J.; Wu, X. F., Palladium - Catalyzed Carbonylative Coupling of Aryl Iodides with Alkyl Bromides: Efficient Synthesis of Alkyl Aryl Ketones. *Adv. Synth. Catal.* **2018,** *360* (21), 4153-4160.

(5) Shee, S.; Kundu, S., Rhenium(I)-Catalyzed C-Methylation of Ketones, Indoles, and Arylacetonitriles Using Methanol. *J. Org. Chem.* **2021,** *86* (9), 6943-6951.

(6) Liu, Z.; Yang, Z.; Yu, X.; Zhang, H.; Yu, B.; Zhao, Y.; Liu, Z., Methylation of C(sp3)–H/C(sp2)–H Bonds with Methanol Catalyzed by Cobalt System. *Org. Lett.* **2017,** *19* (19), 5228-5231.

(7) Zhao, K.; Knowles, R. R., Contra-Thermodynamic Positional Isomerization of Olefins. *J. Am. Chem. Soc.* **2021,** *144* (1), 137-144.

(8) Swyka, R. A.; Shuler, W. G.; Spinello, B. J.; Zhang, W.; Lan, C.; Krische, M. J., Conversion of Aldehydes to Branched or Linear Ketones via Regiodivergent Rhodium-Catalyzed Vinyl Bromide Reductive Coupling–Redox Isomerization Mediated by Formate. *J. Am. Chem. Soc.* **2019,** *141* (17), 6864-6868.

(9) Walter, A.; Eisenreich, W.; Storch, G., Photochemical Desaturation and Epoxidation with Oxygen by Sequential Flavin Catalysis. *Angew. Chem. Int. Ed.* **2023,** *62* (42).

(10) Choi, G. J.; Zhu, Q.; Miller, D. C.; Gu, C. J.; Knowles, R. R., Catalytic alkylation of remote C–H bonds enabled by proton-coupled electron transfer. *Nature* **2016,** *539* (7628), 268-271.

(11) Lowry, M. S.; Goldsmith, J. I.; Slinker, J. D.; Rohl, R.; Pascal, R. A.; Malliaras, G. G.; Bernhard, S., Single-Layer Electroluminescent Devices and Photoinduced Hydrogen Production from an Ionic Iridium(III) Complex. *Chemistry of Materials* **2005,** *17* (23), 5712-5719.

(12) Rohe, S.; Morris, A. O.; McCallum, T.; Barriault, L., Hydrogen Atom Transfer Reactions via Photoredox Catalyzed Chlorine Atom Generation. *Angew. Chem. Int. Ed.* **2018,** *57* (48), 15664-15669.

(13) Apolinar, O.; Kang, T.; Alturaifi, T. M.; Bedekar, P. G.; Rubel, C. Z.; Derosa, J.; Sanchez, B. B.; Wong, Q. N.; Sturgell, E. J.; Chen, J. S.; Wisniewski, S. R.; Liu, P.; Engle, K. M., Three-Component Asymmetric Ni-Catalyzed 1,2-Dicarbofunctionalization of Unactivated Alkenes via Stereoselective Migratory Insertion. *J. Am. Chem. Soc.* **2022,** *144* (42), 19337-19343.

(14) MacroModel, version 11.8, Schrödinger, LLC, New York, NY, 2017.

(15) Harder, E.; Damm, W.; Maple, J.; Wu, C.; Reboul, M.; Xiang, J. Y.; Wang, L.; Lupyan, D.; Dahlgren, M. K.; Knight, J. L.; Kaus, J. W.; Cerutti, D. S.; Krilov, G.; Jorgensen, W. L.; Abel, R.; Friesner, R. A., OPLS3: A Force Field Providing Broad Coverage of Drug-like Small Molecules and Proteins. *Journal of Chemical Theory and Computation* **2015,** *12* (1), 281-296.

(16) Gaussian 16, Revision A.03, M. J. Frisch, G. W. Trucks, H. B. Schlegel, G. E. Scuseria, M. A. Robb, J. R. Cheeseman, G. Scalmani, V. Barone, G. A. Petersson, H. Nakatsuji, X. Li, M. Caricato, A. V. Marenich, J. Bloino, B. G. Janesko, R. Gomperts, B. Mennucci, H. P. Hratchian, J. V. Ortiz, A. F. Izmaylov, J. L. Sonnenberg, D. Williams-Young, F. Ding, F. Lipparini, F. Egidi, J. Goings, B. Peng, A. Petrone, T. Henderson, D. Ranasinghe, V. G. Zakrzewski, J. Gao, N. Rega, G. Zheng, W. Liang, M. Hada, M. Ehara, K. Toyota, R. Fukuda, J. Hasegawa, M. Ishida, T. Nakajima, Y. Honda, O. Kitao, H. Nakai, T. Vreven, K. Throssell, J. A. Montgomery, Jr., J. E. Peralta, F. Ogliaro, M. J. Bearpark, J. J. Heyd, E. N. Brothers, K. N. Kudin, V. N. Staroverov, T. A. Keith, R.

Kobayashi, J. Normand, K. Raghavachari, A. P. Rendell, J. C. Burant, S. S. Iyengar, J. Tomasi, M. Cossi, J. M. Millam, M. Klene, C. Adamo, R. Cammi, J. W. Ochterski, R. L. Martin, K. Morokuma, O. Farkas, J. B. Foresman, and D. J. Fox, Gaussian, Inc., Wallingford CT, 2016.

(17) Warren, J.J.; Tronic, T. A.; Mayer, J. M. Thermochemistry of Proton-Coupled Electron Transfer Reagents and its Implications. *Chem. Rev.* **2010**, 110(12), 6961-7001.
